# Supplementary figures and images for: Replisome loading reduces chromatin motion independent of DNA synthesis (part 1 of 2)
Source: eLife. 2023 Oct 31;12:RP87572. doi: 10.7554/eLife.87572 (PMC10617993; doi:10.7554/eLife.87572)

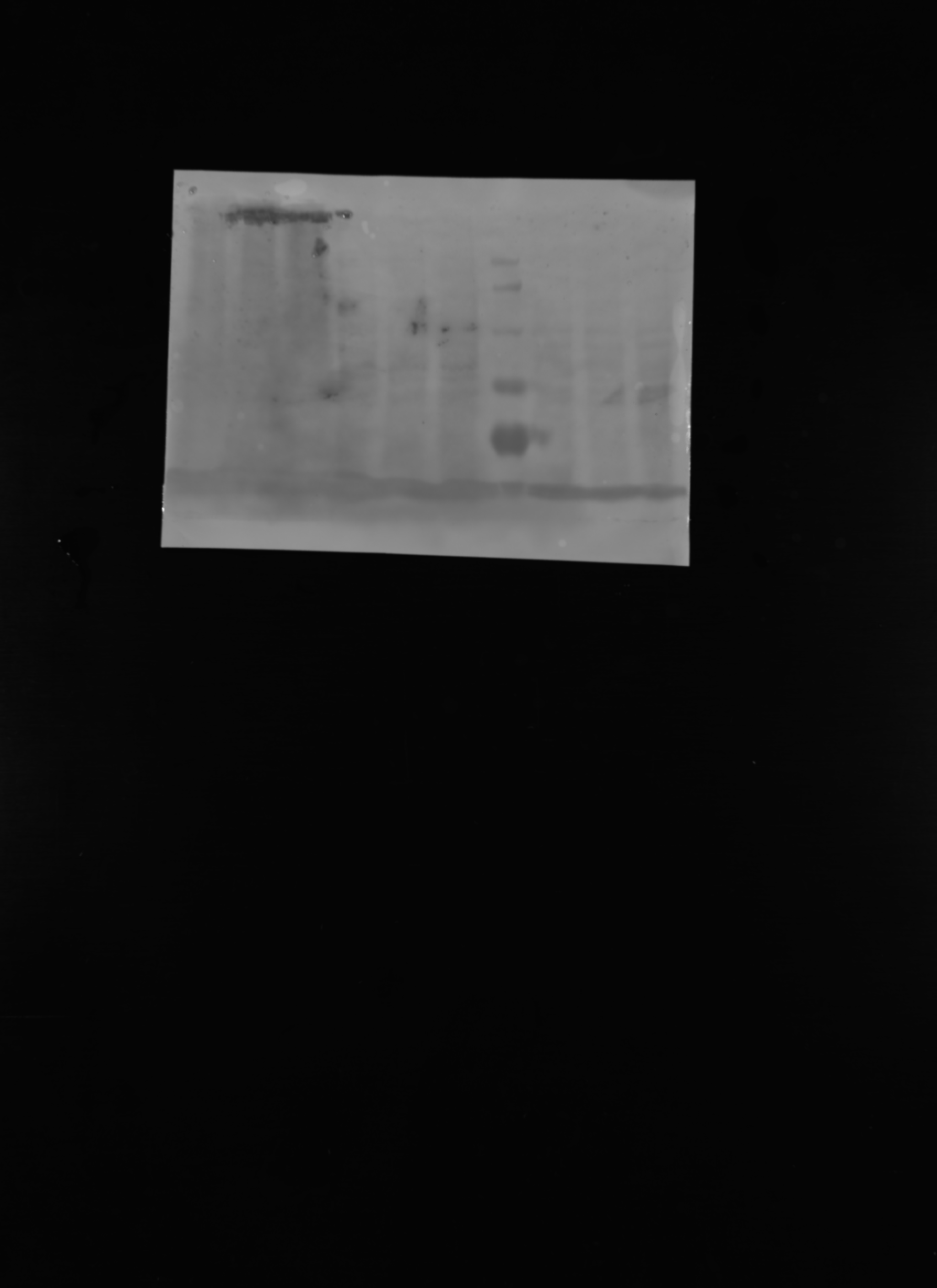

Supplement: Figure 5—source data 1. [file elife-87572-fig5-data1.zip › MCM2/Rep2/Z3-P-MCM2 2022.11.29_16.26.35_Co/Z3-P-MCM2 2022.11.29_16.26.35_Co.tif]

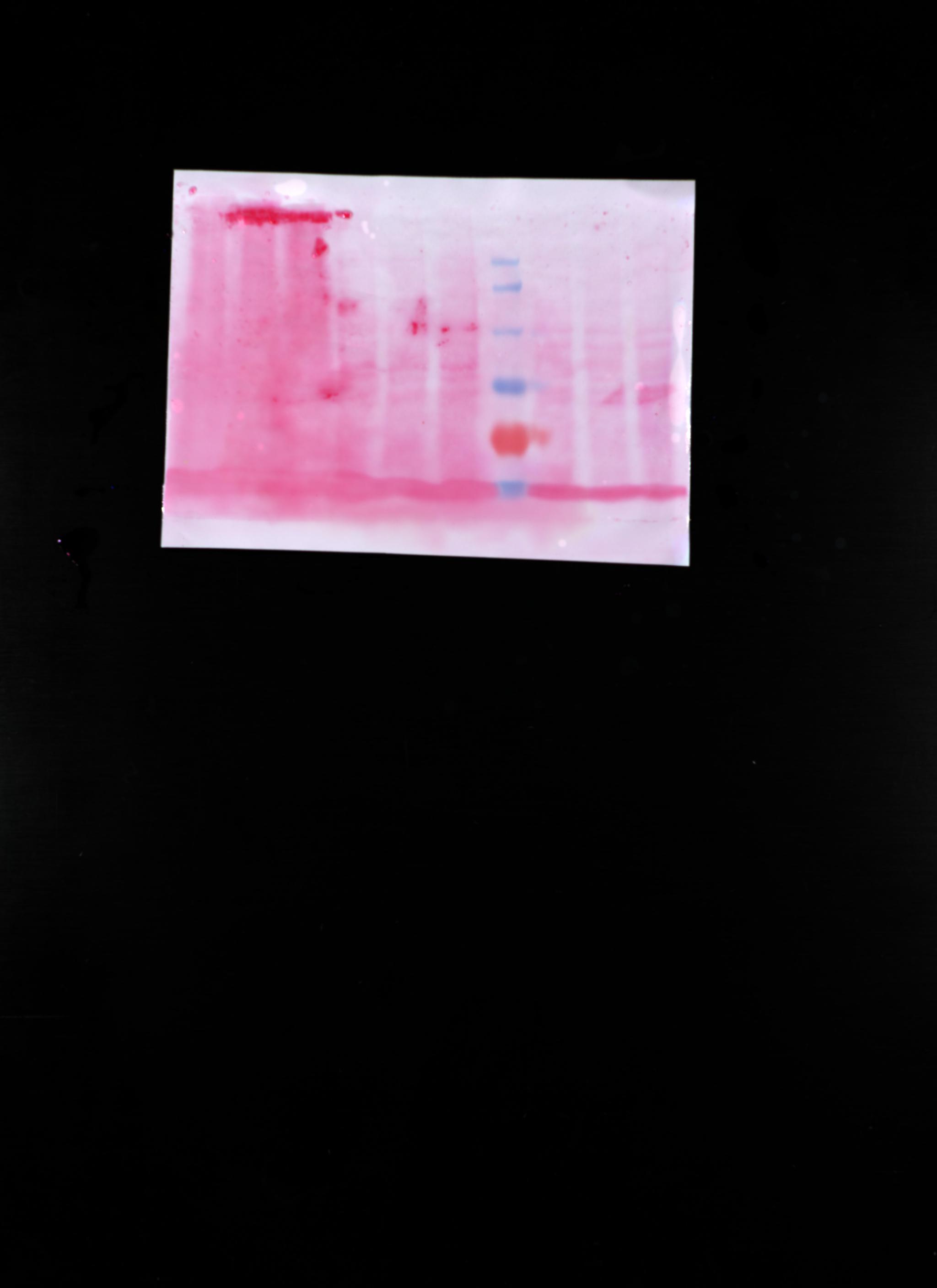

Supplement: Figure 5—source data 1. [file elife-87572-fig5-data1.zip › MCM2/Rep2/Z3-P-MCM2 2022.11.29_16.26.35_Co/Z3-P-MCM2 2022.11.29_16.26.35_Co.jpg]

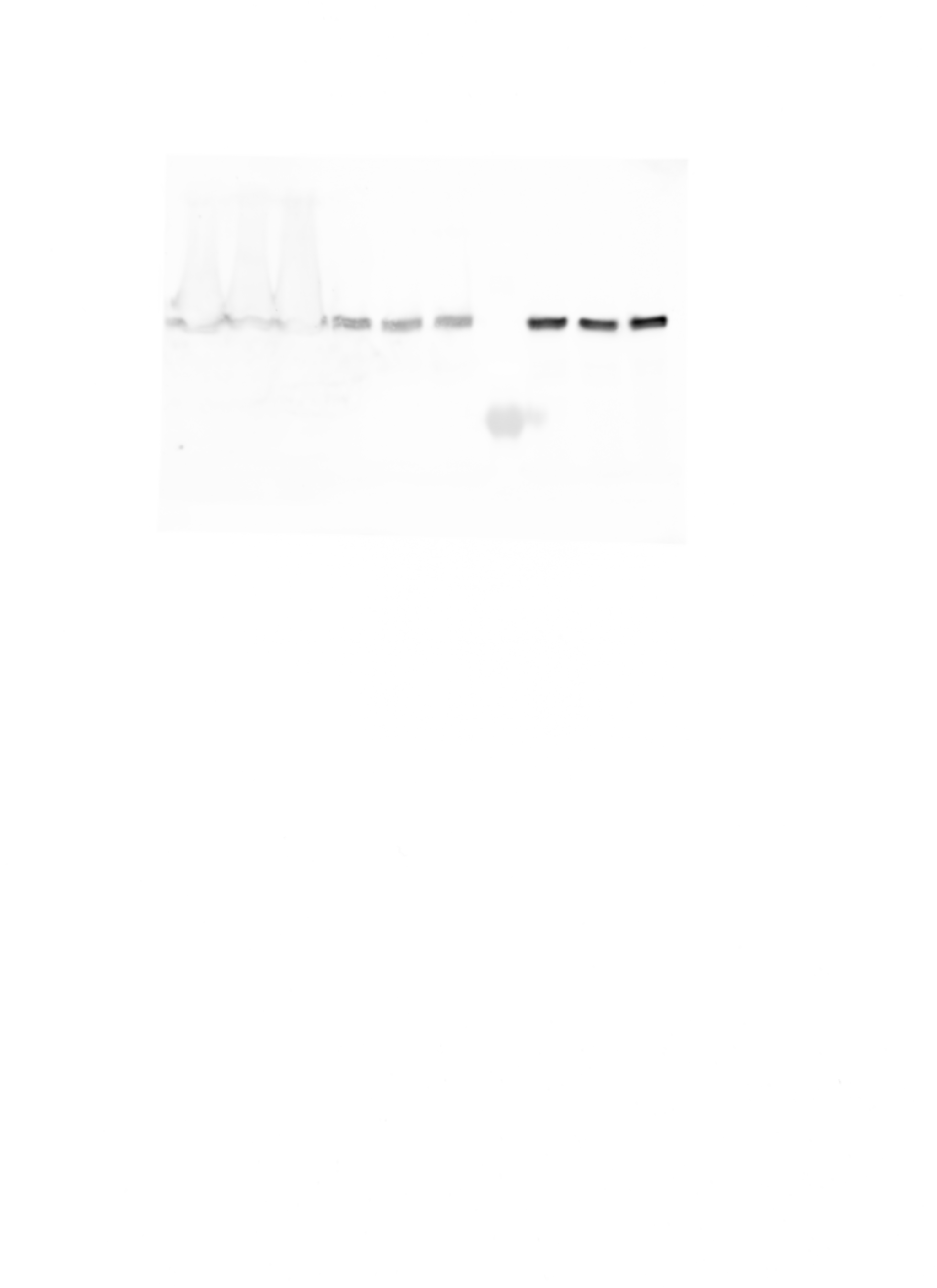

Supplement: Figure 5—source data 1. [file elife-87572-fig5-data1.zip › MCM2/Rep2/Z3-MCM2-Cy3 2022.11.30_15.45.34_Fl-Green/Z3-MCM2-Cy3 2022.11.30_15.45.34_Fl-Green.tif]

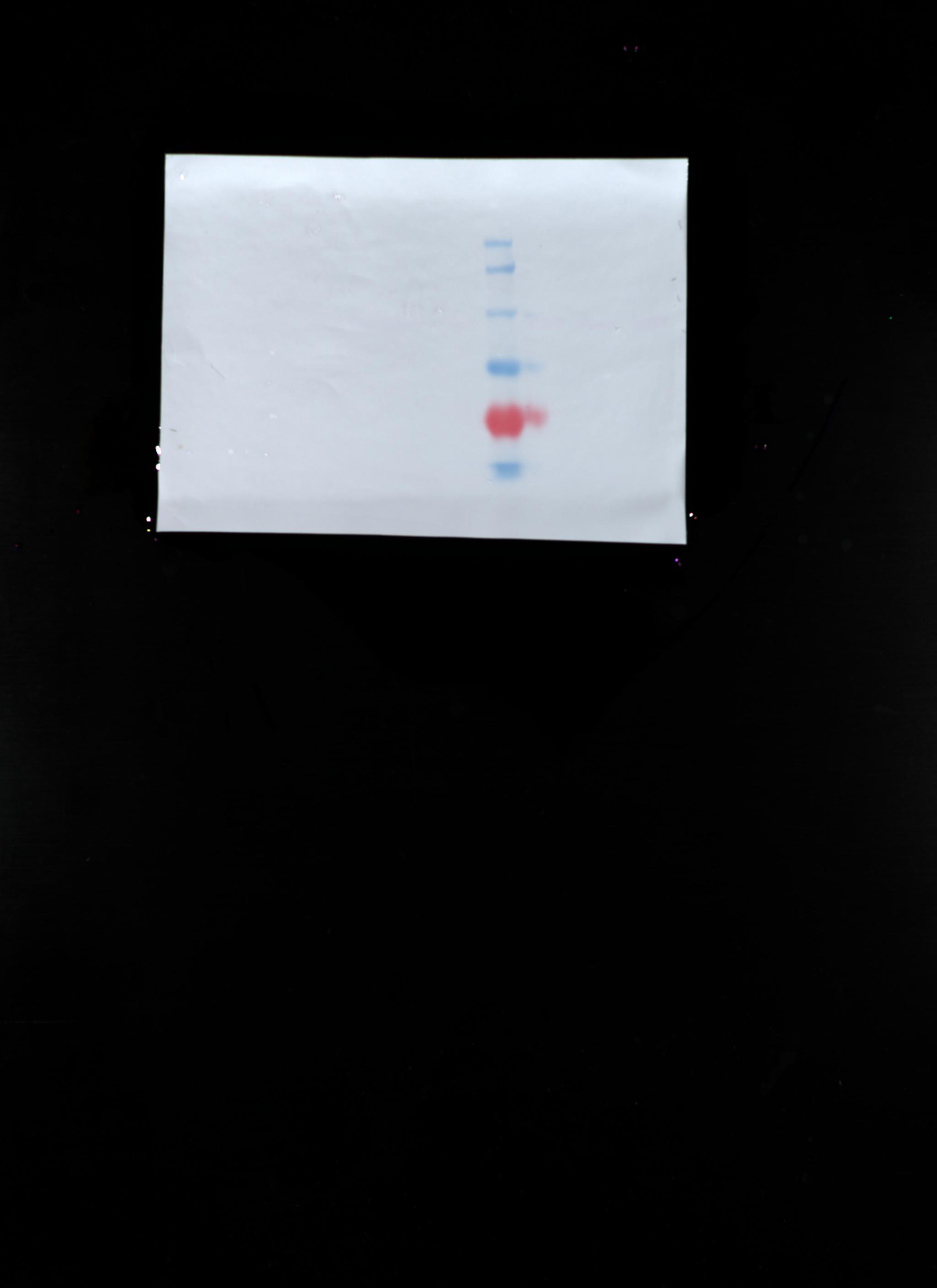

Supplement: Figure 5—source data 1. [file elife-87572-fig5-data1.zip › MCM2/Rep2/Z3-MCM2-Cy3 2022.11.30_15.45.34_Fl-Green/Z3-MCM2-Cy3 2022.11.30_15.45.34_Fl-Green-Marker.jpg]

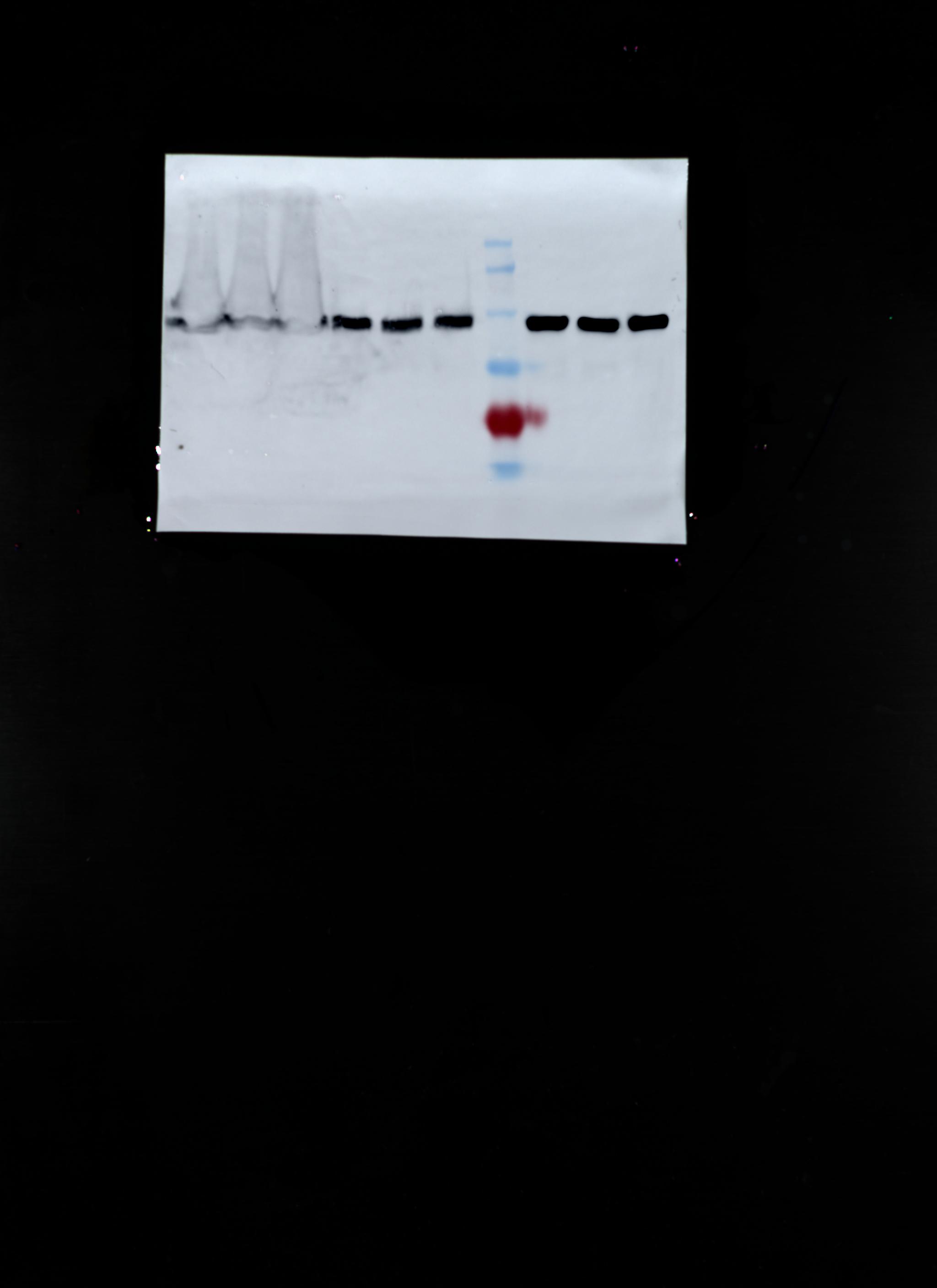

Supplement: Figure 5—source data 1. [file elife-87572-fig5-data1.zip › MCM2/Rep2/Z3-MCM2-Cy3 2022.11.30_15.45.34_Fl-Green/Z3-MCM2-Cy3 2022.11.30_15.45.34_Fl-Green+Marker.jpg]

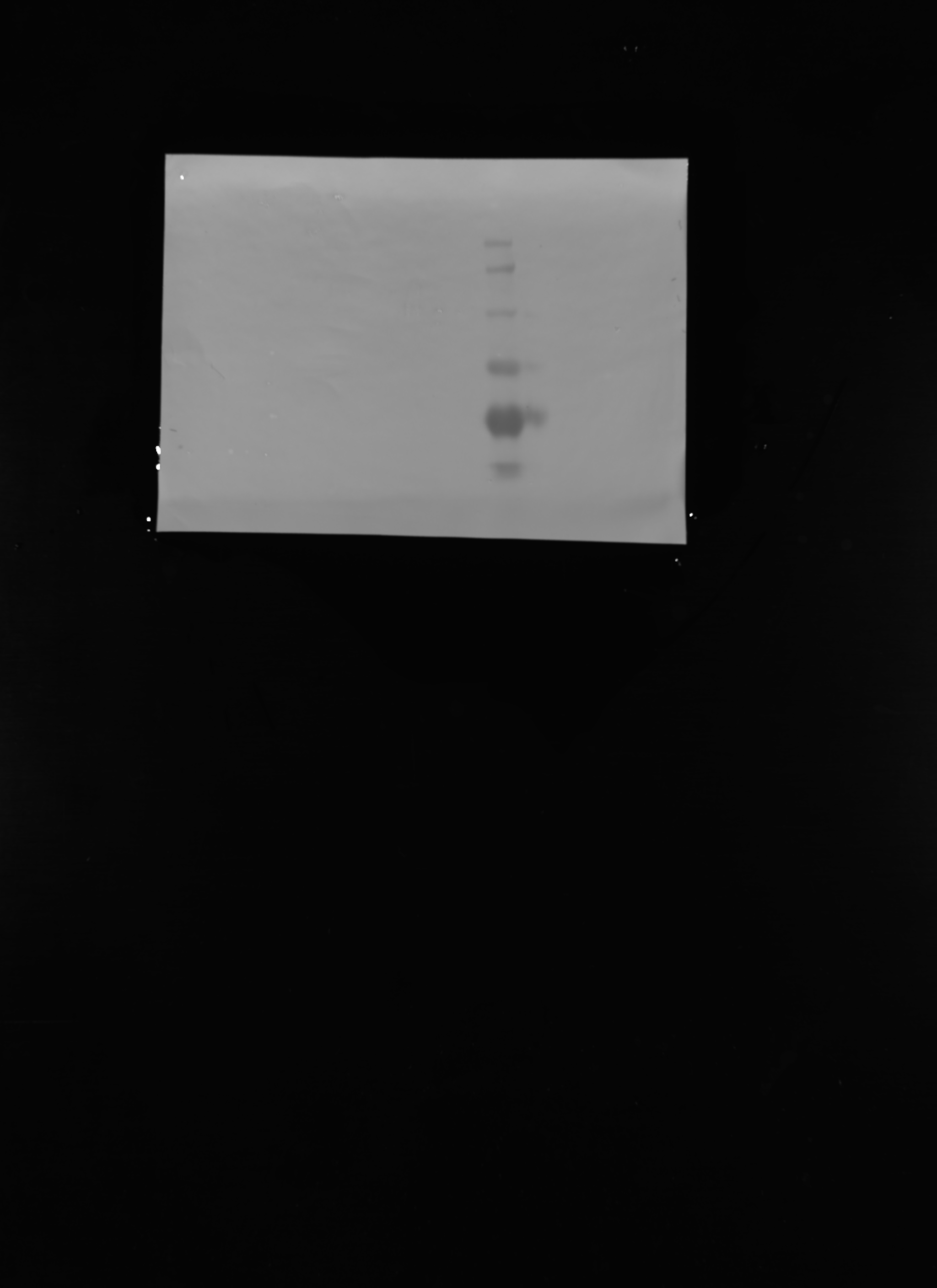

Supplement: Figure 5—source data 1. [file elife-87572-fig5-data1.zip › MCM2/Rep2/Z3-MCM2-Cy3 2022.11.30_15.45.34_Fl-Green/Z3-MCM2-Cy3 2022.11.30_15.45.34_Fl-Green-Marker.tif]

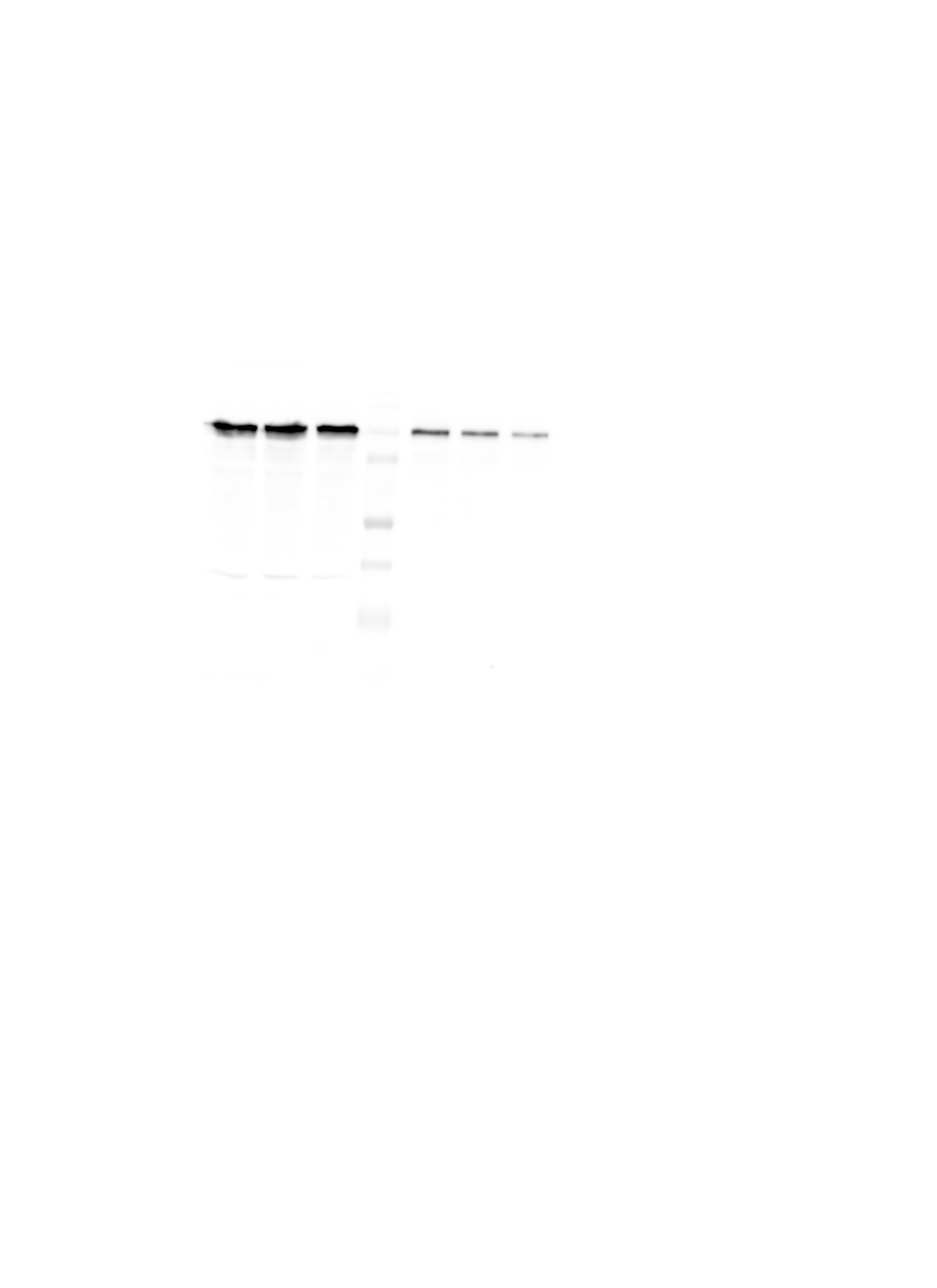

Supplement: Figure 5—source data 1. [file elife-87572-fig5-data1.zip › MCM2/Rep3/cdc45 mcm2 rfc2 2022.08.02_12.05.31_Fl/cdc45 mcm2 rfc2 2022.08.02_12.05.31_Fl-Red.tif]

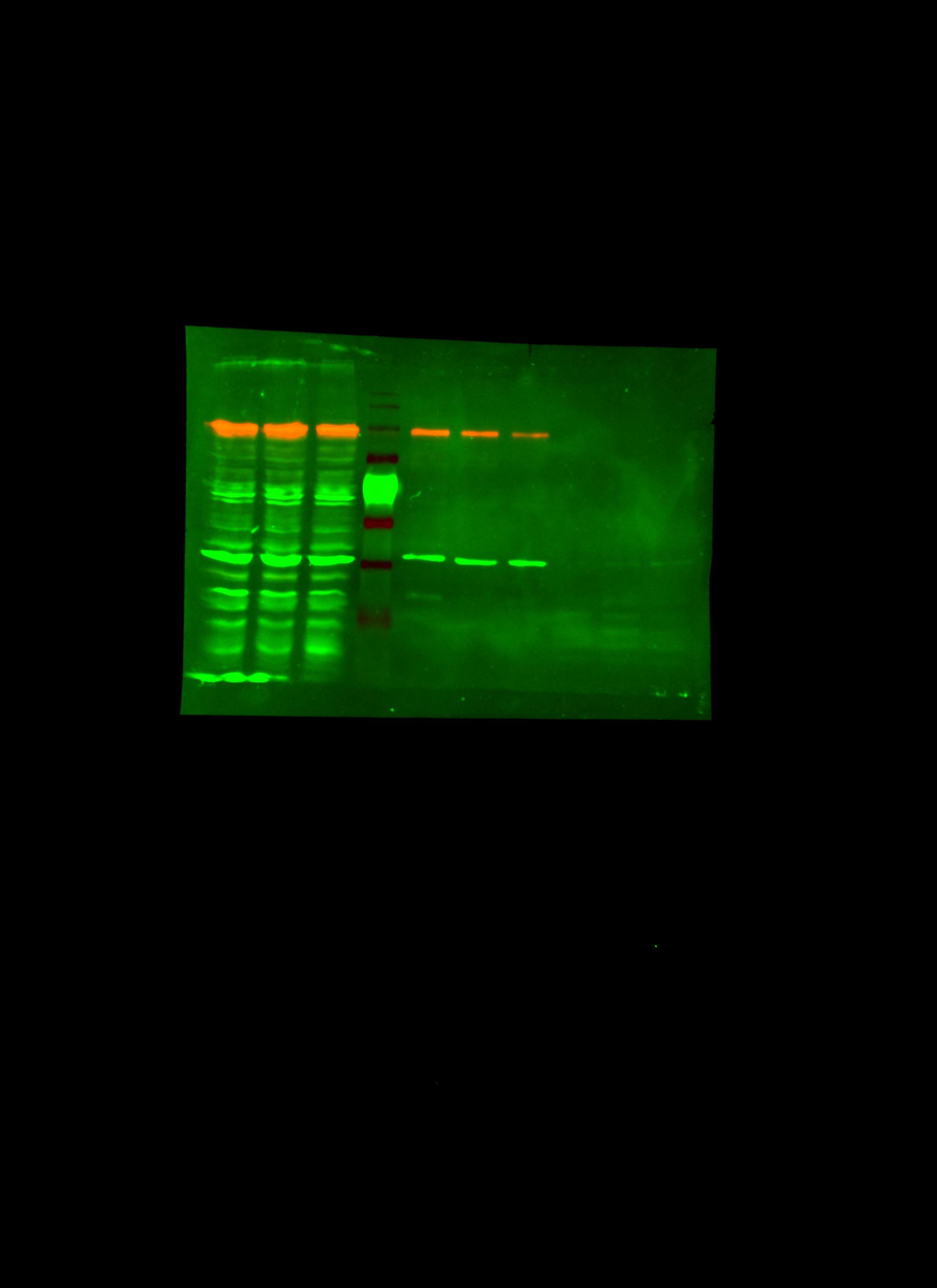

Supplement: Figure 5—source data 1. [file elife-87572-fig5-data1.zip › MCM2/Rep3/cdc45 mcm2 rfc2 2022.08.02_12.05.31_Fl/cdc45 mcm2 rfc2 2022.08.02_12.05.31_Fl.jpg]

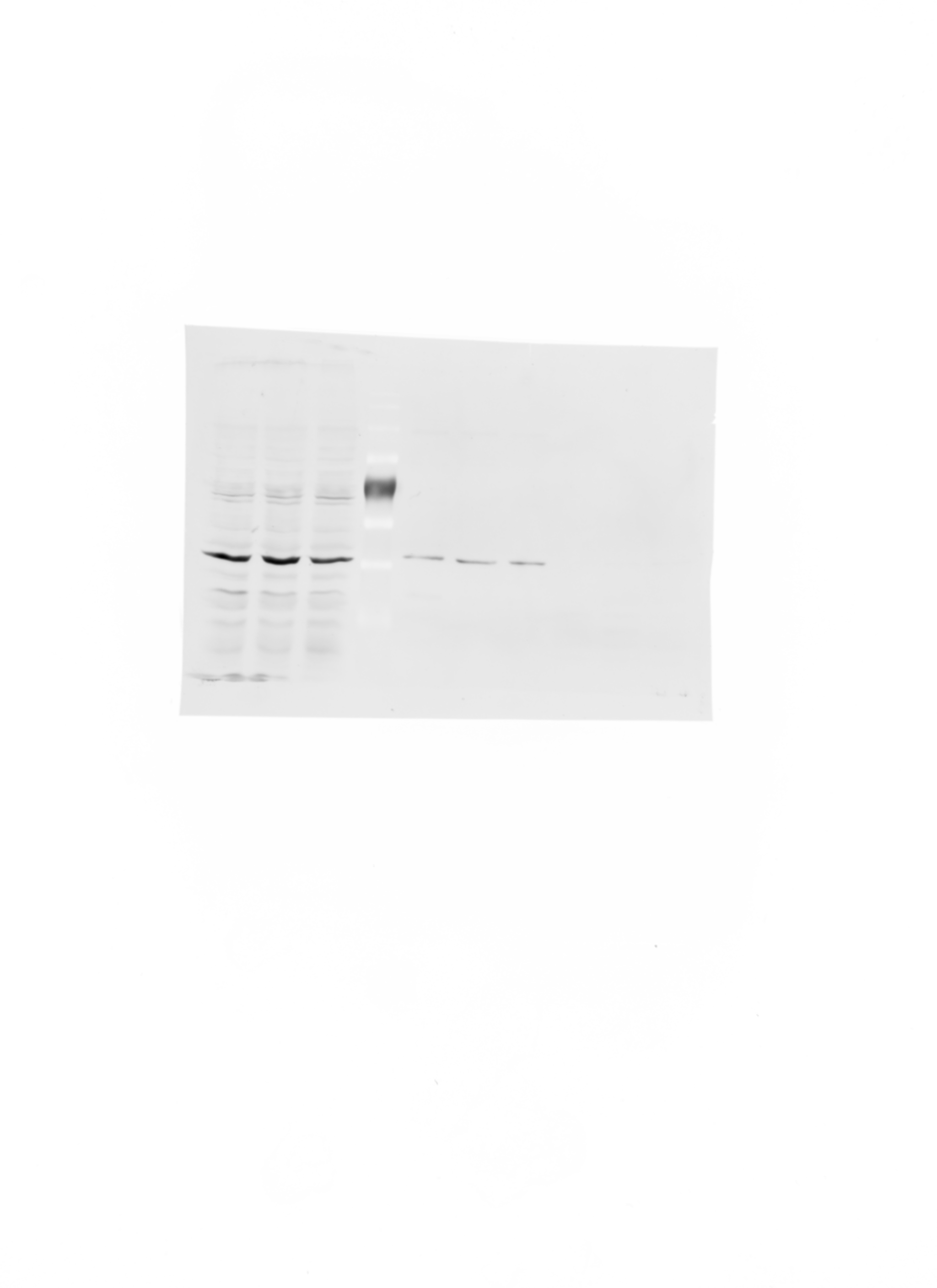

Supplement: Figure 5—source data 1. [file elife-87572-fig5-data1.zip › MCM2/Rep3/cdc45 mcm2 rfc2 2022.08.02_12.05.31_Fl/cdc45 mcm2 rfc2 2022.08.02_12.05.31_Fl-Green.tif]

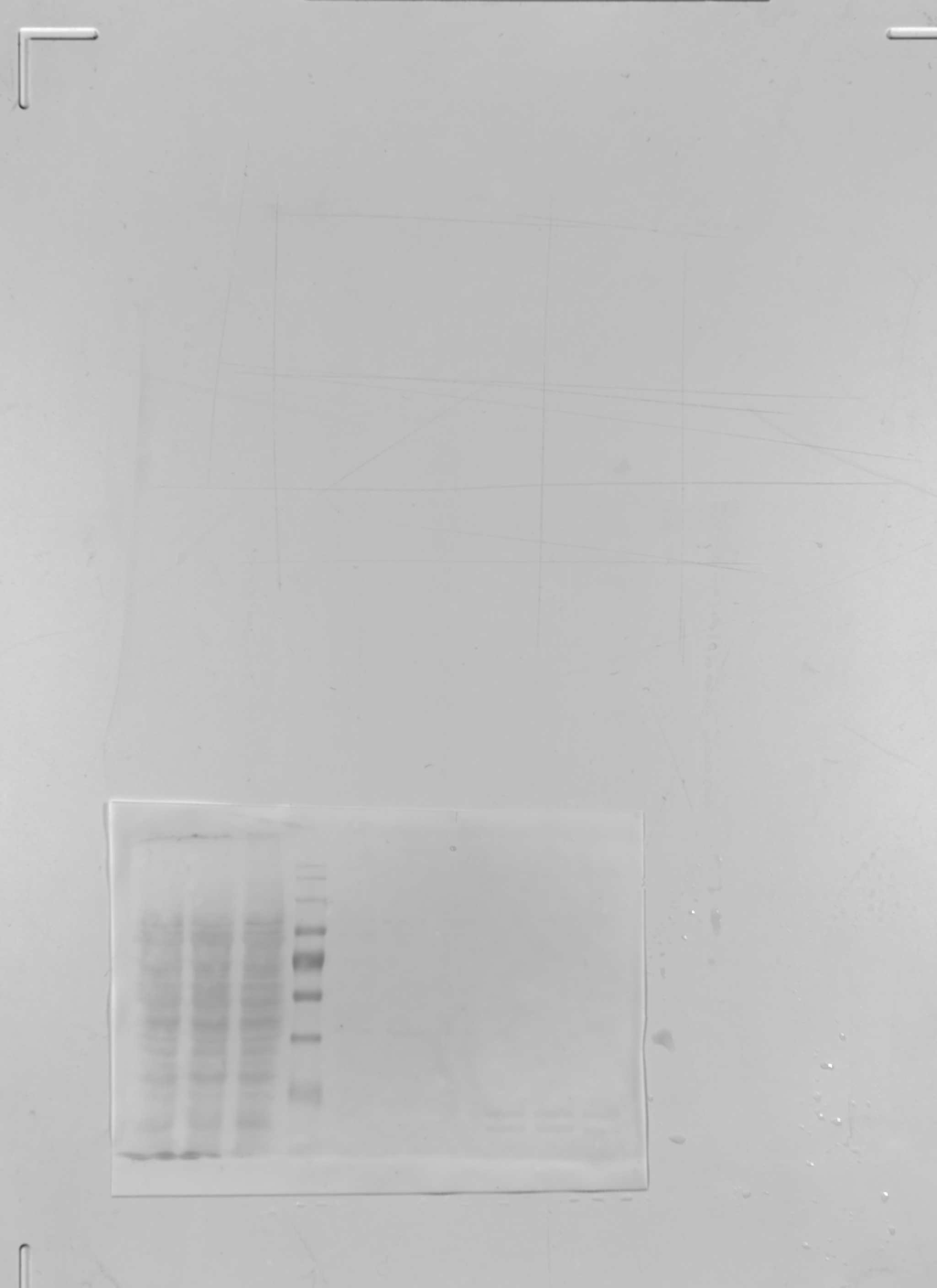

Supplement: Figure 5—source data 1. [file elife-87572-fig5-data1.zip › MCM2/Rep3/cdc45 mcm2 rfc2 ponceau 2022.08.01_13.30.26_Co/cdc45 mcm2 rfc2 ponceau 2022.08.01_13.30.26_Co.tif]

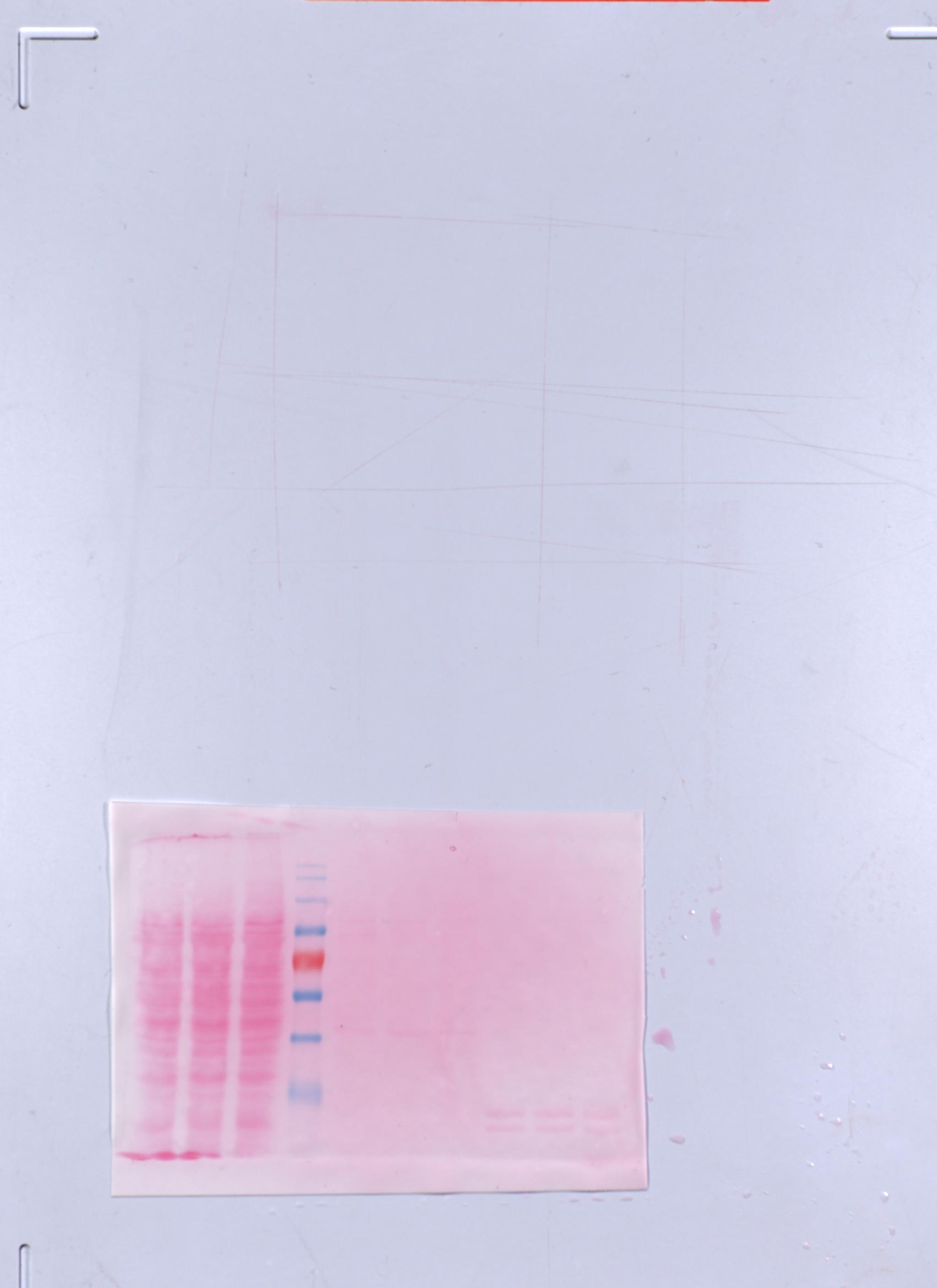

Supplement: Figure 5—source data 1. [file elife-87572-fig5-data1.zip › MCM2/Rep3/cdc45 mcm2 rfc2 ponceau 2022.08.01_13.30.26_Co/cdc45 mcm2 rfc2 ponceau 2022.08.01_13.30.26_Co.jpg]

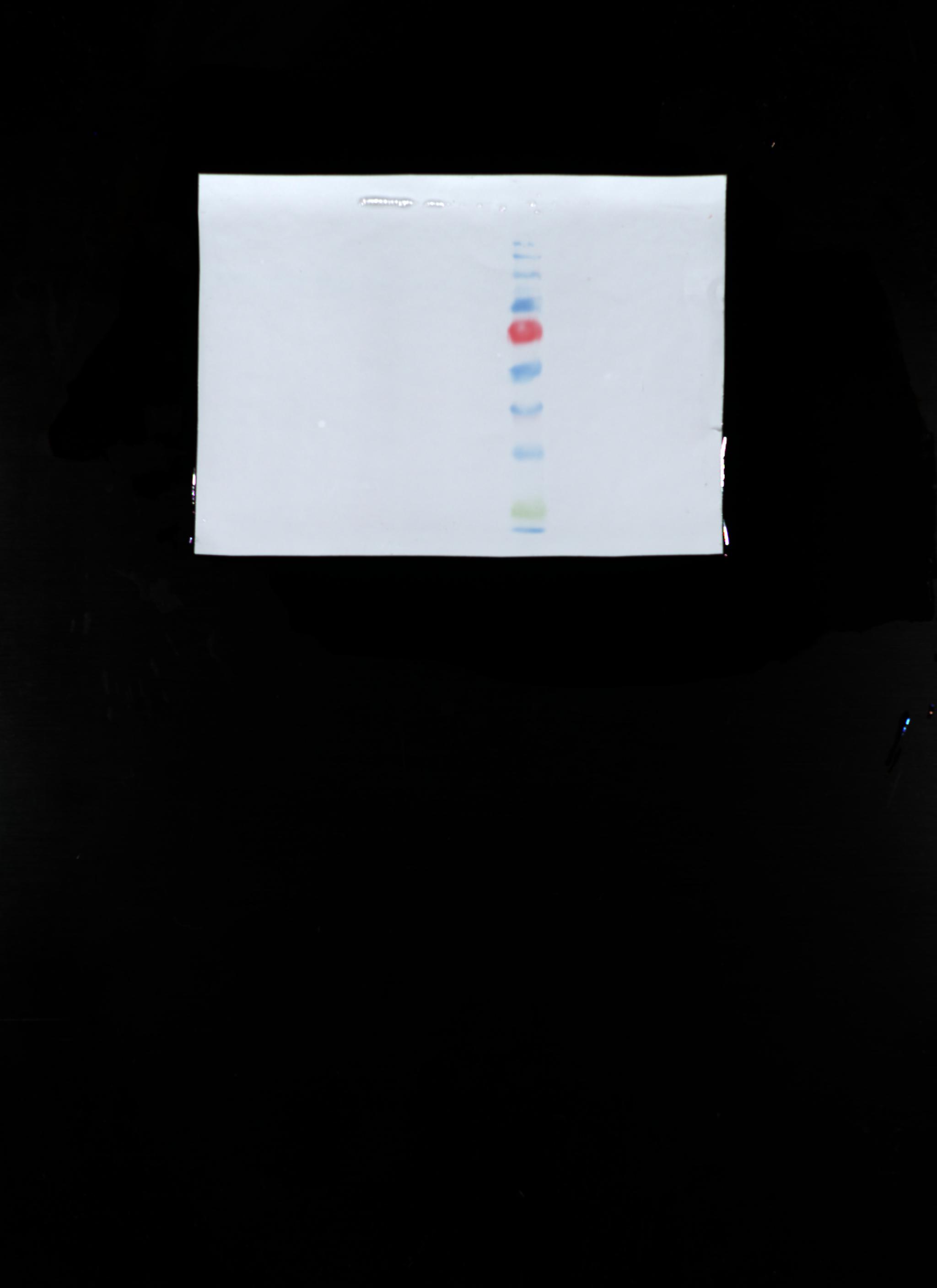

Supplement: Figure 5—source data 1. [file elife-87572-fig5-data1.zip › MCM2/Rep1/G4-mcmcy5-h2a1c5-0.5 2022.11.19_16.58.26_Fl-Red/G4-mcmcy5-h2a1c5-0.5 2022.11.19_16.58.26_Fl-Red-Marker.jpg]

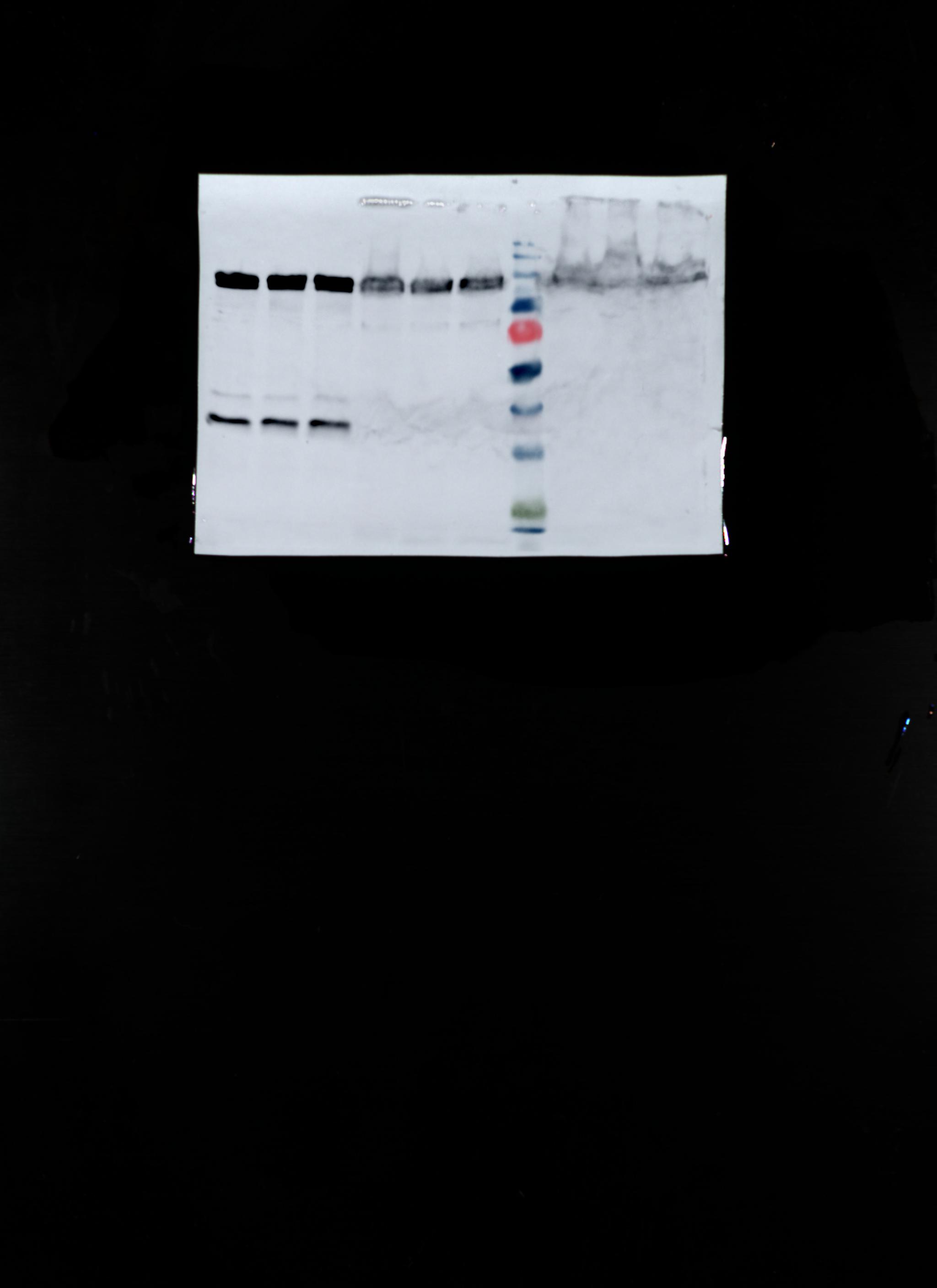

Supplement: Figure 5—source data 1. [file elife-87572-fig5-data1.zip › MCM2/Rep1/G4-mcmcy5-h2a1c5-0.5 2022.11.19_16.58.26_Fl-Red/G4-mcmcy5-h2a1c5-0.5 2022.11.19_16.58.26_Fl-Red+Marker.jpg]

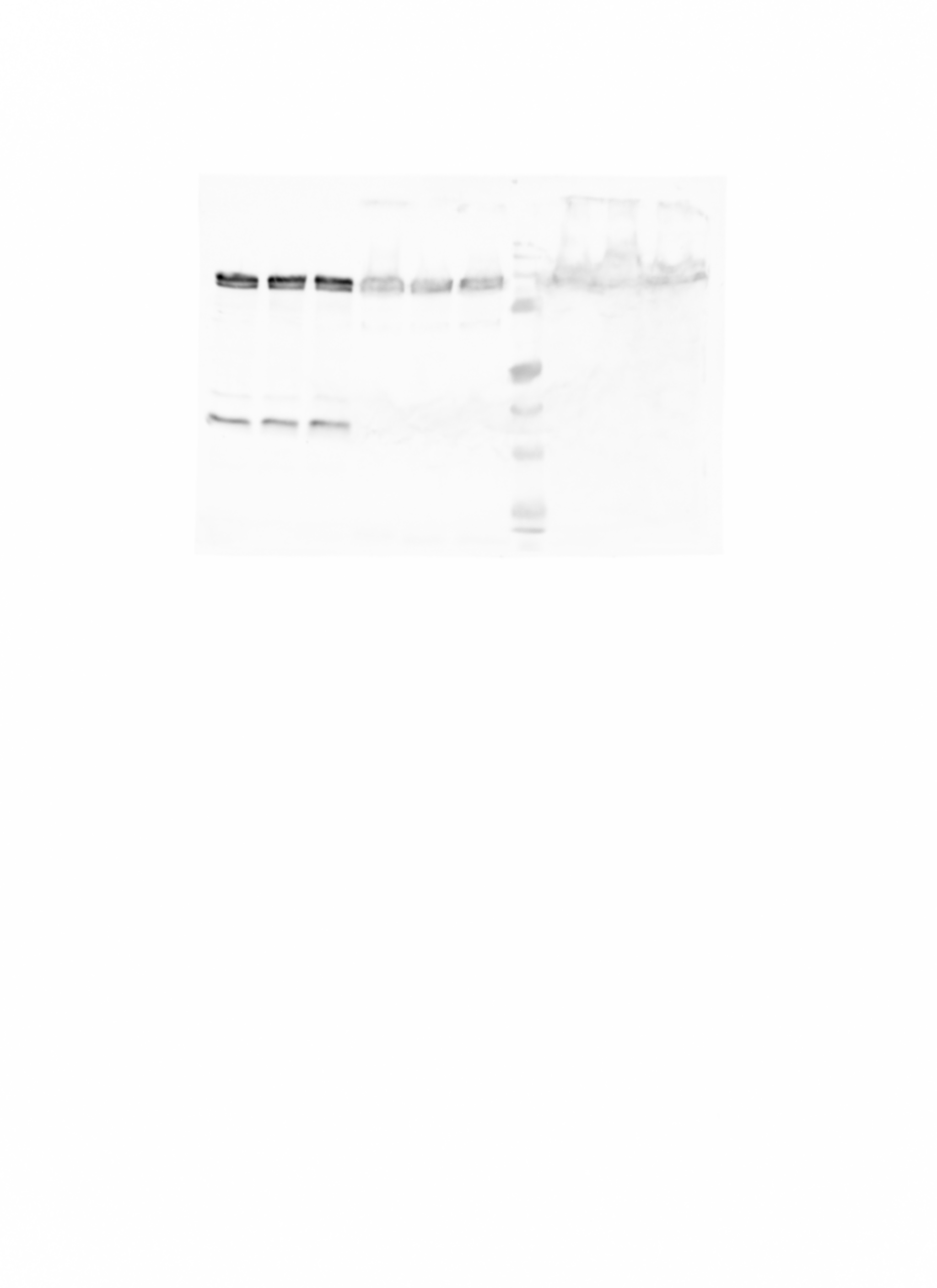

Supplement: Figure 5—source data 1. [file elife-87572-fig5-data1.zip › MCM2/Rep1/G4-mcmcy5-h2a1c5-0.5 2022.11.19_16.58.26_Fl-Red/G4-mcmcy5-h2a1c5-0.5 2022.11.19_16.58.26_Fl-Red.tif]

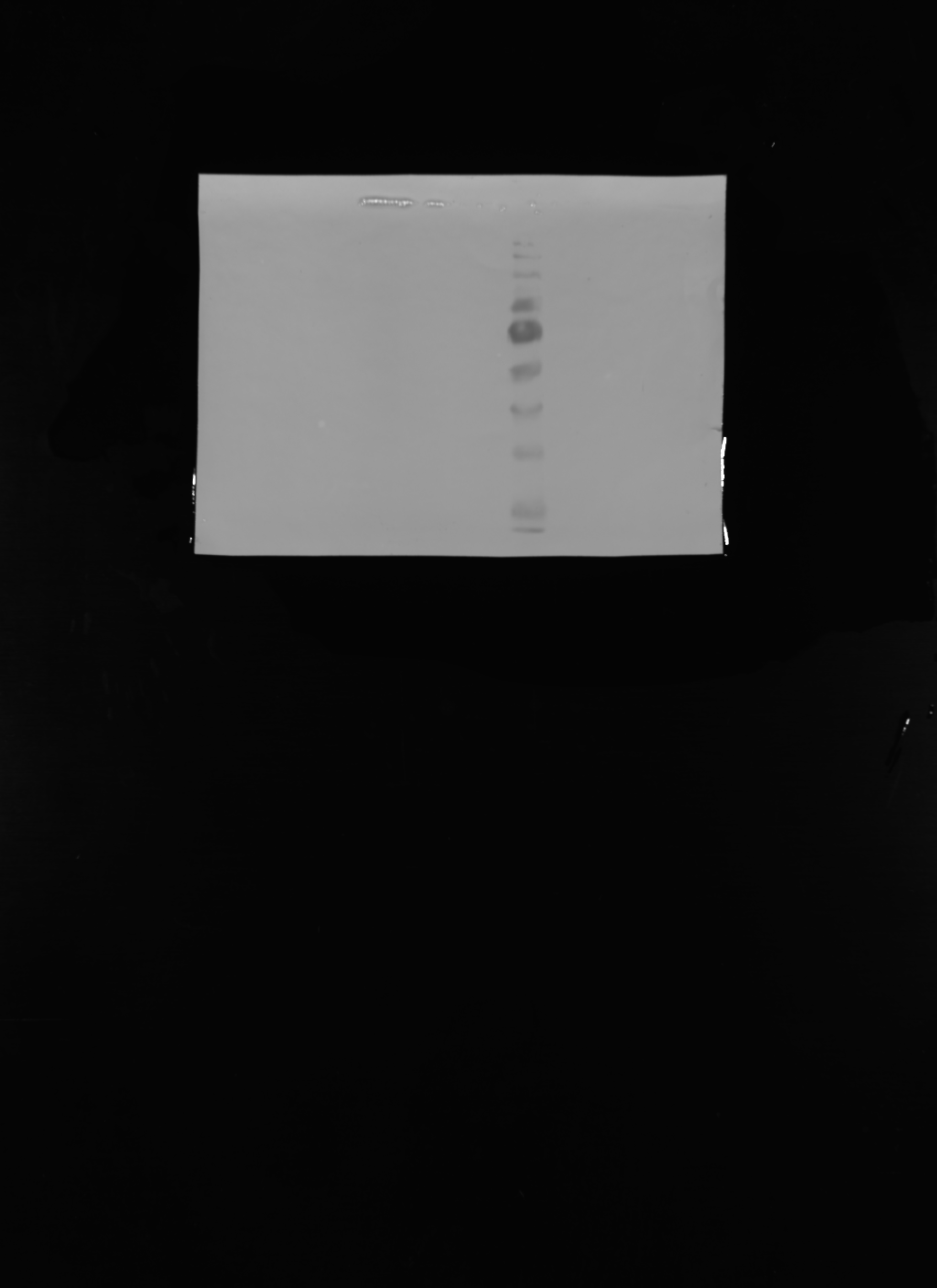

Supplement: Figure 5—source data 1. [file elife-87572-fig5-data1.zip › MCM2/Rep1/G4-mcmcy5-h2a1c5-0.5 2022.11.19_16.58.26_Fl-Red/G4-mcmcy5-h2a1c5-0.5 2022.11.19_16.58.26_Fl-Red-Marker.tif]

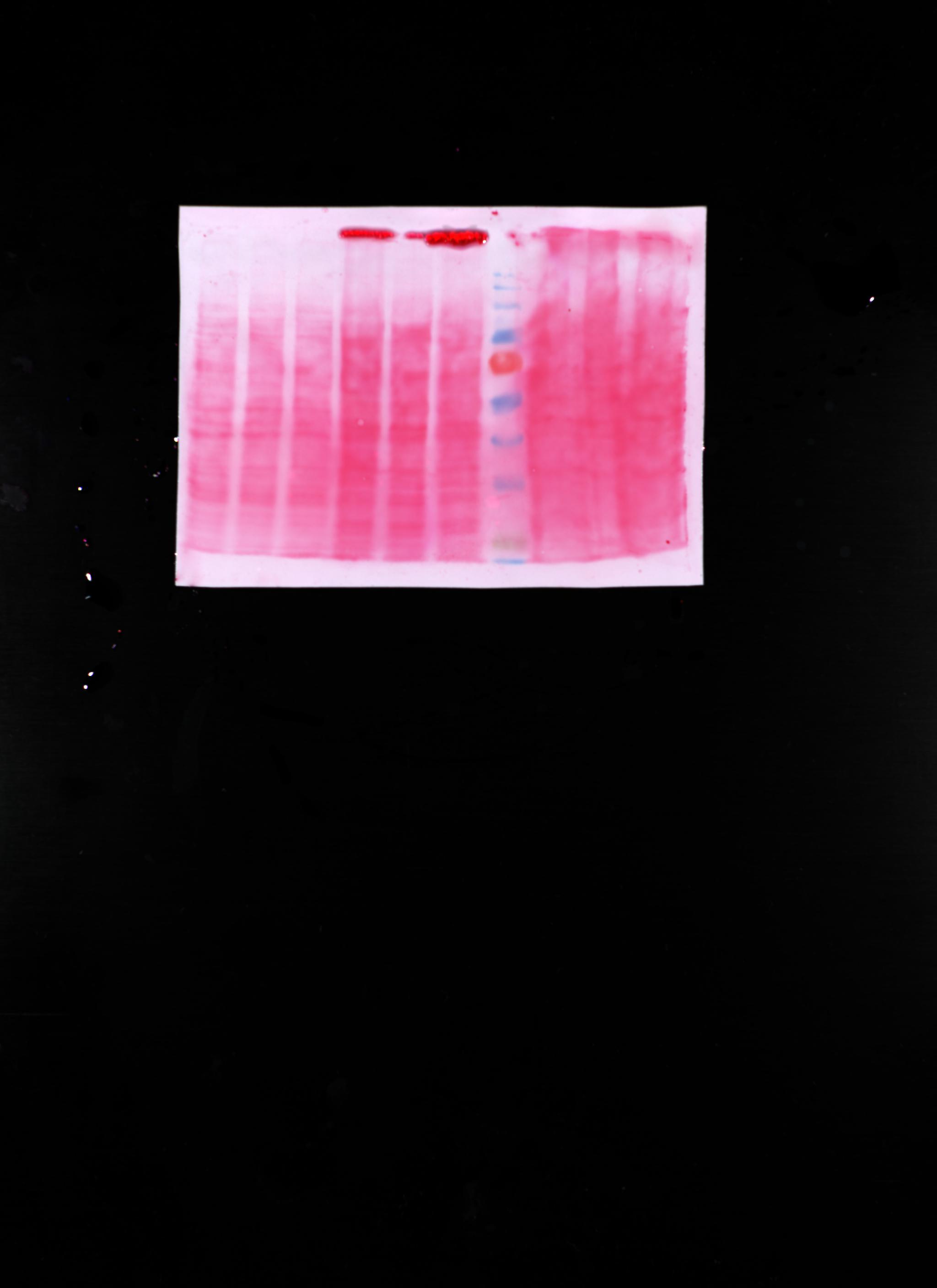

Supplement: Figure 5—source data 1. [file elife-87572-fig5-data1.zip › MCM2/Rep1/P-G4-PolDMCM2-mH2A1 2022.11.18_14.36.44_Co/P-G4-PolDMCM2-mH2A1 2022.11.18_14.36.44_Co.jpg]

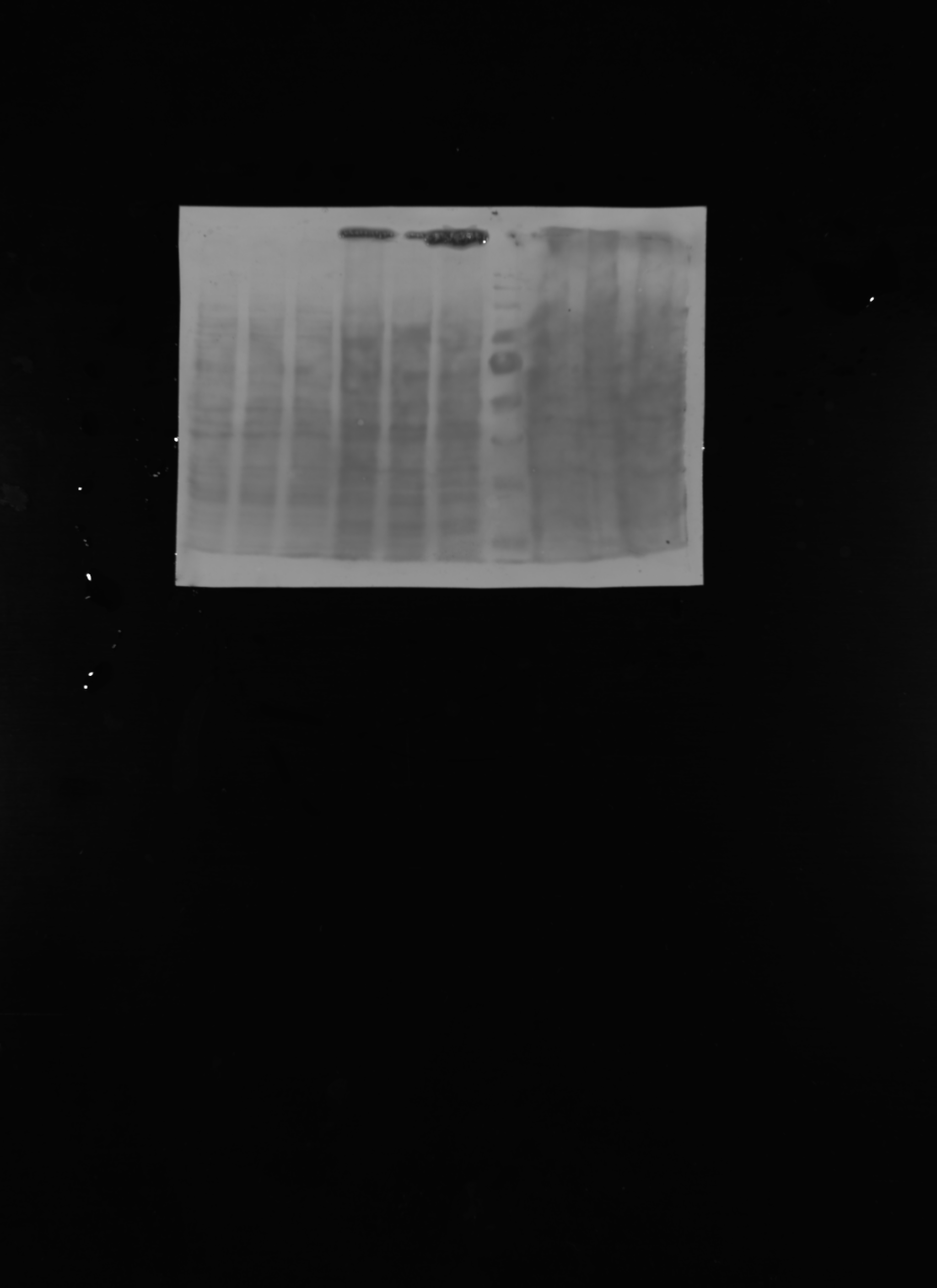

Supplement: Figure 5—source data 1. [file elife-87572-fig5-data1.zip › MCM2/Rep1/P-G4-PolDMCM2-mH2A1 2022.11.18_14.36.44_Co/P-G4-PolDMCM2-mH2A1 2022.11.18_14.36.44_Co.tif]

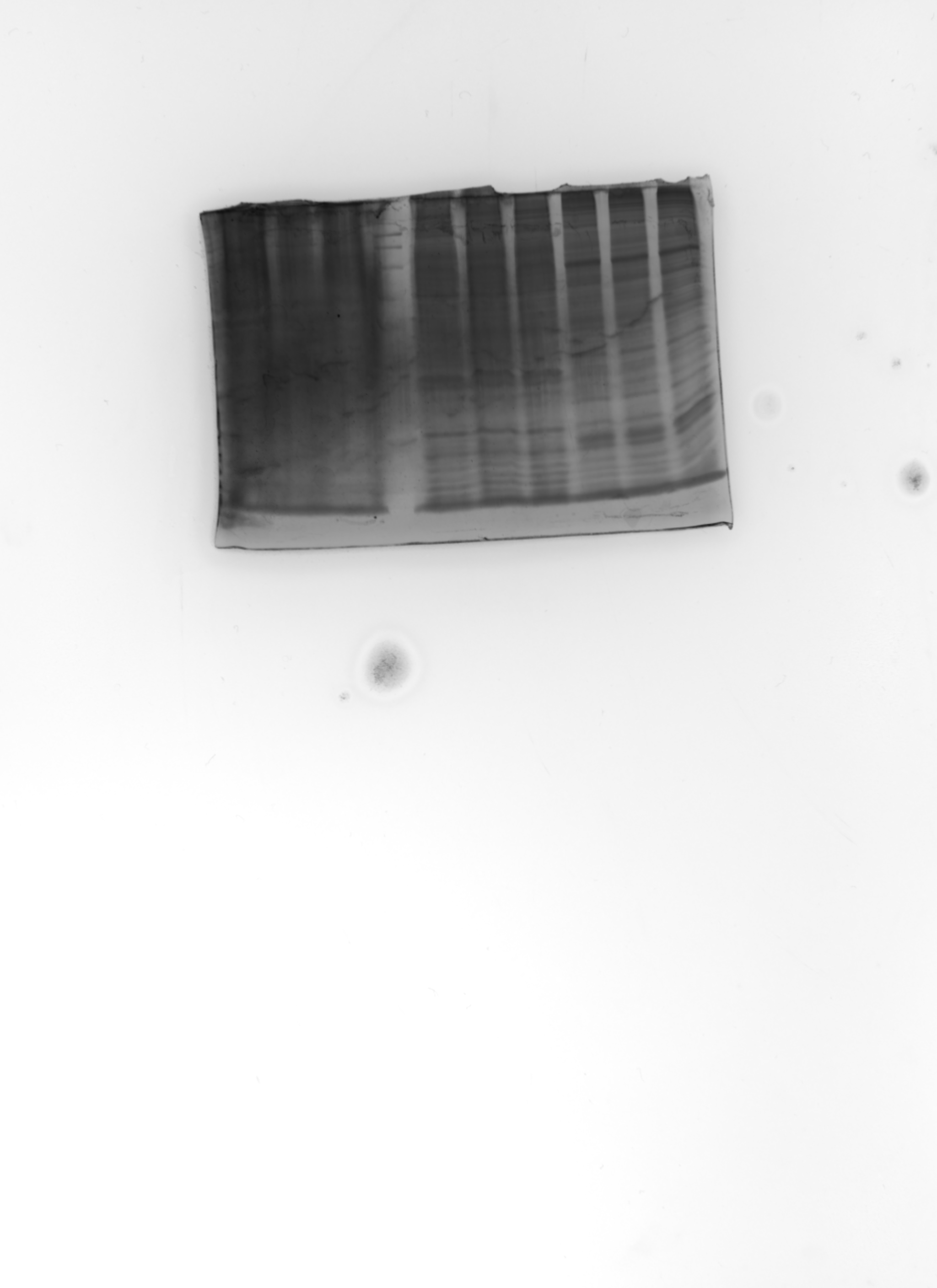

Supplement: Figure 5—source data 2. [file elife-87572-fig5-data2.zip › mH2A1/Rep2/C-G4-mcm2-h2a1 2022.11.21_10.58.43_Co/C-G4-mcm2-h2a1 2022.11.21_10.58.43_Co.tif]

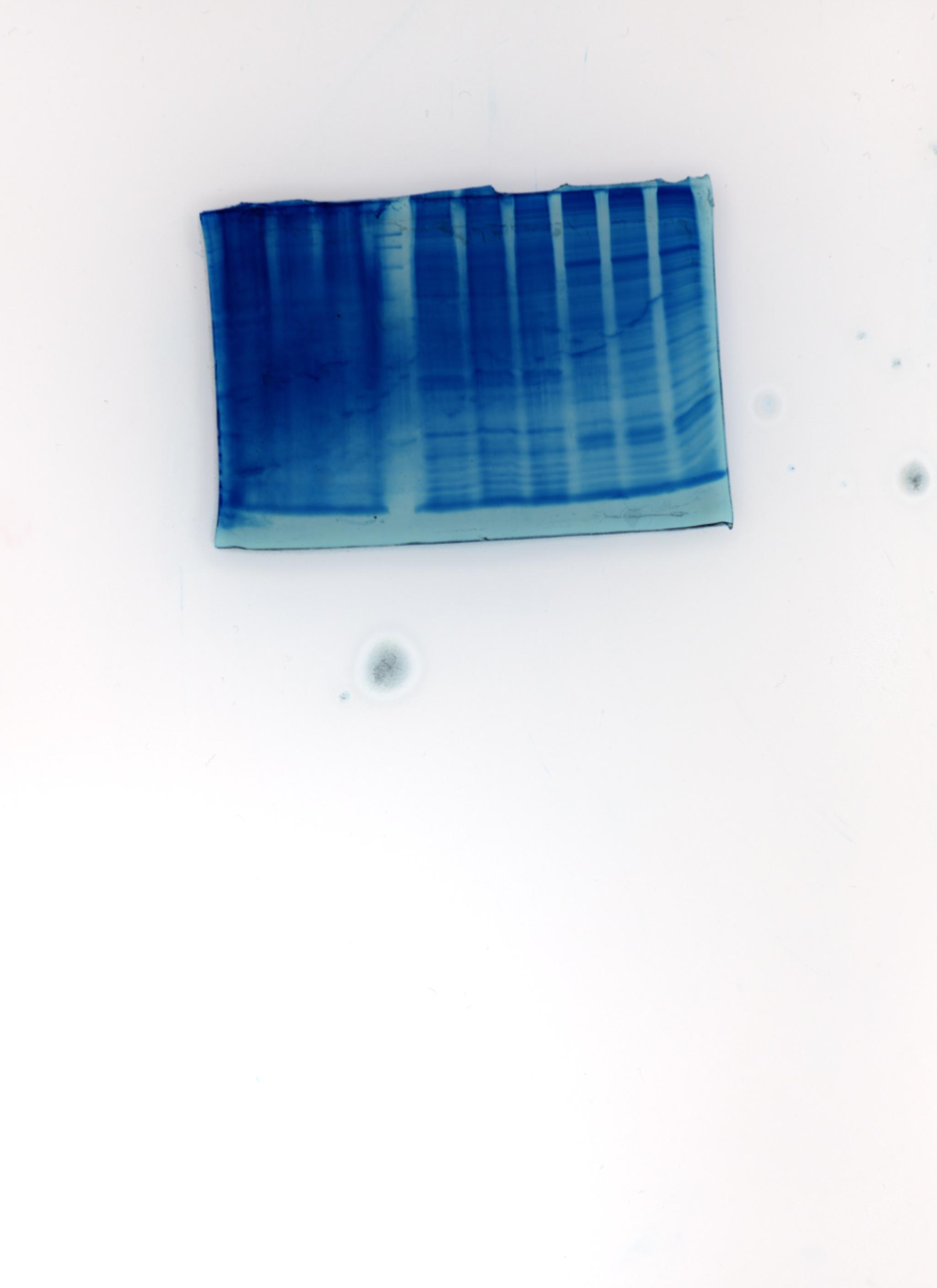

Supplement: Figure 5—source data 2. [file elife-87572-fig5-data2.zip › mH2A1/Rep2/C-G4-mcm2-h2a1 2022.11.21_10.58.43_Co/C-G4-mcm2-h2a1 2022.11.21_10.58.43_Co.jpg]

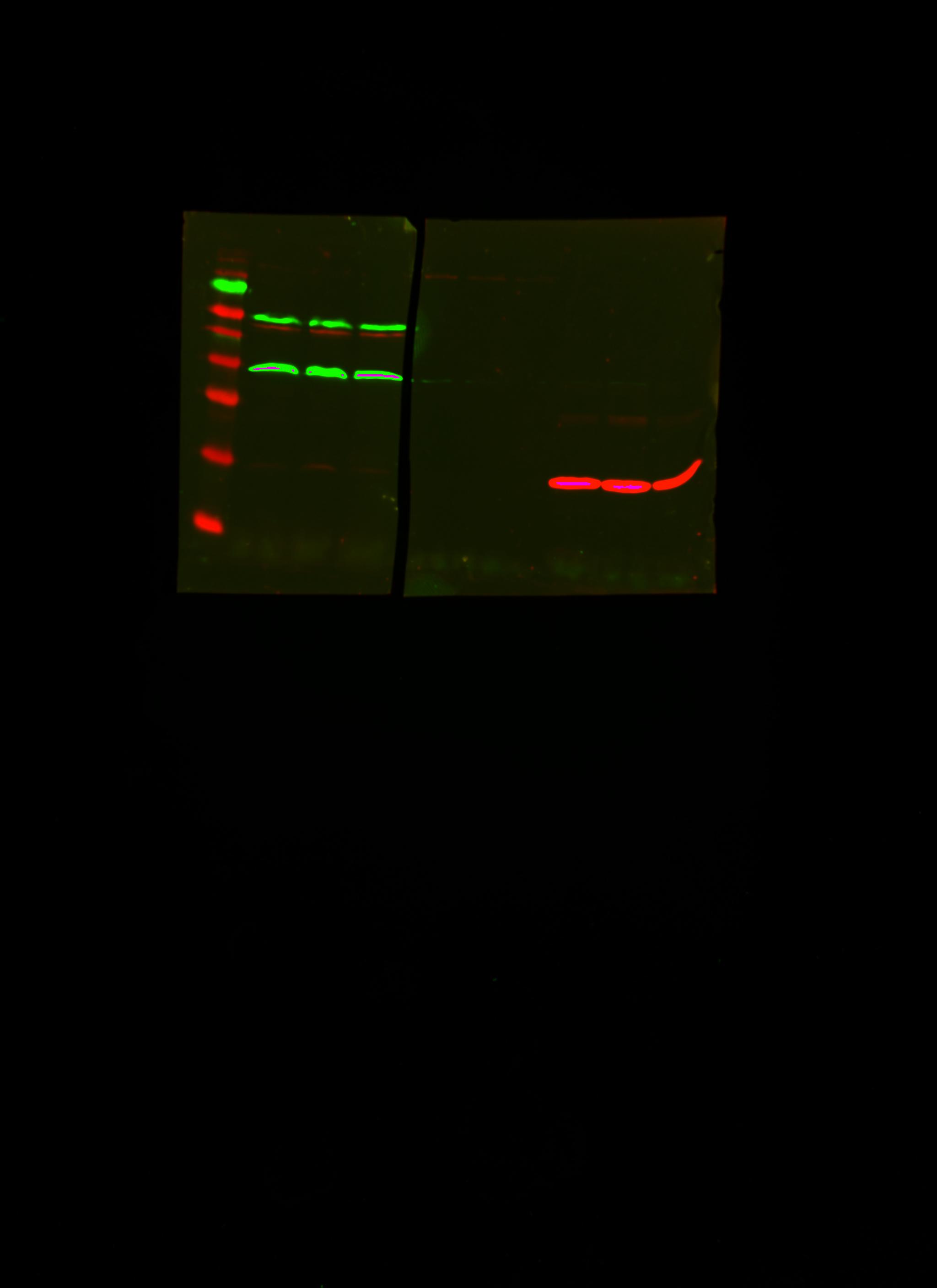

Supplement: Figure 5—source data 2. [file elife-87572-fig5-data2.zip › mH2A1/Rep3/H3Cy5 tubcy3+PCNACy3 2022.07.21_11.24.45_Fl/H3Cy5 tubcy3+PCNACy3 2022.07.21_11.24.45_Fl.jpg]

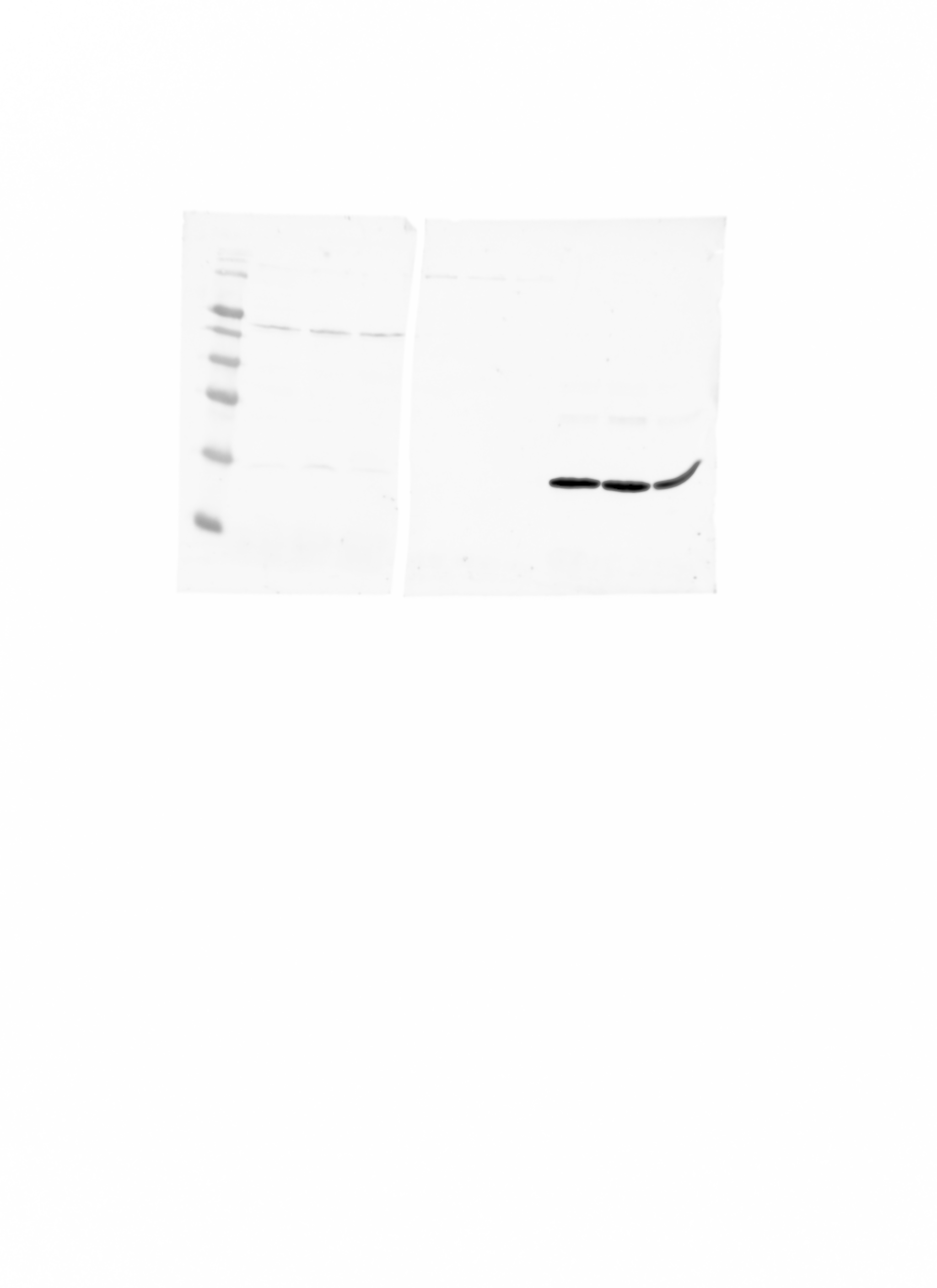

Supplement: Figure 5—source data 2. [file elife-87572-fig5-data2.zip › mH2A1/Rep3/H3Cy5 tubcy3+PCNACy3 2022.07.21_11.24.45_Fl/H3Cy5 tubcy3+PCNACy3 2022.07.21_11.24.45_Fl-Red.tif]

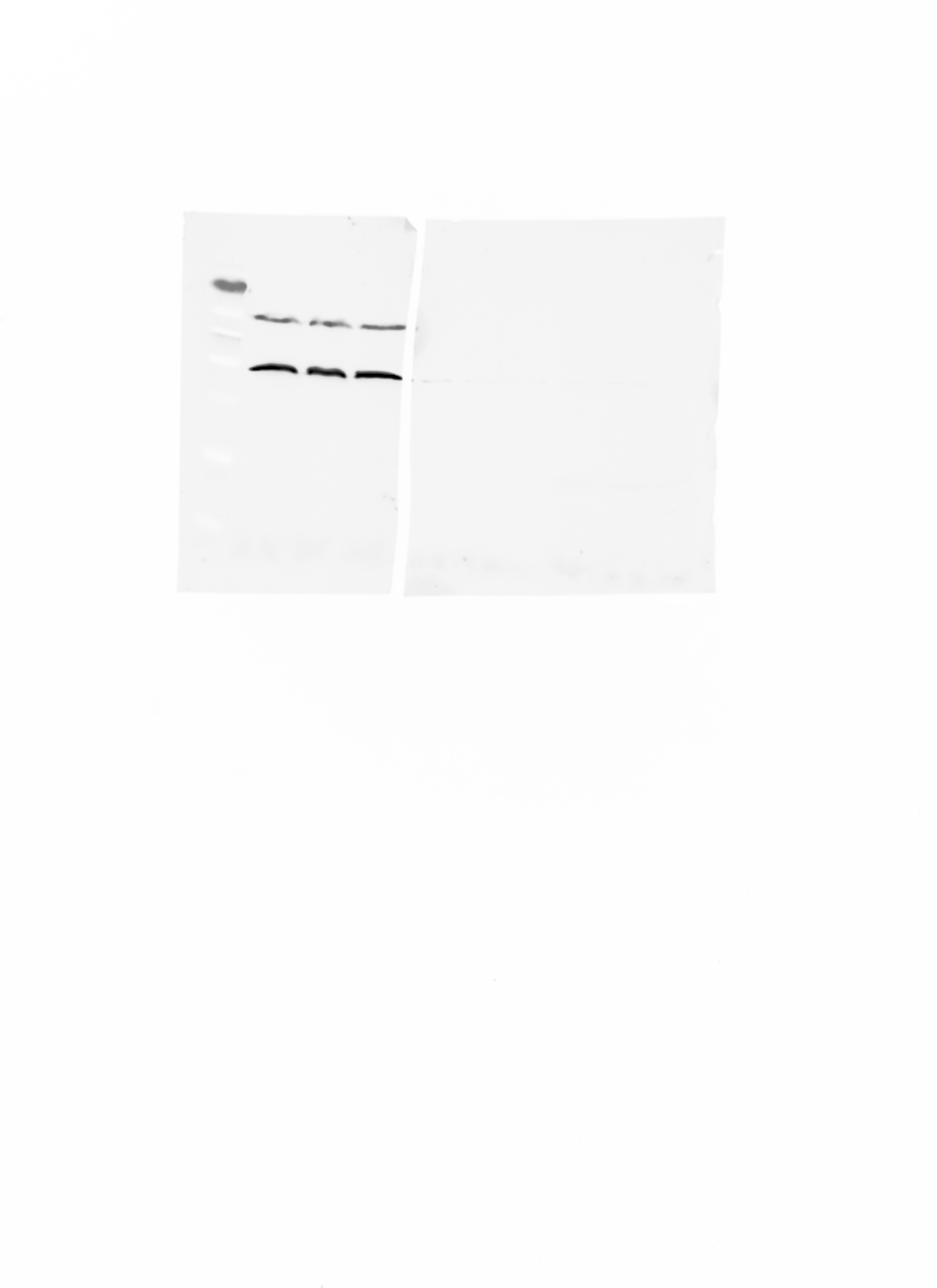

Supplement: Figure 5—source data 2. [file elife-87572-fig5-data2.zip › mH2A1/Rep3/H3Cy5 tubcy3+PCNACy3 2022.07.21_11.24.45_Fl/H3Cy5 tubcy3+PCNACy3 2022.07.21_11.24.45_Fl-Green.tif]

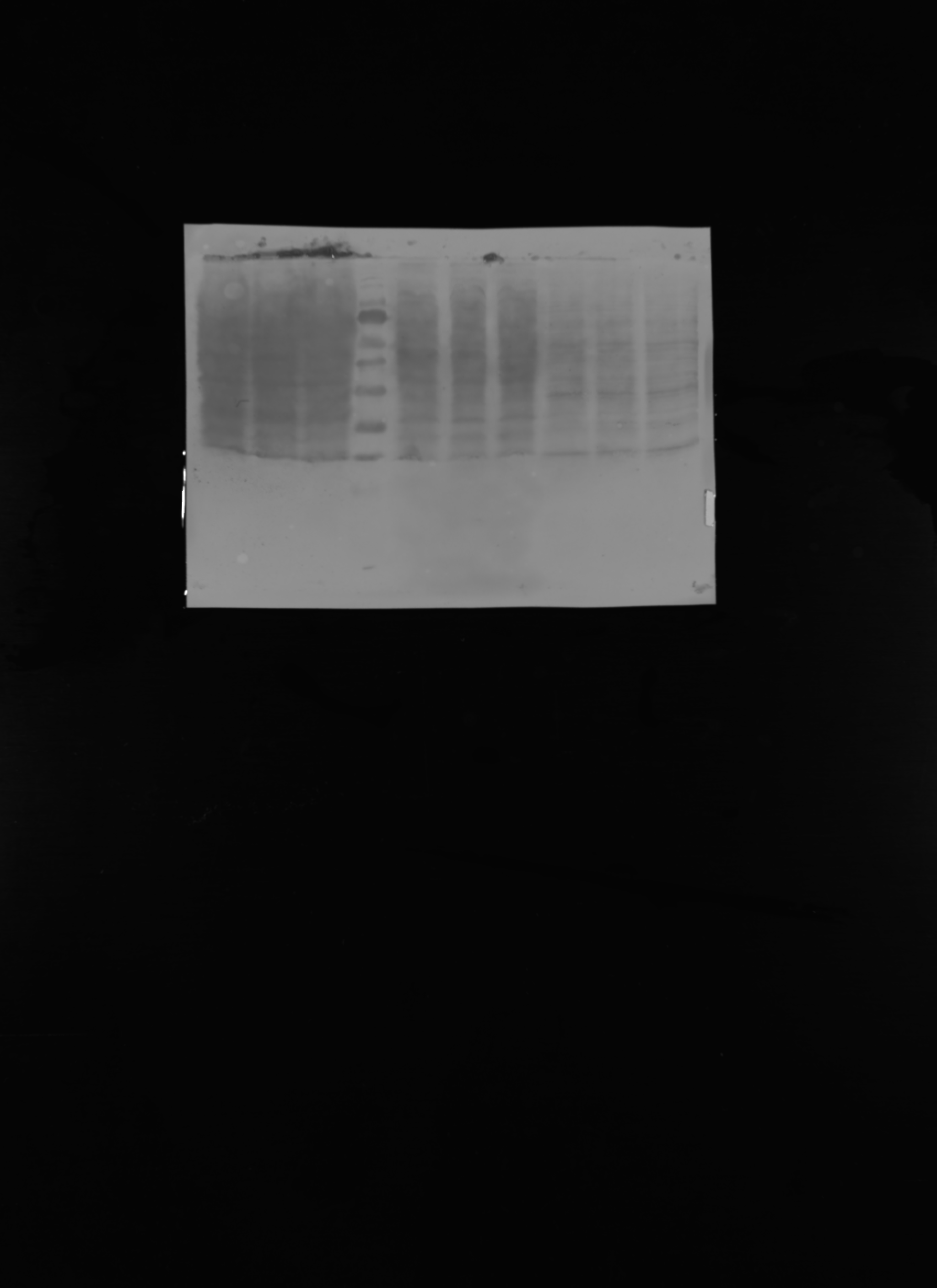

Supplement: Figure 5—source data 2. [file elife-87572-fig5-data2.zip › mH2A1/Rep1/G1-mTubCy3-rabH2A1Cy5-12%Gel-Firstprobe/P-G1-mtub-rbHis-12% 2022.11.10_17.32.55_Co/P-G1-mtub-rbHis-12% 2022.11.10_17.32.55_Co.tif]

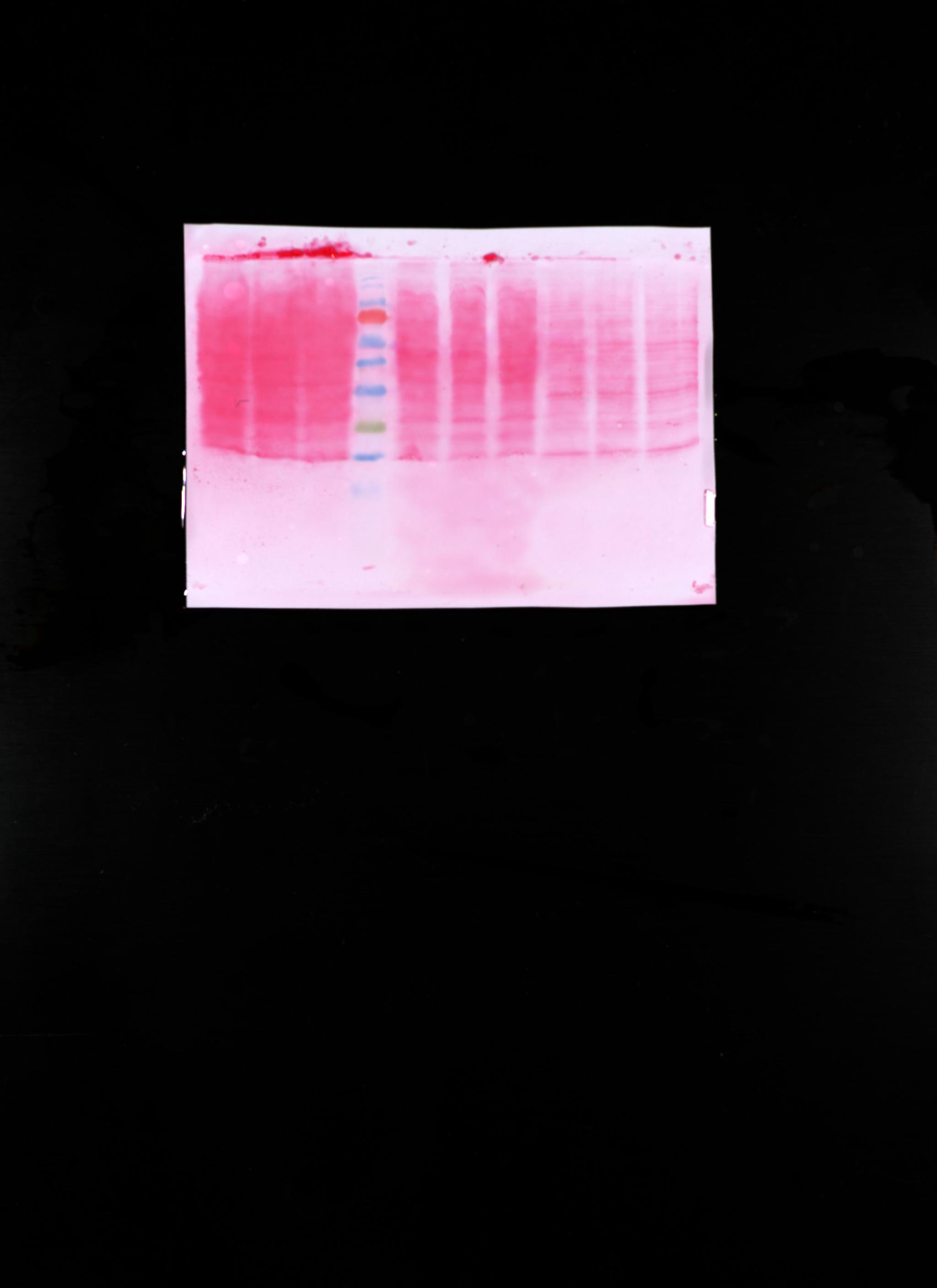

Supplement: Figure 5—source data 2. [file elife-87572-fig5-data2.zip › mH2A1/Rep1/G1-mTubCy3-rabH2A1Cy5-12%Gel-Firstprobe/P-G1-mtub-rbHis-12% 2022.11.10_17.32.55_Co/P-G1-mtub-rbHis-12% 2022.11.10_17.32.55_Co.jpg]

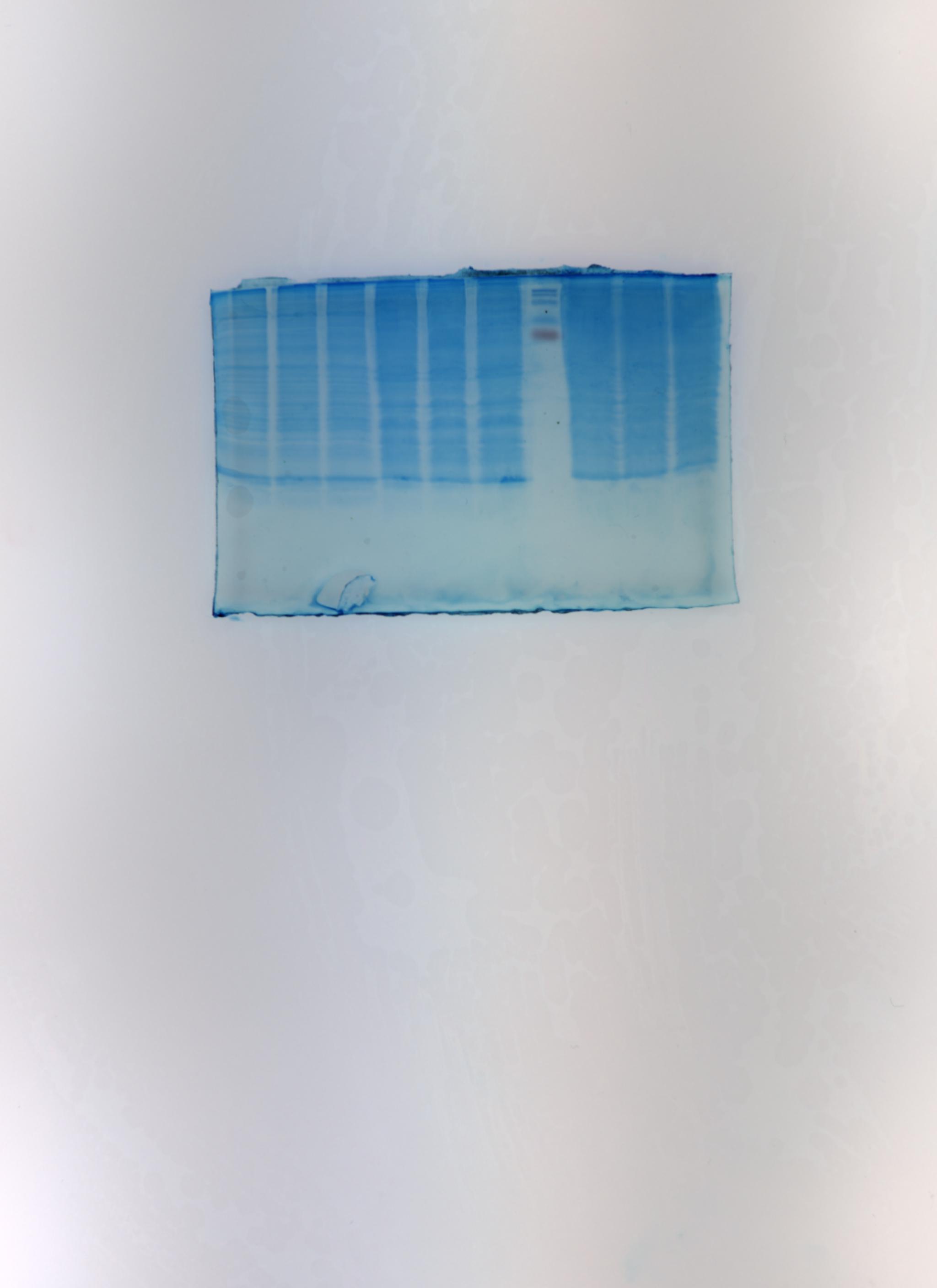

Supplement: Figure 5—source data 2. [file elife-87572-fig5-data2.zip › mH2A1/Rep1/G1-mTubCy3-rabH2A1Cy5-12%Gel-Firstprobe/C-G1-tub-h2a1 2022.11.11_16.40.07_Co/C-G1-tub-h2a1 2022.11.11_16.40.07_Co.jpg]

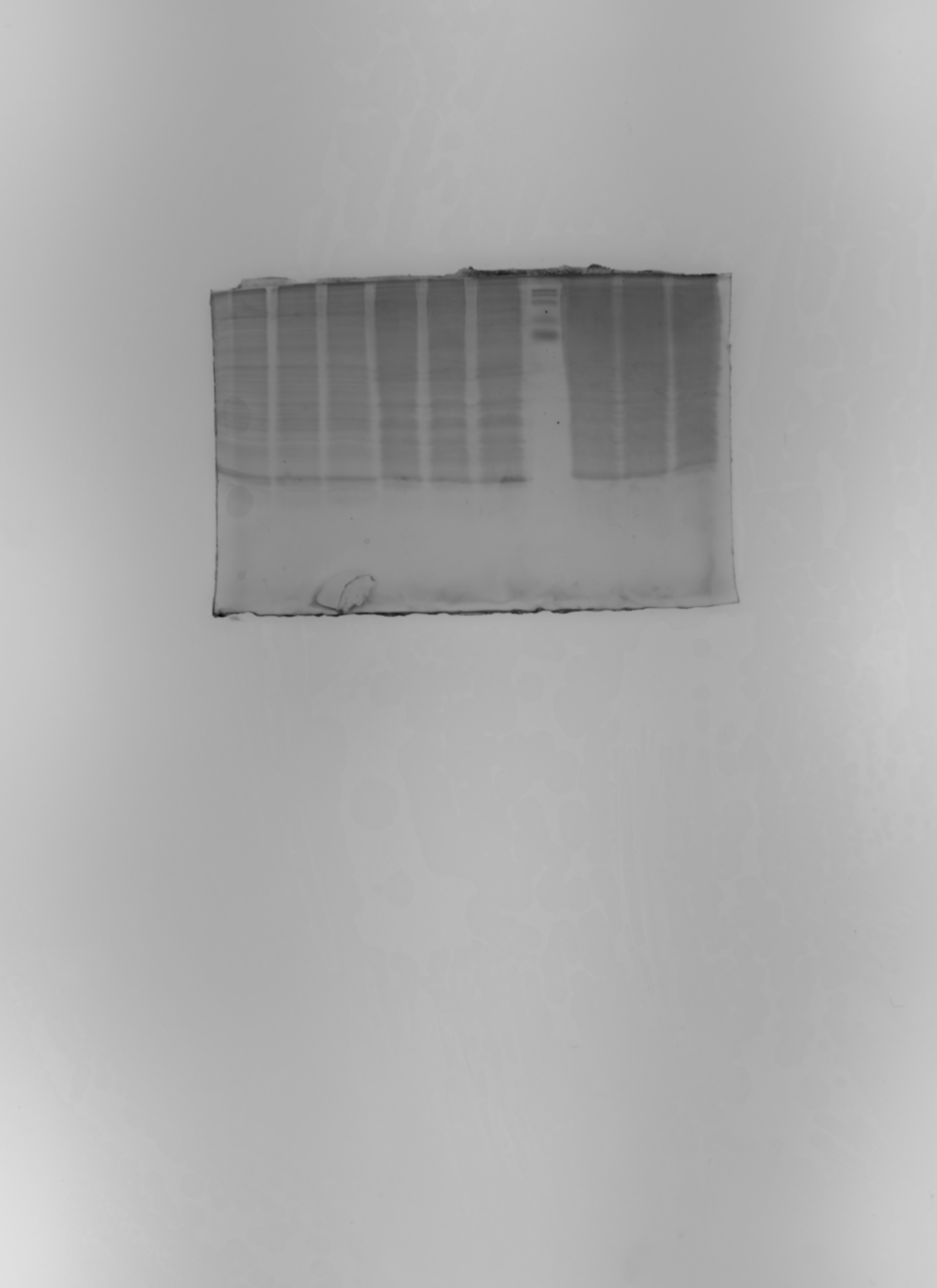

Supplement: Figure 5—source data 2. [file elife-87572-fig5-data2.zip › mH2A1/Rep1/G1-mTubCy3-rabH2A1Cy5-12%Gel-Firstprobe/C-G1-tub-h2a1 2022.11.11_16.40.07_Co/C-G1-tub-h2a1 2022.11.11_16.40.07_Co.tif]

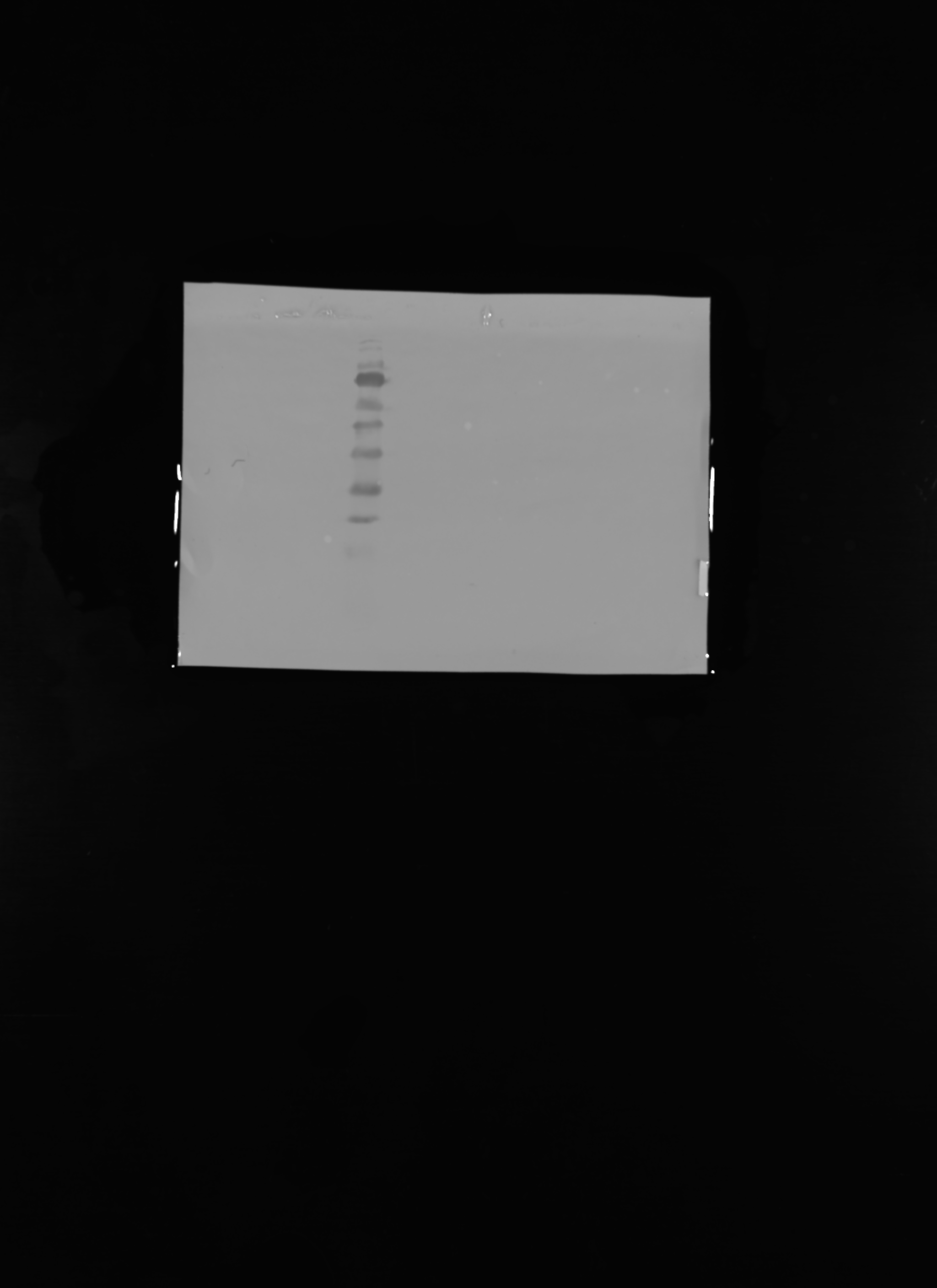

Supplement: Figure 5—source data 2. [file elife-87572-fig5-data2.zip › mH2A1/Rep1/G1-mTubCy3-rabH2A1Cy5-12%Gel-Firstprobe/G1-rH2a1Cy5-1s-12% 2022.11.11_12.15.37_Fl-Red/G1-rH2a1Cy5-1s-12% 2022.11.11_12.15.37_Fl-Red-Marker.tif]

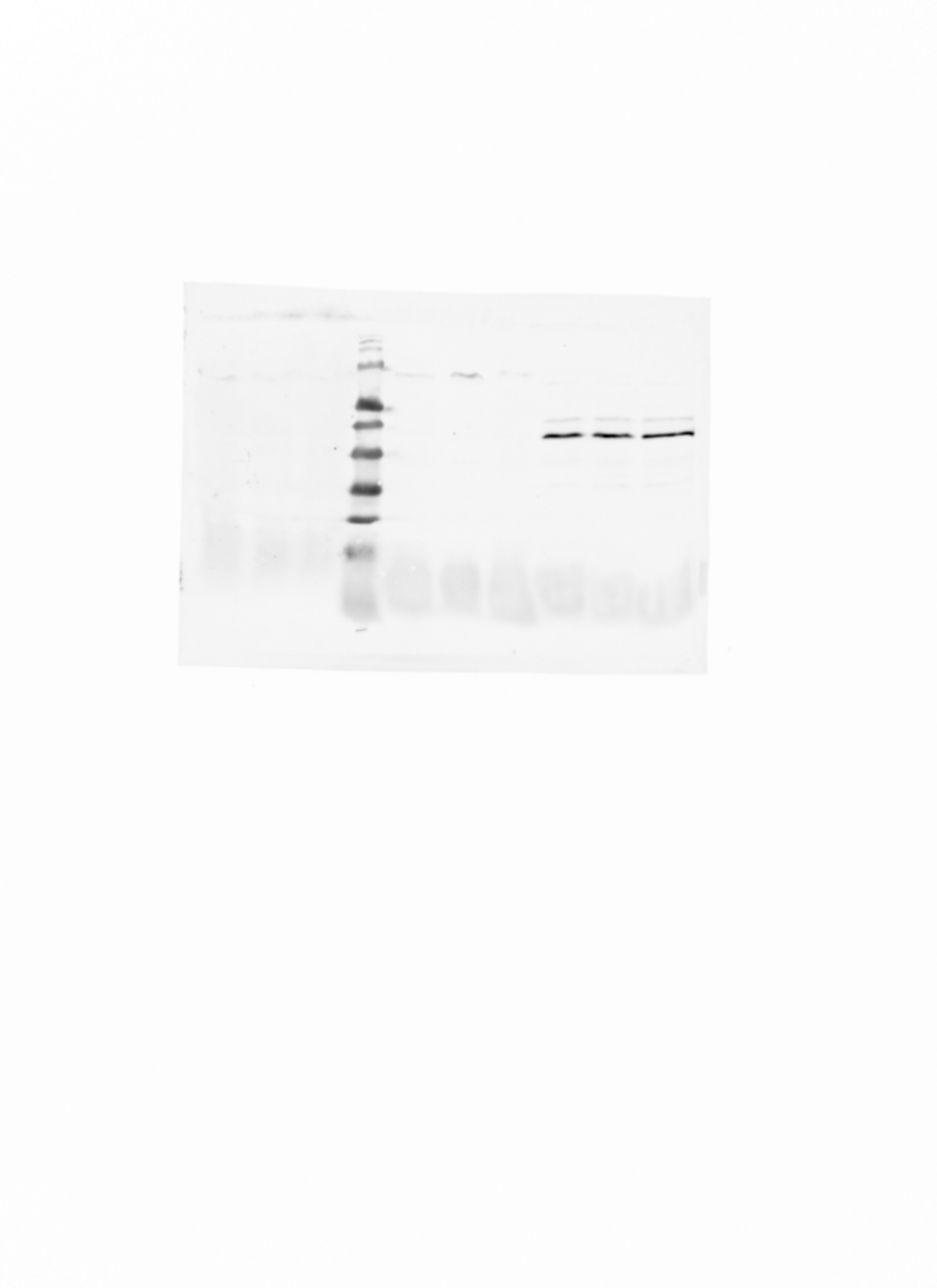

Supplement: Figure 5—source data 2. [file elife-87572-fig5-data2.zip › mH2A1/Rep1/G1-mTubCy3-rabH2A1Cy5-12%Gel-Firstprobe/G1-rH2a1Cy5-1s-12% 2022.11.11_12.15.37_Fl-Red/G1-rH2a1Cy5-1s-12% 2022.11.11_12.15.37_Fl-Red.tif]

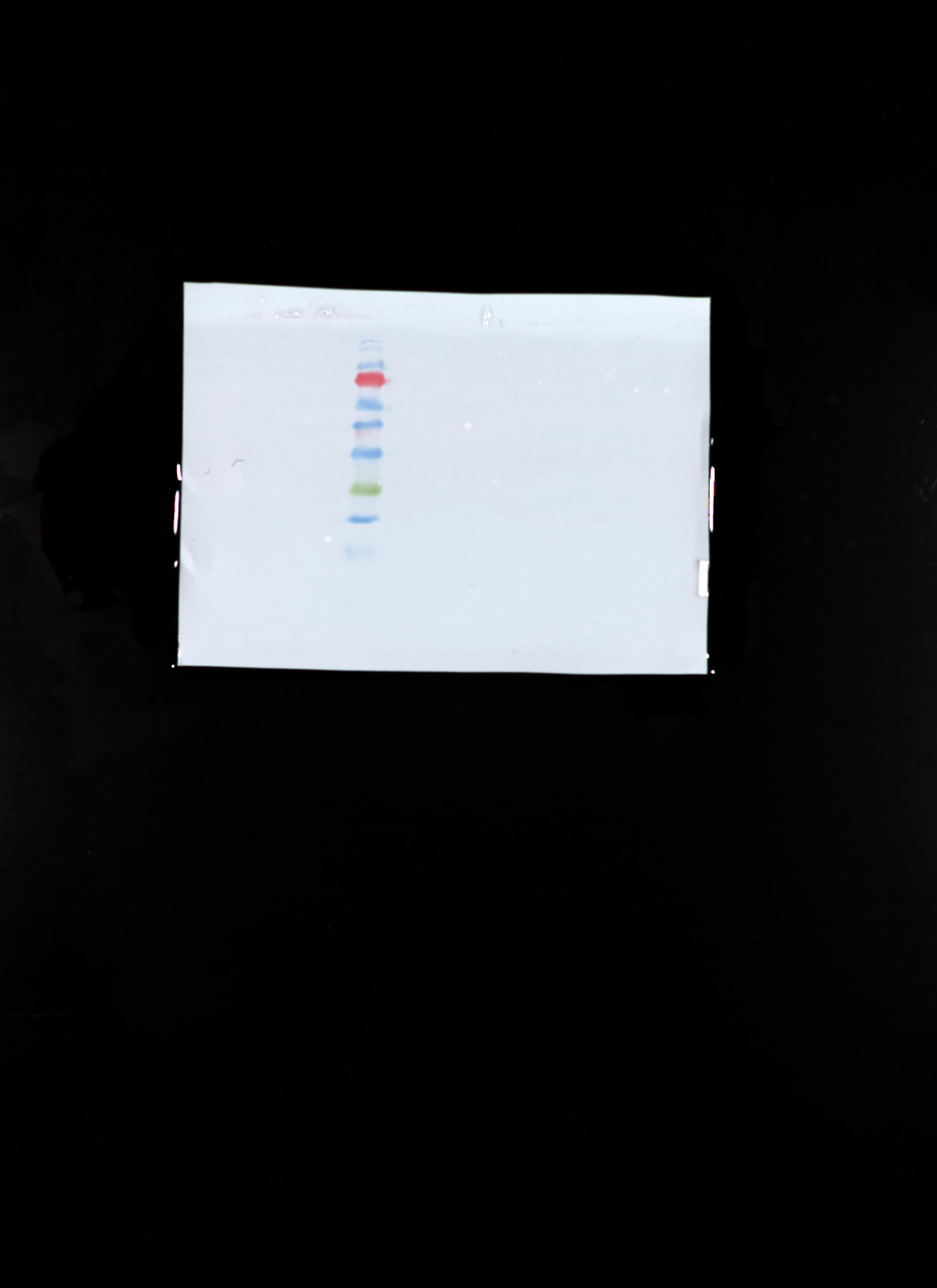

Supplement: Figure 5—source data 2. [file elife-87572-fig5-data2.zip › mH2A1/Rep1/G1-mTubCy3-rabH2A1Cy5-12%Gel-Firstprobe/G1-rH2a1Cy5-1s-12% 2022.11.11_12.15.37_Fl-Red/G1-rH2a1Cy5-1s-12% 2022.11.11_12.15.37_Fl-Red-Marker.jpg]

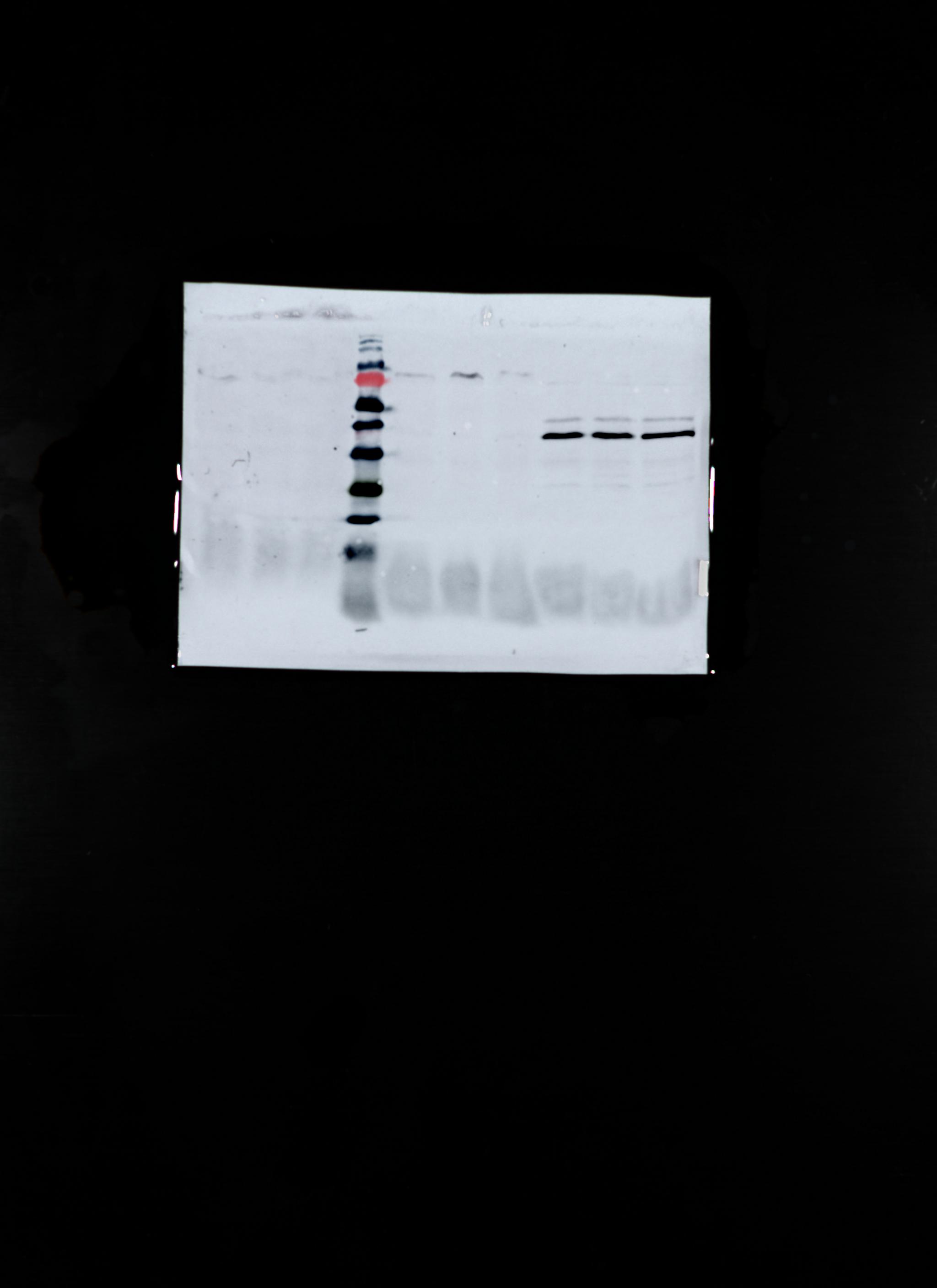

Supplement: Figure 5—source data 2. [file elife-87572-fig5-data2.zip › mH2A1/Rep1/G1-mTubCy3-rabH2A1Cy5-12%Gel-Firstprobe/G1-rH2a1Cy5-1s-12% 2022.11.11_12.15.37_Fl-Red/G1-rH2a1Cy5-1s-12% 2022.11.11_12.15.37_Fl-Red+Marker.jpg]

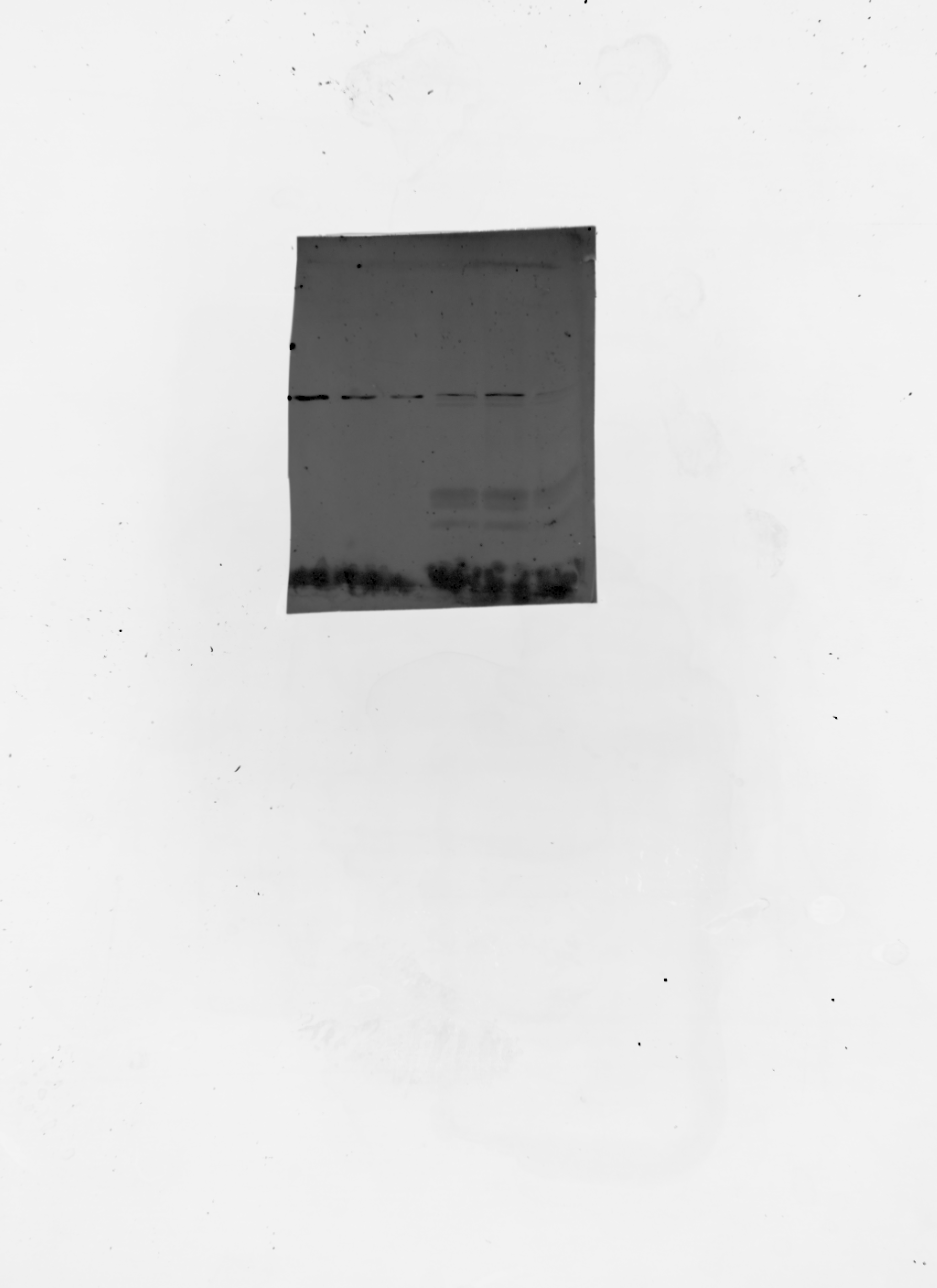

Supplement: Figure 5—source data 3. [file elife-87572-fig5-data3.zip › PCNA/Rep2/wb pcnatub fraction 2022.07.19_12.42.02_Fl-Green/wb pcnatub fraction 2022.07.19_12.42.02_Fl-Green.tif]

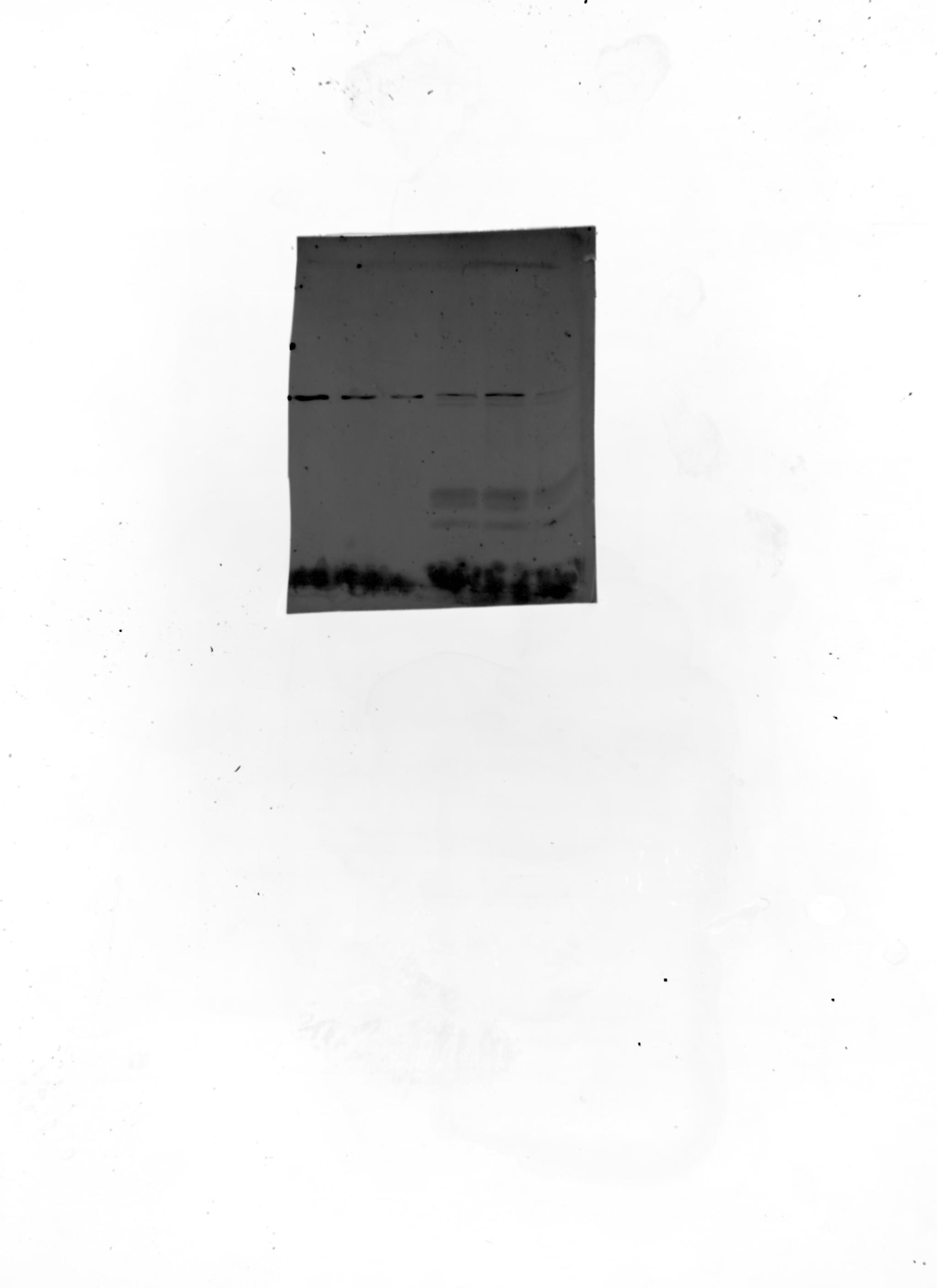

Supplement: Figure 5—source data 3. [file elife-87572-fig5-data3.zip › PCNA/Rep2/wb pcnatub fraction 2022.07.19_12.42.02_Fl-Green/wb pcnatub fraction 2022.07.19_12.42.02_Fl-Green.jpg]

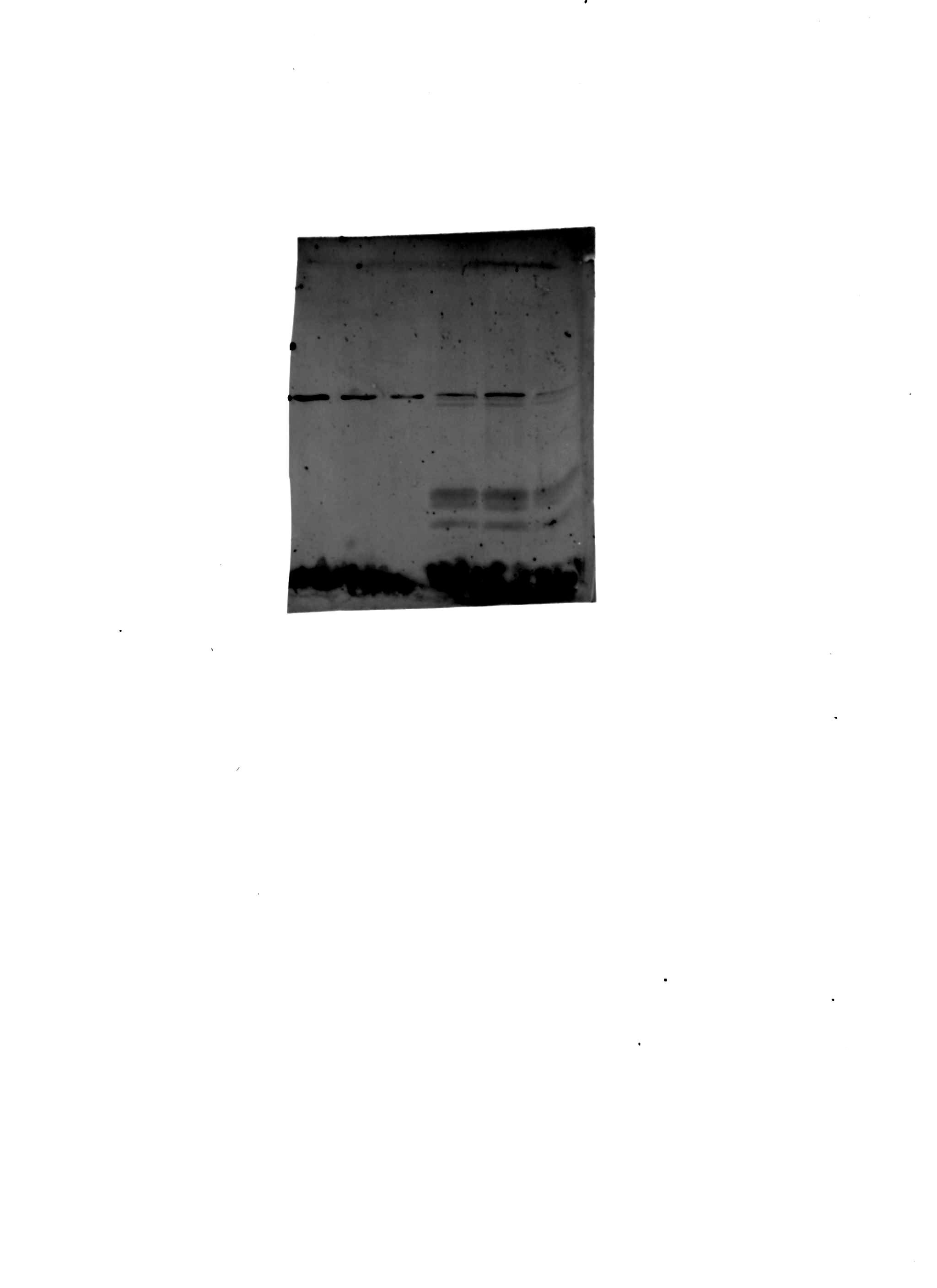

Supplement: Figure 5—source data 3. [file elife-87572-fig5-data3.zip › PCNA/Rep2/wb pcnatub fraction 2022.07.19_12.42.02_Fl-Green/ewb pcnatub fraction 2022.07.19_12.42.02_Fl-Green.tif]

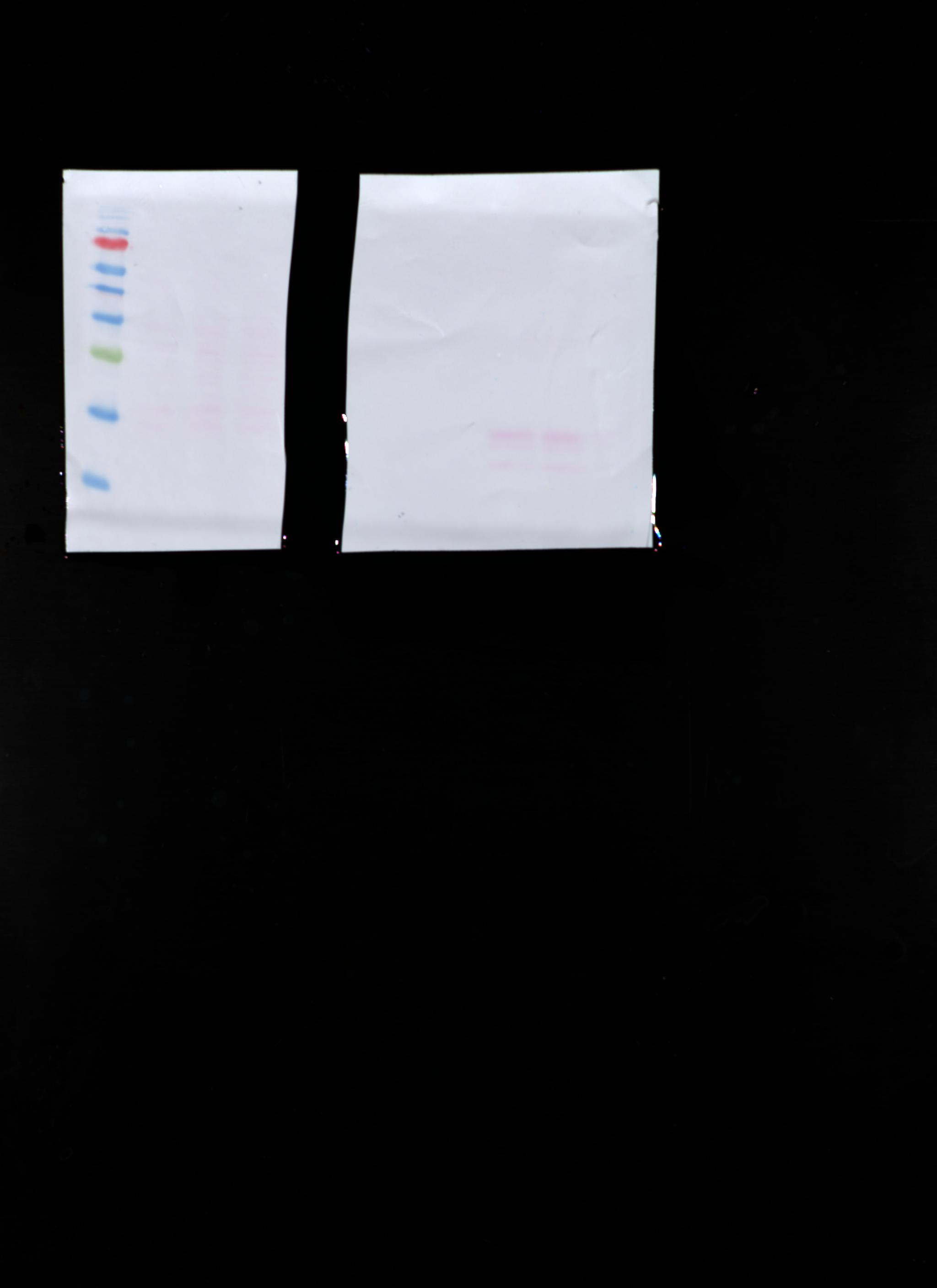

Supplement: Figure 5—source data 3. [file elife-87572-fig5-data3.zip › PCNA/Rep2/wb pcnatub fraction2 2022.07.19_12.18.47_Fl-Green/wb pcnatub fraction2 2022.07.19_12.18.47_Fl-Green-Marker.jpg]

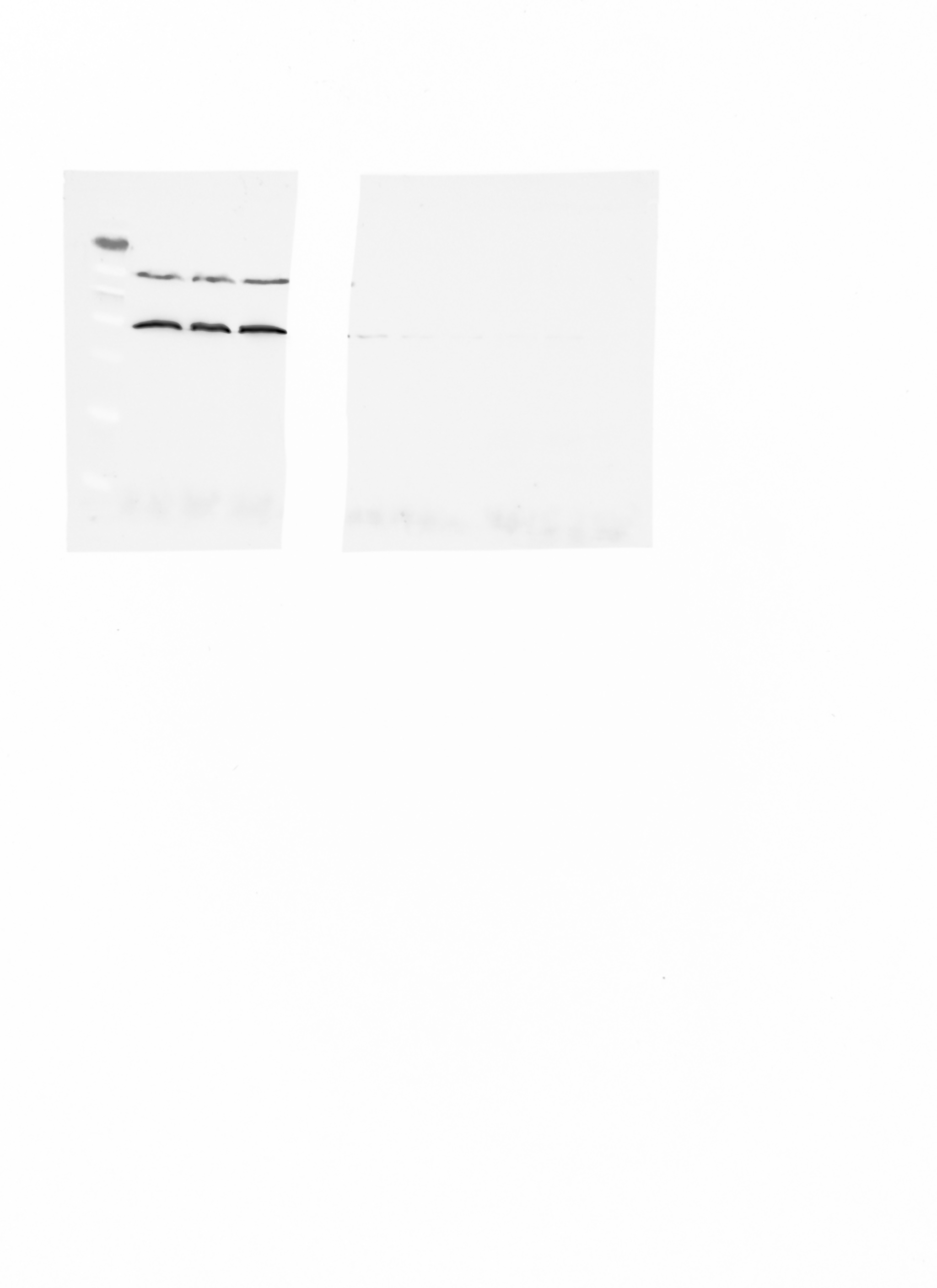

Supplement: Figure 5—source data 3. [file elife-87572-fig5-data3.zip › PCNA/Rep2/wb pcnatub fraction2 2022.07.19_12.18.47_Fl-Green/wb pcnatub fraction2 2022.07.19_12.18.47_Fl-Green.tif]

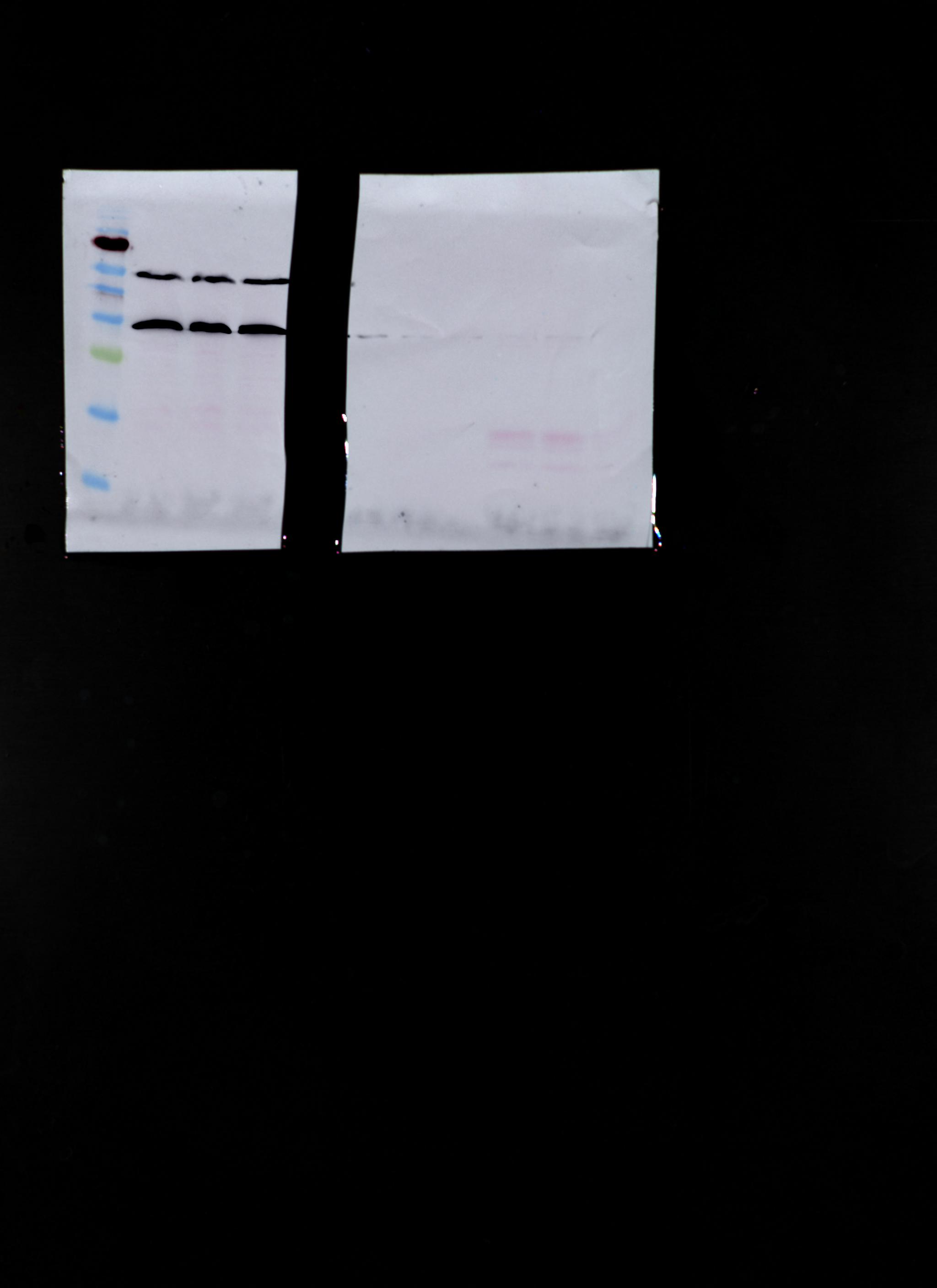

Supplement: Figure 5—source data 3. [file elife-87572-fig5-data3.zip › PCNA/Rep2/wb pcnatub fraction2 2022.07.19_12.18.47_Fl-Green/wb pcnatub fraction2 2022.07.19_12.18.47_Fl-Green+Marker.jpg]

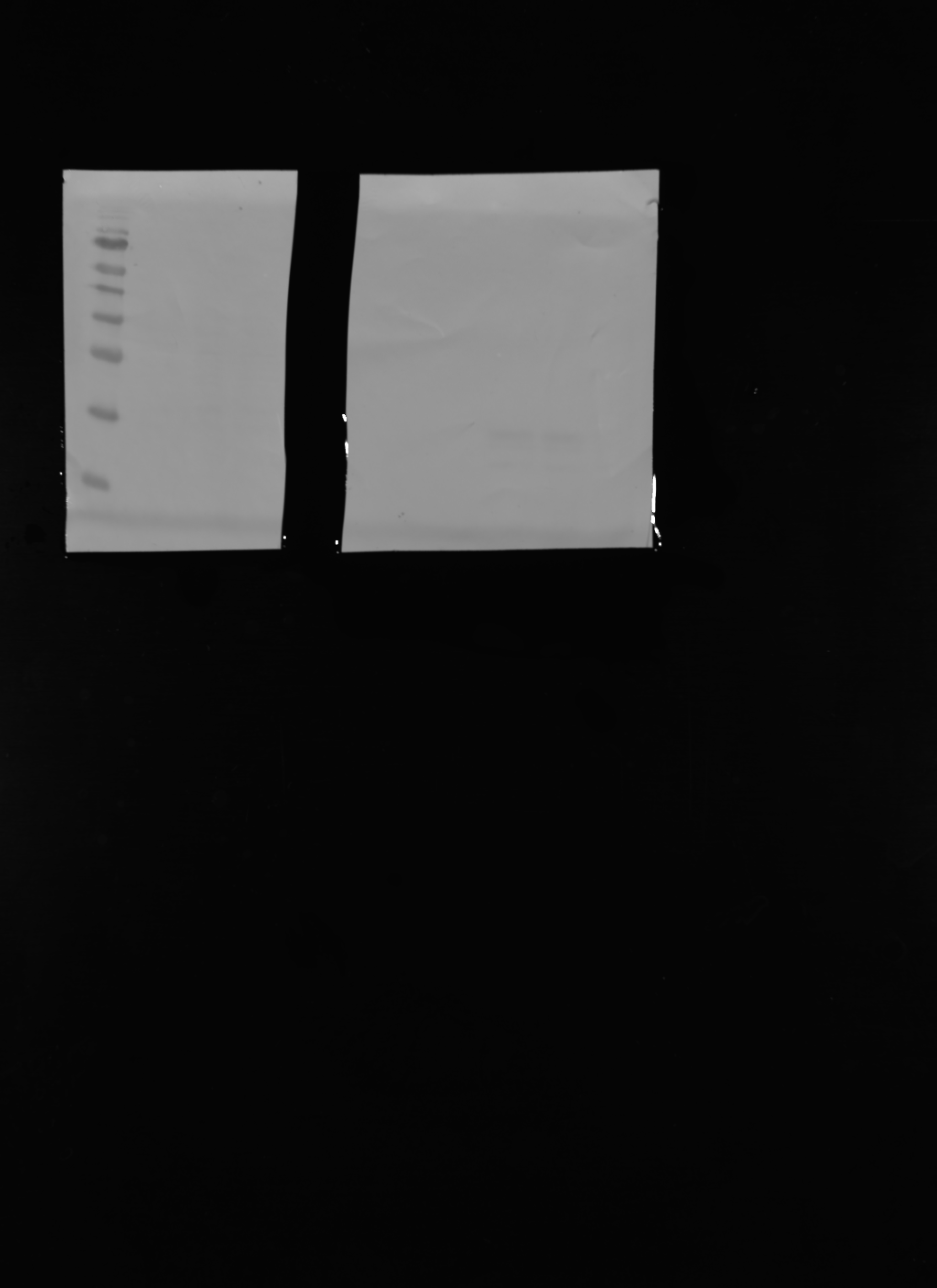

Supplement: Figure 5—source data 3. [file elife-87572-fig5-data3.zip › PCNA/Rep2/wb pcnatub fraction2 2022.07.19_12.18.47_Fl-Green/wb pcnatub fraction2 2022.07.19_12.18.47_Fl-Green-Marker.tif]

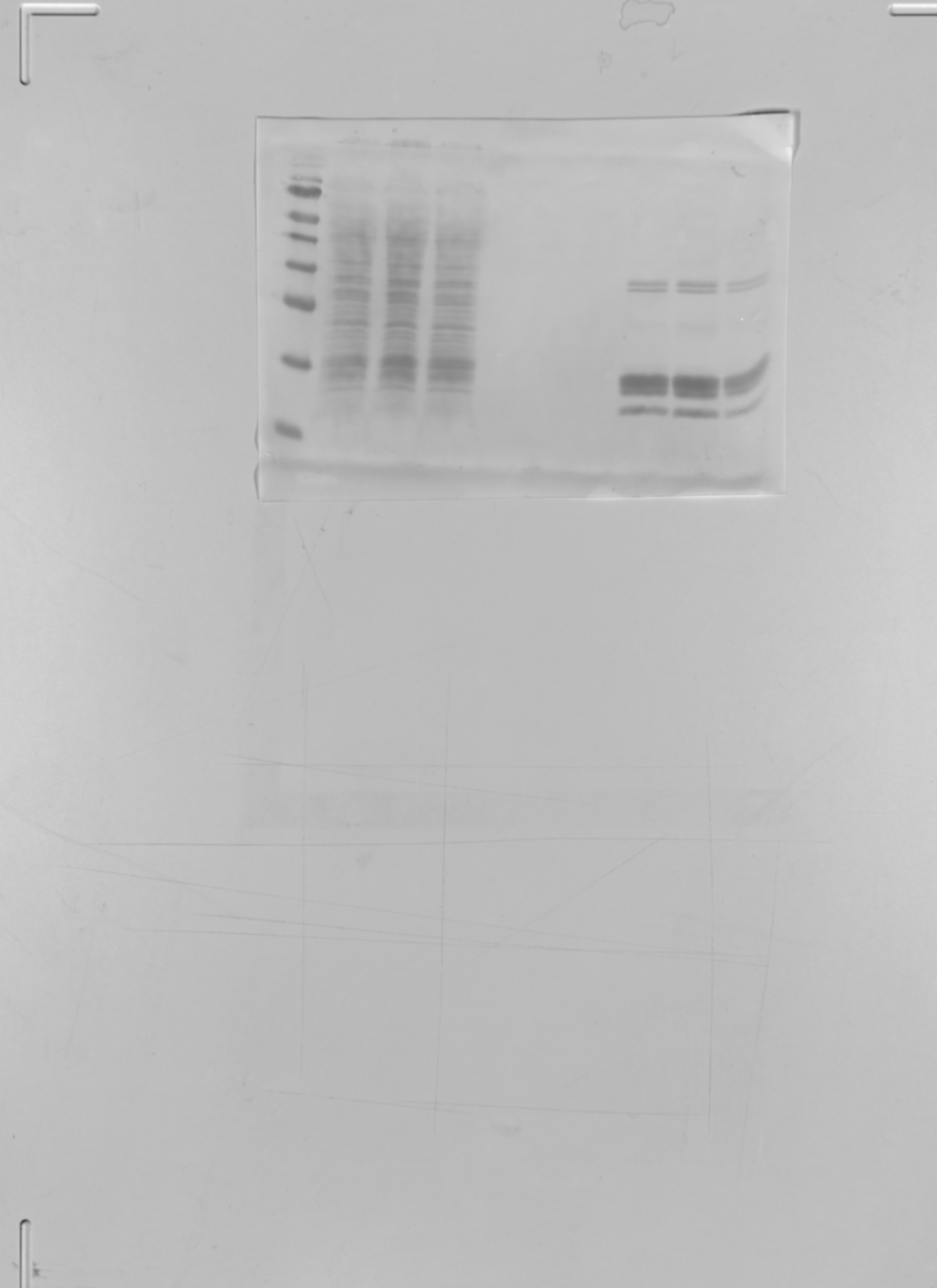

Supplement: Figure 5—source data 3. [file elife-87572-fig5-data3.zip › PCNA/Rep2/AK E1 E2 E3 Ctr HU Aph 2022.07.18_14.54.42_Co/AK E1 E2 E3 Ctr HU Aph 2022.07.18_14.54.42_Co.tif]

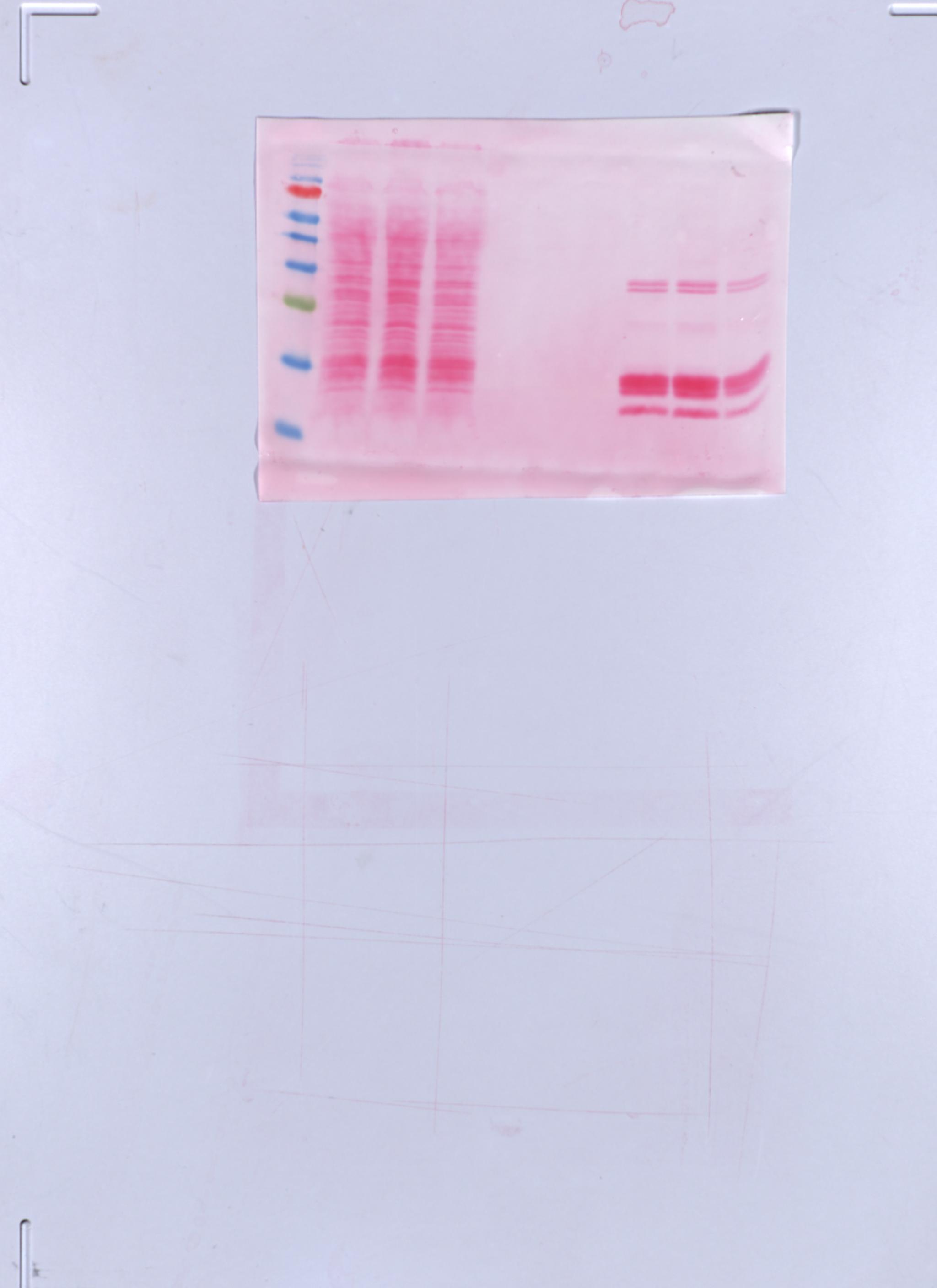

Supplement: Figure 5—source data 3. [file elife-87572-fig5-data3.zip › PCNA/Rep2/AK E1 E2 E3 Ctr HU Aph 2022.07.18_14.54.42_Co/AK E1 E2 E3 Ctr HU Aph 2022.07.18_14.54.42_Co.jpg]

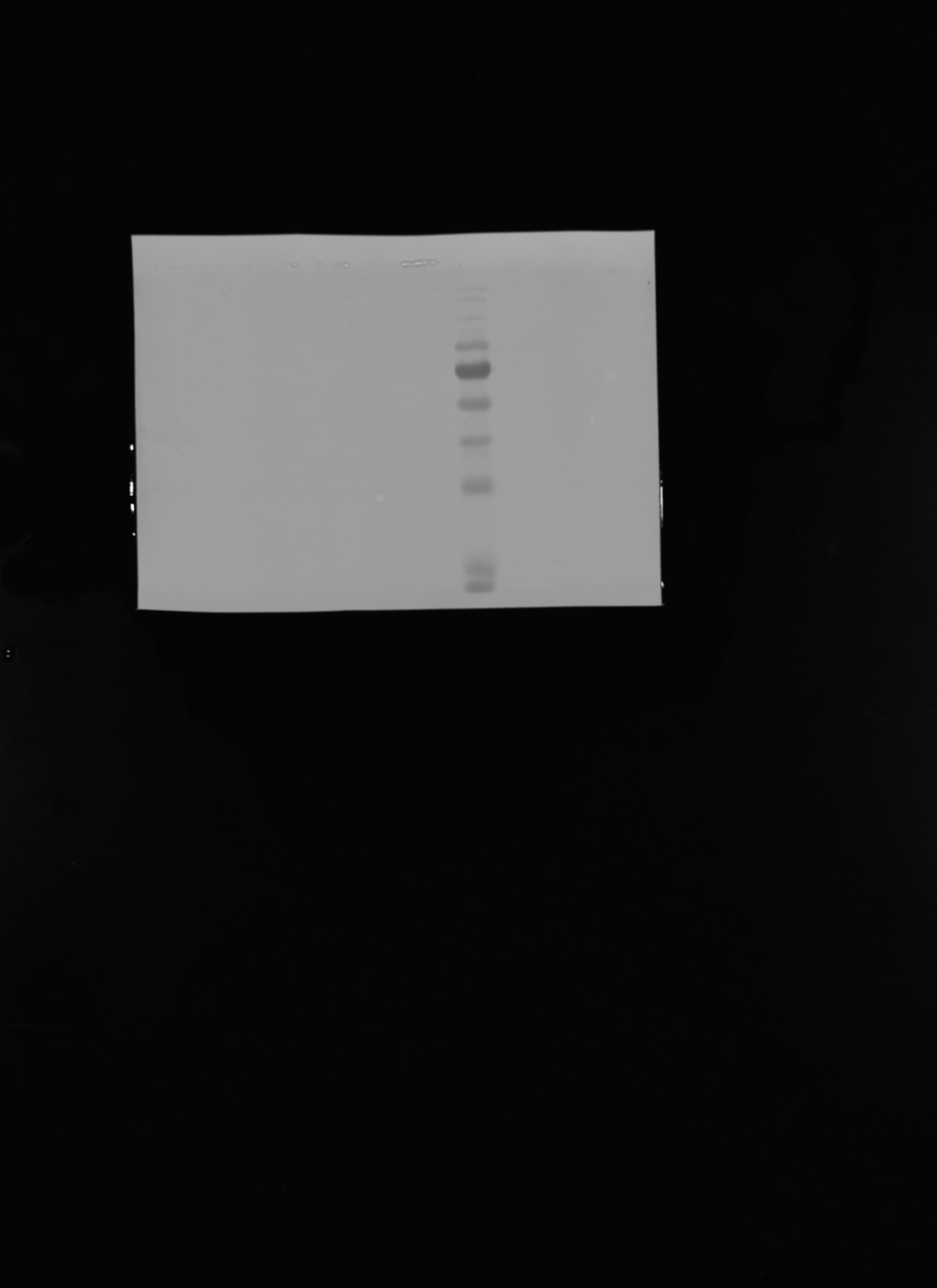

Supplement: Figure 5—source data 3. [file elife-87572-fig5-data3.zip › PCNA/Rep3/Z1-PCNA-Cy3 2022.11.30_15.25.02_Fl-Green/Z1-PCNA-Cy3 2022.11.30_15.25.02_Fl-Green-Marker.tif]

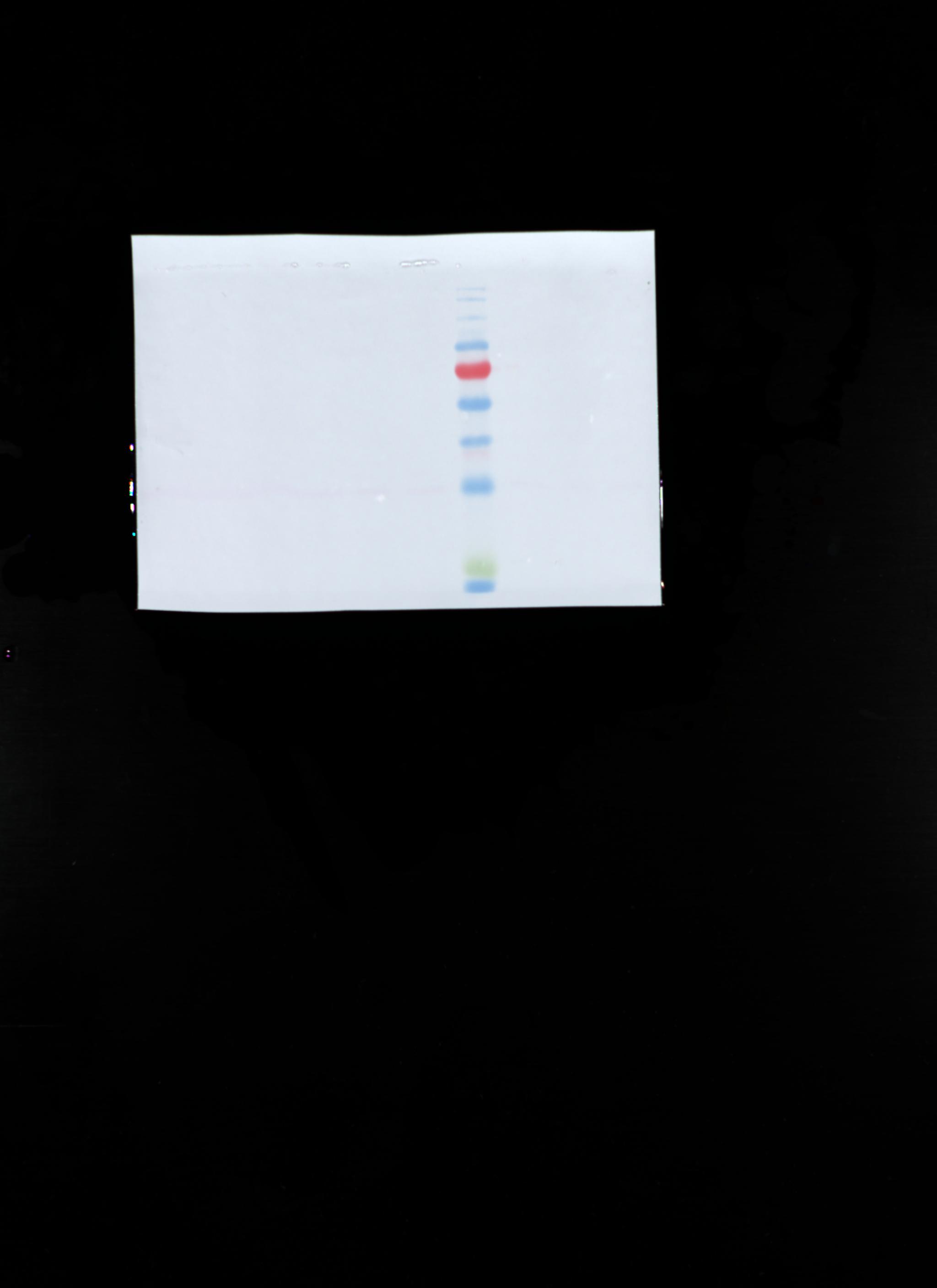

Supplement: Figure 5—source data 3. [file elife-87572-fig5-data3.zip › PCNA/Rep3/Z1-PCNA-Cy3 2022.11.30_15.25.02_Fl-Green/Z1-PCNA-Cy3 2022.11.30_15.25.02_Fl-Green-Marker.jpg]

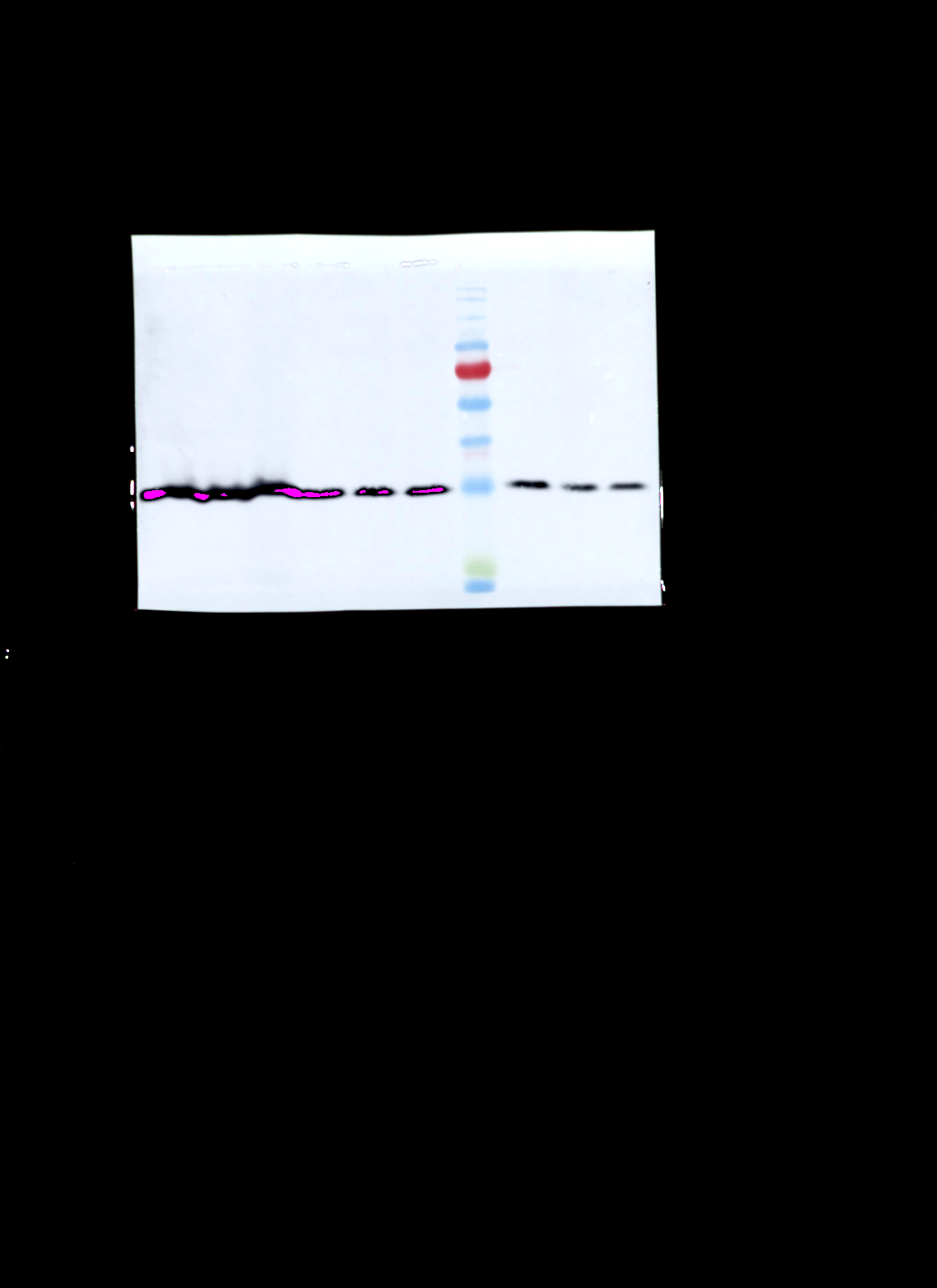

Supplement: Figure 5—source data 3. [file elife-87572-fig5-data3.zip › PCNA/Rep3/Z1-PCNA-Cy3 2022.11.30_15.25.02_Fl-Green/Z1-PCNA-Cy3 2022.11.30_15.30.25_Fl-Green+Marker.tif]

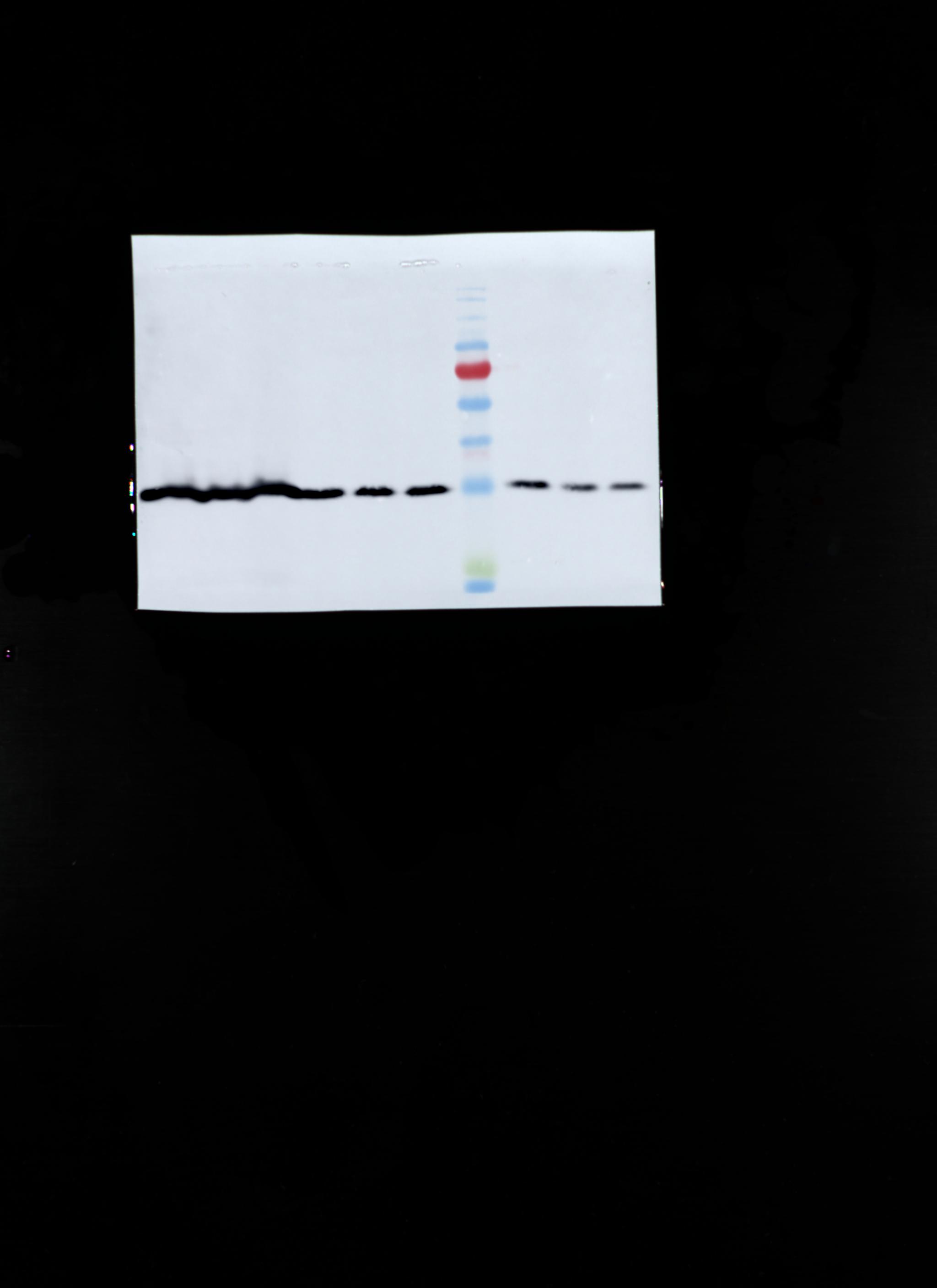

Supplement: Figure 5—source data 3. [file elife-87572-fig5-data3.zip › PCNA/Rep3/Z1-PCNA-Cy3 2022.11.30_15.25.02_Fl-Green/Z1-PCNA-Cy3 2022.11.30_15.25.02_Fl-Green+Marker.jpg]

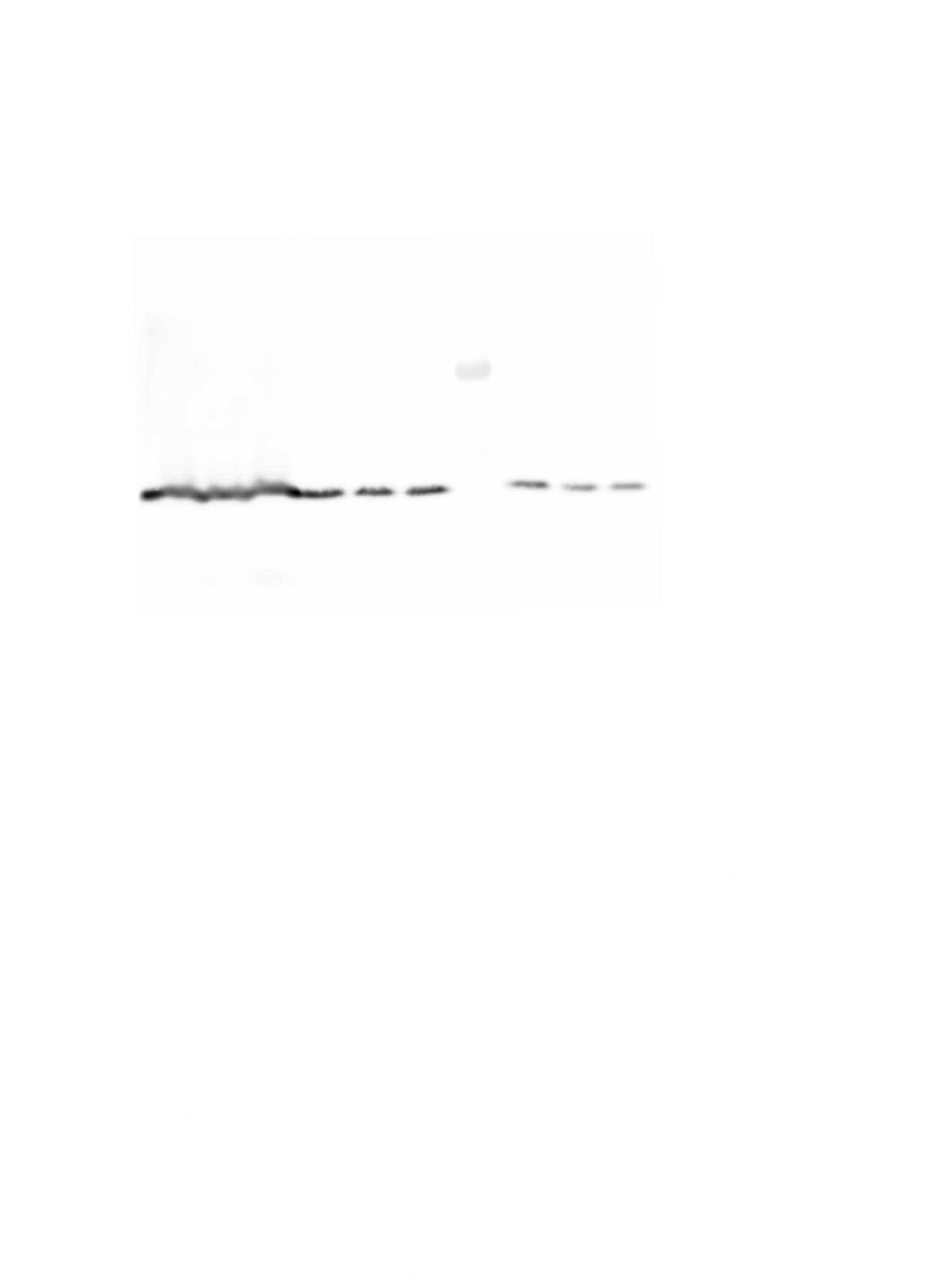

Supplement: Figure 5—source data 3. [file elife-87572-fig5-data3.zip › PCNA/Rep3/Z1-PCNA-Cy3 2022.11.30_15.25.02_Fl-Green/Z1-PCNA-Cy3 2022.11.30_15.25.02_Fl-Green.tif]

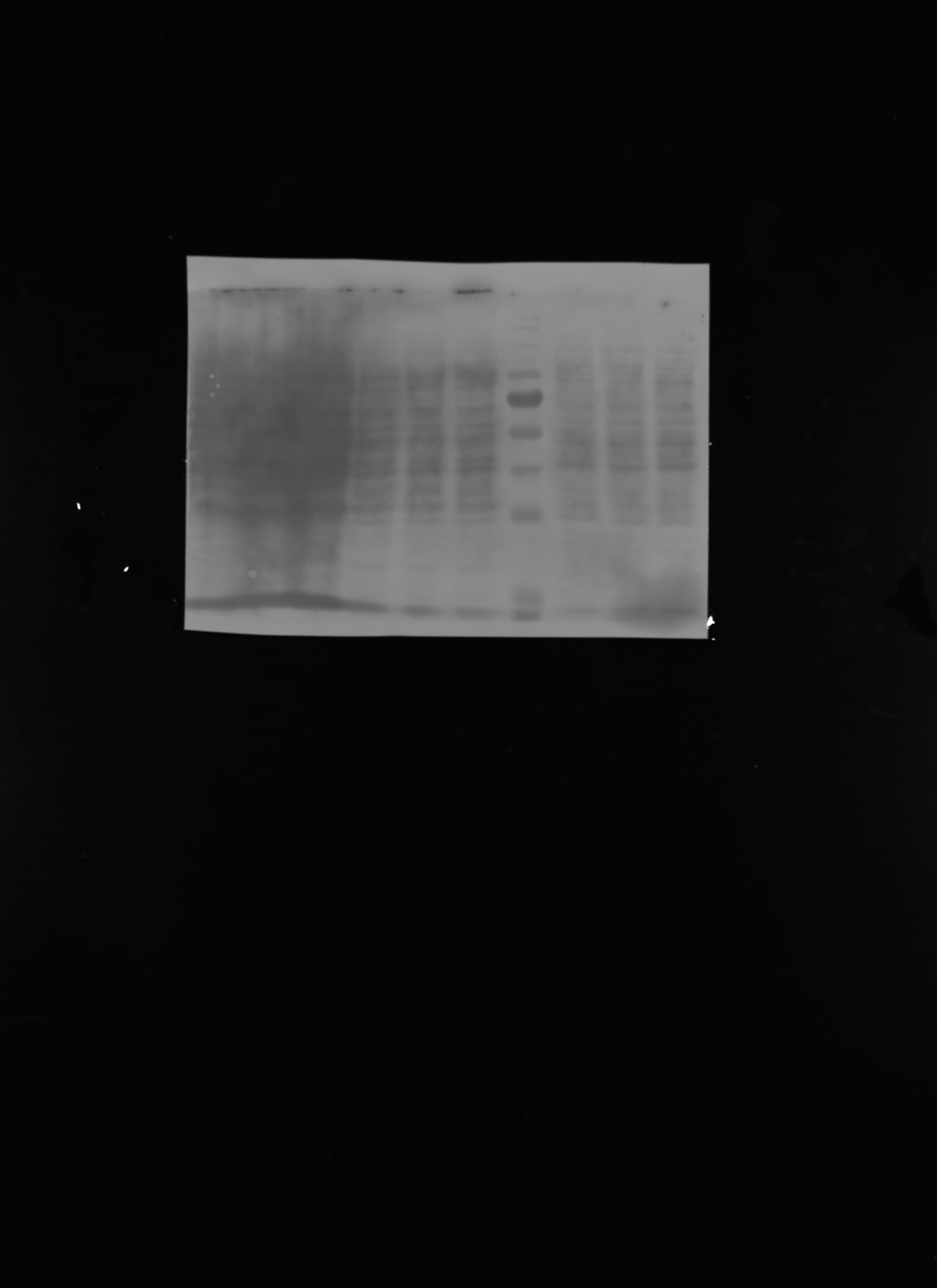

Supplement: Figure 5—source data 3. [file elife-87572-fig5-data3.zip › PCNA/Rep3/Z1-P-PCNA 2022.11.29_16.38.38_Co/Z1-P-PCNA 2022.11.29_16.38.38_Co.tif]

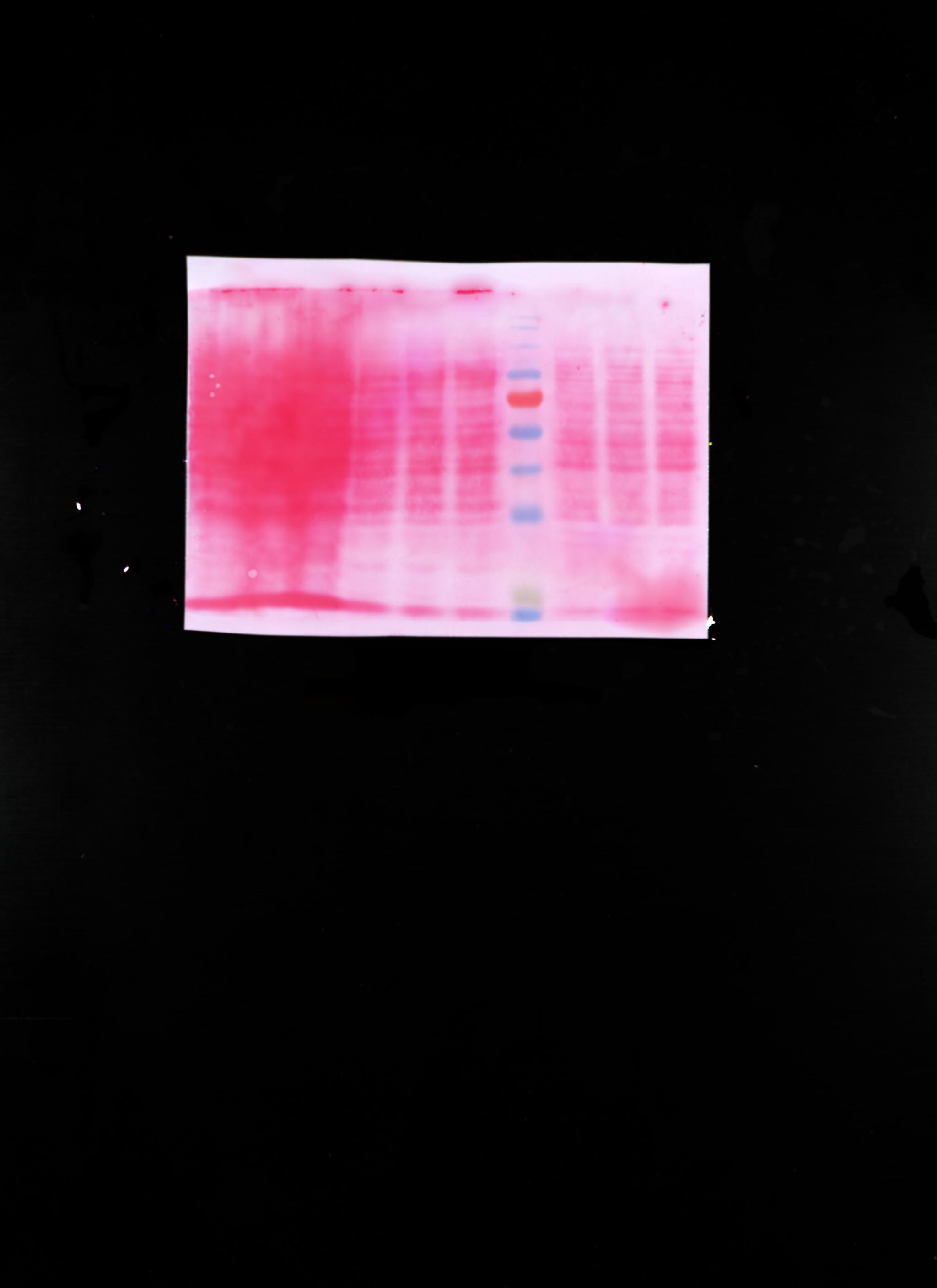

Supplement: Figure 5—source data 3. [file elife-87572-fig5-data3.zip › PCNA/Rep3/Z1-P-PCNA 2022.11.29_16.38.38_Co/Z1-P-PCNA 2022.11.29_16.38.38_Co.jpg]

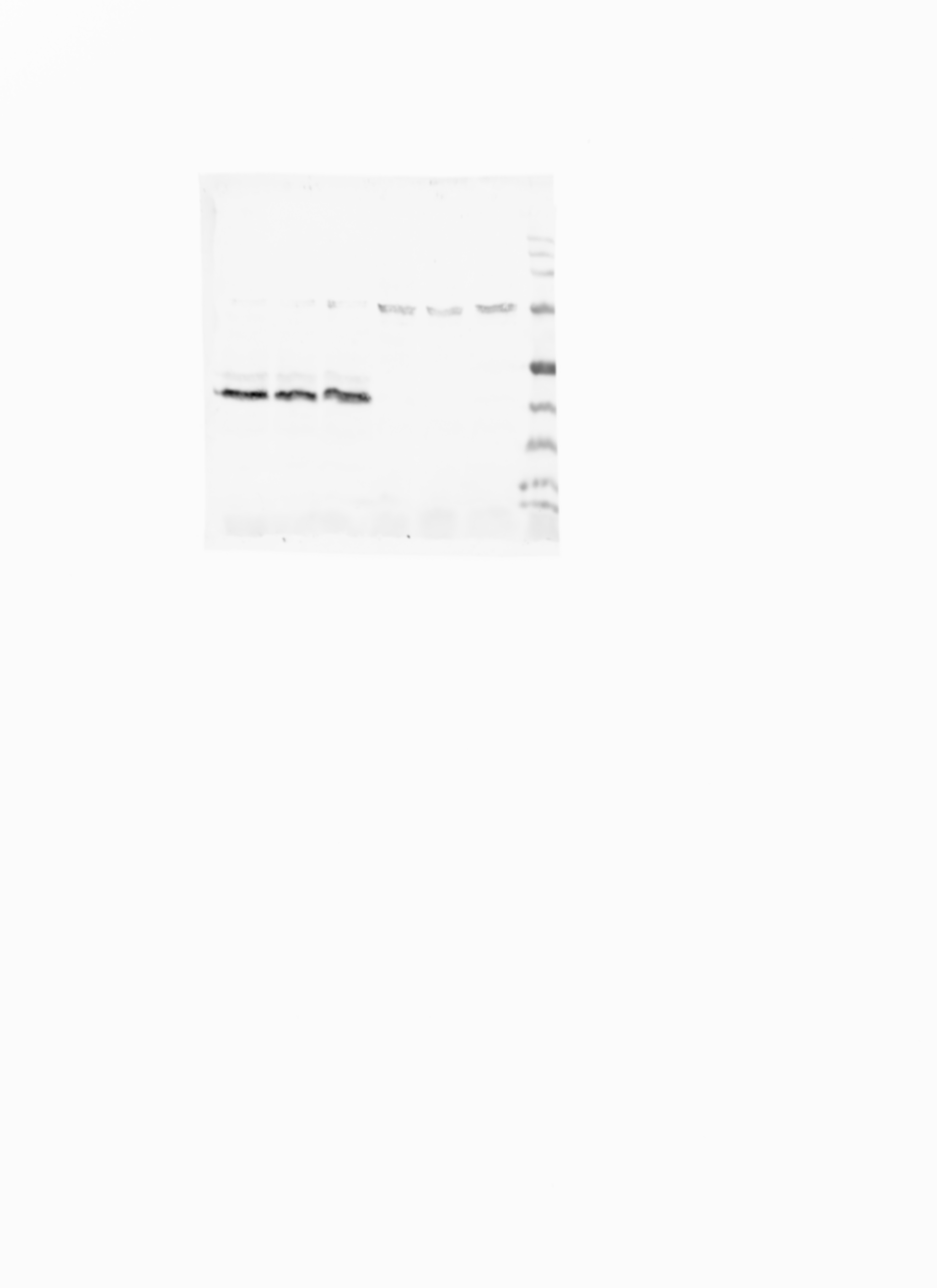

Supplement: Figure 5—source data 3. [file elife-87572-fig5-data3.zip › PCNA/Rep1/G2-mPCNACy3-rabH2A1Cy5-10%Gel-Firstprobe/G2-PCNACy3-H2a1Cy5 2022.11.11_16.24.04_Fl/G2-PCNACy3-H2a1Cy5 2022.11.11_16.24.04_Fl-Red.tif]

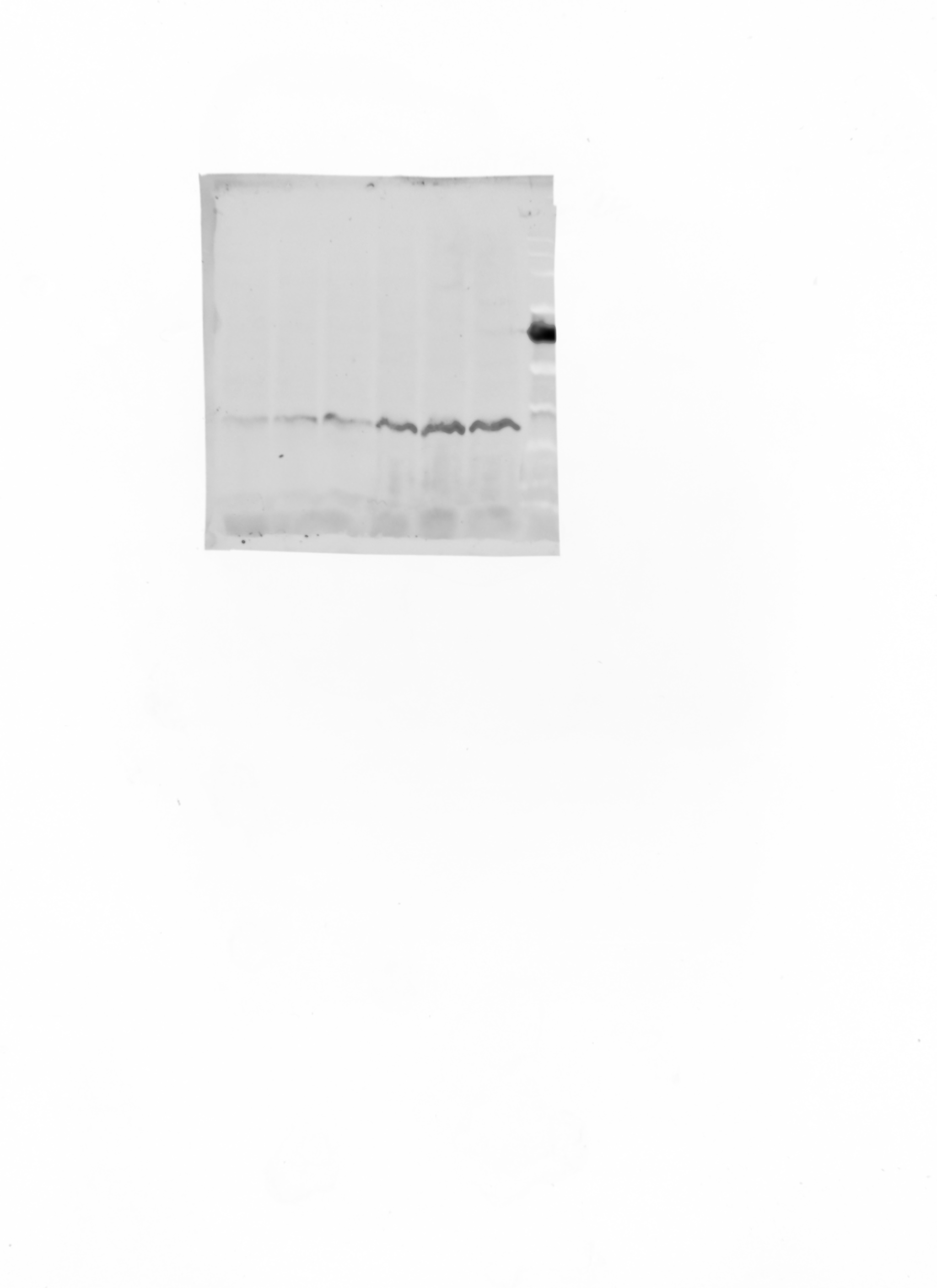

Supplement: Figure 5—source data 3. [file elife-87572-fig5-data3.zip › PCNA/Rep1/G2-mPCNACy3-rabH2A1Cy5-10%Gel-Firstprobe/G2-PCNACy3-H2a1Cy5 2022.11.11_16.24.04_Fl/G2-PCNACy3-H2a1Cy5 2022.11.11_16.24.04_Fl-Green.tif]

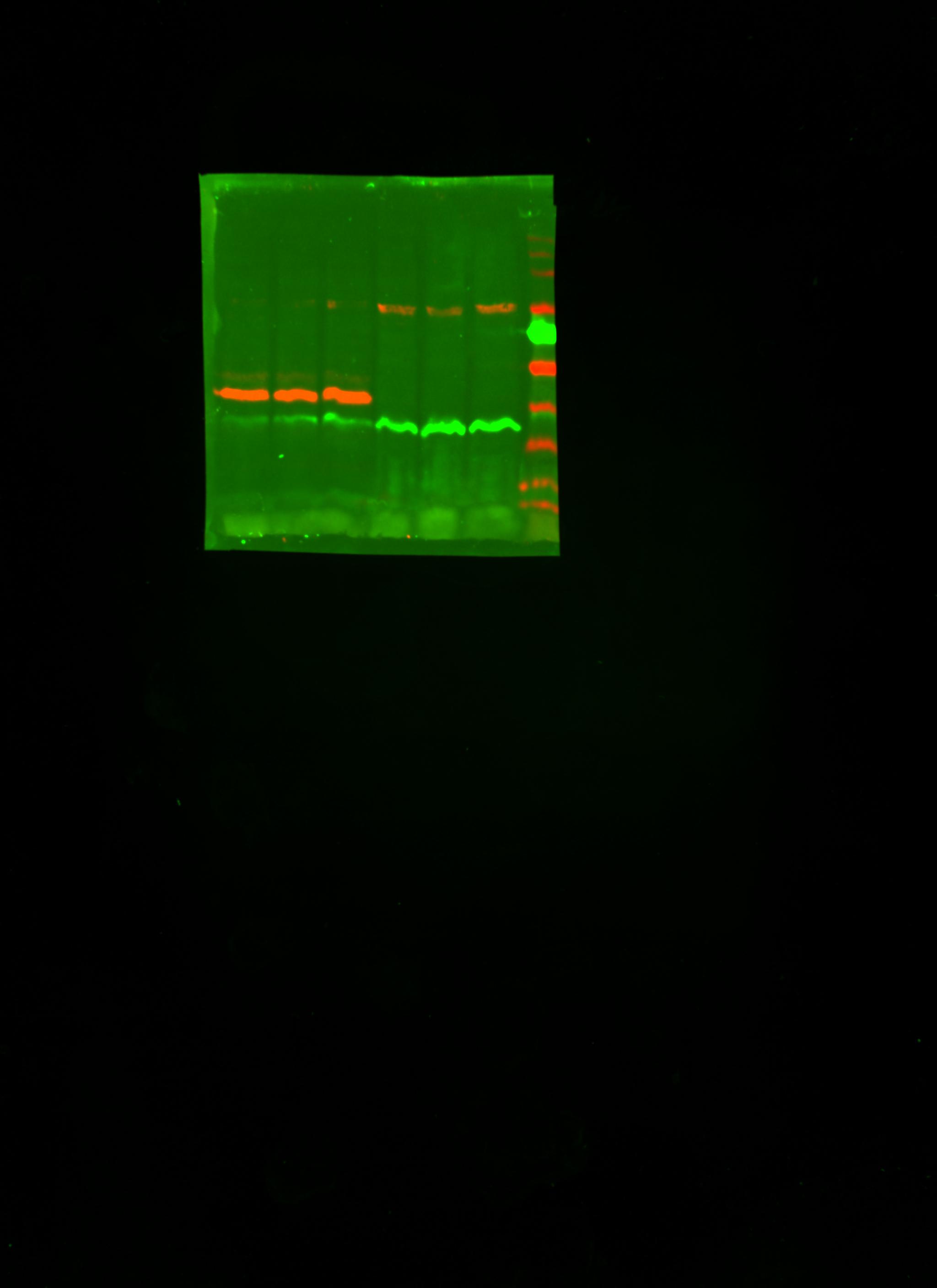

Supplement: Figure 5—source data 3. [file elife-87572-fig5-data3.zip › PCNA/Rep1/G2-mPCNACy3-rabH2A1Cy5-10%Gel-Firstprobe/G2-PCNACy3-H2a1Cy5 2022.11.11_16.24.04_Fl/G2-PCNACy3-H2a1Cy5 2022.11.11_16.24.04_Fl.jpg]

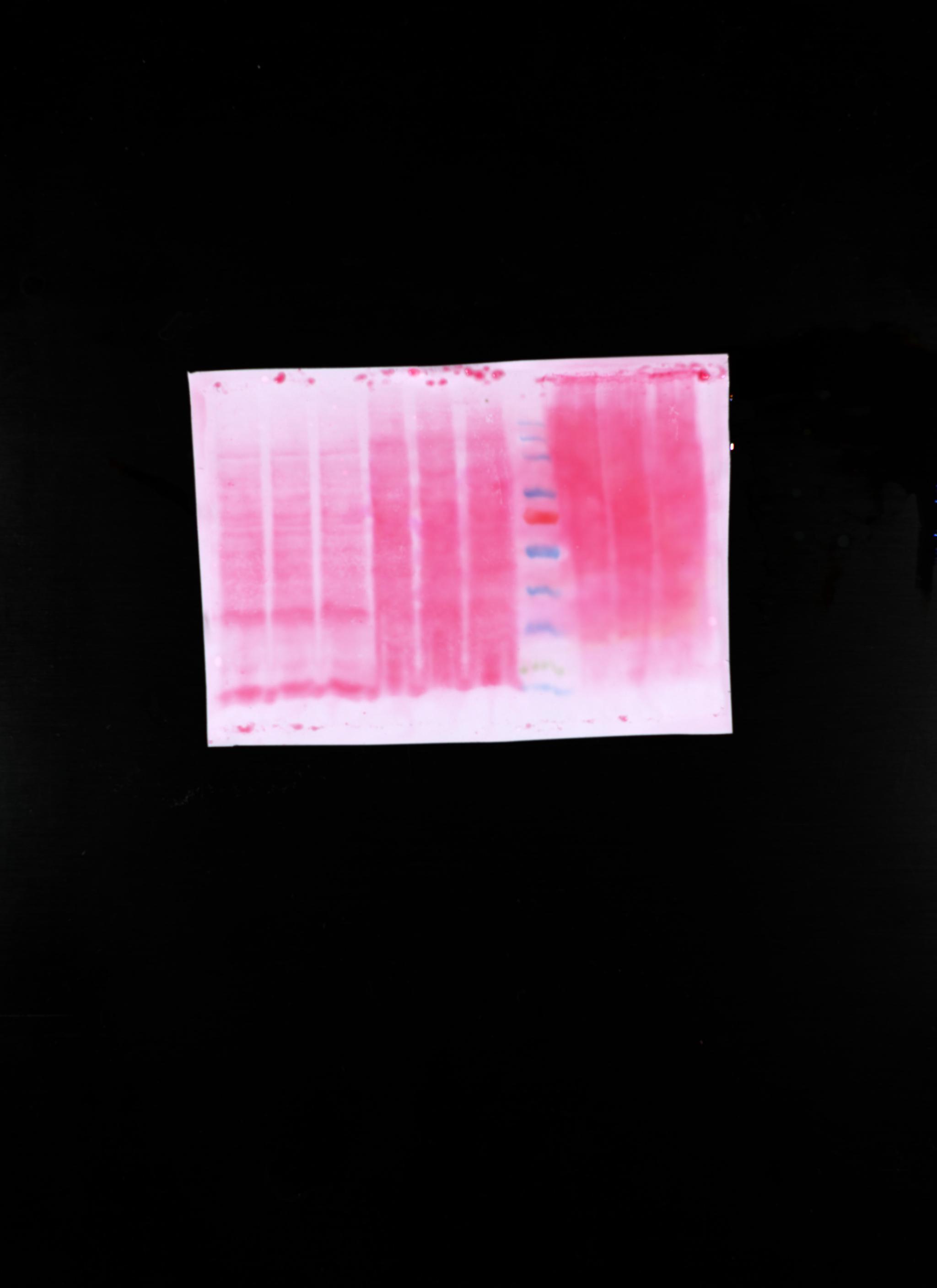

Supplement: Figure 5—source data 3. [file elife-87572-fig5-data3.zip › PCNA/Rep1/G2-mPCNACy3-rabH2A1Cy5-10%Gel-Firstprobe/P-G2-mPCNA-rbHis-10% 2022.11.10_17.28.08_Co/P-G2-mPCNA-rbHis-10% 2022.11.10_17.28.08_Co.jpg]

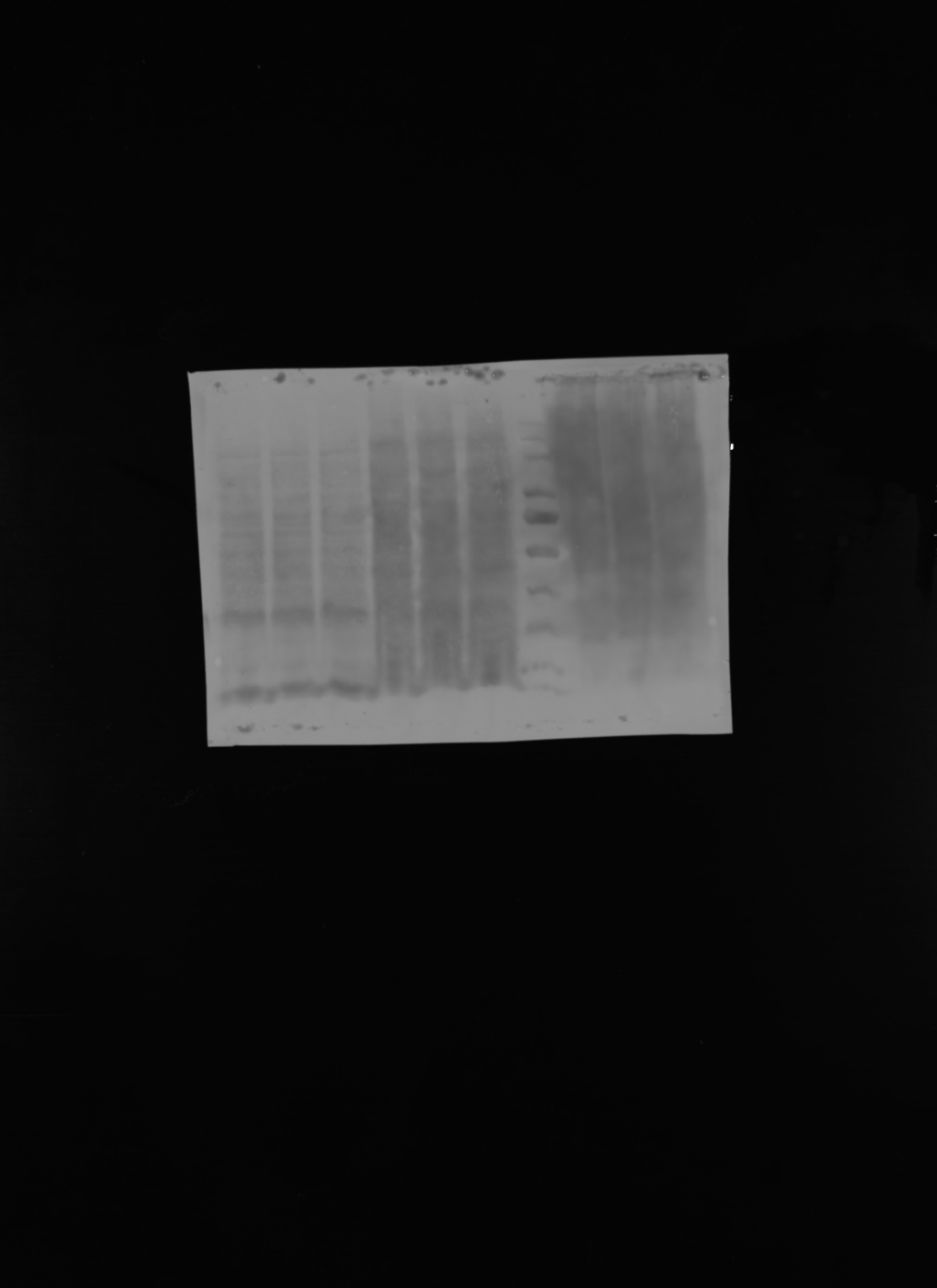

Supplement: Figure 5—source data 3. [file elife-87572-fig5-data3.zip › PCNA/Rep1/G2-mPCNACy3-rabH2A1Cy5-10%Gel-Firstprobe/P-G2-mPCNA-rbHis-10% 2022.11.10_17.28.08_Co/P-G2-mPCNA-rbHis-10% 2022.11.10_17.28.08_Co.tif]

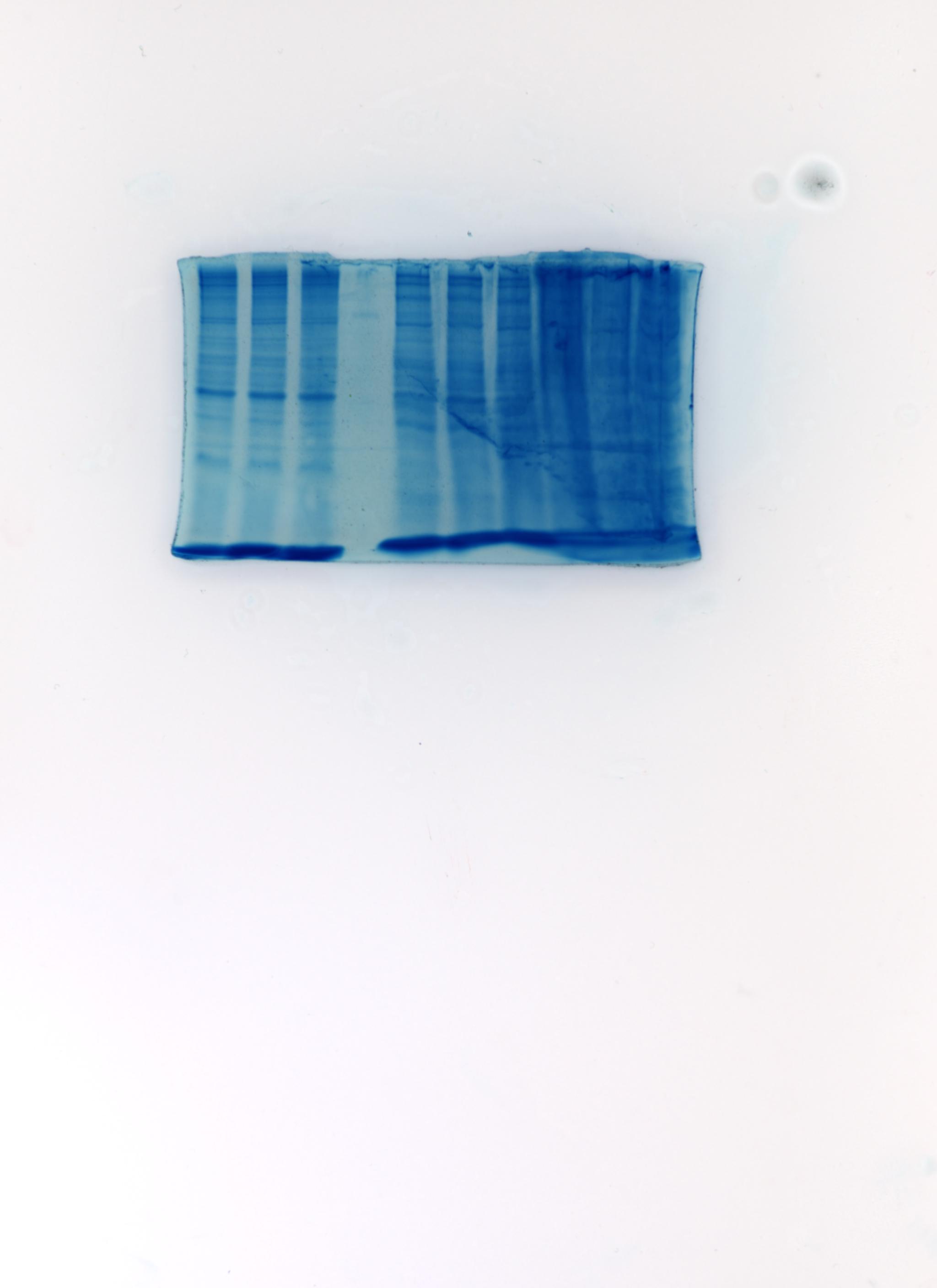

Supplement: Figure 5—source data 4. [file elife-87572-fig5-data4.zip › PolA/Rep2/Z6-C-polE,A 2022.12.04_15.39.05_Co/Z6-C-polE,A 2022.12.04_15.39.05_Co.jpg]

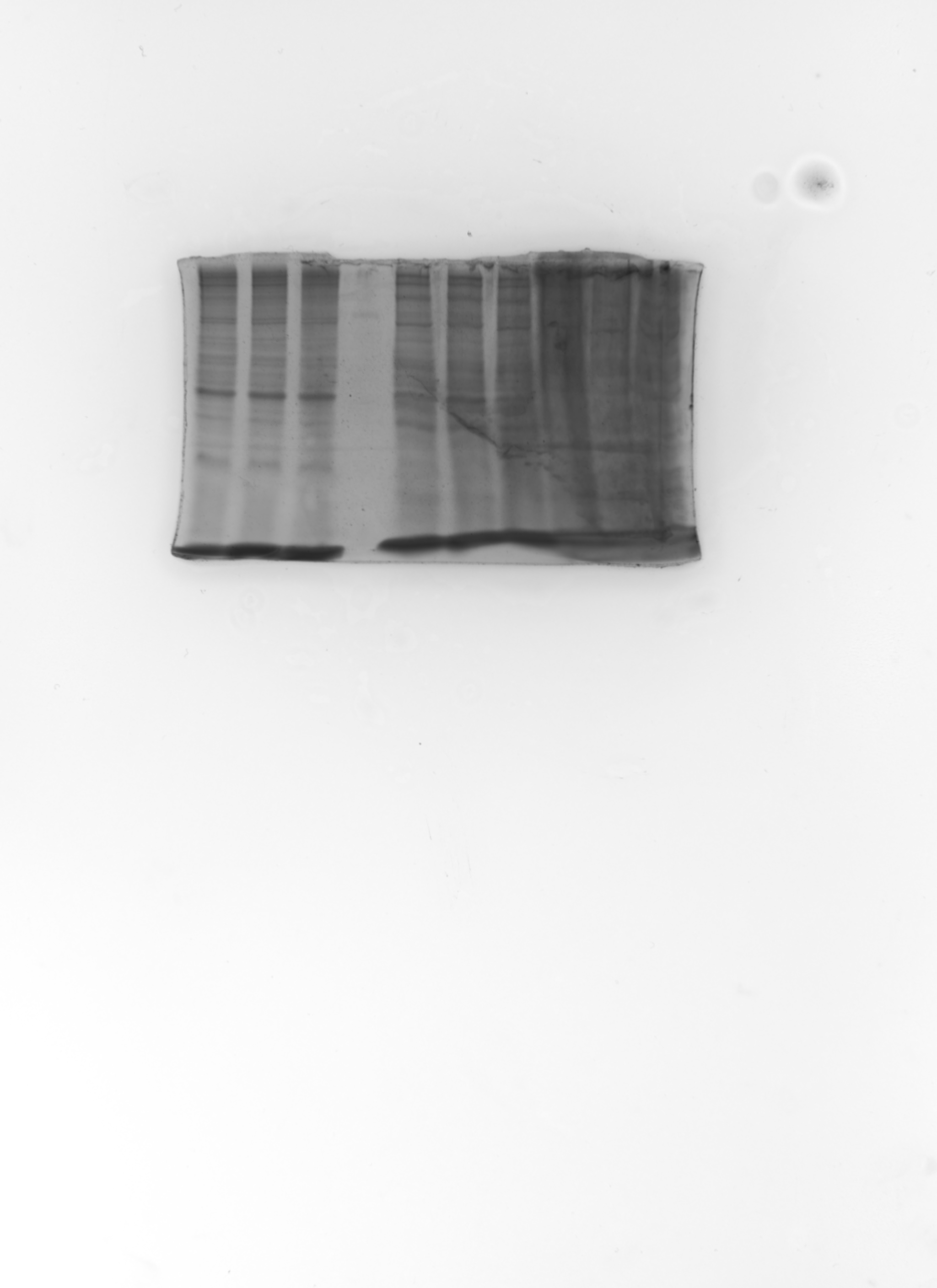

Supplement: Figure 5—source data 4. [file elife-87572-fig5-data4.zip › PolA/Rep2/Z6-C-polE,A 2022.12.04_15.39.05_Co/Z6-C-polE,A 2022.12.04_15.39.05_Co.tif]

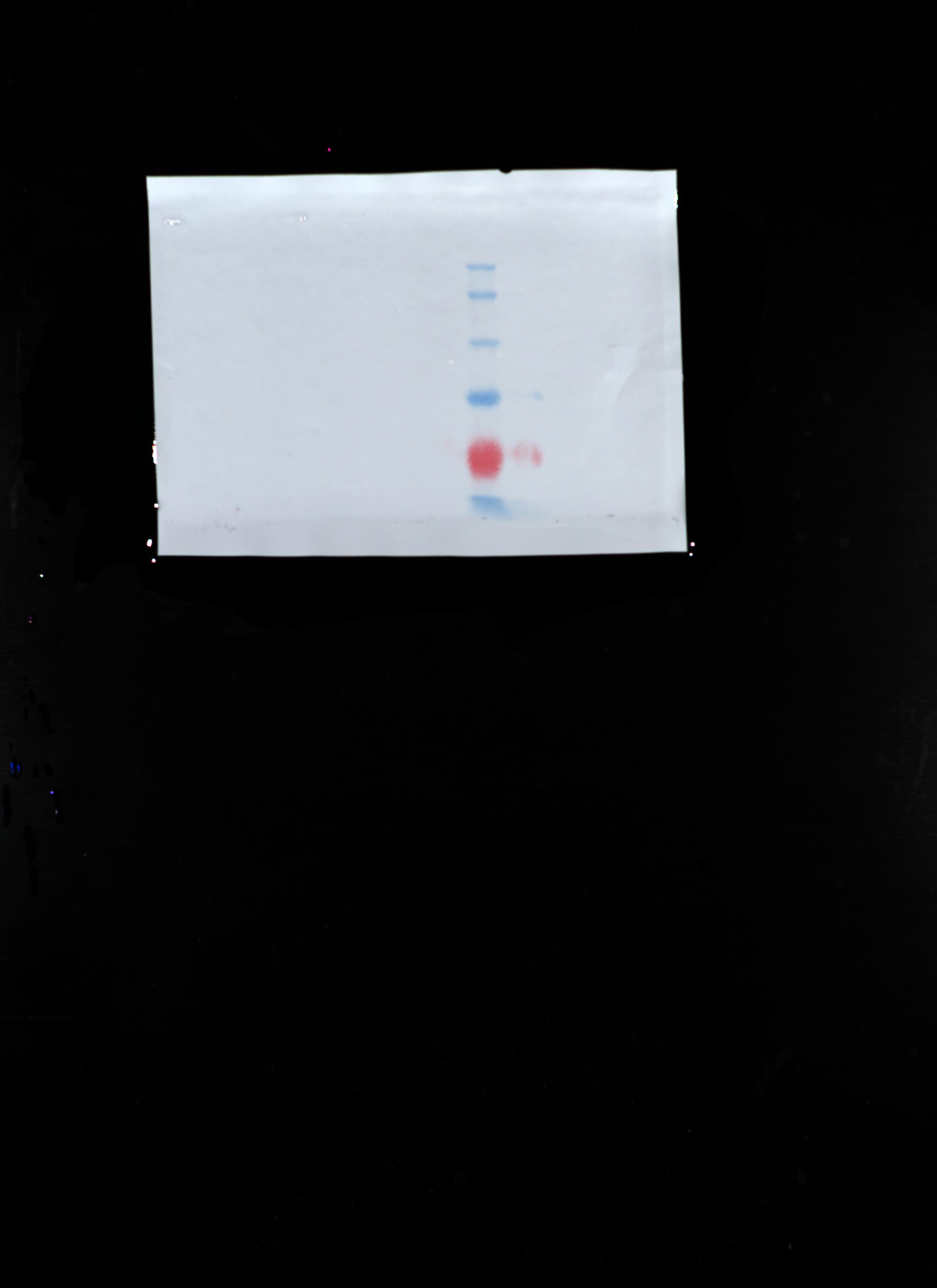

Supplement: Figure 5—source data 4. [file elife-87572-fig5-data4.zip › PolA/Rep2/Z6-polA-Cy5 2022.12.01_17.13.37_Fl-Red/Z6-polA-Cy5 2022.12.01_17.13.37_Fl-Red-Marker.jpg]

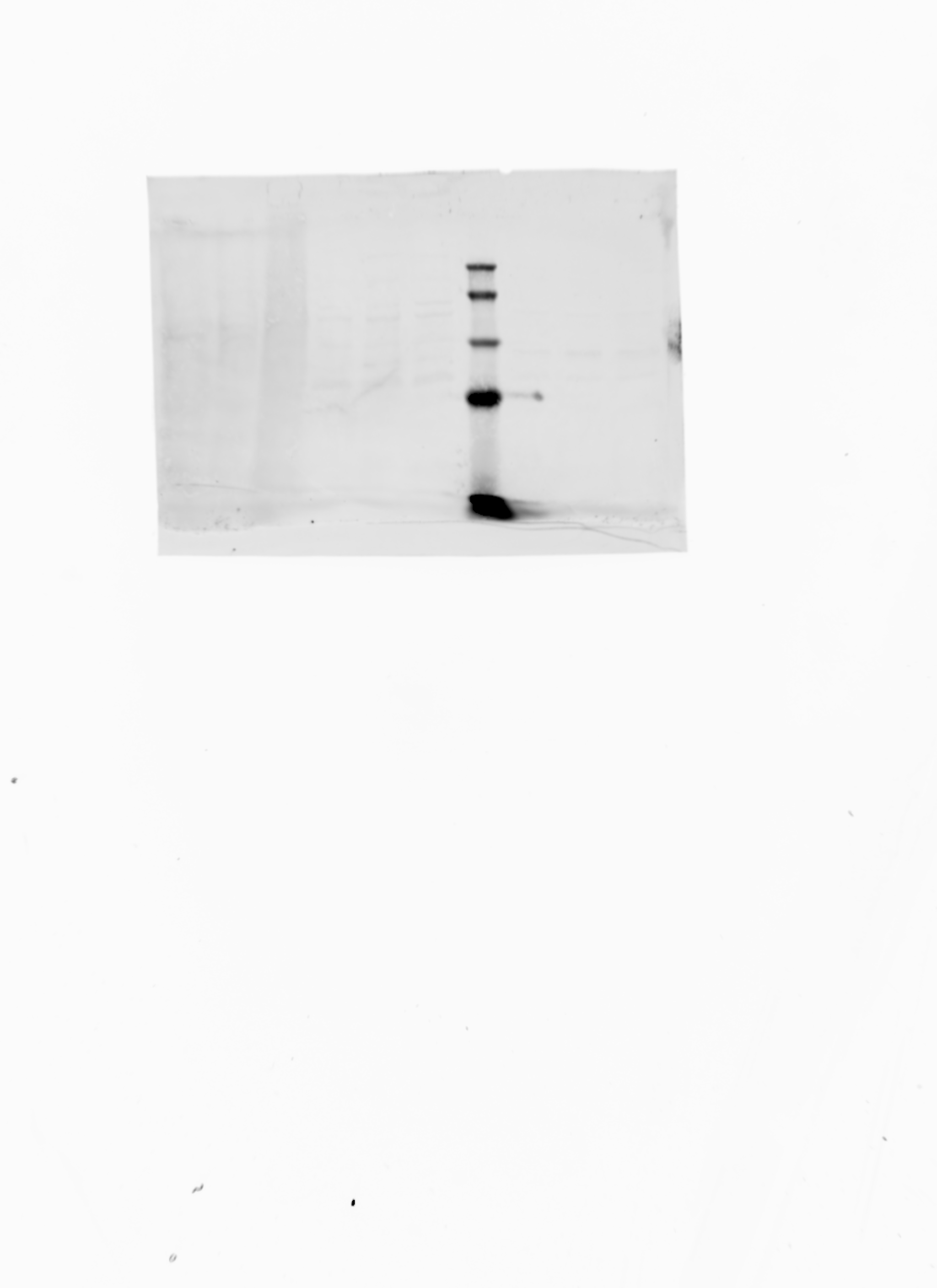

Supplement: Figure 5—source data 4. [file elife-87572-fig5-data4.zip › PolA/Rep2/Z6-polA-Cy5 2022.12.01_17.13.37_Fl-Red/Z6-polA-Cy5 2022.12.01_17.13.37_Fl-Red.tif]

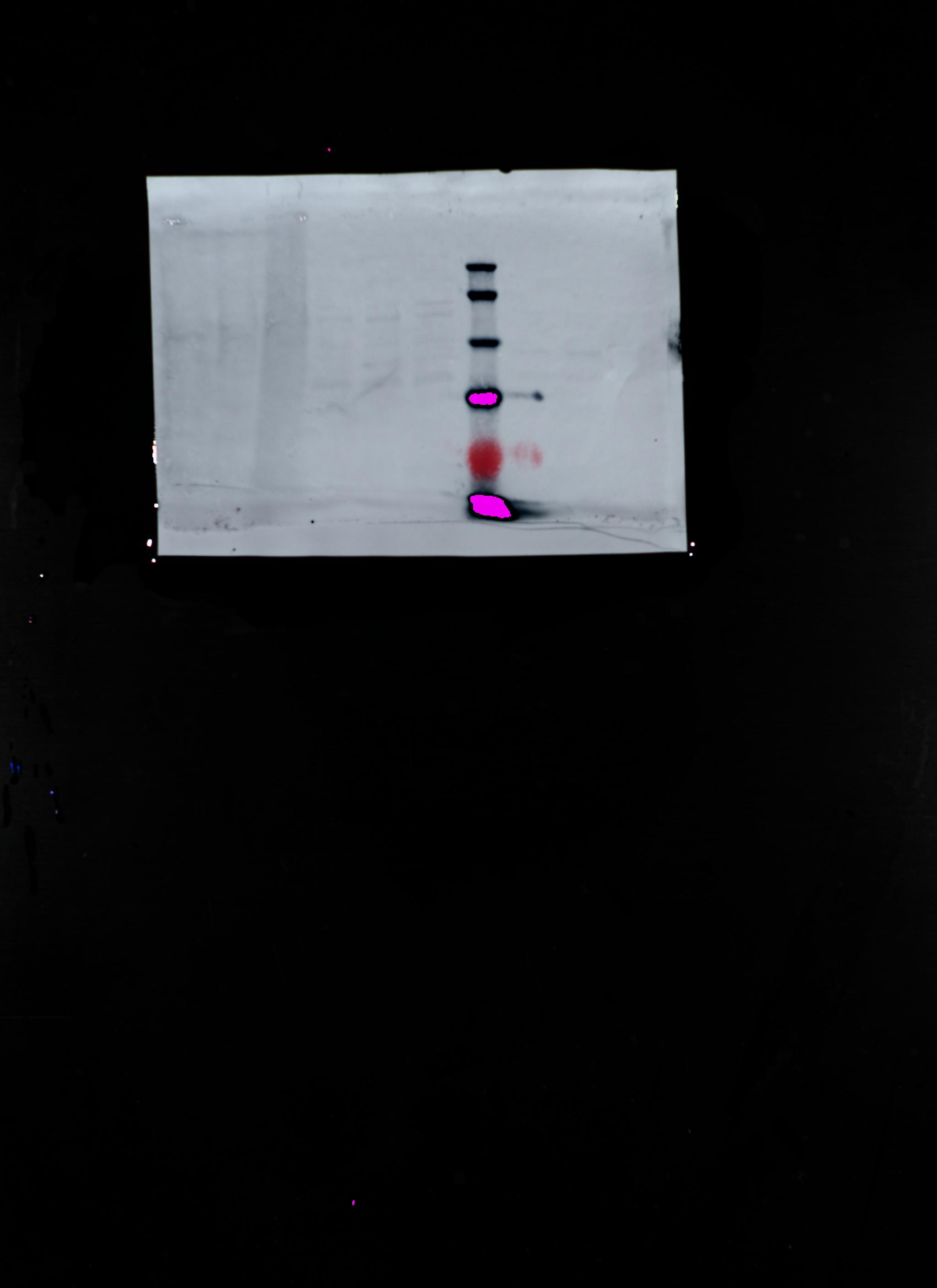

Supplement: Figure 5—source data 4. [file elife-87572-fig5-data4.zip › PolA/Rep2/Z6-polA-Cy5 2022.12.01_17.13.37_Fl-Red/Z6-polA-Cy5 2022.12.01_17.13.37_Fl-Red+Marker.jpg]

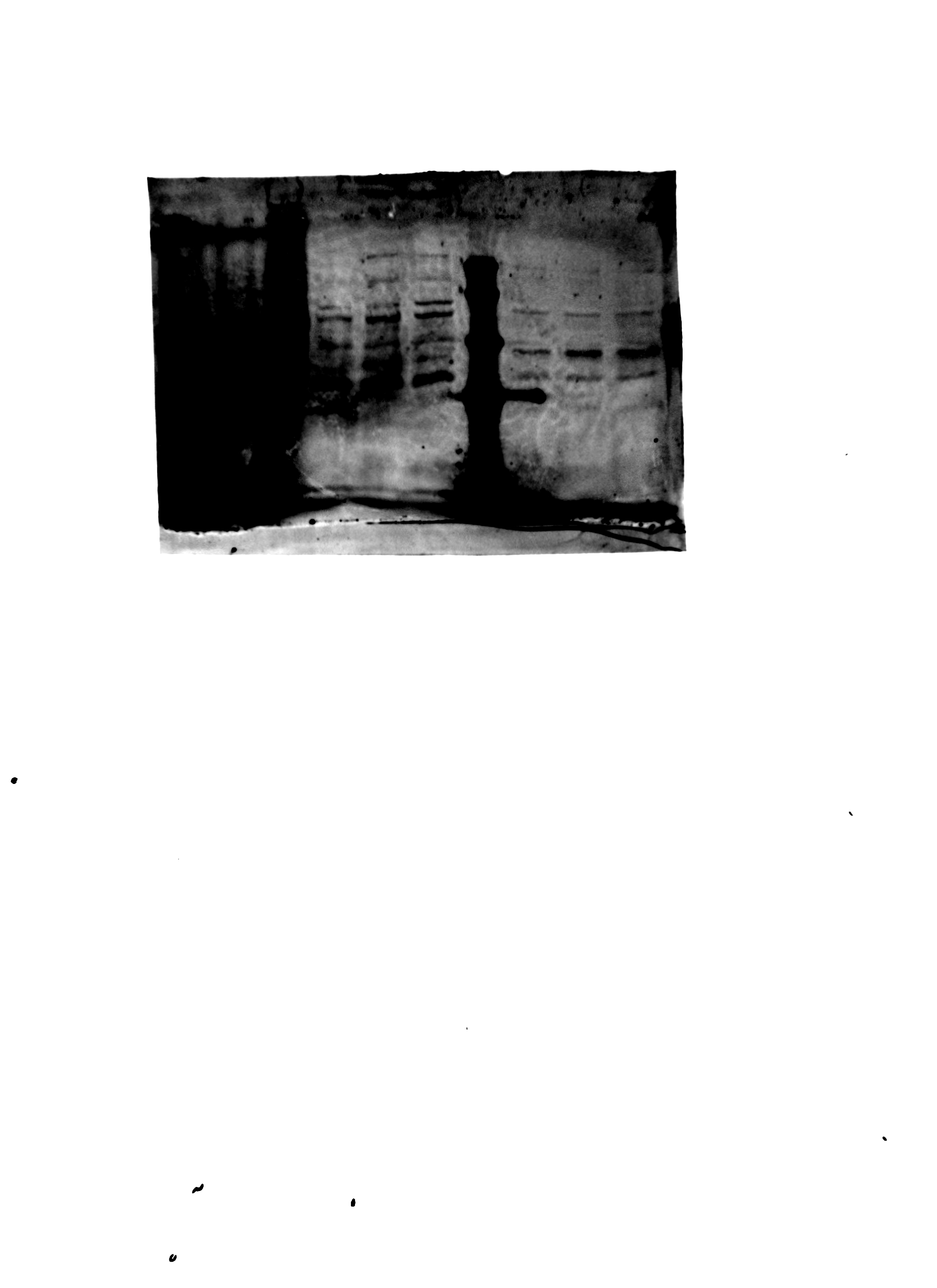

Supplement: Figure 5—source data 4. [file elife-87572-fig5-data4.zip › PolA/Rep2/Z6-polA-Cy5 2022.12.01_17.13.37_Fl-Red/newZ6-polA-Cy5 2022.12.01_17.13.37_Fl-Red.tif]

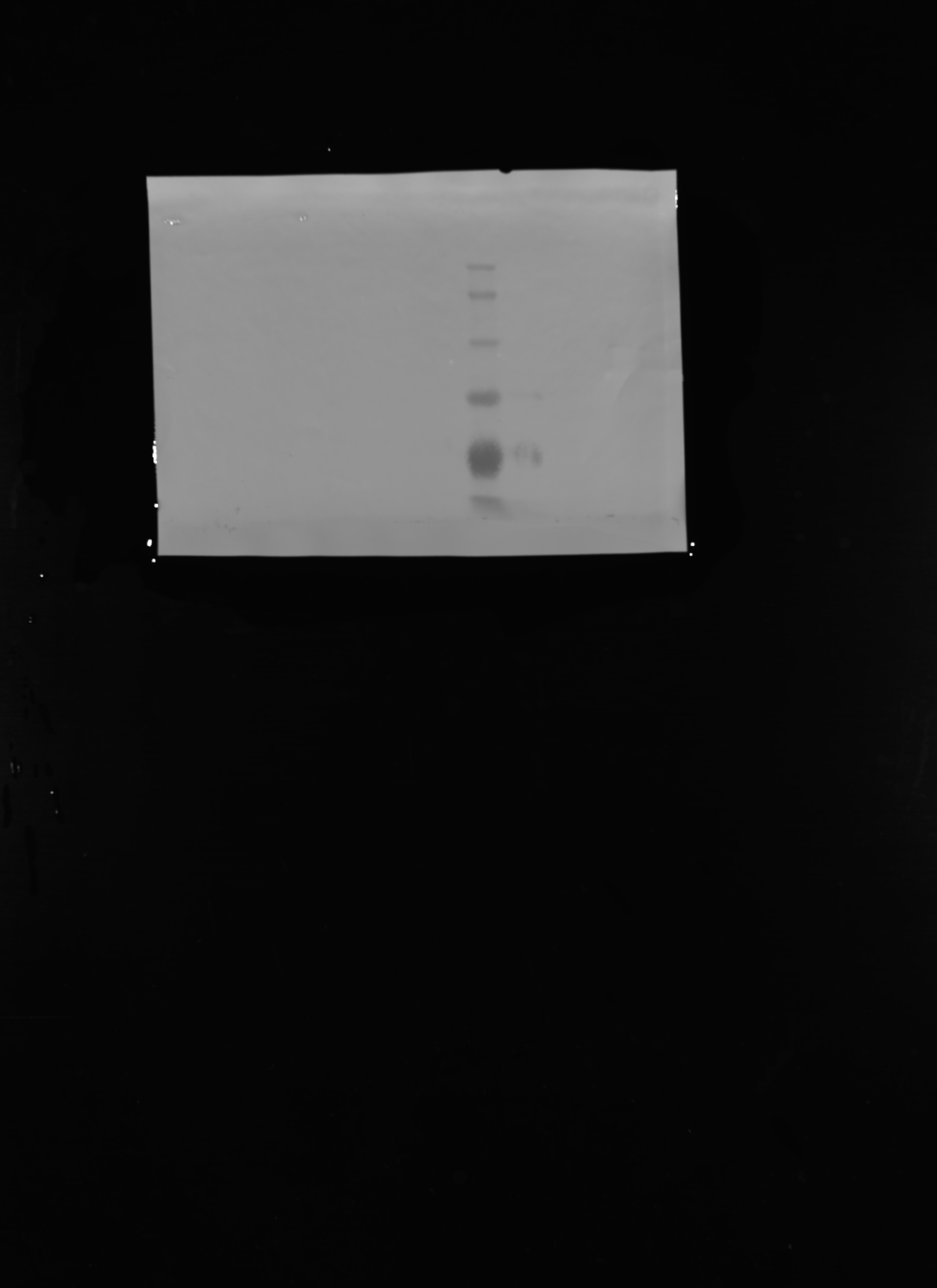

Supplement: Figure 5—source data 4. [file elife-87572-fig5-data4.zip › PolA/Rep2/Z6-polA-Cy5 2022.12.01_17.13.37_Fl-Red/Z6-polA-Cy5 2022.12.01_17.13.37_Fl-Red-Marker.tif]

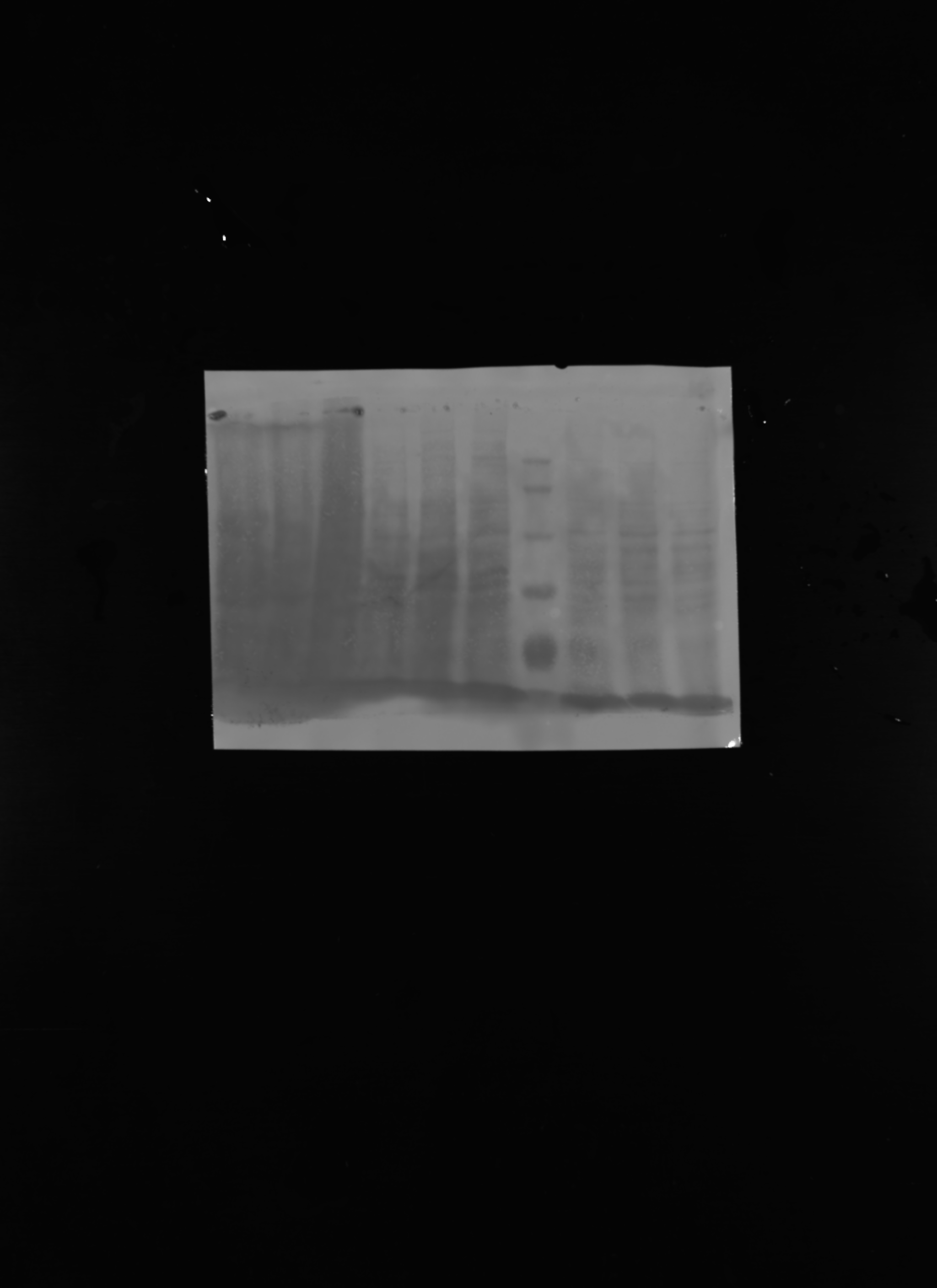

Supplement: Figure 5—source data 4. [file elife-87572-fig5-data4.zip › PolA/Rep2/Z6-P-PolE 2022.11.29_16.31.32_Co/Z6-P-PolE 2022.11.29_16.31.32_Co.tif]

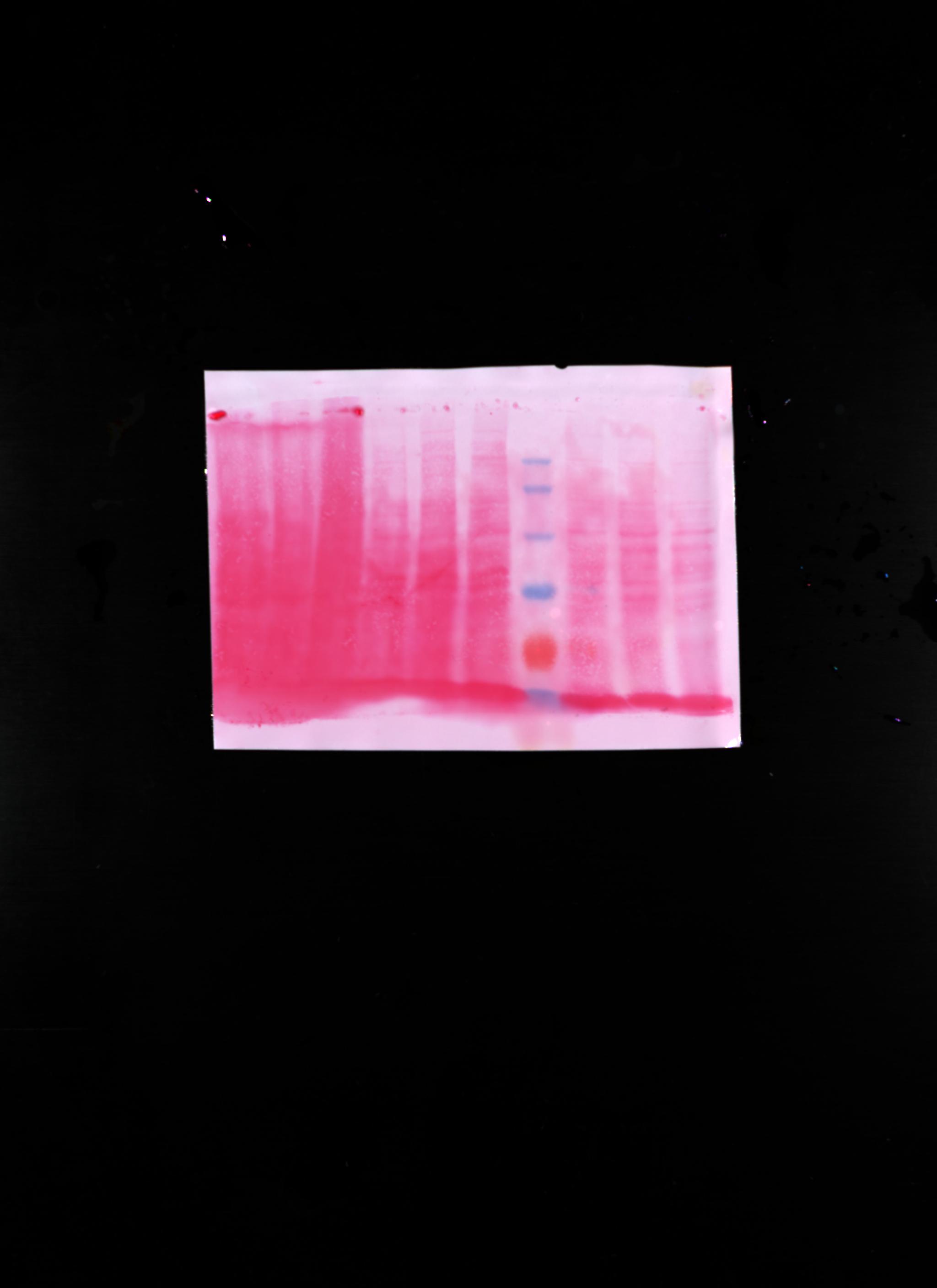

Supplement: Figure 5—source data 4. [file elife-87572-fig5-data4.zip › PolA/Rep2/Z6-P-PolE 2022.11.29_16.31.32_Co/Z6-P-PolE 2022.11.29_16.31.32_Co.jpg]

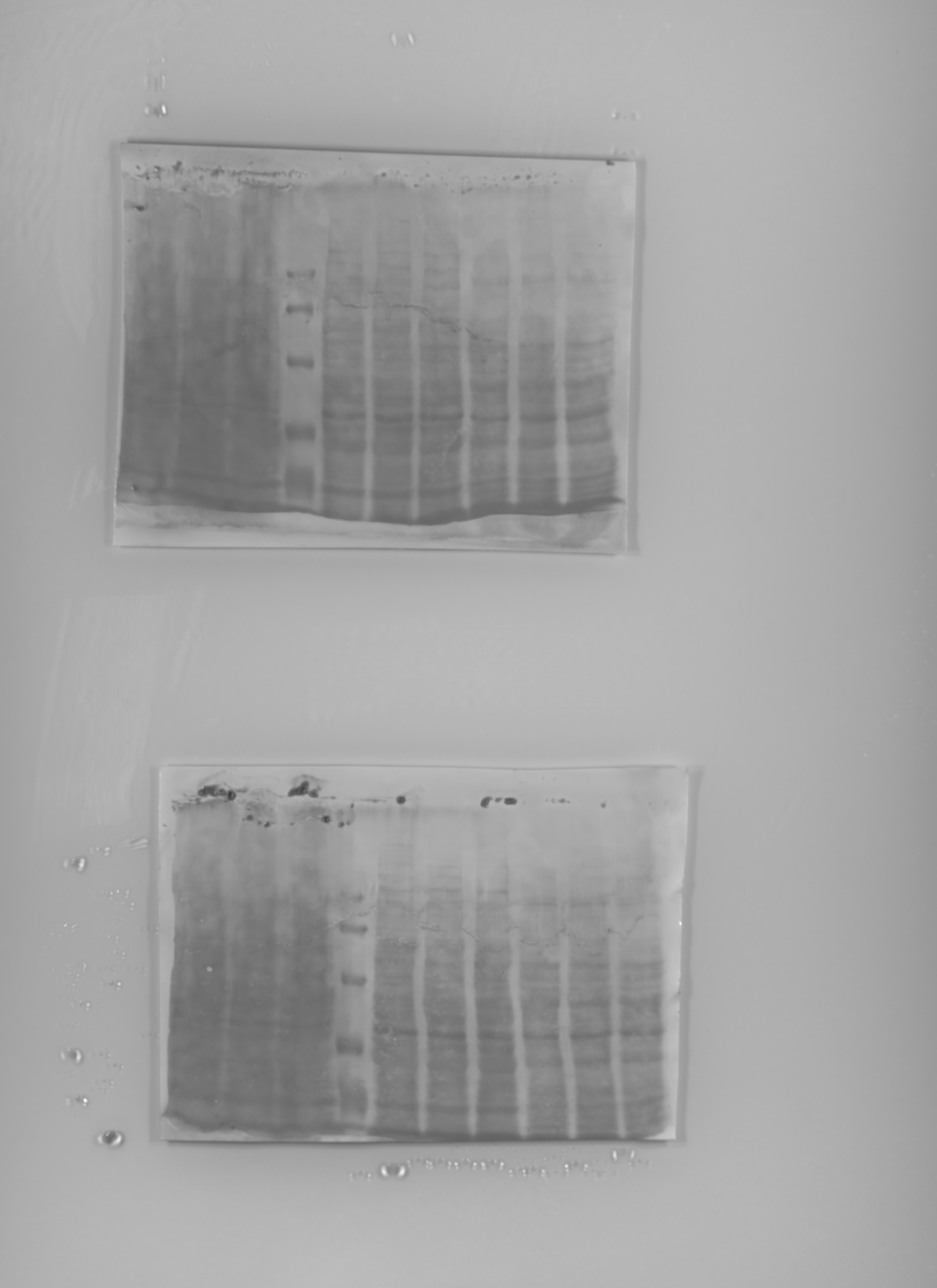

Supplement: Figure 5—source data 4. [file elife-87572-fig5-data4.zip › PolA/Rep1/gel1-2 6% pon mk 2022.08.16_14.38.33_Co/gel1-2 6% pon mk 2022.08.16_14.38.33_Co.tif]

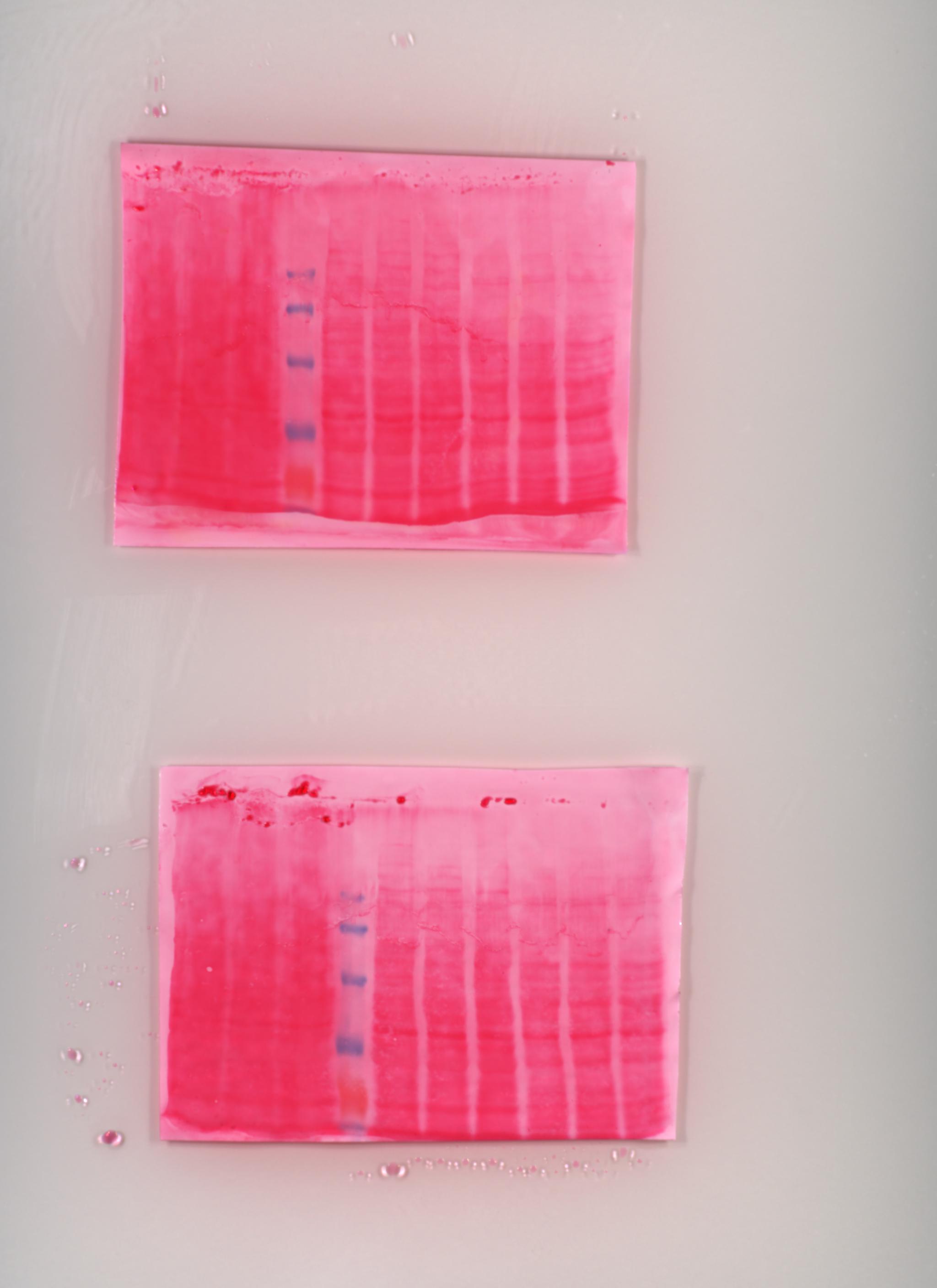

Supplement: Figure 5—source data 4. [file elife-87572-fig5-data4.zip › PolA/Rep1/gel1-2 6% pon mk 2022.08.16_14.38.33_Co/gel1-2 6% pon mk 2022.08.16_14.38.33_Co.jpg]

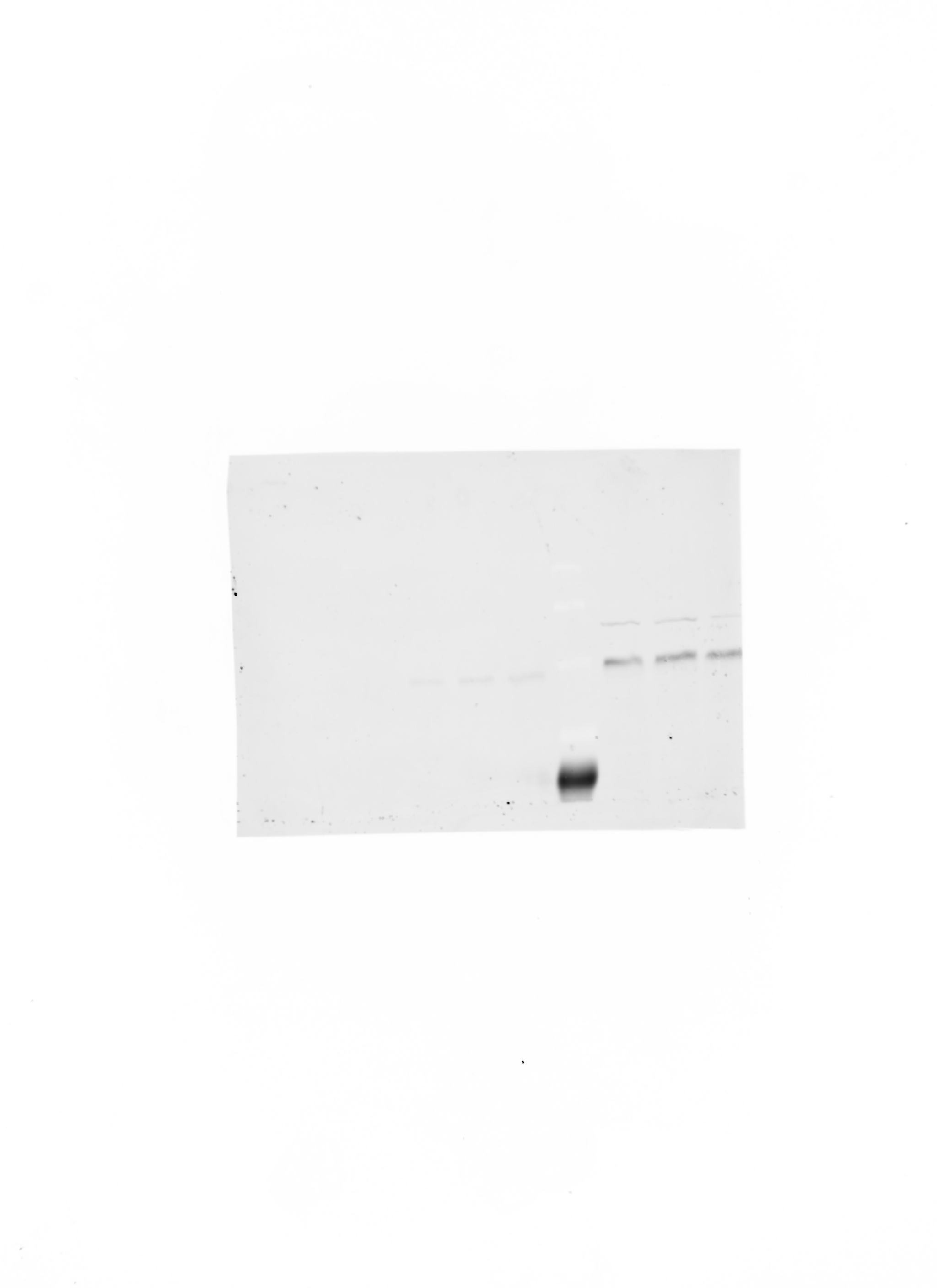

Supplement: Figure 5—source data 4. [file elife-87572-fig5-data4.zip › PolA/Rep1/AK MCM2 PolE,A Lig1 2022.07.28_12.24.23_Fl/AK MCM2 PolE,A Lig1 2022.07.28_12.24.23_Fl-Green.tif]

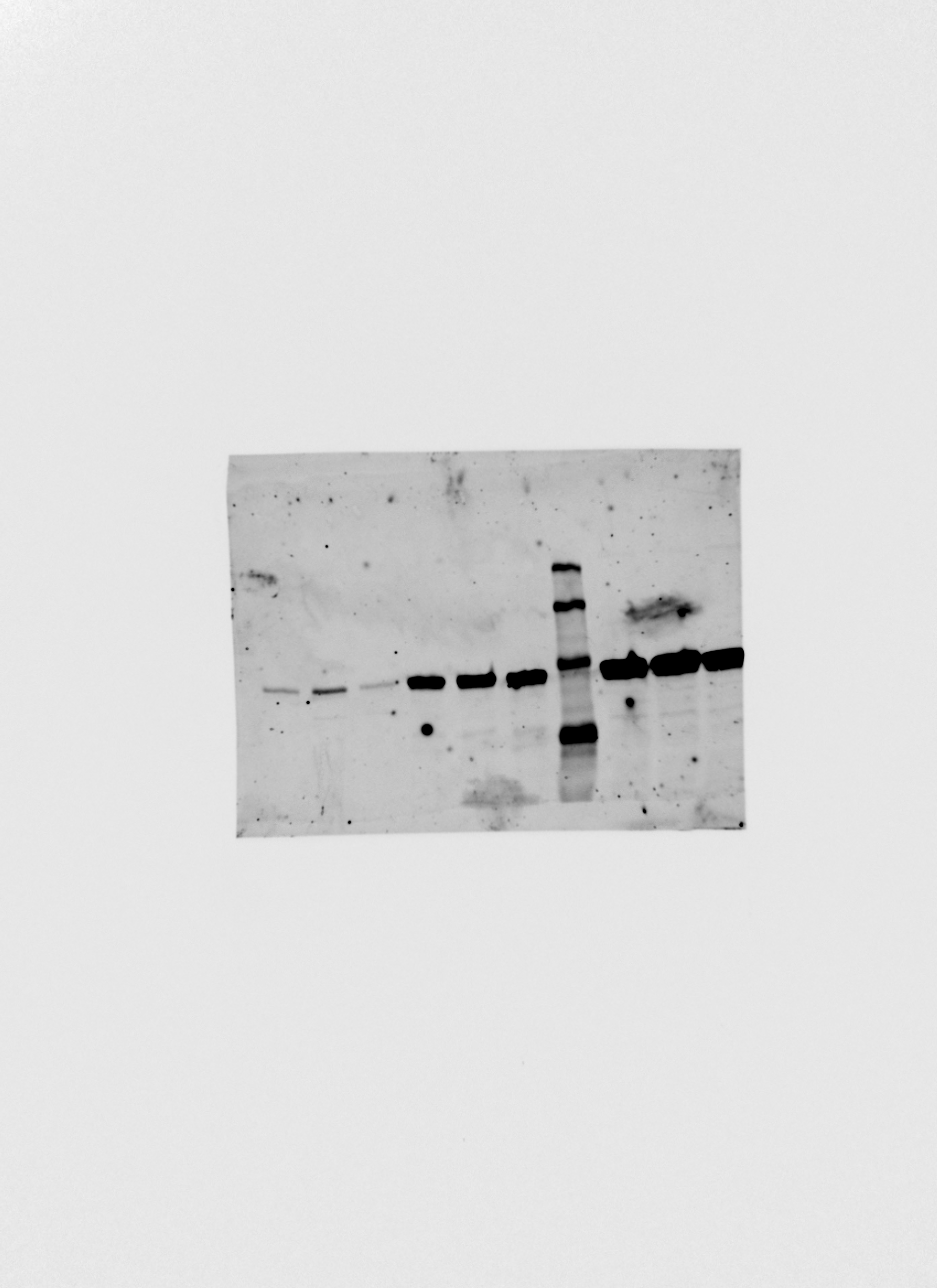

Supplement: Figure 5—source data 4. [file elife-87572-fig5-data4.zip › PolA/Rep1/AK MCM2 PolE,A Lig1 2022.07.28_12.24.23_Fl/AK MCM2 PolE,A Lig1 2022.07.28_12.24.23_Fl-Red.tif]

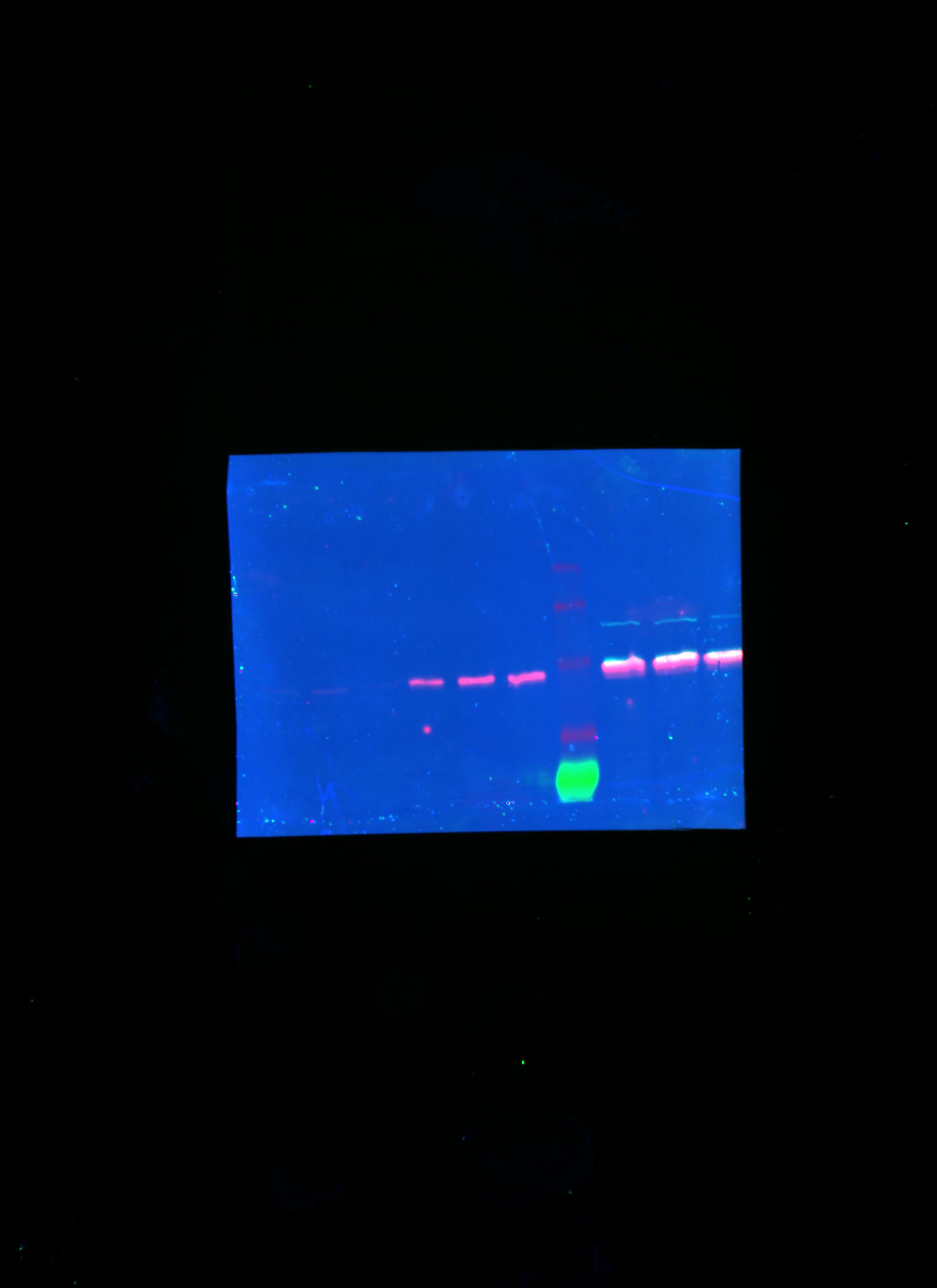

Supplement: Figure 5—source data 4. [file elife-87572-fig5-data4.zip › PolA/Rep1/AK MCM2 PolE,A Lig1 2022.07.28_12.24.23_Fl/AK MCM2 PolE,A Lig1 2022.07.28_12.24.23_Fl.jpg]

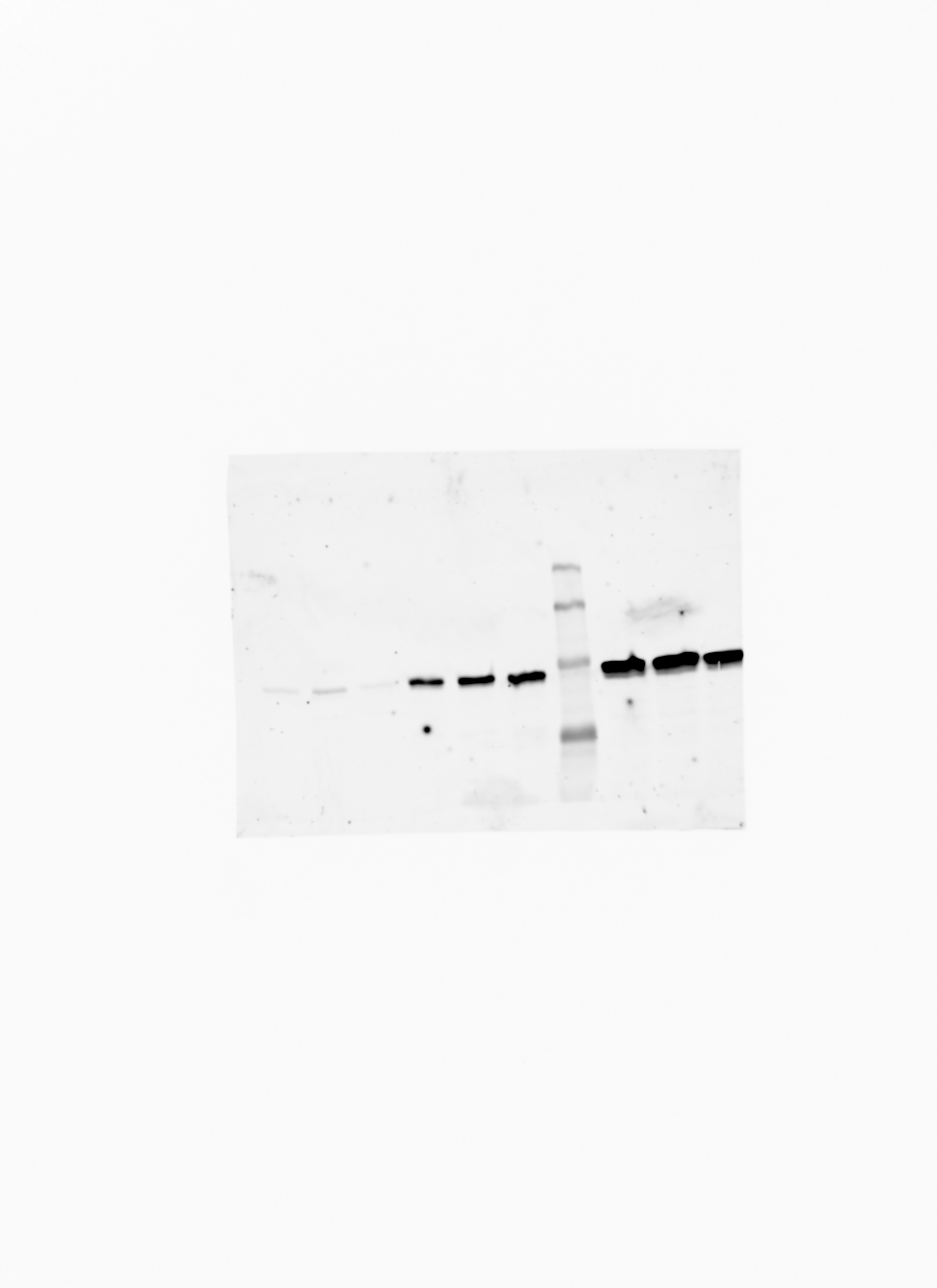

Supplement: Figure 5—source data 4. [file elife-87572-fig5-data4.zip › PolA/Rep1/AK MCM2 PolE,A Lig1 2022.07.28_12.24.23_Fl/AK MCM2 PolE,A Lig1 2022.07.28_12.24.23_Fl-Red.png]

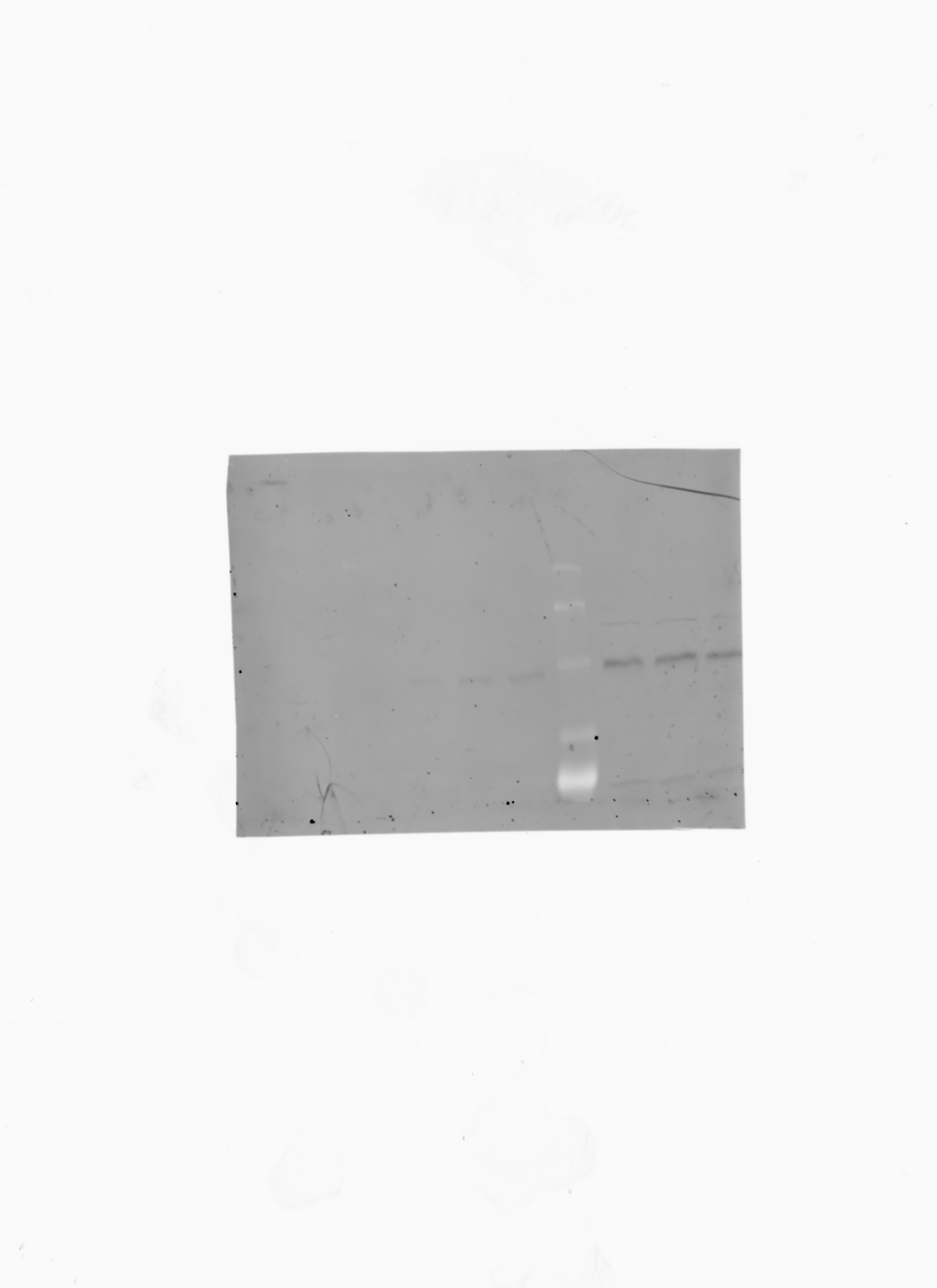

Supplement: Figure 5—source data 4. [file elife-87572-fig5-data4.zip › PolA/Rep1/AK MCM2 PolE,A Lig1 2022.07.28_12.24.23_Fl/AK MCM2 PolE,A Lig1 2022.07.28_12.24.23_Fl-Blue.tif]

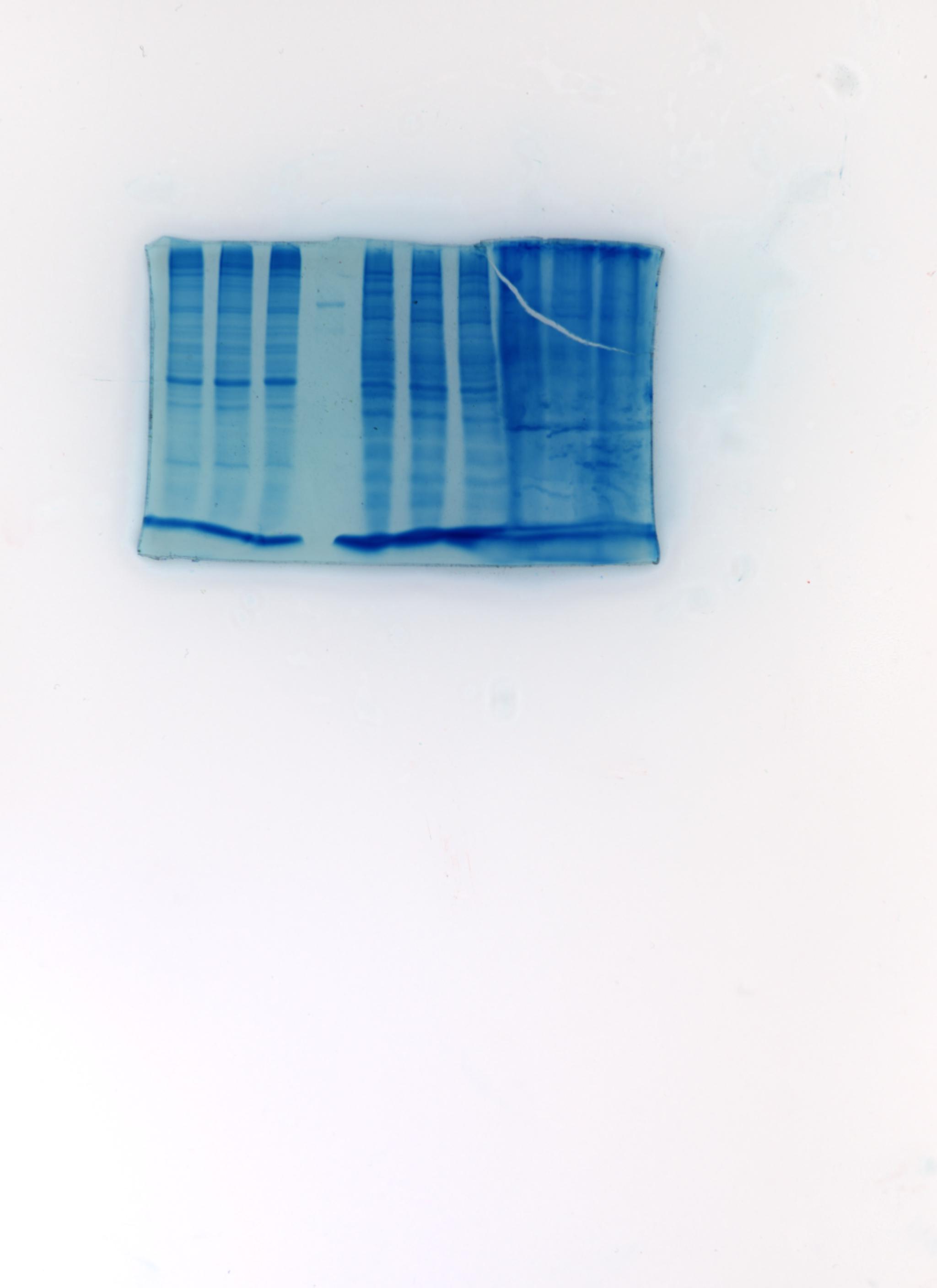

Supplement: Figure 5—source data 5. [file elife-87572-fig5-data5.zip › POLD/Rep2/Z5-C-polD 2022.12.04_15.40.56_Co/Z5-C-polD 2022.12.04_15.40.56_Co.jpg]

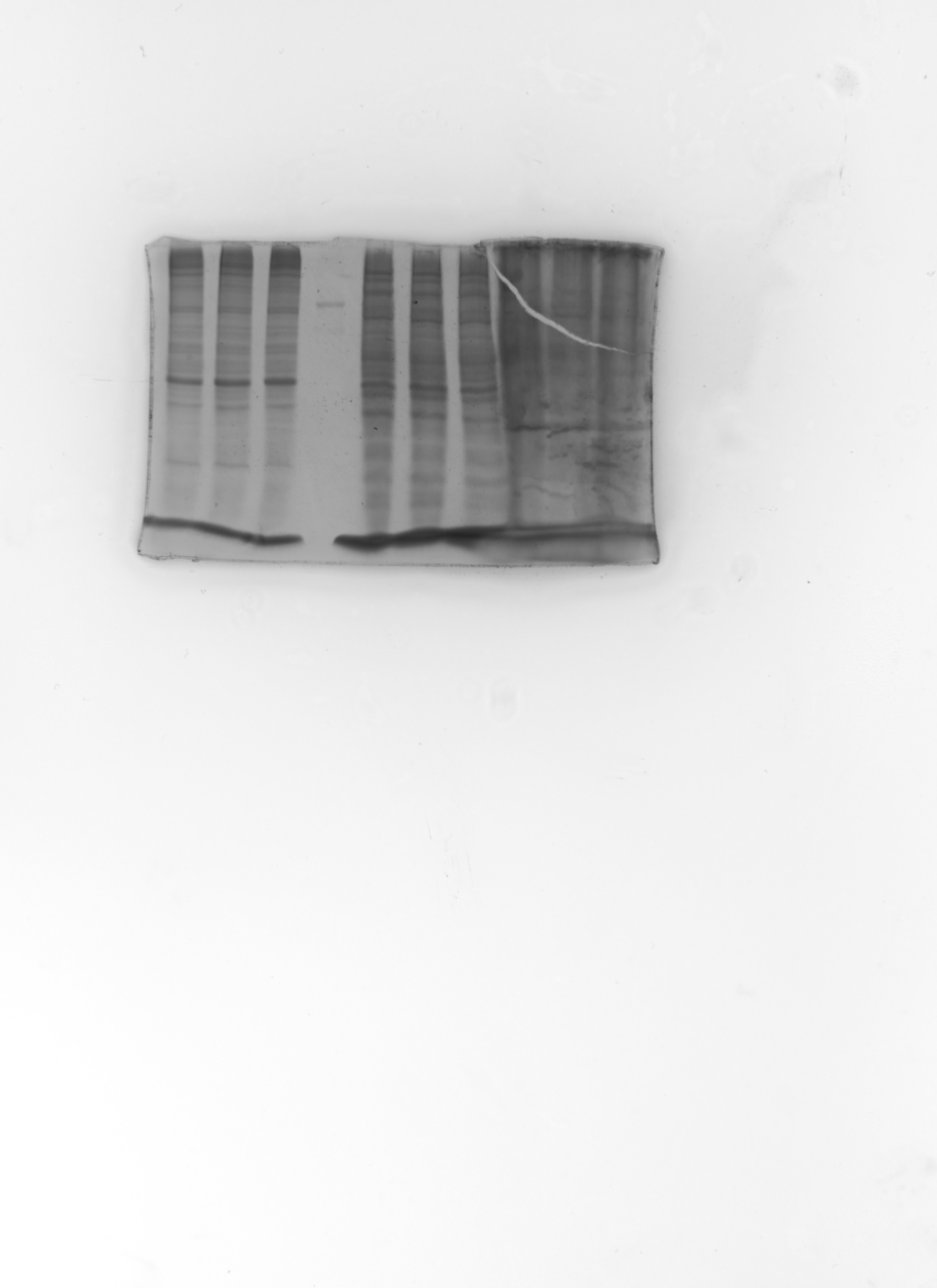

Supplement: Figure 5—source data 5. [file elife-87572-fig5-data5.zip › POLD/Rep2/Z5-C-polD 2022.12.04_15.40.56_Co/Z5-C-polD 2022.12.04_15.40.56_Co.tif]

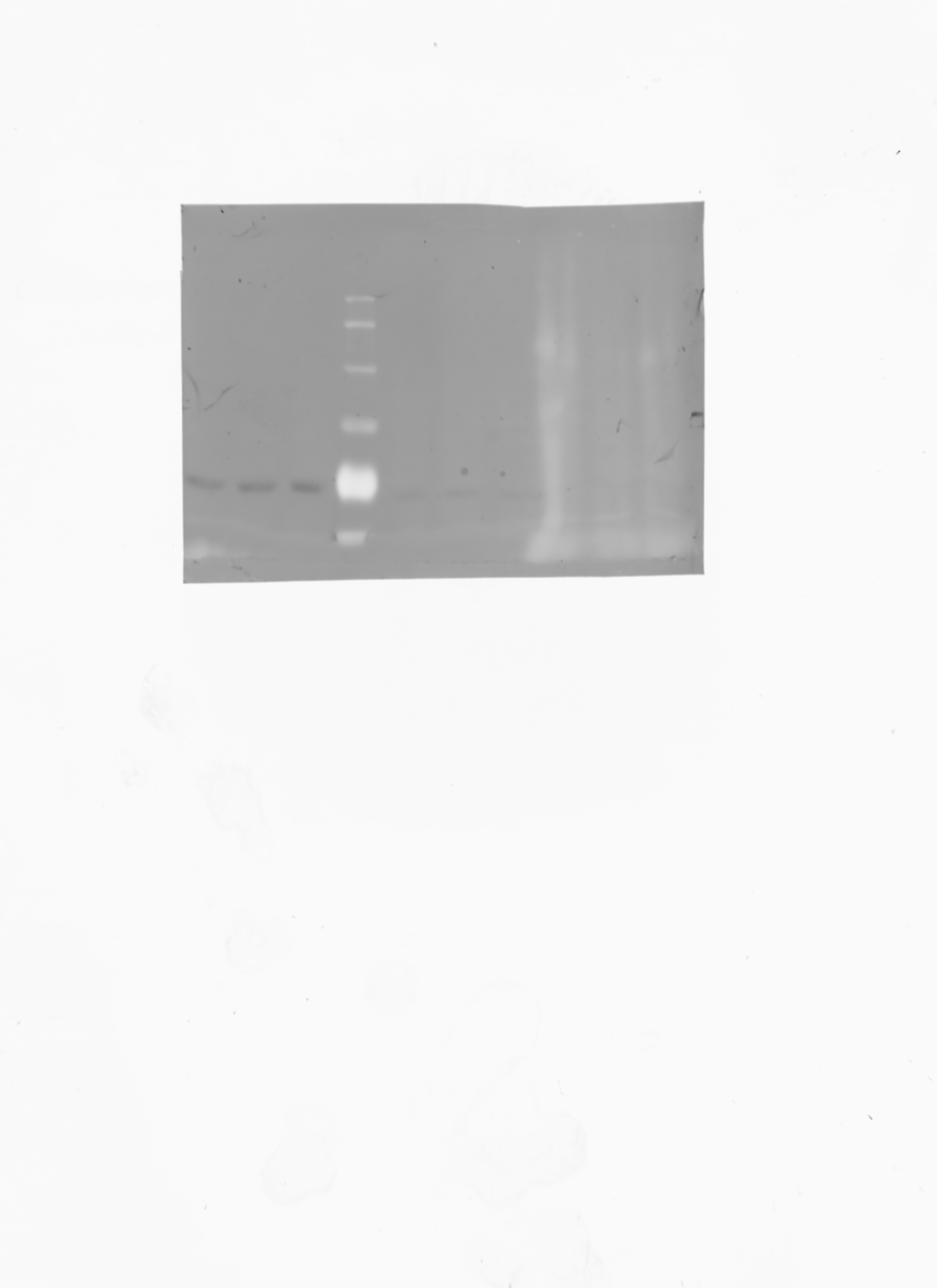

Supplement: Figure 5—source data 5. [file elife-87572-fig5-data5.zip › POLD/Rep2/polD z5 bf reprobe 2022.12.14_12.49.33_Fl/polD z5 bf reprobe 2022.12.14_12.49.33_Fl-Blue.tif]

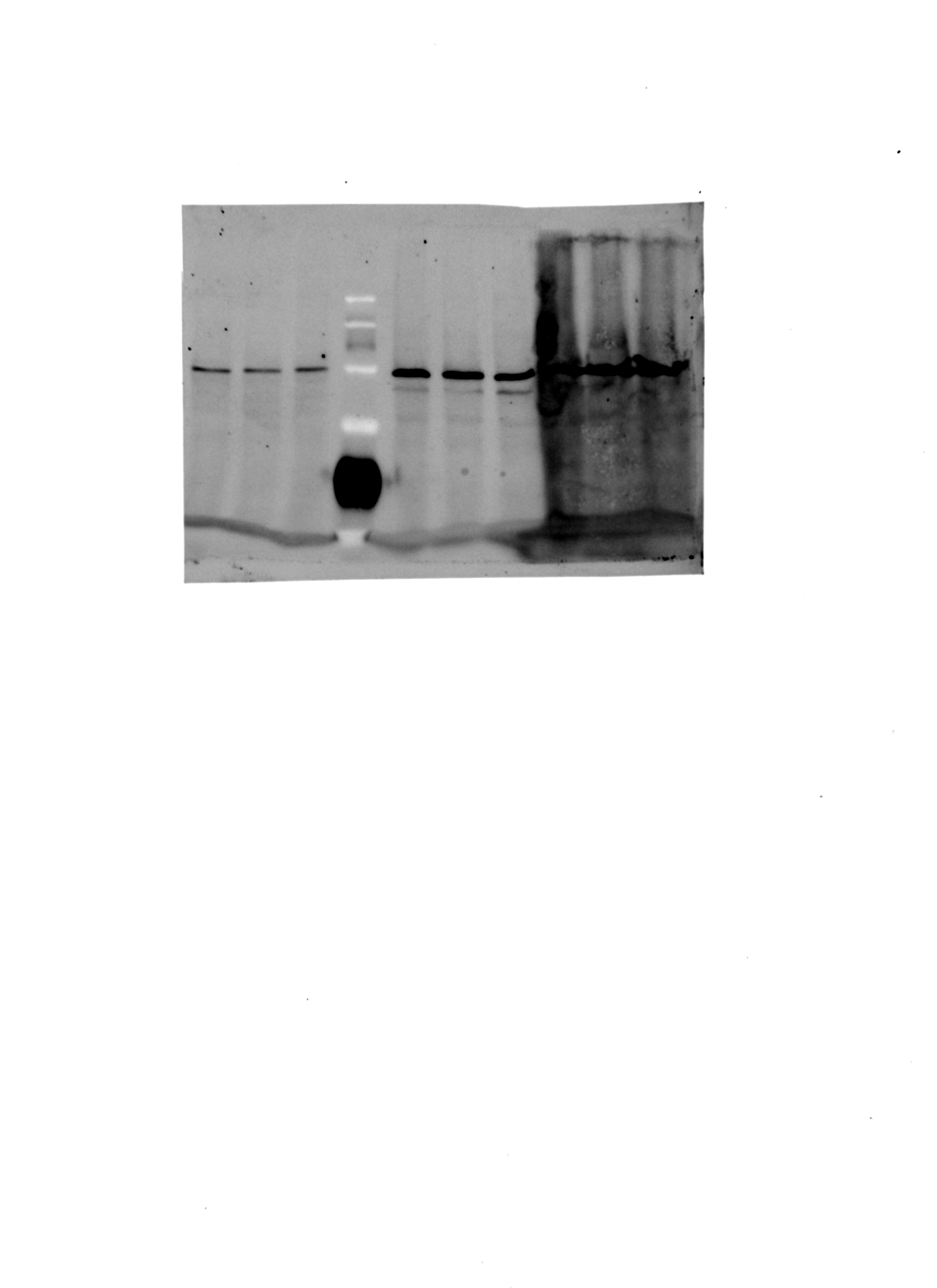

Supplement: Figure 5—source data 5. [file elife-87572-fig5-data5.zip › POLD/Rep2/polD z5 bf reprobe 2022.12.14_12.49.33_Fl/polD z5 bf reprobe 2022.12.14_12.49.33_Fl-Green.tif]

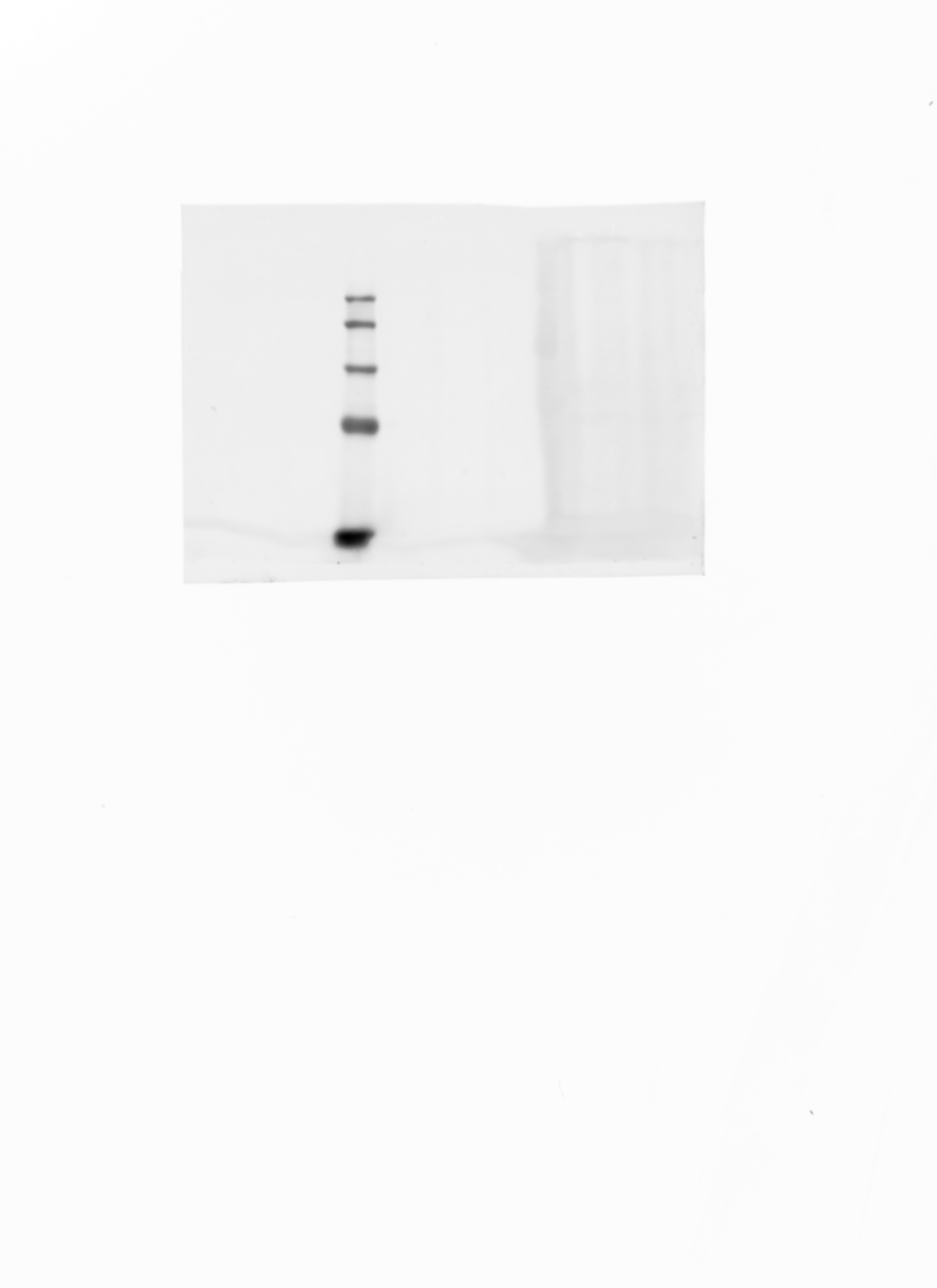

Supplement: Figure 5—source data 5. [file elife-87572-fig5-data5.zip › POLD/Rep2/polD z5 bf reprobe 2022.12.14_12.49.33_Fl/polD z5 bf reprobe 2022.12.14_12.49.33_Fl-Red.tif]

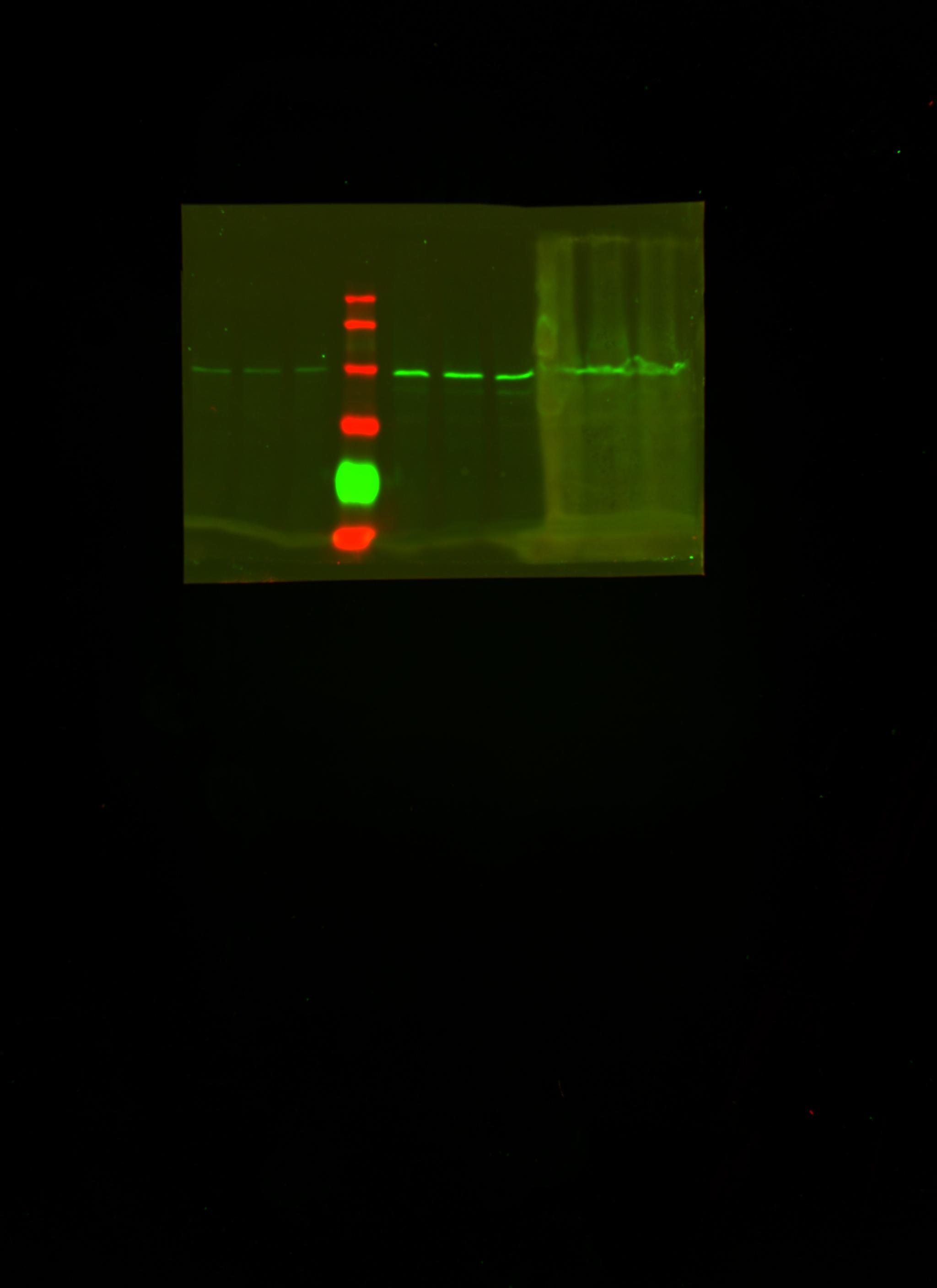

Supplement: Figure 5—source data 5. [file elife-87572-fig5-data5.zip › POLD/Rep2/polD z5 bf reprobe 2022.12.14_12.49.33_Fl/polD z5 bf reprobe 2022.12.14_12.49.33_Fl.jpg]

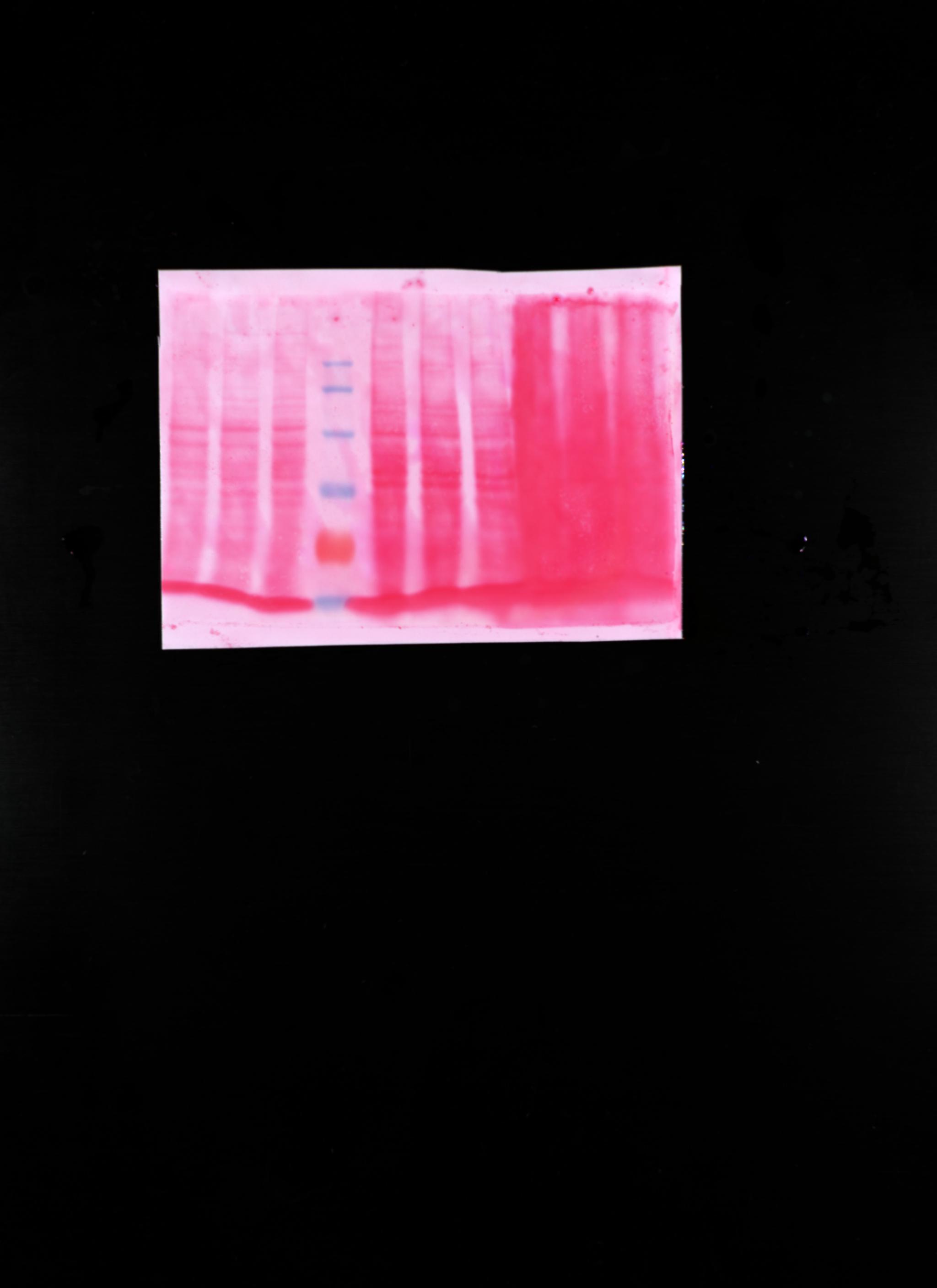

Supplement: Figure 5—source data 5. [file elife-87572-fig5-data5.zip › POLD/Rep2/Z5-P-PolD 2022.11.29_16.28.37_Co/Z5-P-PolD 2022.11.29_16.28.37_Co.jpg]

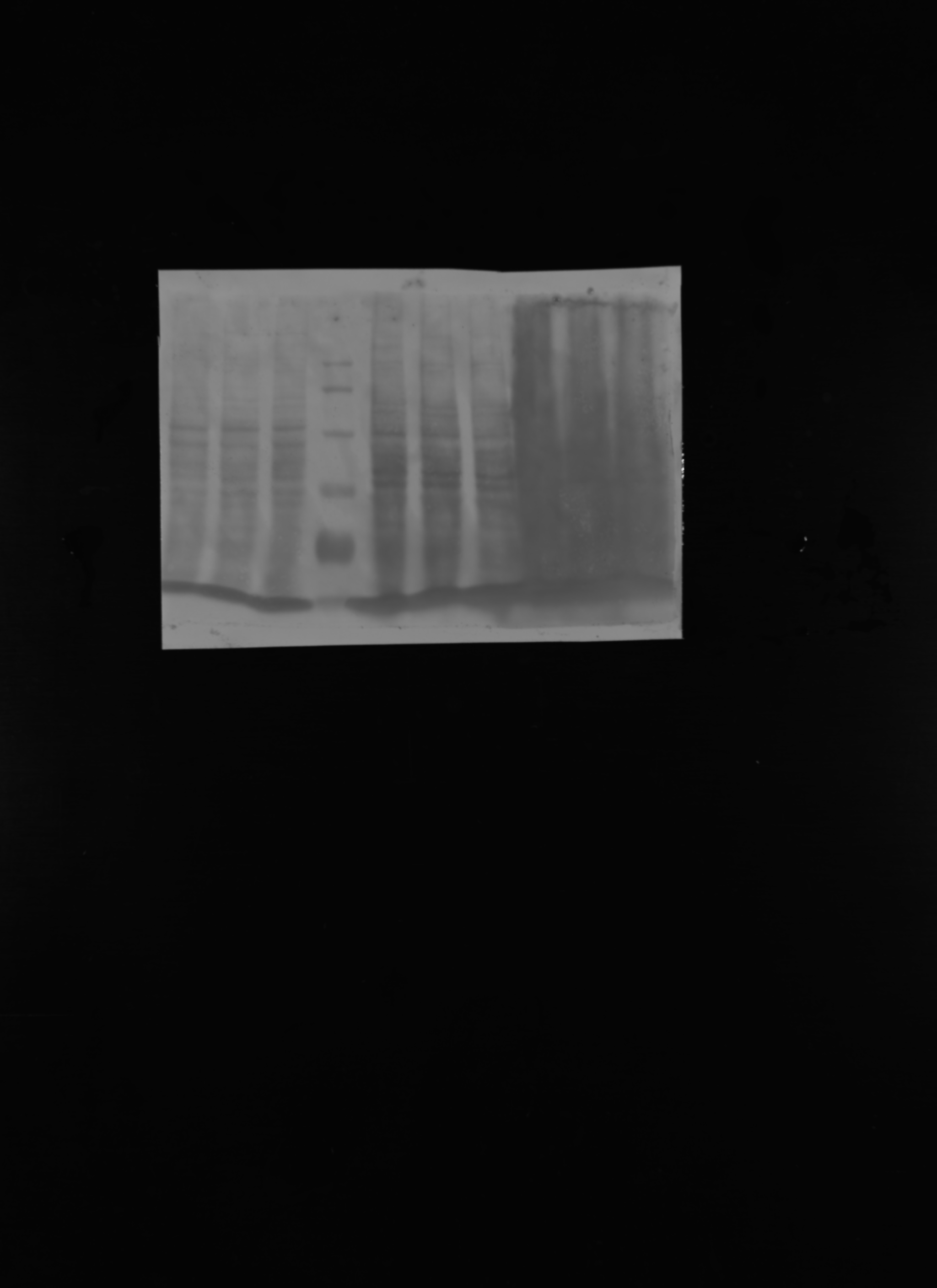

Supplement: Figure 5—source data 5. [file elife-87572-fig5-data5.zip › POLD/Rep2/Z5-P-PolD 2022.11.29_16.28.37_Co/Z5-P-PolD 2022.11.29_16.28.37_Co.tif]

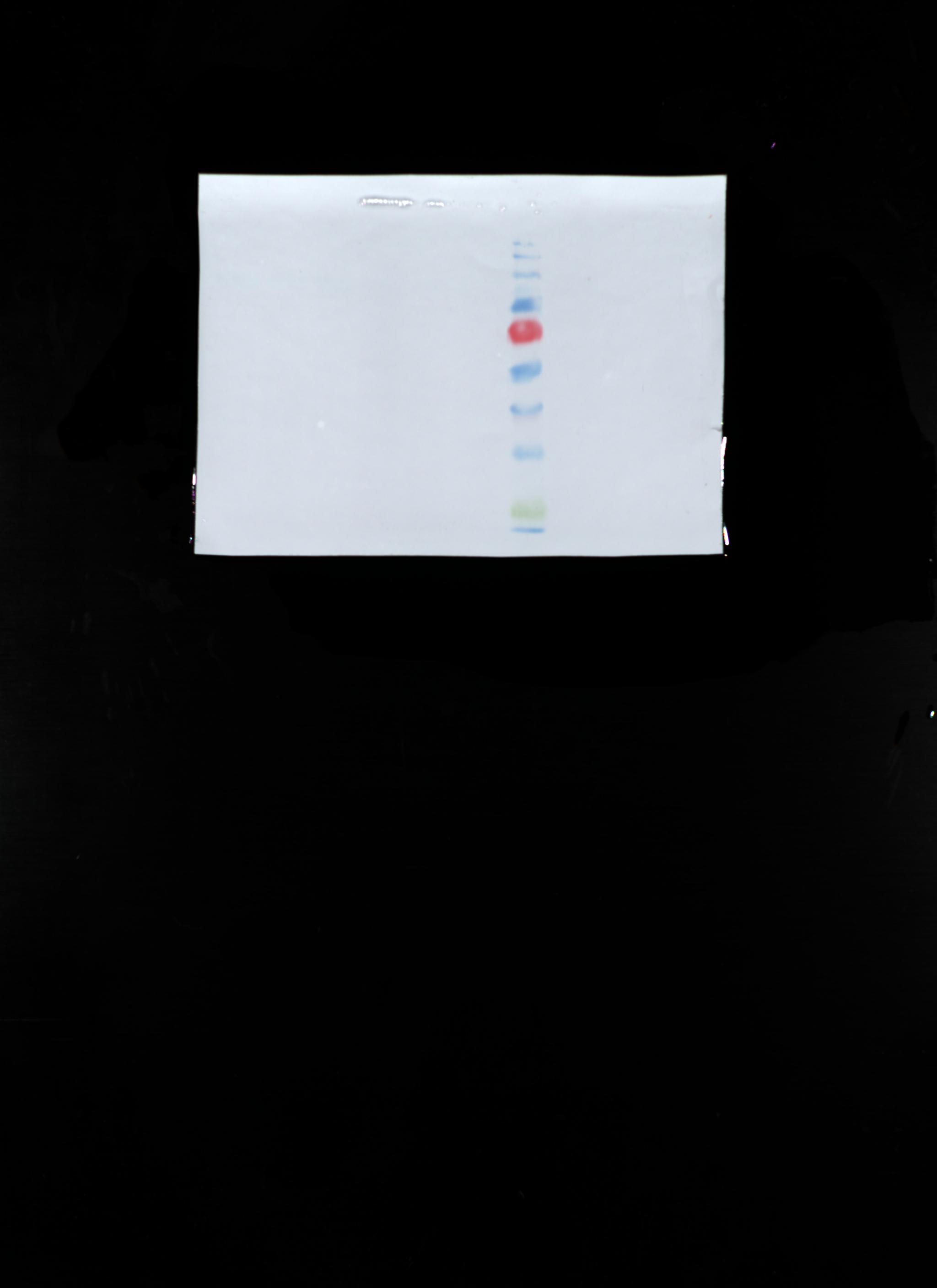

Supplement: Figure 5—source data 5. [file elife-87572-fig5-data5.zip › POLD/Rep1/G4-polDcy3-8s 2022.11.19_17.00.32_Fl-Green/G4-polDcy3-8s 2022.11.19_17.00.32_Fl-Green-Marker.jpg]

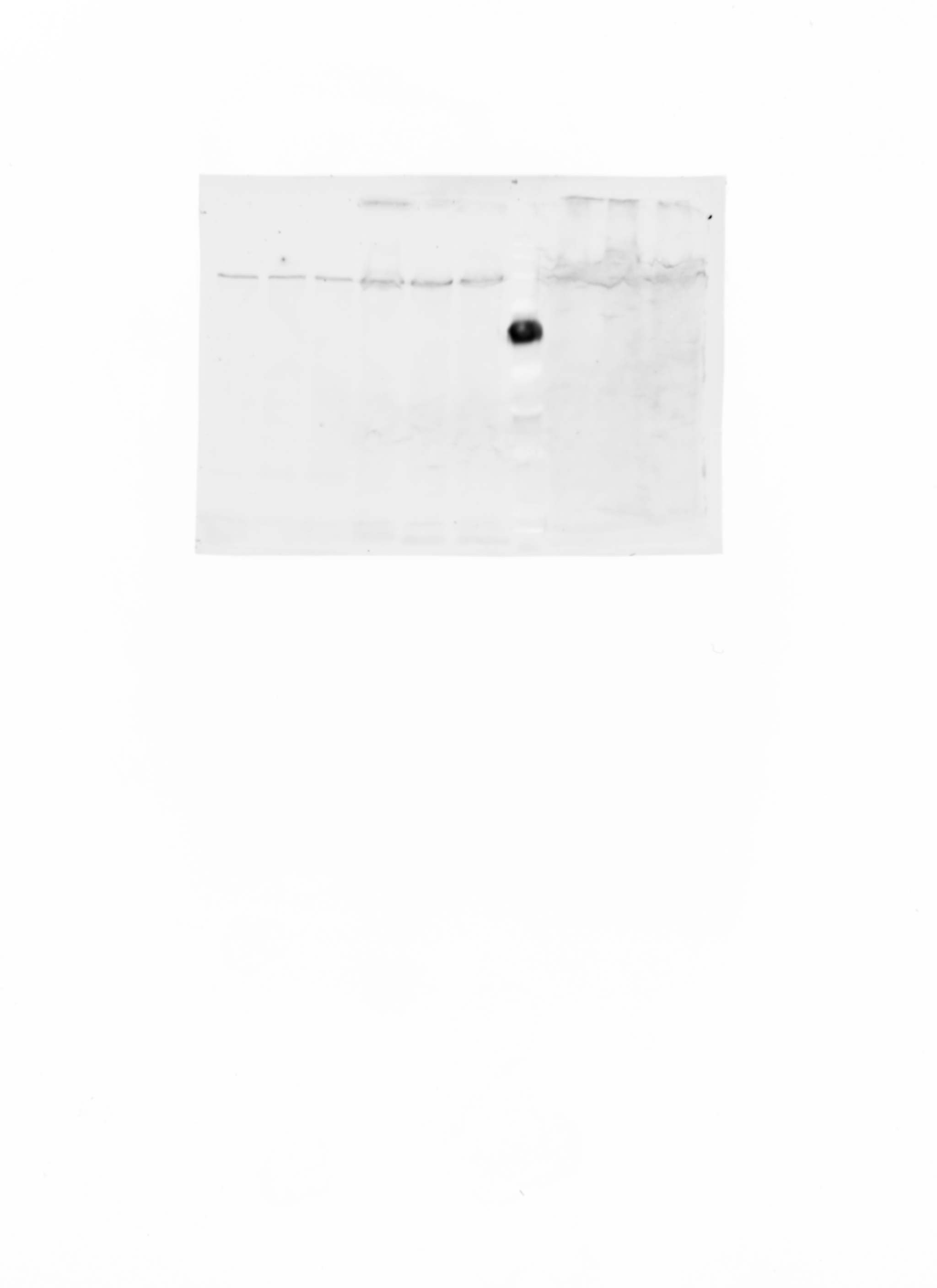

Supplement: Figure 5—source data 5. [file elife-87572-fig5-data5.zip › POLD/Rep1/G4-polDcy3-8s 2022.11.19_17.00.32_Fl-Green/G4-polDcy3-8s 2022.11.19_17.00.32_Fl-Green.tif]

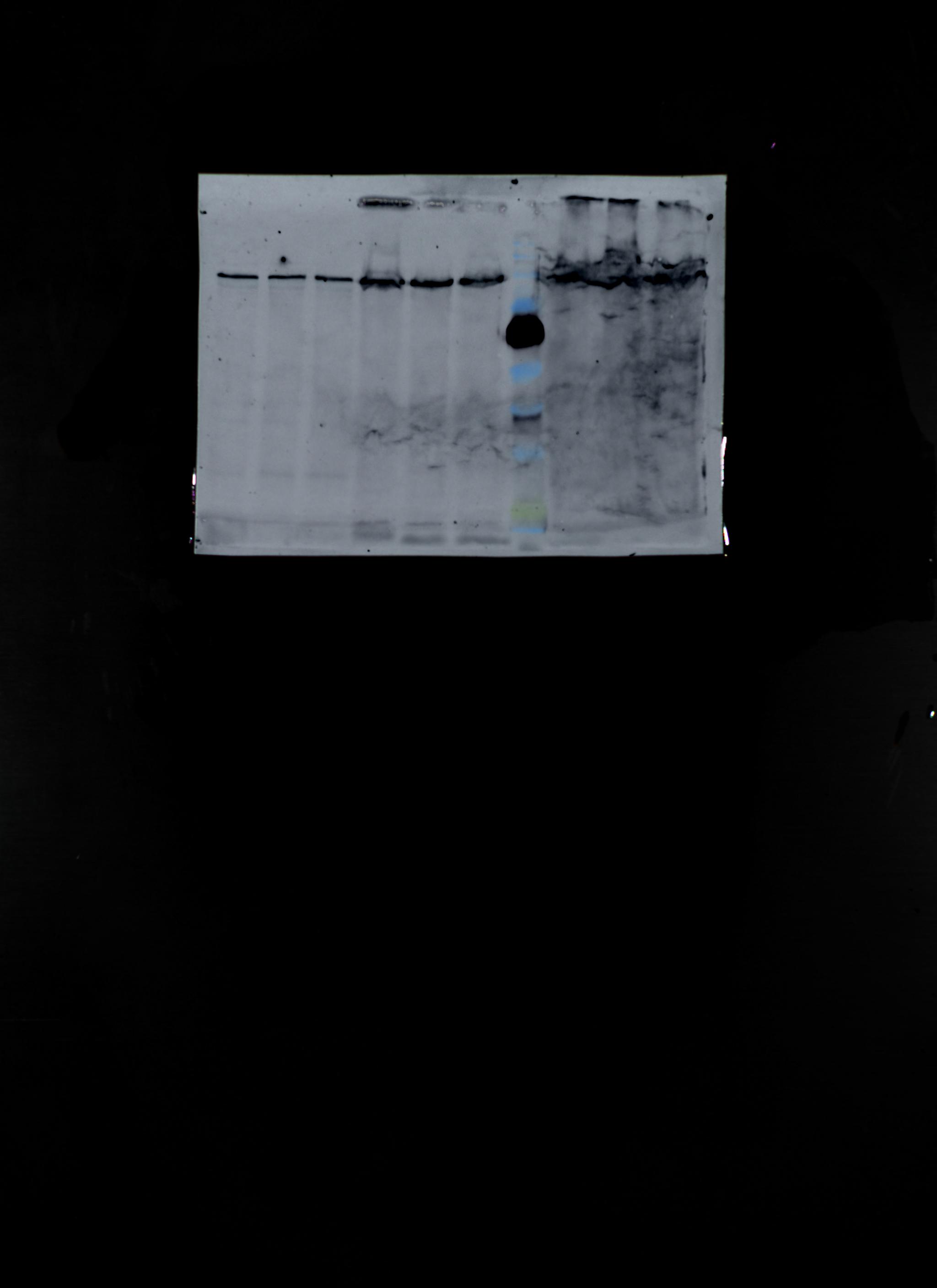

Supplement: Figure 5—source data 5. [file elife-87572-fig5-data5.zip › POLD/Rep1/G4-polDcy3-8s 2022.11.19_17.00.32_Fl-Green/G4-polDcy3-8s 2022.11.19_17.00.32_Fl-Green+Marker.jpg]

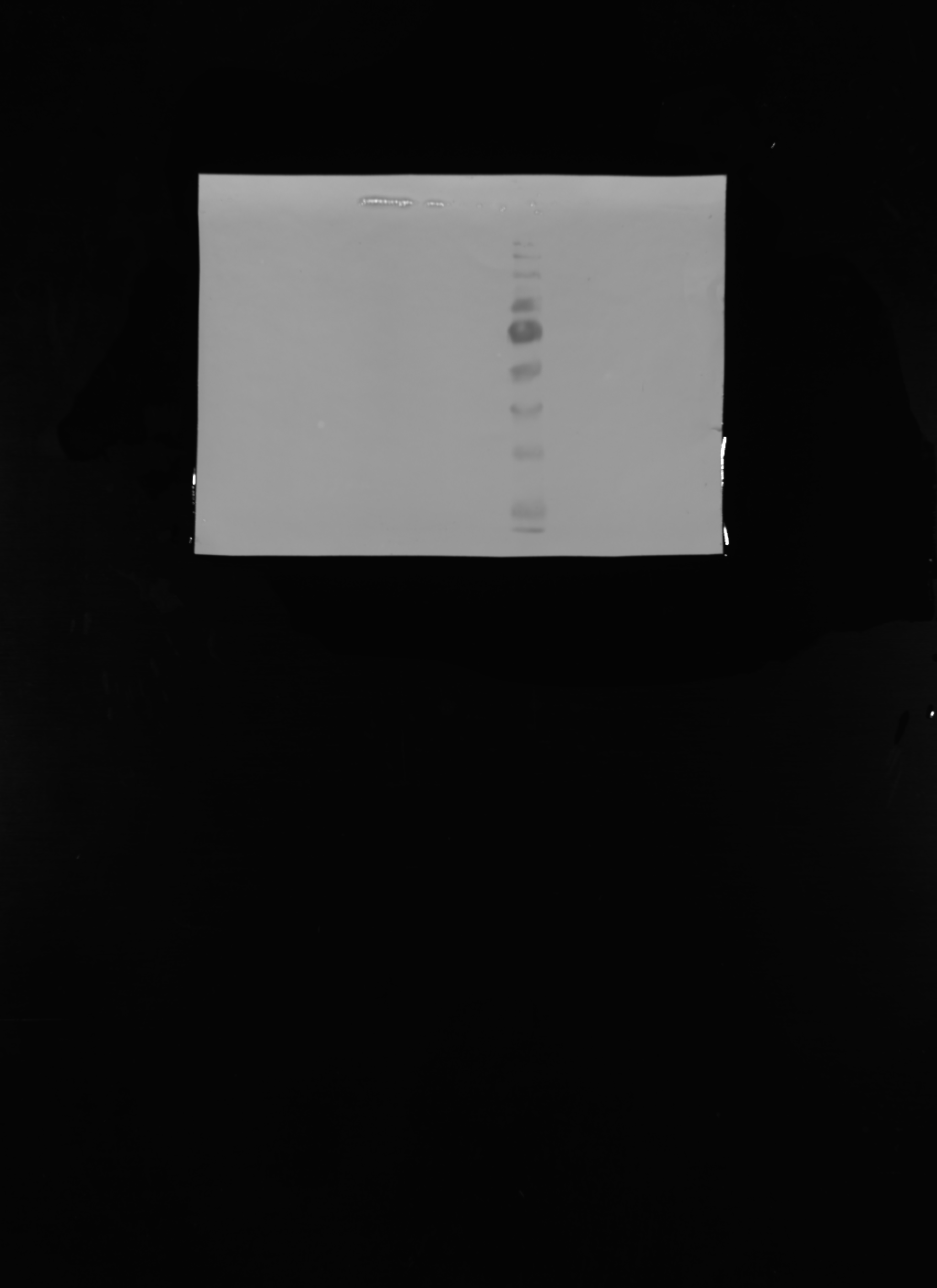

Supplement: Figure 5—source data 5. [file elife-87572-fig5-data5.zip › POLD/Rep1/G4-polDcy3-8s 2022.11.19_17.00.32_Fl-Green/G4-polDcy3-8s 2022.11.19_17.00.32_Fl-Green-Marker.tif]

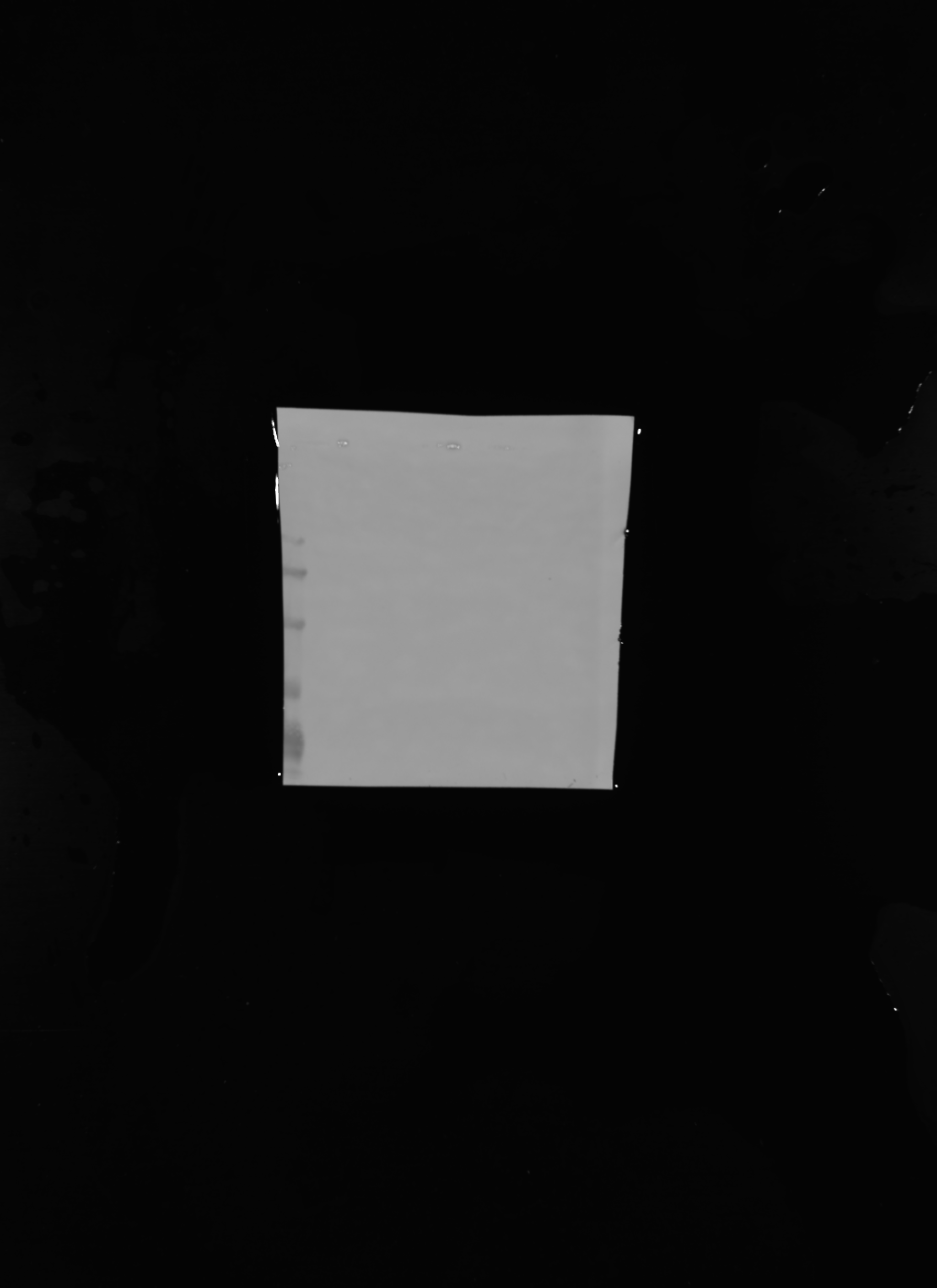

Supplement: Figure 5—source data 6. [file elife-87572-fig5-data6.zip › POLE/Rep2/6%G2-polE-120s 2022.08.17_14.57.22_Fl-Red/6%G2-polE-120s 2022.08.17_14.57.22_Fl-Red-Marker.tif]

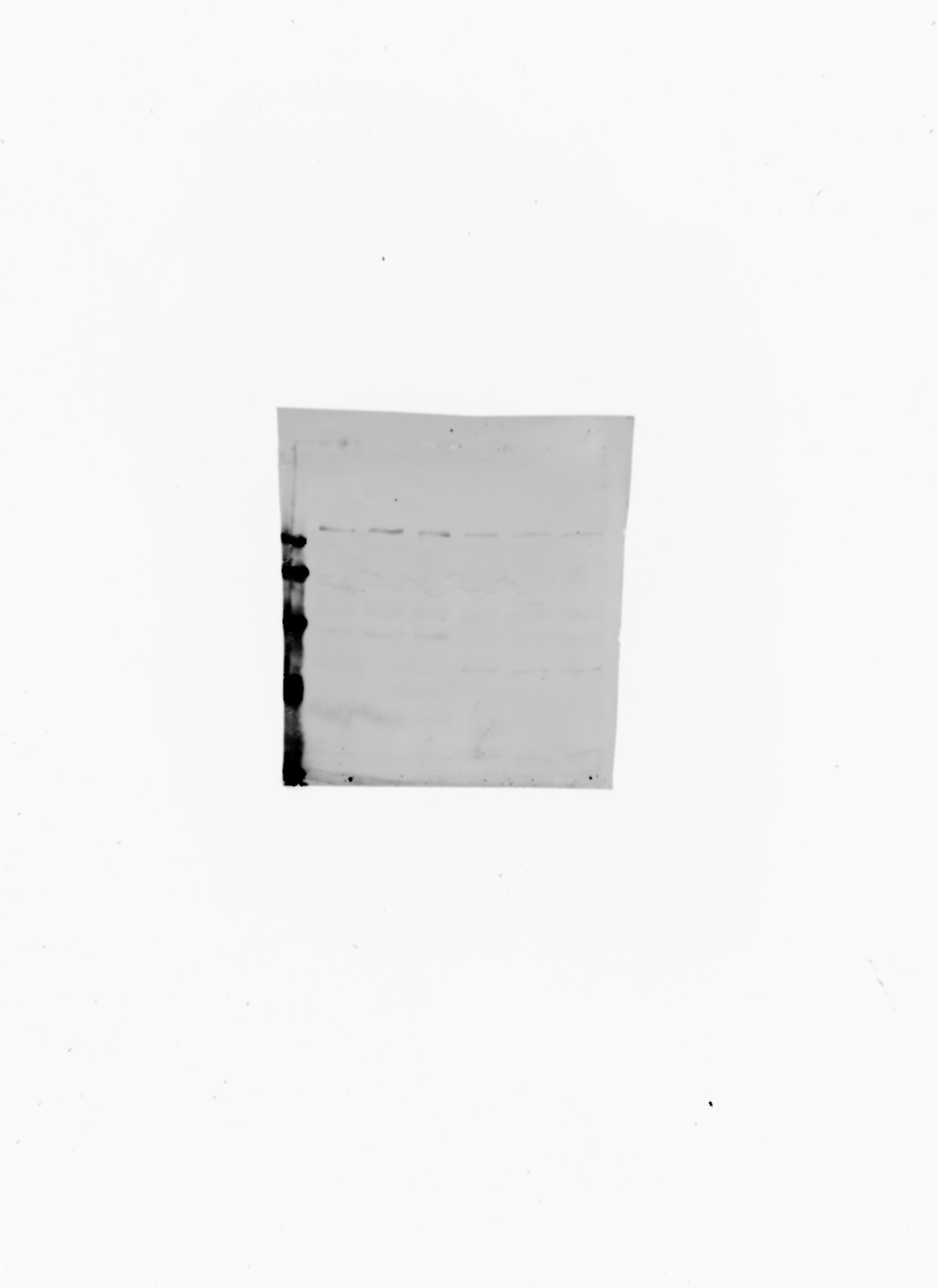

Supplement: Figure 5—source data 6. [file elife-87572-fig5-data6.zip › POLE/Rep2/6%G2-polE-120s 2022.08.17_14.57.22_Fl-Red/6%G2-polE-120s 2022.08.17_14.57.22_Fl-Red.tif]

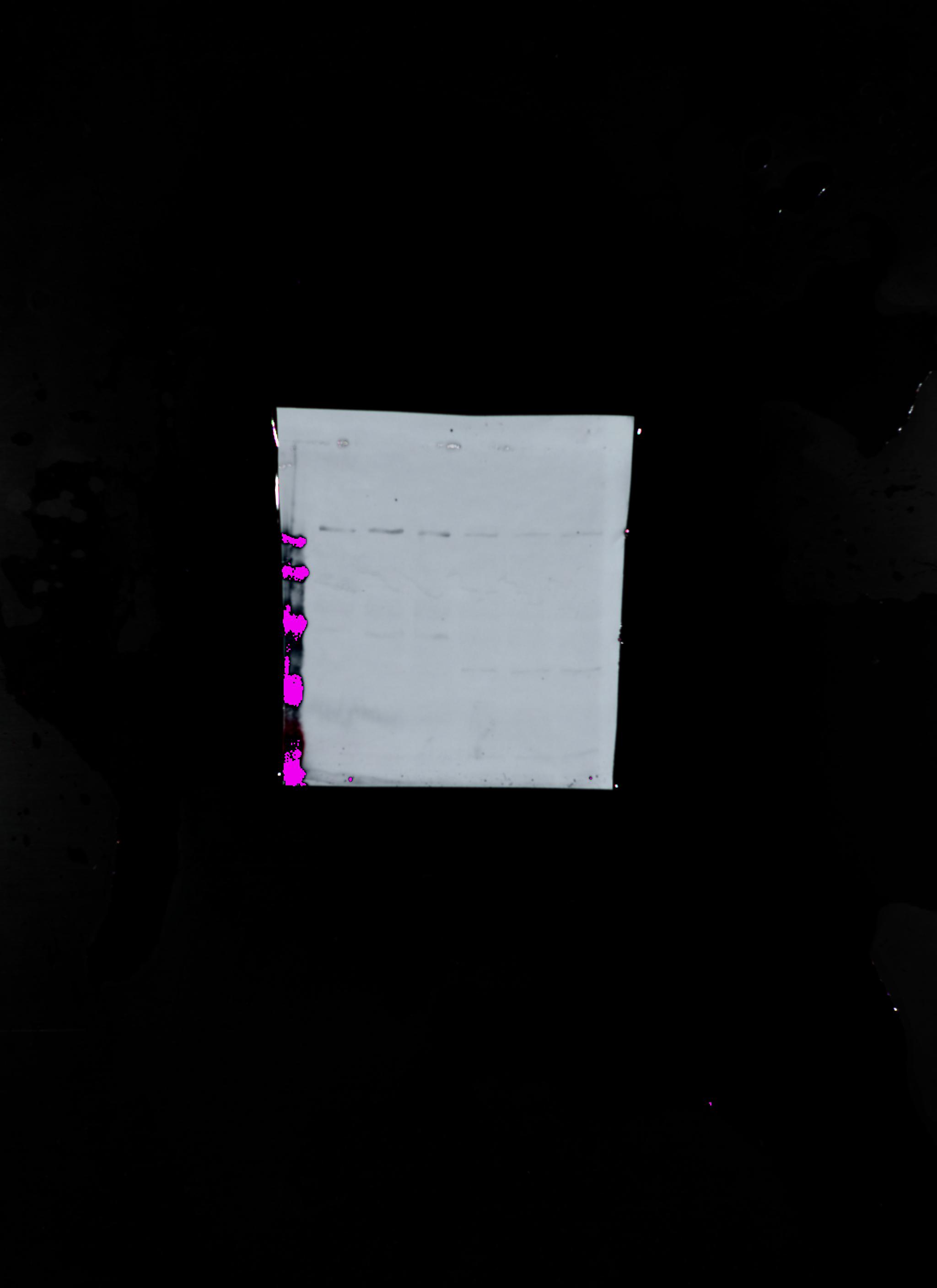

Supplement: Figure 5—source data 6. [file elife-87572-fig5-data6.zip › POLE/Rep2/6%G2-polE-120s 2022.08.17_14.57.22_Fl-Red/6%G2-polE-120s 2022.08.17_14.57.22_Fl-Red+Marker.jpg]

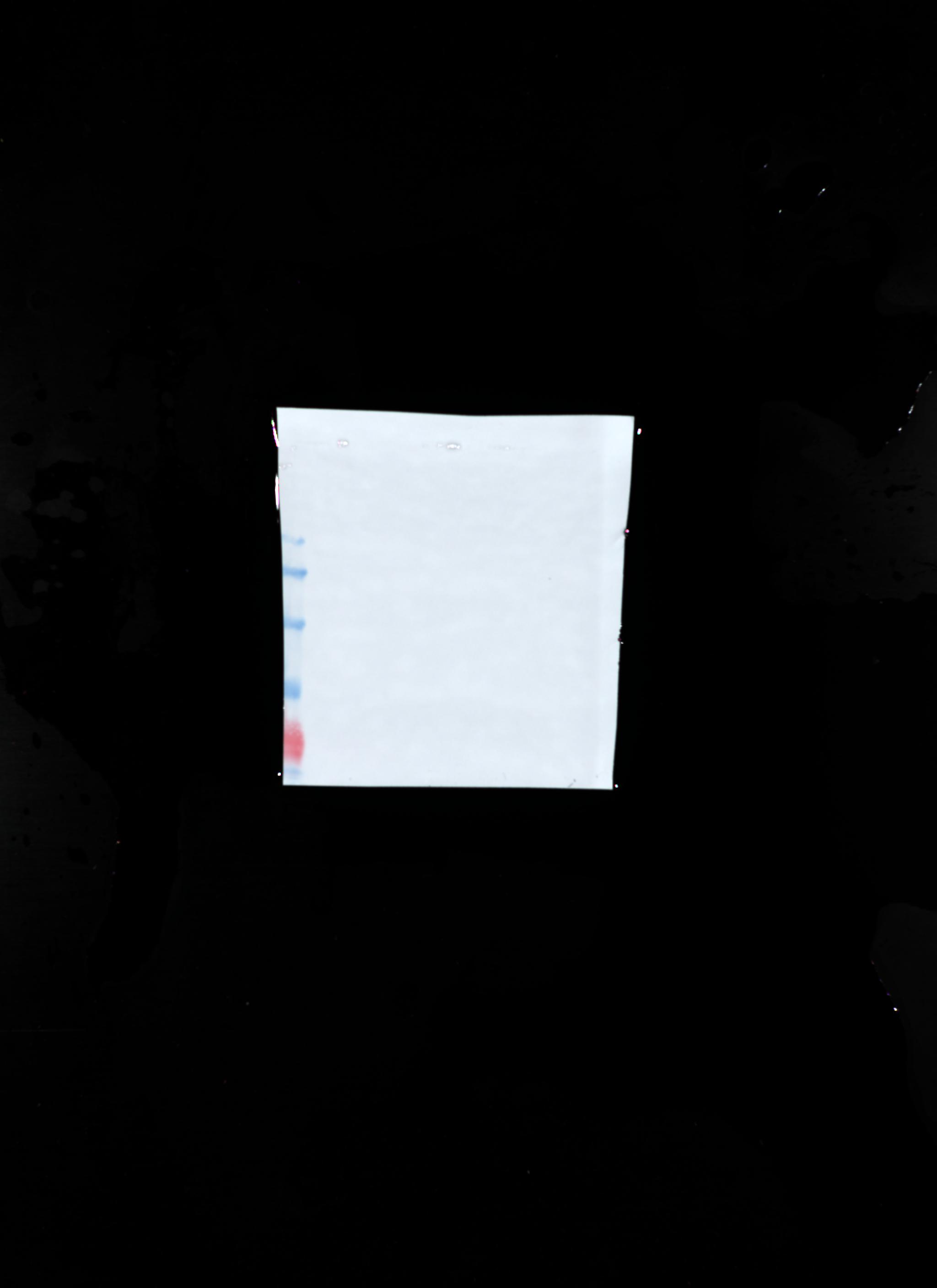

Supplement: Figure 5—source data 6. [file elife-87572-fig5-data6.zip › POLE/Rep2/6%G2-polE-120s 2022.08.17_14.57.22_Fl-Red/6%G2-polE-120s 2022.08.17_14.57.22_Fl-Red-Marker.jpg]

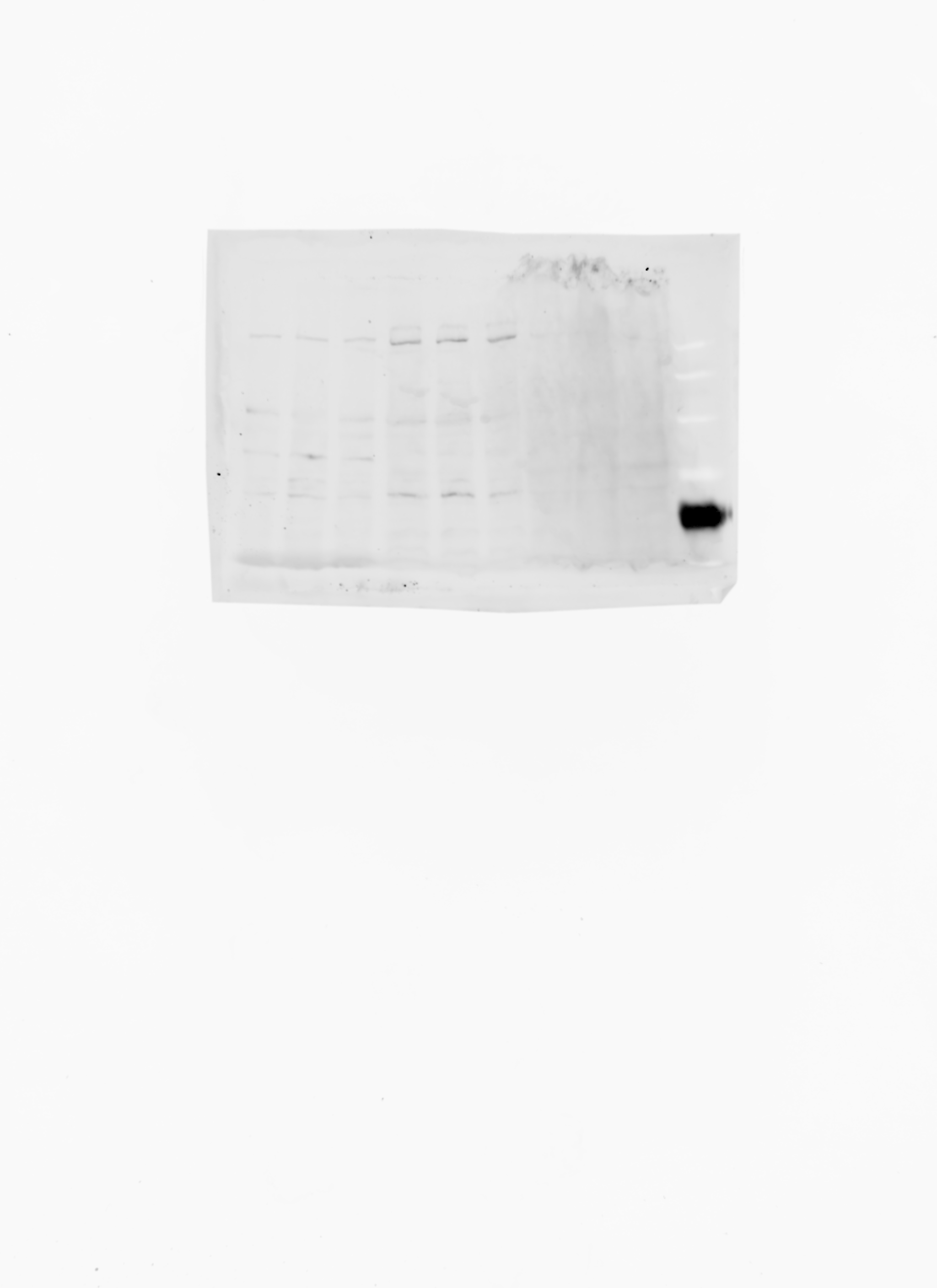

Supplement: Figure 5—source data 6. [file elife-87572-fig5-data6.zip › POLE/Rep3/polE-6%-Hu-Q1 2023.01.10_15.25.42_Fl-Green/polE-6%-Hu-Q1 2023.01.10_15.25.42_Fl-Green.tif]

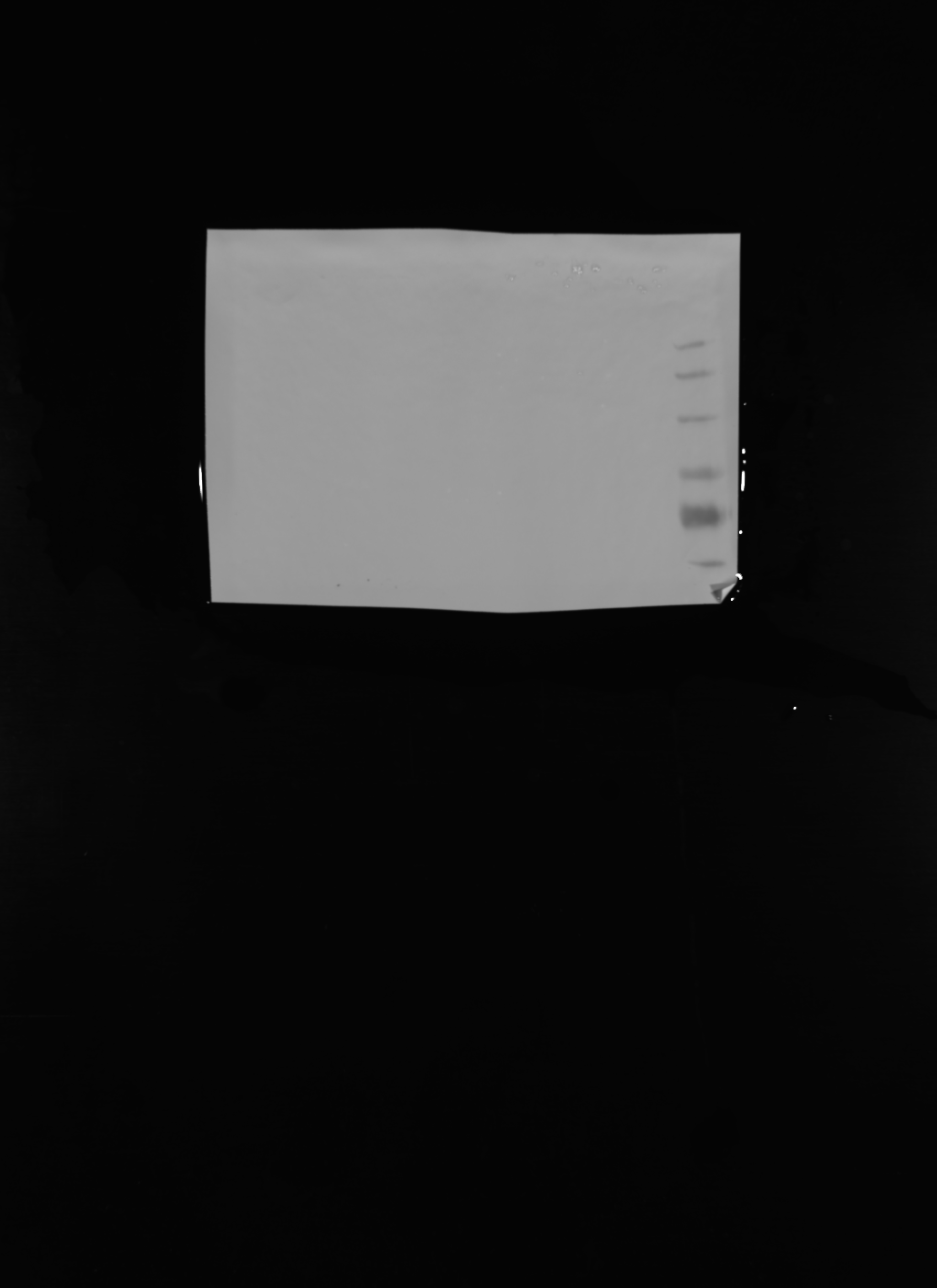

Supplement: Figure 5—source data 6. [file elife-87572-fig5-data6.zip › POLE/Rep3/polE-6%-Hu-Q1 2023.01.10_15.25.42_Fl-Green/polE-6%-Hu-Q1 2023.01.10_15.25.42_Fl-Green-Marker.tif]

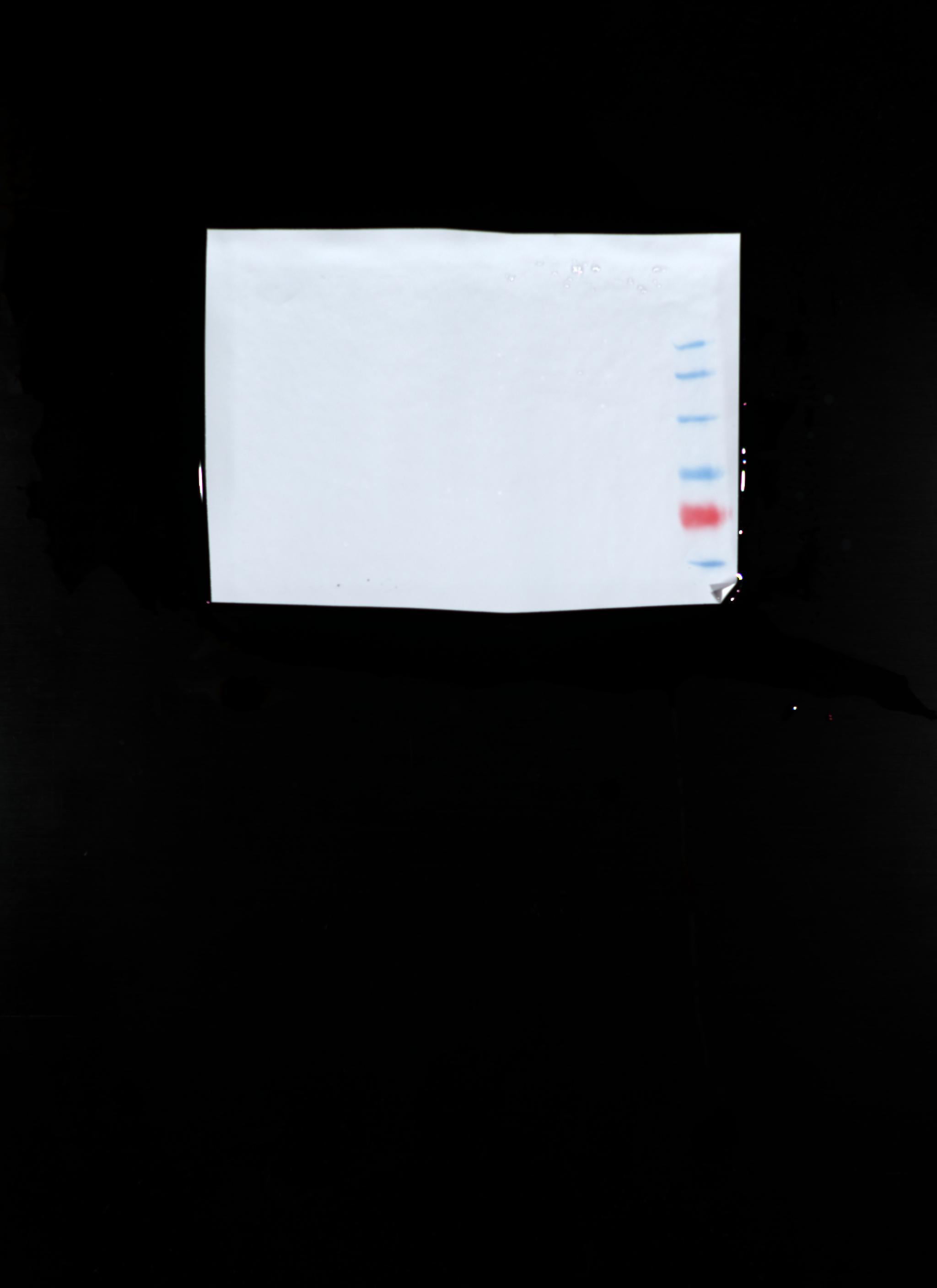

Supplement: Figure 5—source data 6. [file elife-87572-fig5-data6.zip › POLE/Rep3/polE-6%-Hu-Q1 2023.01.10_15.25.42_Fl-Green/polE-6%-Hu-Q1 2023.01.10_15.25.42_Fl-Green-Marker.jpg]

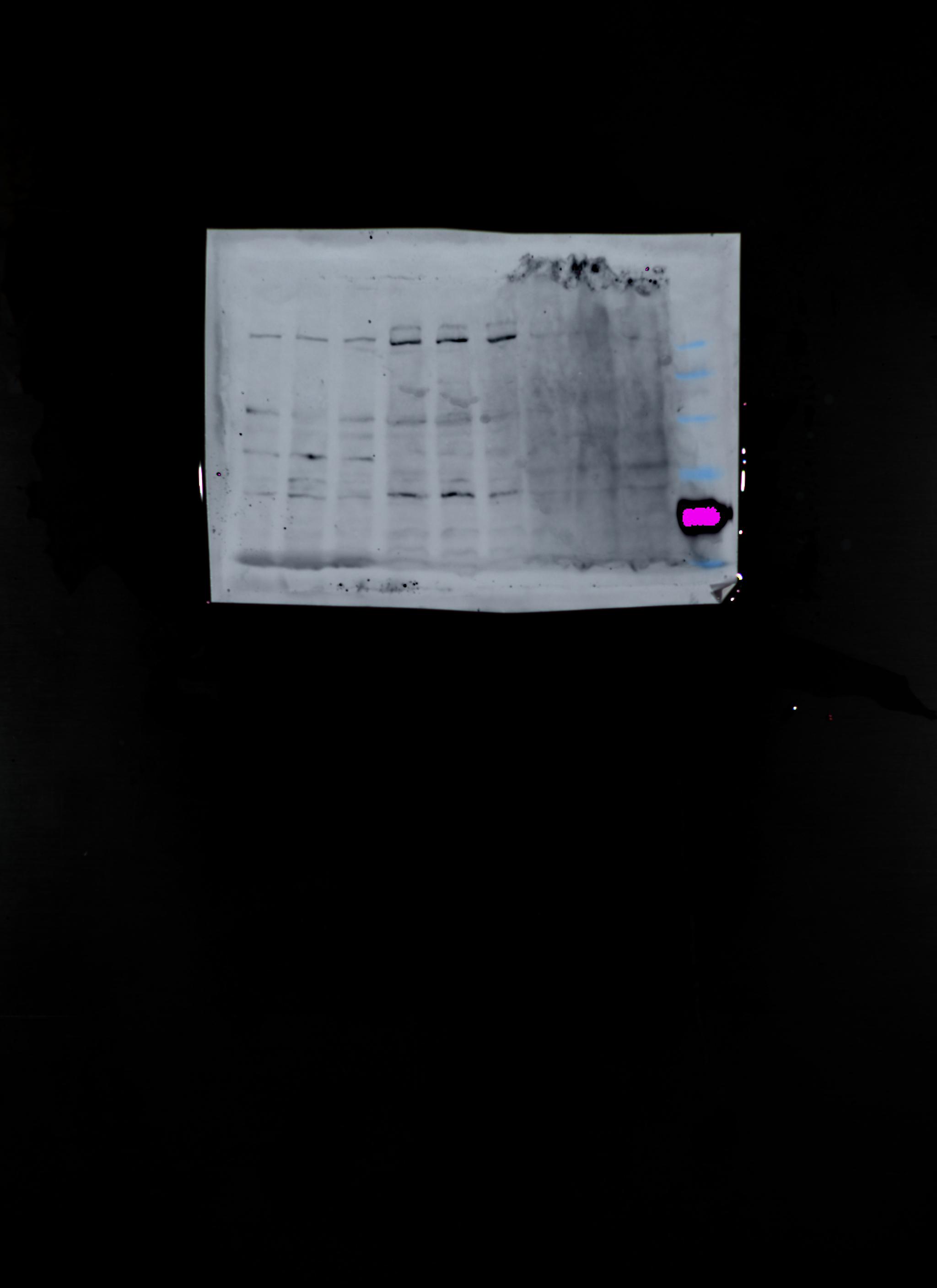

Supplement: Figure 5—source data 6. [file elife-87572-fig5-data6.zip › POLE/Rep3/polE-6%-Hu-Q1 2023.01.10_15.25.42_Fl-Green/polE-6%-Hu-Q1 2023.01.10_15.25.42_Fl-Green+Marker.jpg]

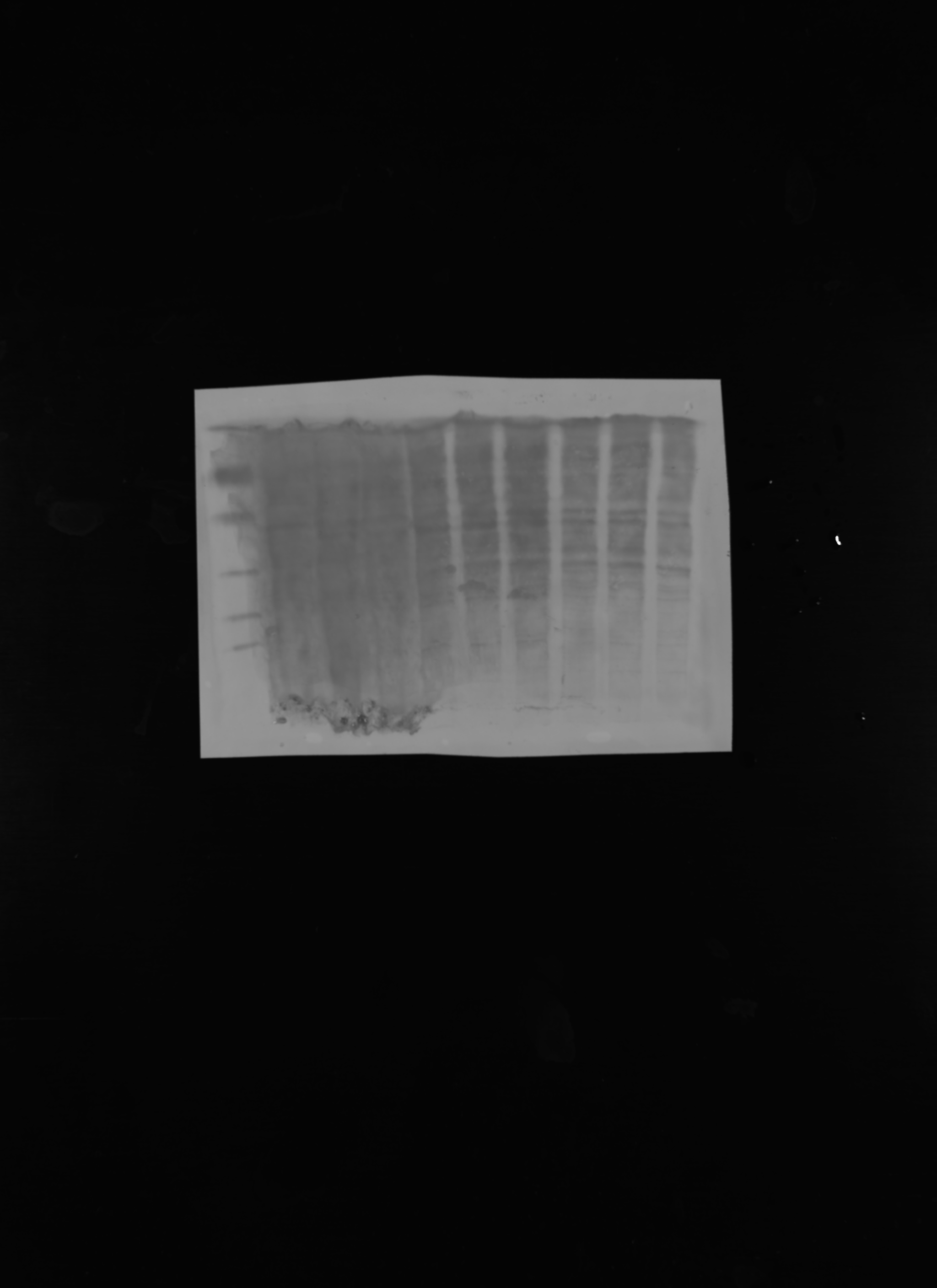

Supplement: Figure 5—source data 6. [file elife-87572-fig5-data6.zip › POLE/Rep3/P-6%-Hu-Q1 2023.01.09_17.51.52_Co/P-6%-Hu-Q1 2023.01.09_17.51.52_Co.tif]

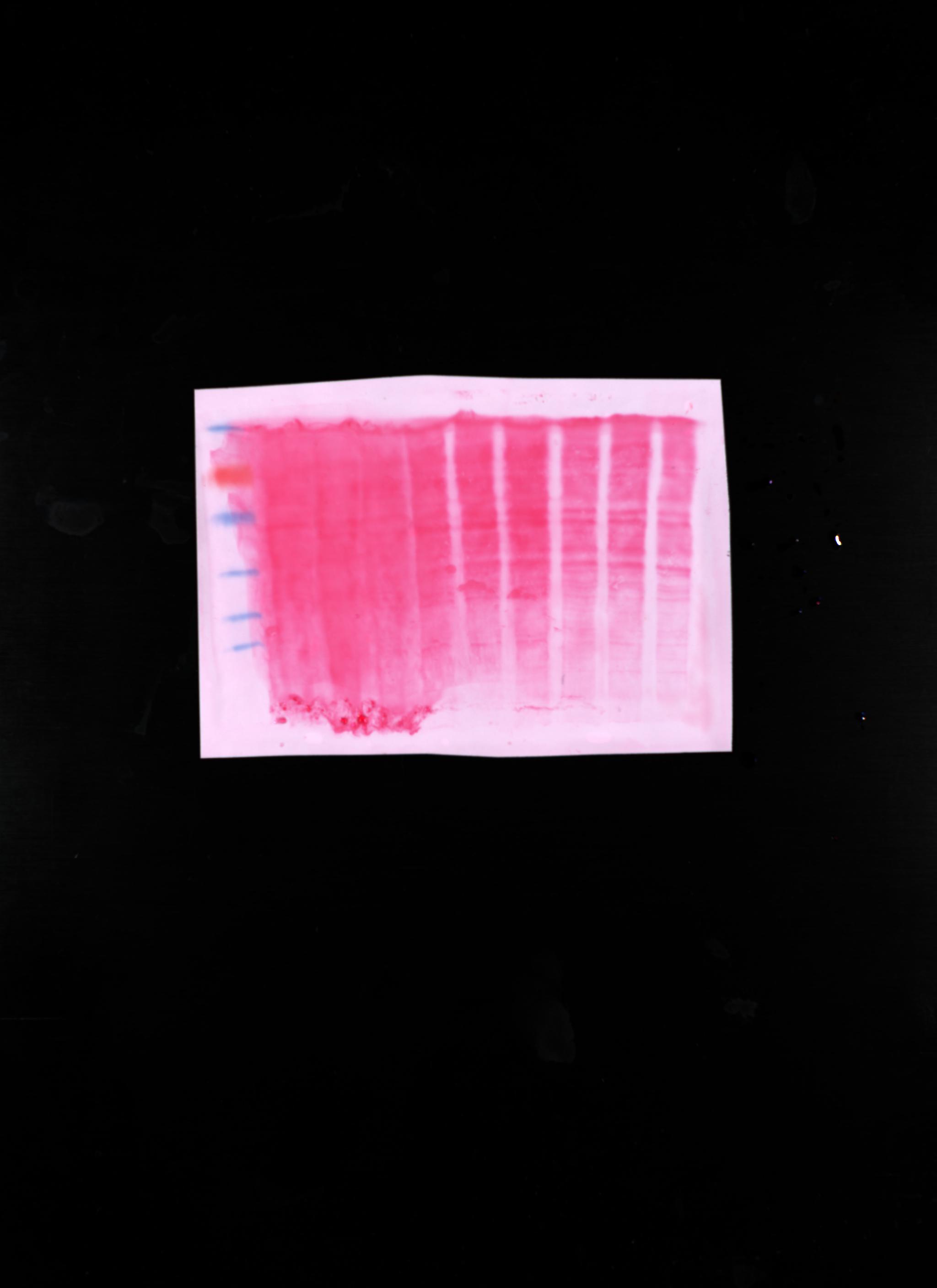

Supplement: Figure 5—source data 6. [file elife-87572-fig5-data6.zip › POLE/Rep3/P-6%-Hu-Q1 2023.01.09_17.51.52_Co/P-6%-Hu-Q1 2023.01.09_17.51.52_Co.jpg]

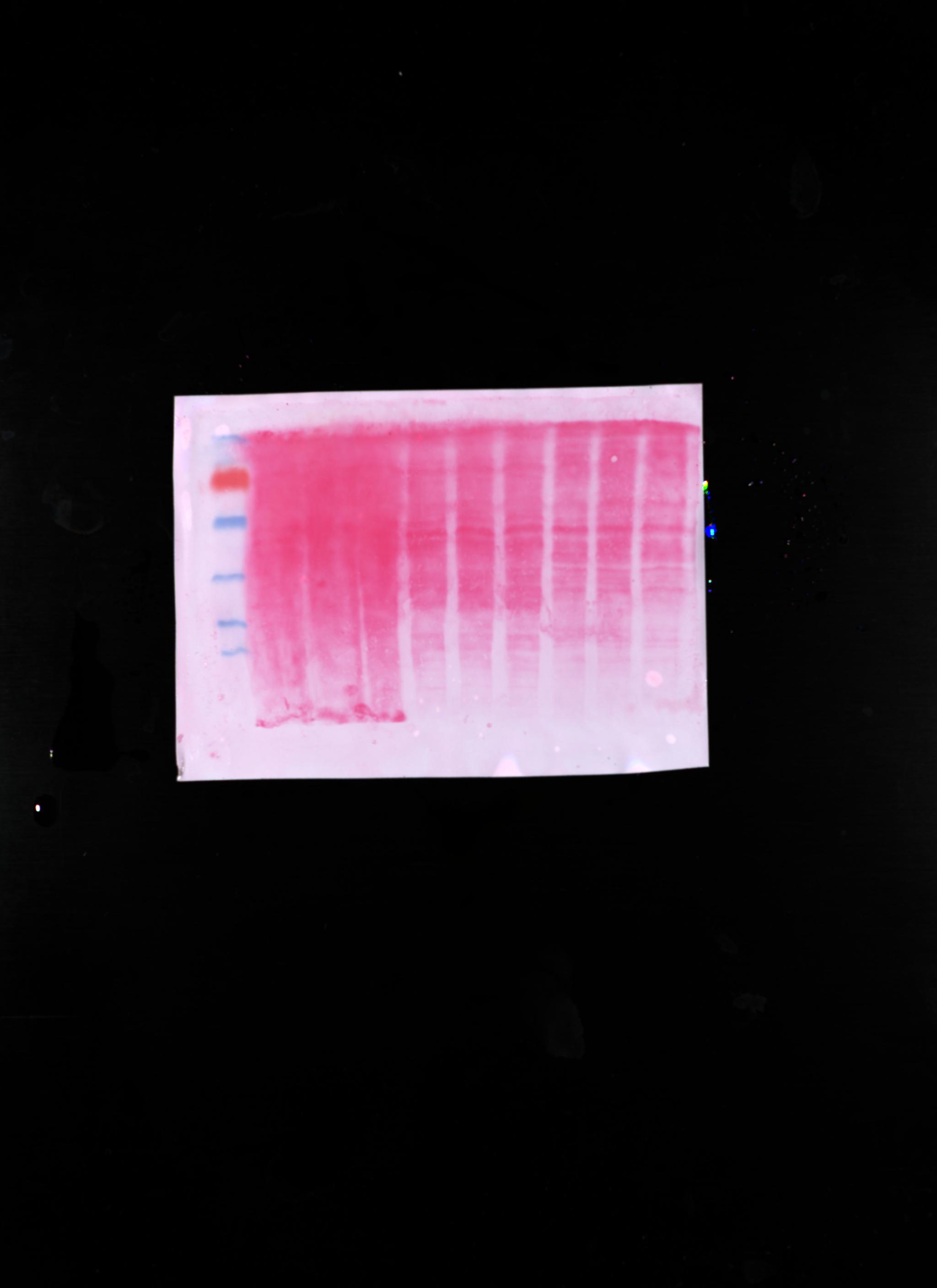

Supplement: Figure 5—source data 6. [file elife-87572-fig5-data6.zip › POLE/Rep1/P-6%-Mu-Q2 2023.01.09_17.56.57_Co/P-6%-Mu-Q2 2023.01.09_17.56.57_Co.jpg]

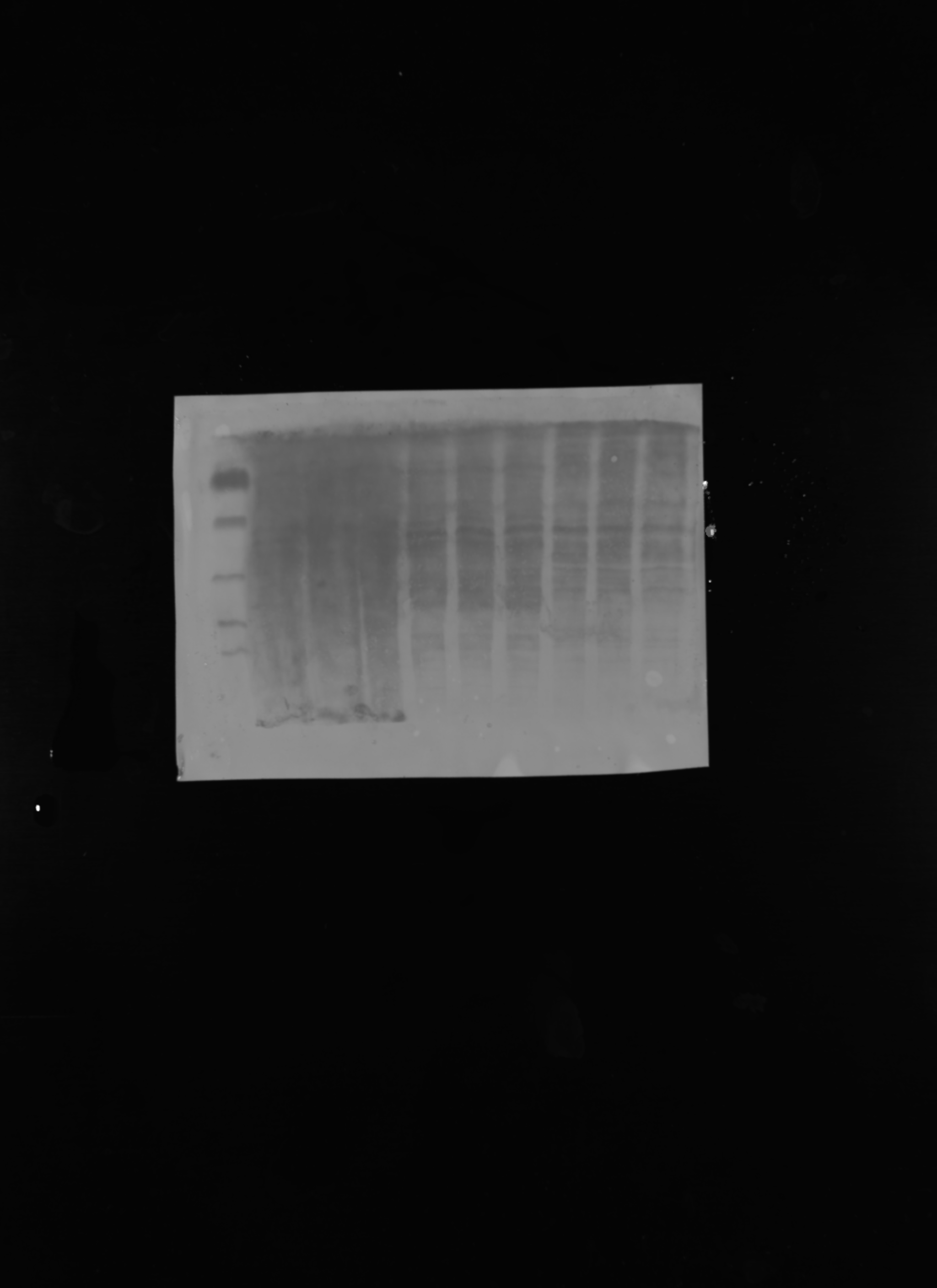

Supplement: Figure 5—source data 6. [file elife-87572-fig5-data6.zip › POLE/Rep1/P-6%-Mu-Q2 2023.01.09_17.56.57_Co/P-6%-Mu-Q2 2023.01.09_17.56.57_Co.tif]

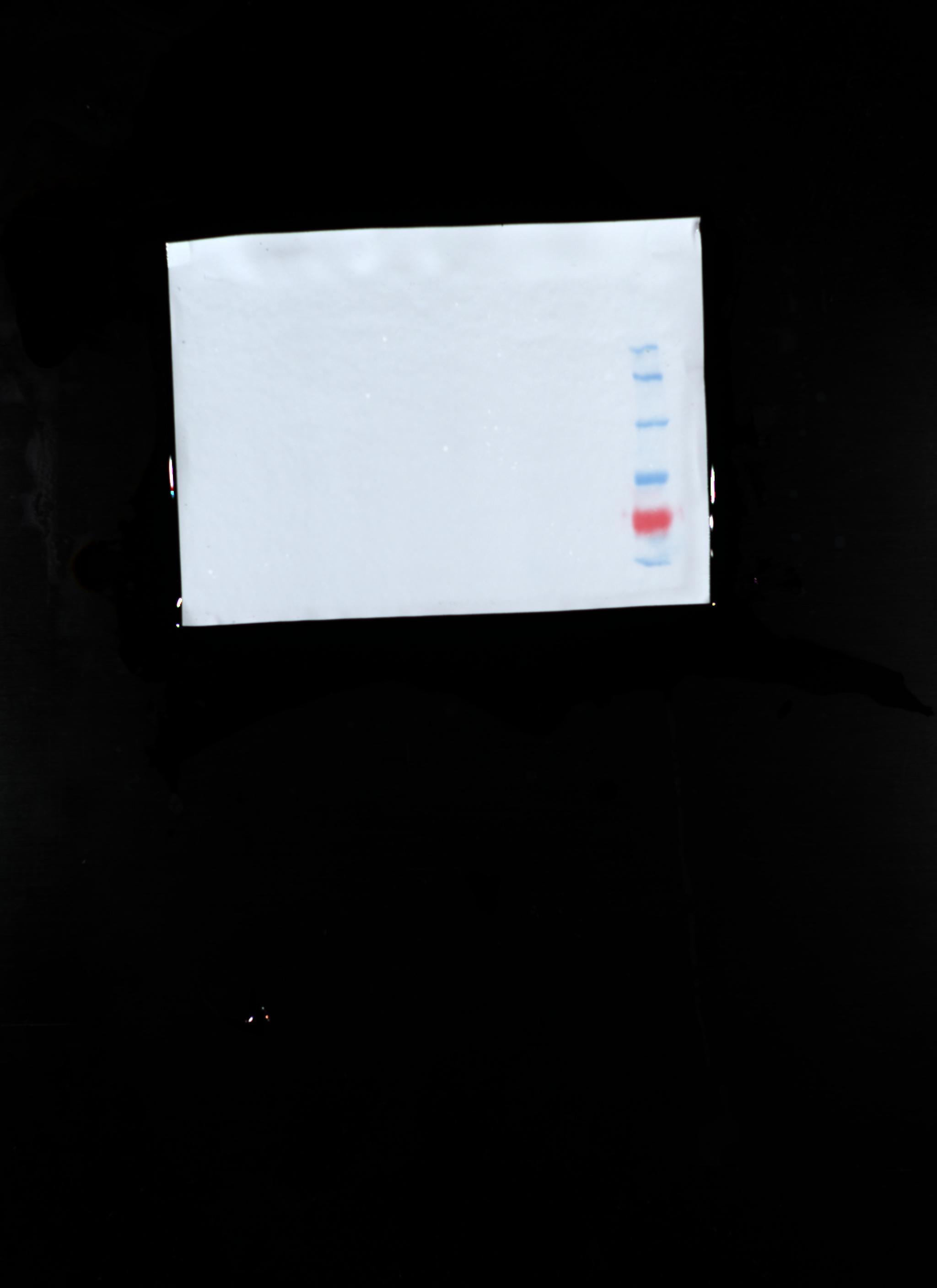

Supplement: Figure 5—source data 6. [file elife-87572-fig5-data6.zip › POLE/Rep1/polE-6%-Mu-Q2 2023.01.10_15.32.27_Fl-Green/polE-6%-Mu-Q2 2023.01.10_15.32.27_Fl-Green-Marker.jpg]

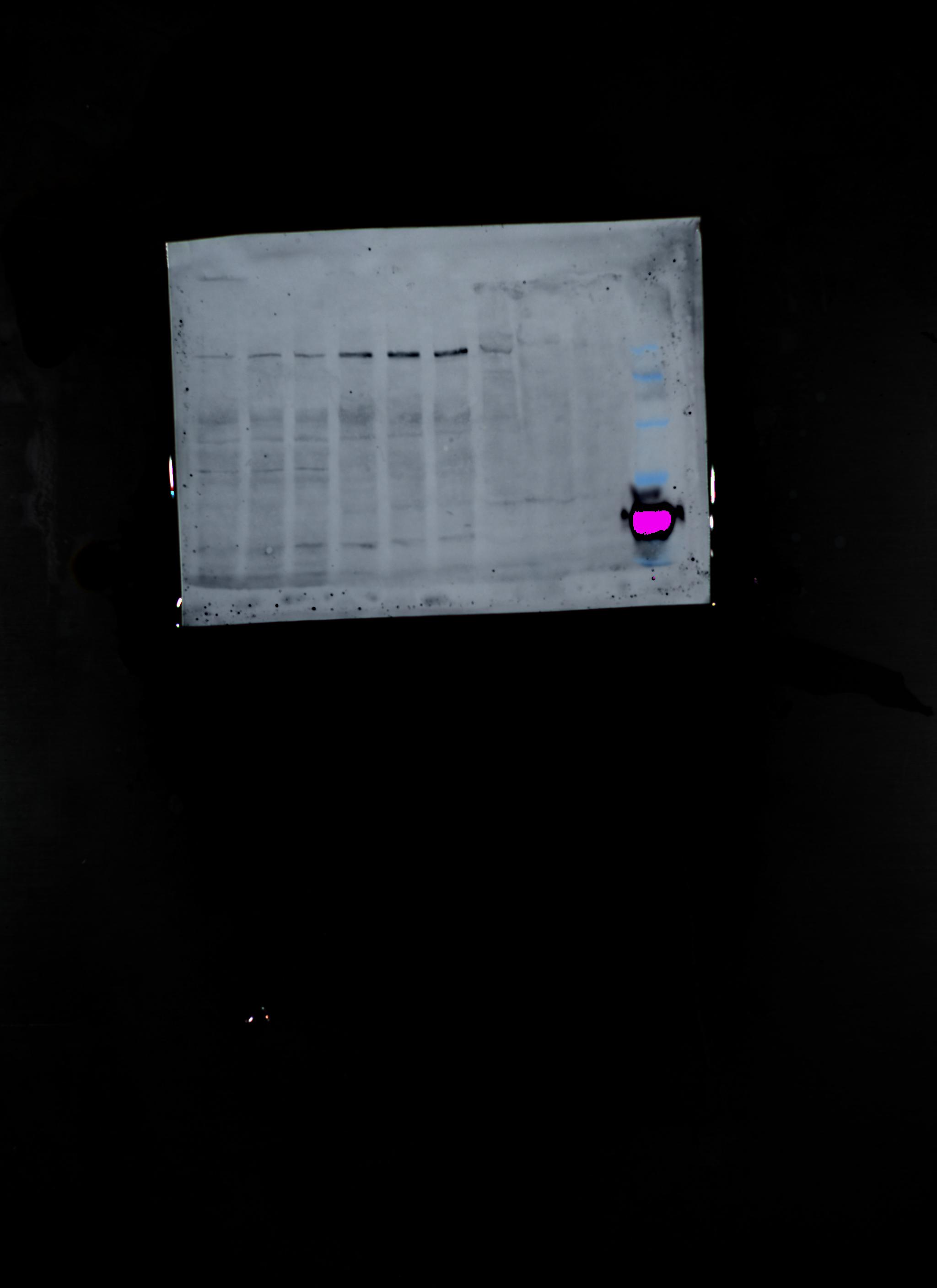

Supplement: Figure 5—source data 6. [file elife-87572-fig5-data6.zip › POLE/Rep1/polE-6%-Mu-Q2 2023.01.10_15.32.27_Fl-Green/polE-6%-Mu-Q2 2023.01.10_15.32.27_Fl-Green+Marker.jpg]

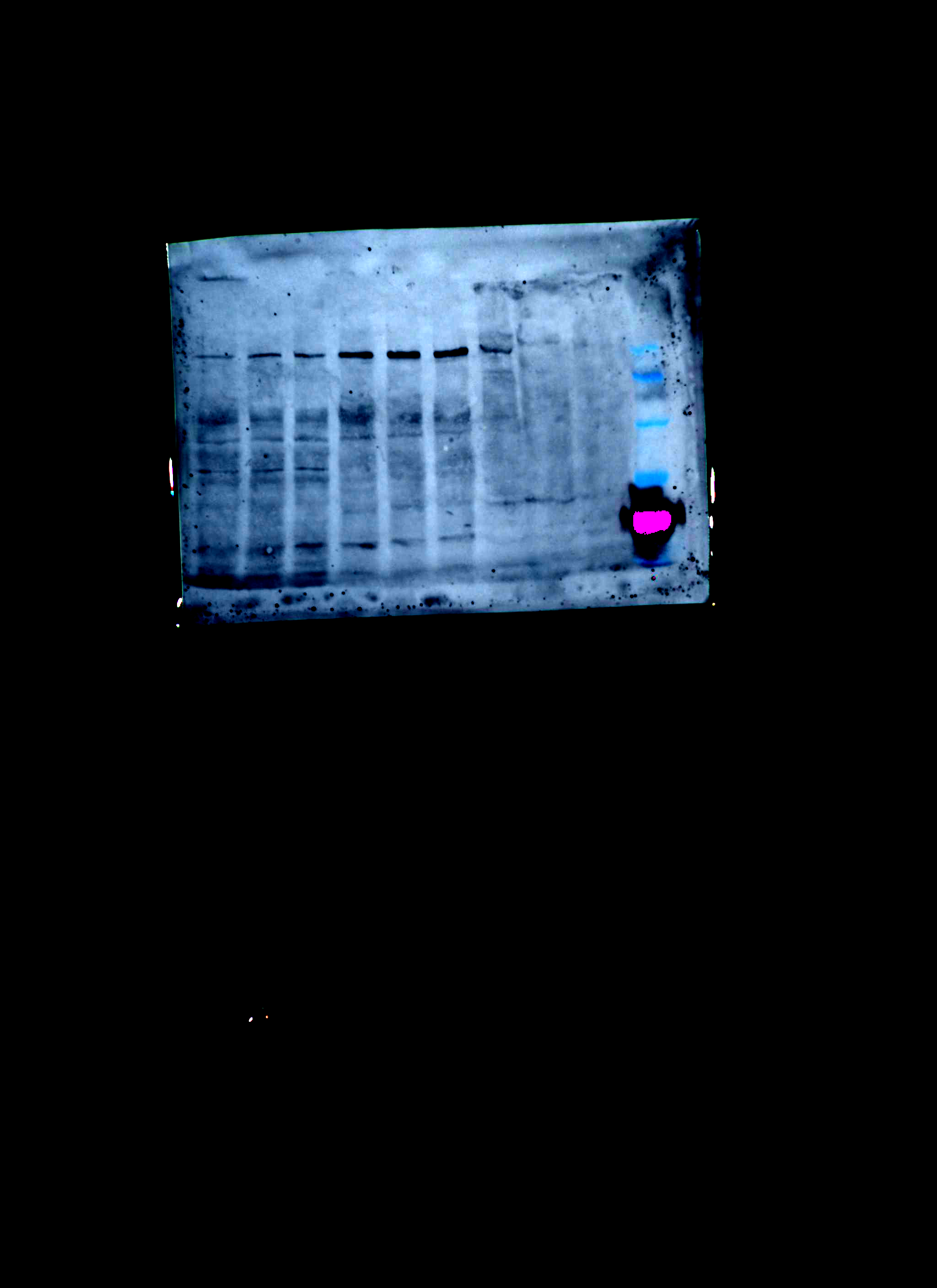

Supplement: Figure 5—source data 6. [file elife-87572-fig5-data6.zip › POLE/Rep1/polE-6%-Mu-Q2 2023.01.10_15.32.27_Fl-Green/polE-6%-Mu-Q2 2023.01.10_15.32.27_Fl-Green+Marker.tif]

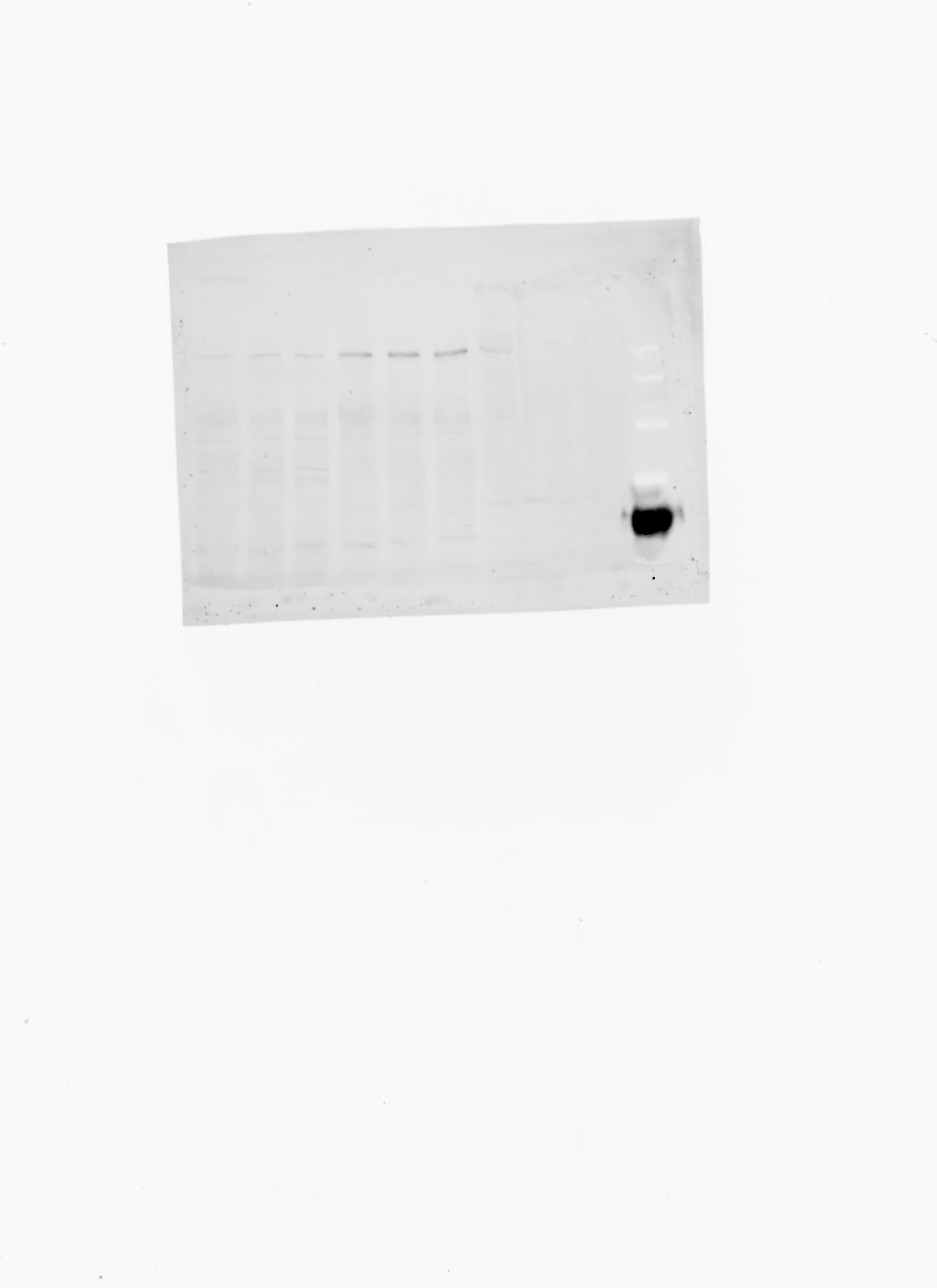

Supplement: Figure 5—source data 6. [file elife-87572-fig5-data6.zip › POLE/Rep1/polE-6%-Mu-Q2 2023.01.10_15.32.27_Fl-Green/polE-6%-Mu-Q2 2023.01.10_15.32.27_Fl-Green.tif]

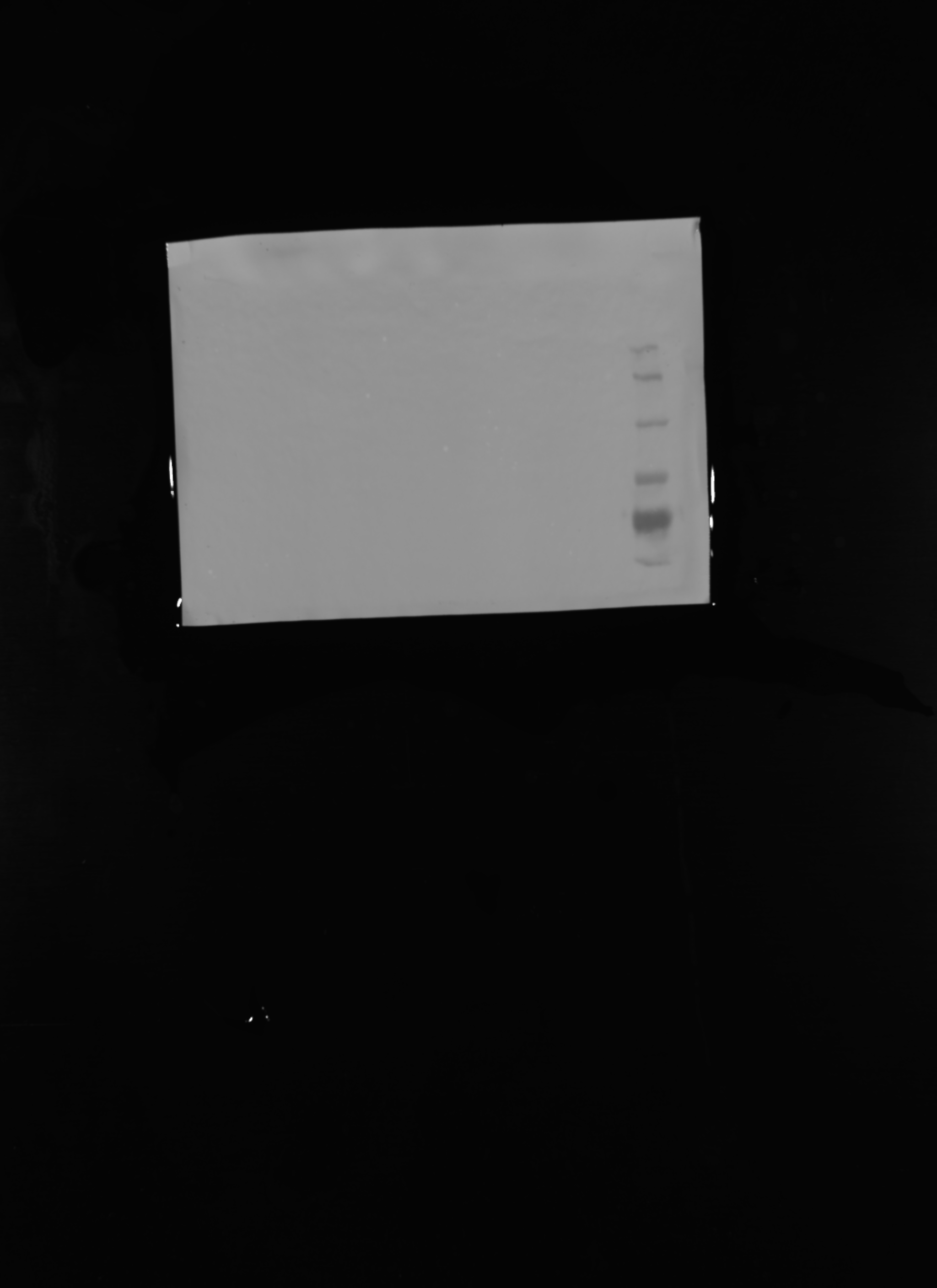

Supplement: Figure 5—source data 6. [file elife-87572-fig5-data6.zip › POLE/Rep1/polE-6%-Mu-Q2 2023.01.10_15.32.27_Fl-Green/polE-6%-Mu-Q2 2023.01.10_15.32.27_Fl-Green-Marker.tif]

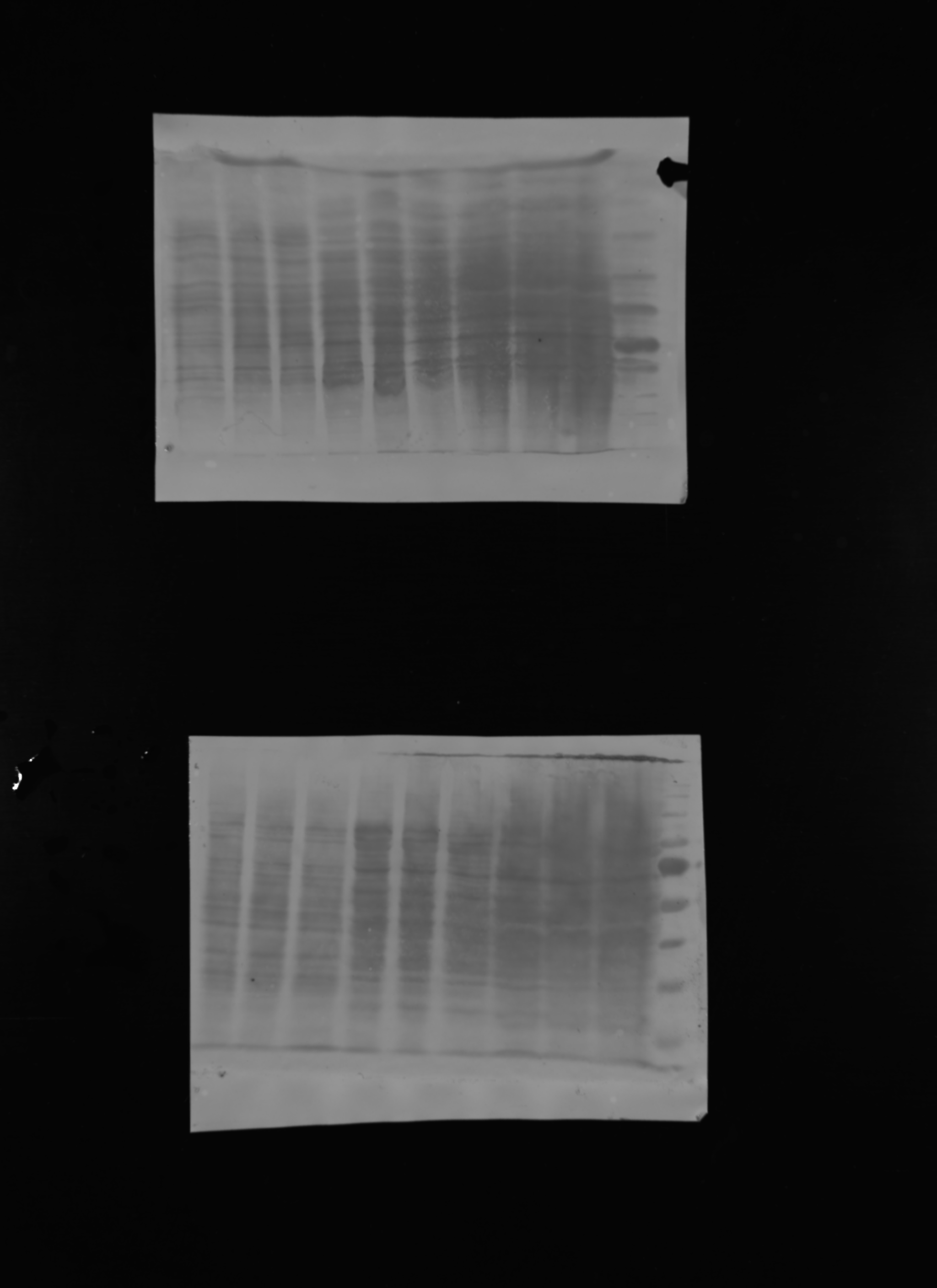

Supplement: Figure 5—source data 7. [file elife-87572-fig5-data7.zip › RPA34/Rep2/P-10%-RpaPcna-E1 2023.01.17_17.08.32_Co/P-10%-RpaPcna-E1 2023.01.17_17.08.32_Co.tif]

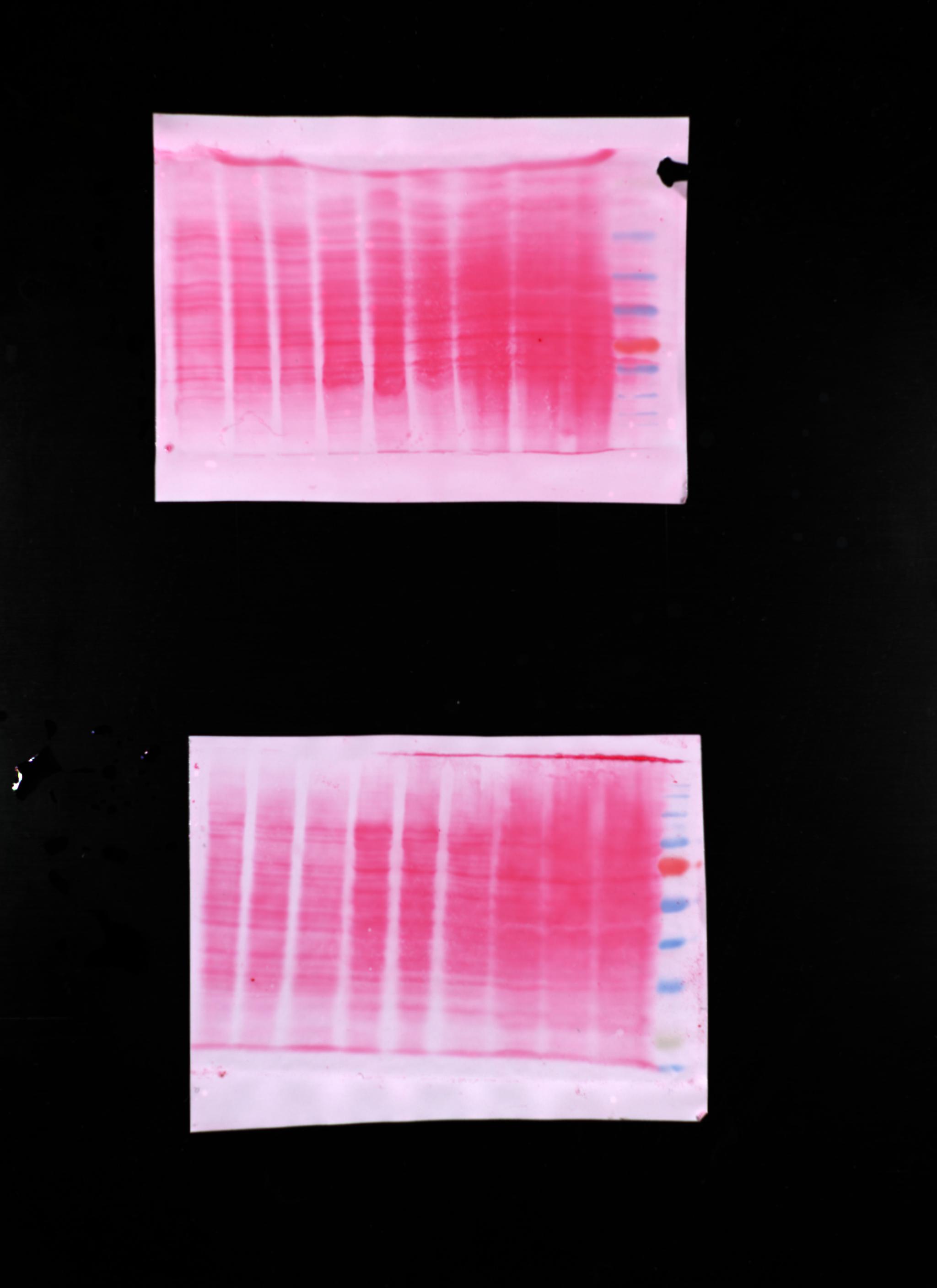

Supplement: Figure 5—source data 7. [file elife-87572-fig5-data7.zip › RPA34/Rep2/P-10%-RpaPcna-E1 2023.01.17_17.08.32_Co/P-10%-RpaPcna-E1 2023.01.17_17.08.32_Co.jpg]

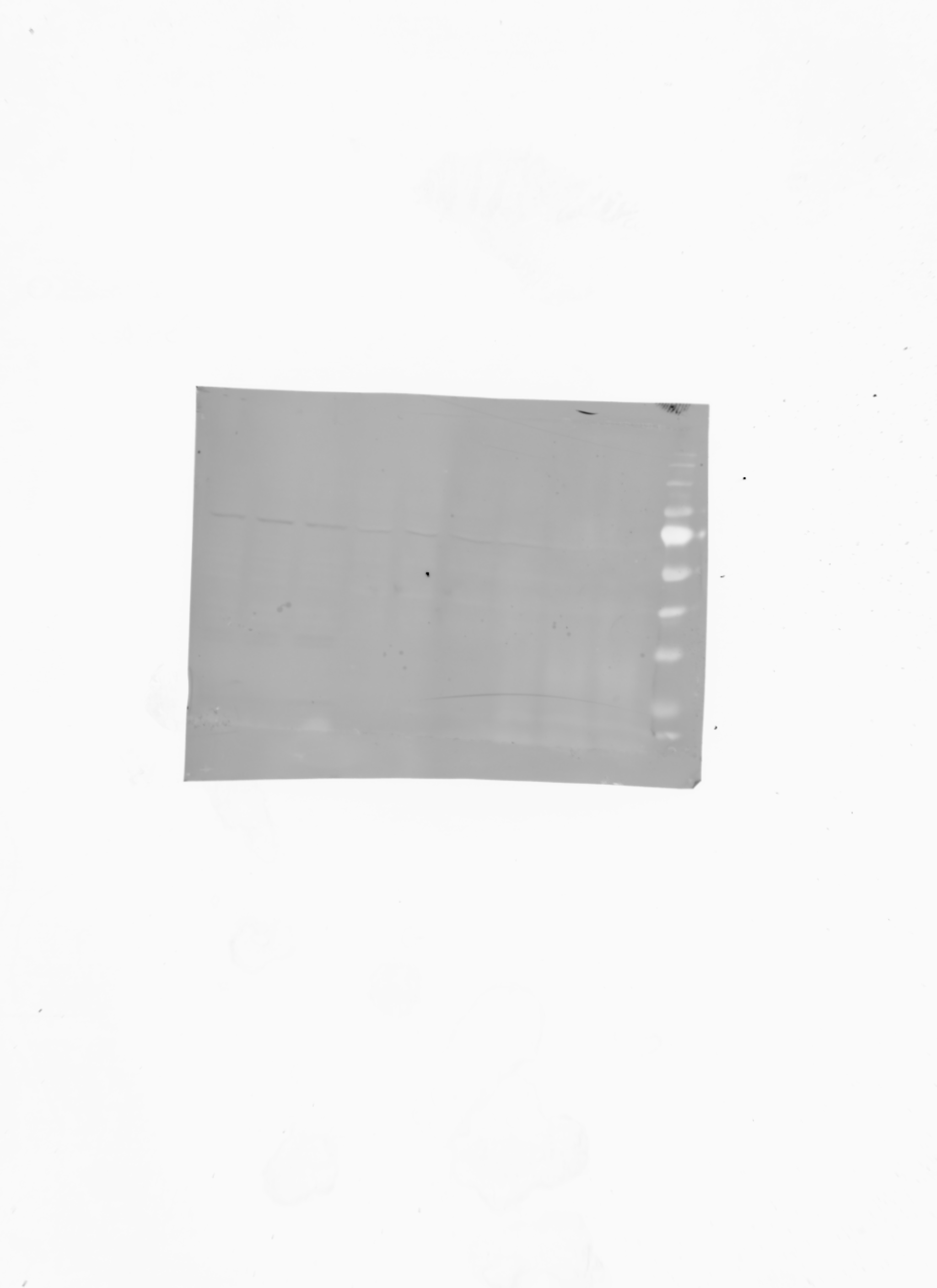

Supplement: Figure 5—source data 7. [file elife-87572-fig5-data7.zip › RPA34/Rep2/10%-Rpa34Pcna-E1 2023.01.18_15.32.22_Fl/10%-Rpa34Pcna-E1 2023.01.18_15.32.22_Fl-Blue.tif]

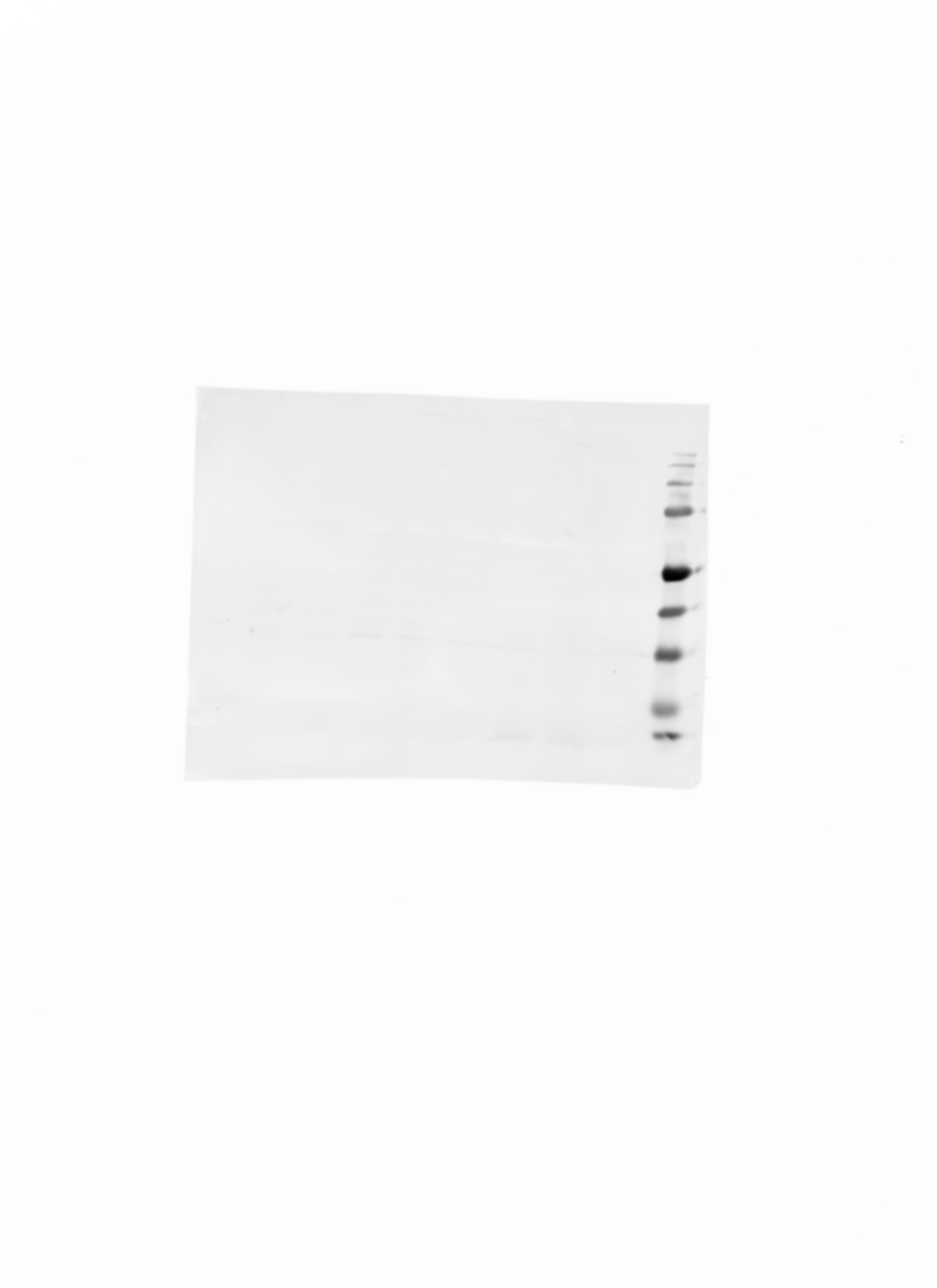

Supplement: Figure 5—source data 7. [file elife-87572-fig5-data7.zip › RPA34/Rep2/10%-Rpa34Pcna-E1 2023.01.18_15.32.22_Fl/10%-Rpa34Pcna-E1 2023.01.18_15.32.22_Fl-Red.tif]
