# Supplementary figures and images for: Replisome loading reduces chromatin motion independent of DNA synthesis (part 2 of 2)
Source: eLife. 2023 Oct 31;12:RP87572. doi: 10.7554/eLife.87572 (PMC10617993; doi:10.7554/eLife.87572)

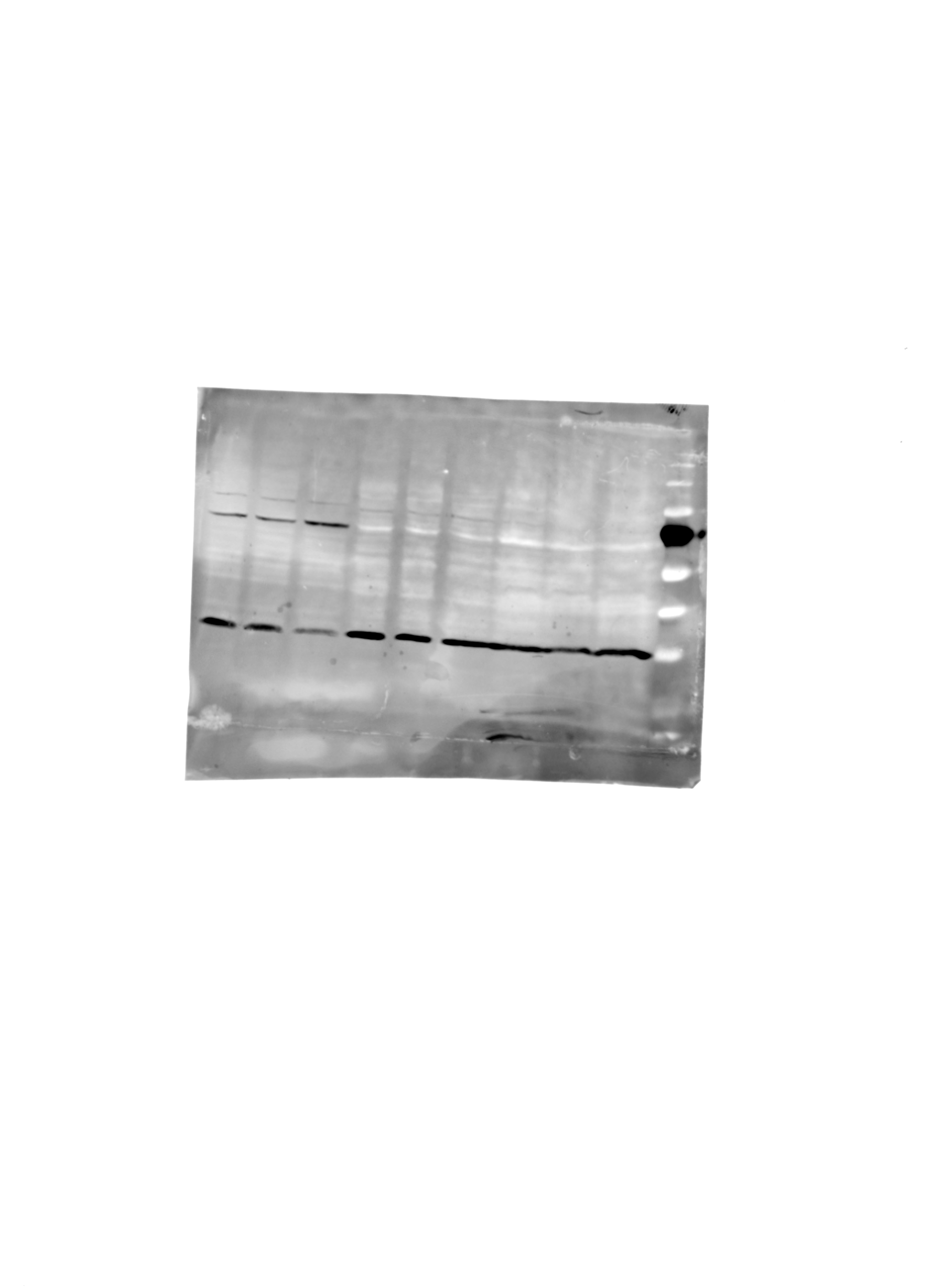

Supplement: Figure 5—source data 7. [file elife-87572-fig5-data7.zip › RPA34/Rep2/10%-Rpa34Pcna-E1 2023.01.18_15.32.22_Fl/10%-Rpa34Pcna-E1 2023.01.18_15.32.22_Fl-Green.tif]

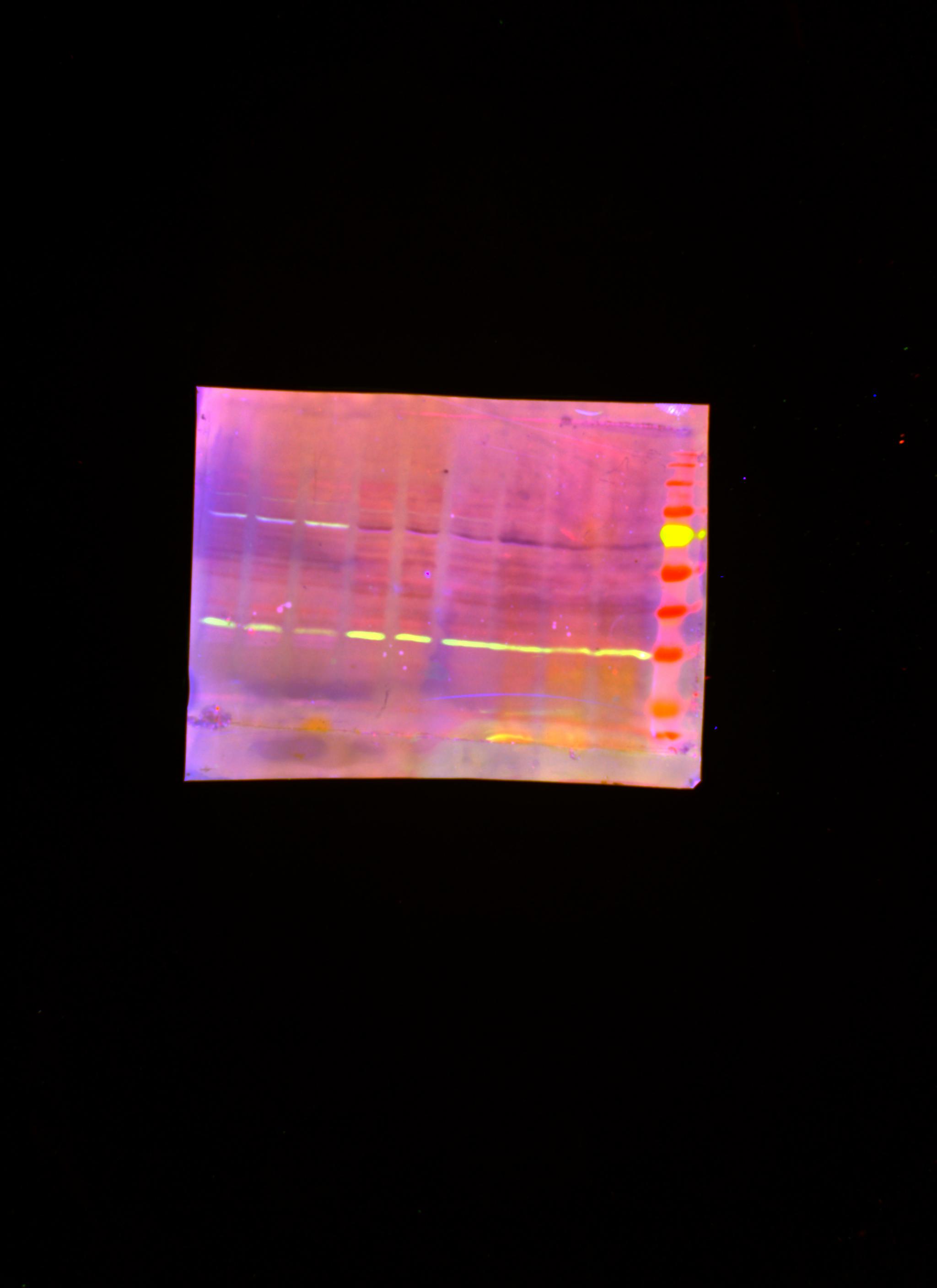

Supplement: Figure 5—source data 7. [file elife-87572-fig5-data7.zip › RPA34/Rep2/10%-Rpa34Pcna-E1 2023.01.18_15.32.22_Fl/10%-Rpa34Pcna-E1 2023.01.18_15.32.22_Fl.jpg]

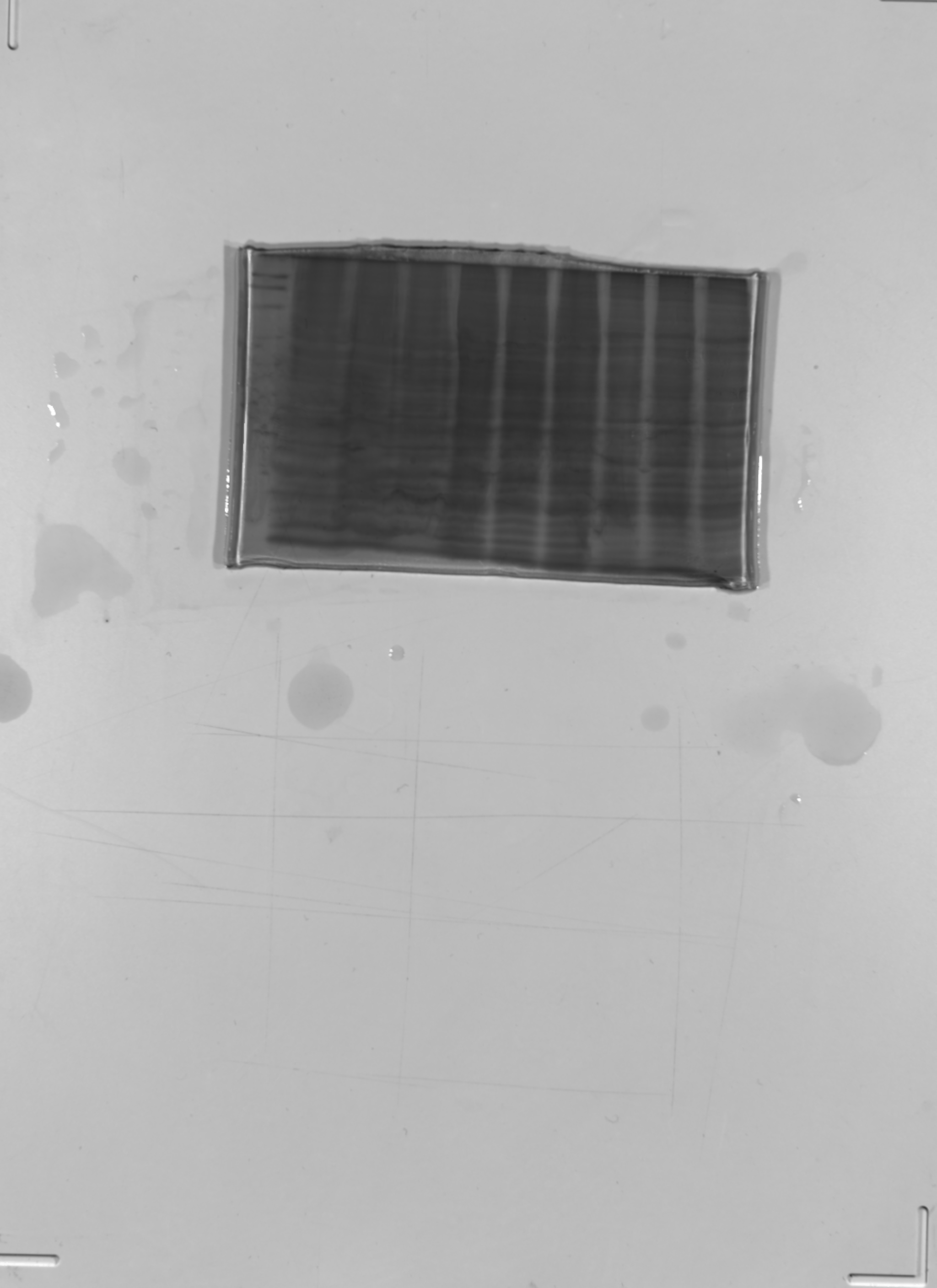

Supplement: Figure 5—source data 7. [file elife-87572-fig5-data7.zip › RPA34/Rep1/C-10%-Hu-Q3 2023.01.10_15.41.00_Co/C-10%-Hu-Q3 2023.01.10_15.41.00_Co.tif]

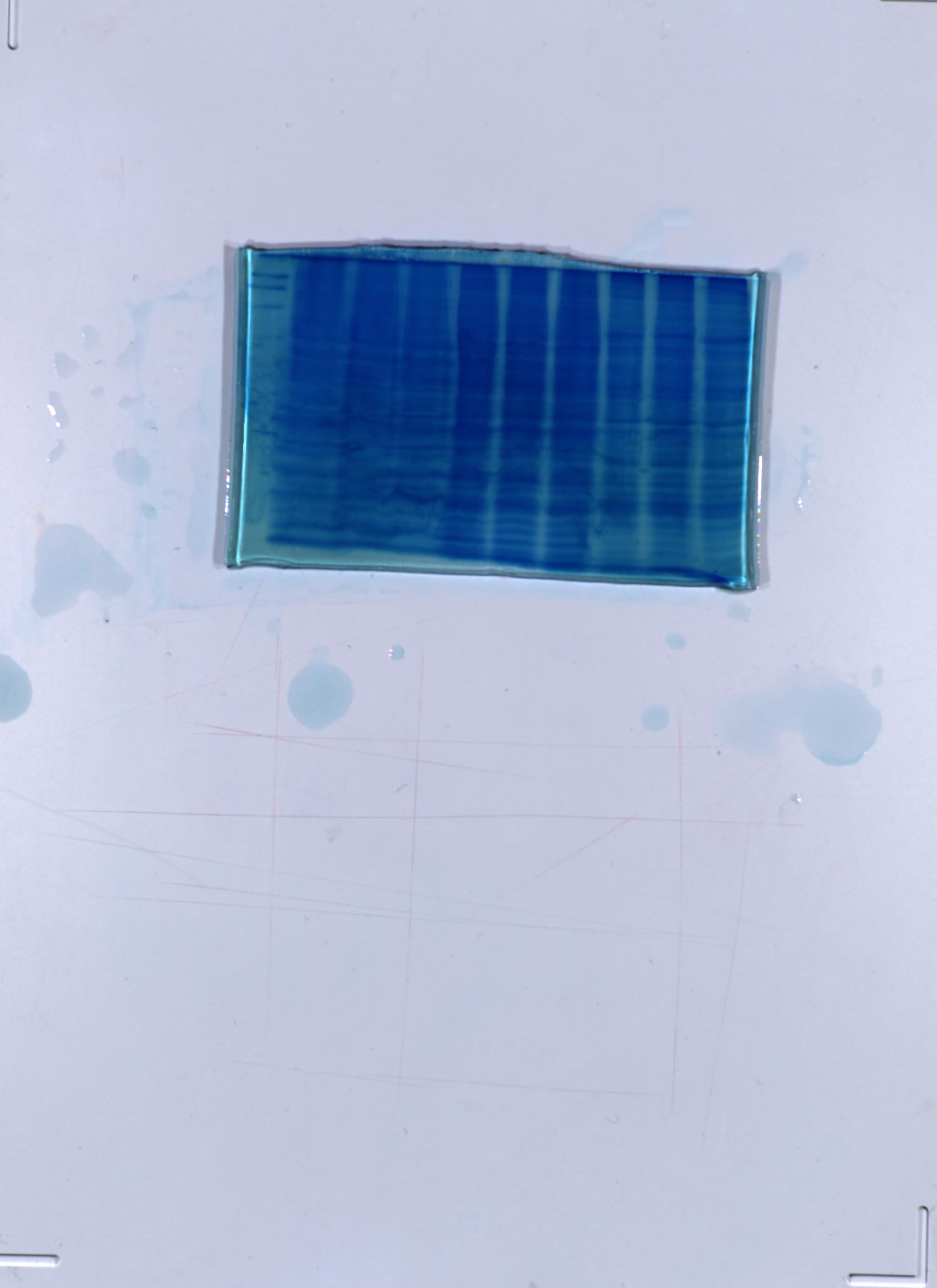

Supplement: Figure 5—source data 7. [file elife-87572-fig5-data7.zip › RPA34/Rep1/C-10%-Hu-Q3 2023.01.10_15.41.00_Co/C-10%-Hu-Q3 2023.01.10_15.41.00_Co.jpg]

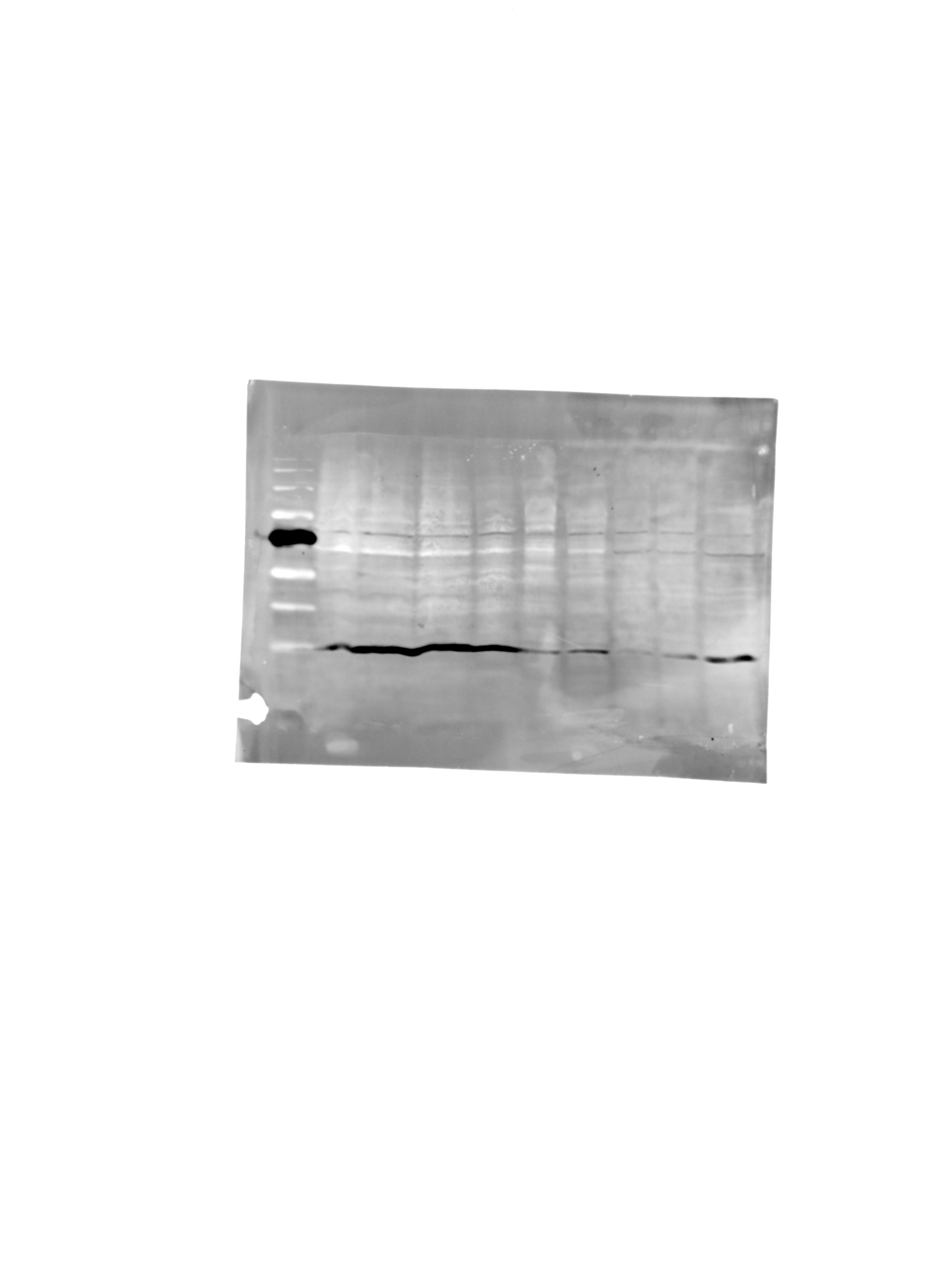

Supplement: Figure 5—source data 7. [file elife-87572-fig5-data7.zip › RPA34/Rep1/E2-rpa34-10% 2023.01.18_16.01.47_Fl-Green/E2-rpa34-10% 2023.01.18_16.01.47_Fl-Green.tif]

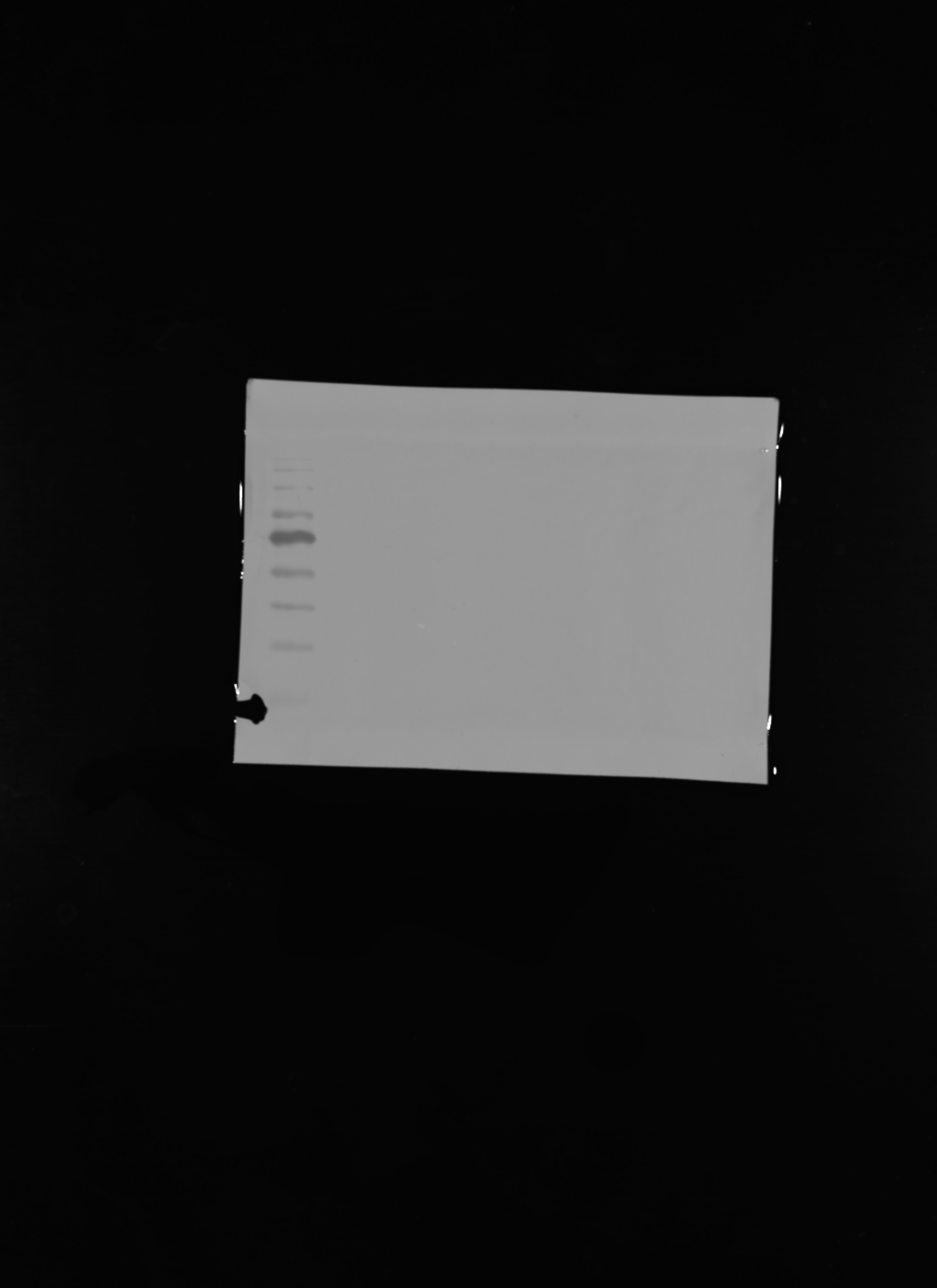

Supplement: Figure 5—source data 7. [file elife-87572-fig5-data7.zip › RPA34/Rep1/E2-rpa34-10% 2023.01.18_16.01.47_Fl-Green/E2-rpa34-10% 2023.01.18_16.01.47_Fl-Green-Marker.tif]

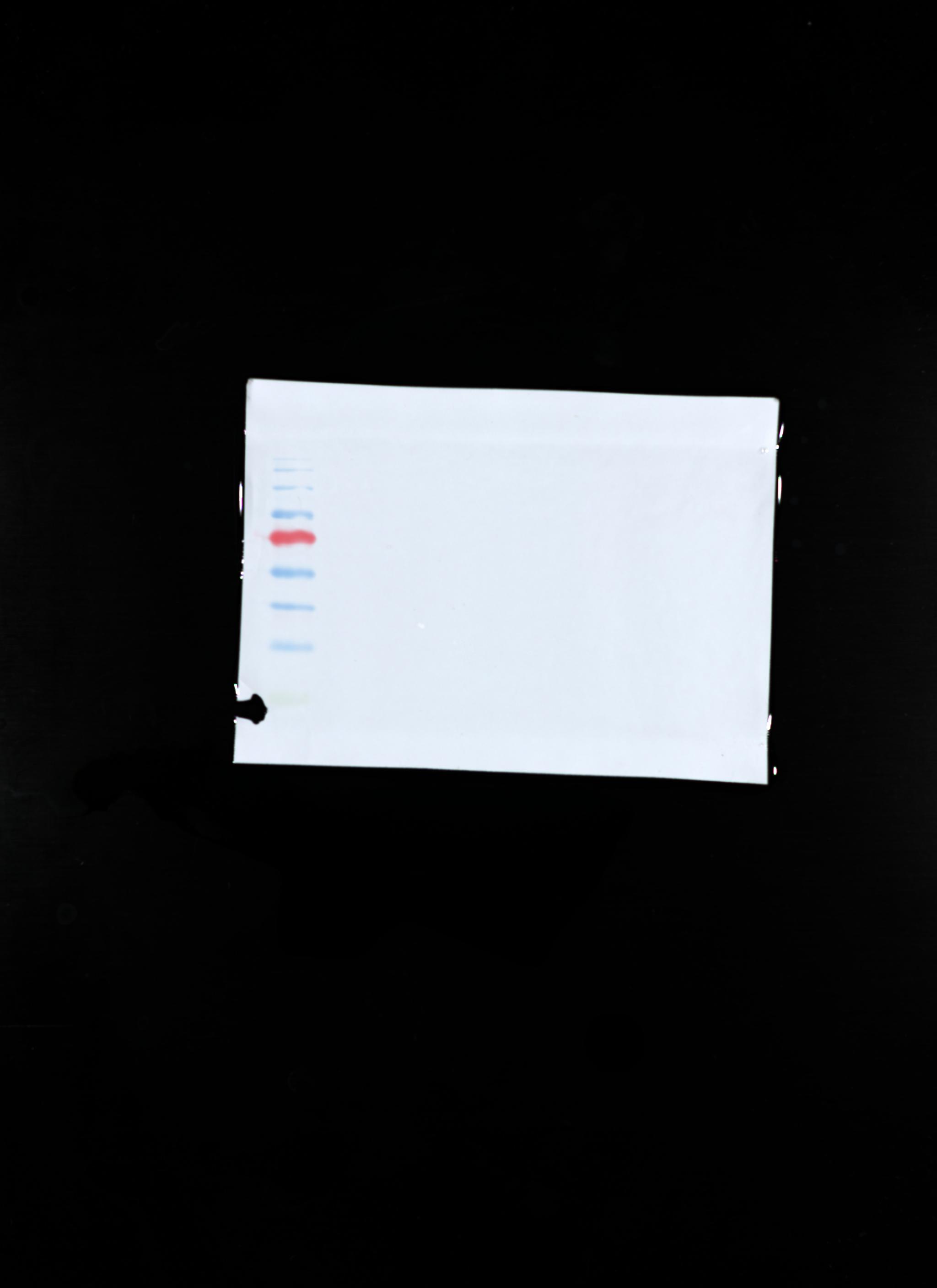

Supplement: Figure 5—source data 7. [file elife-87572-fig5-data7.zip › RPA34/Rep1/E2-rpa34-10% 2023.01.18_16.01.47_Fl-Green/E2-rpa34-10% 2023.01.18_16.01.47_Fl-Green-Marker.jpg]

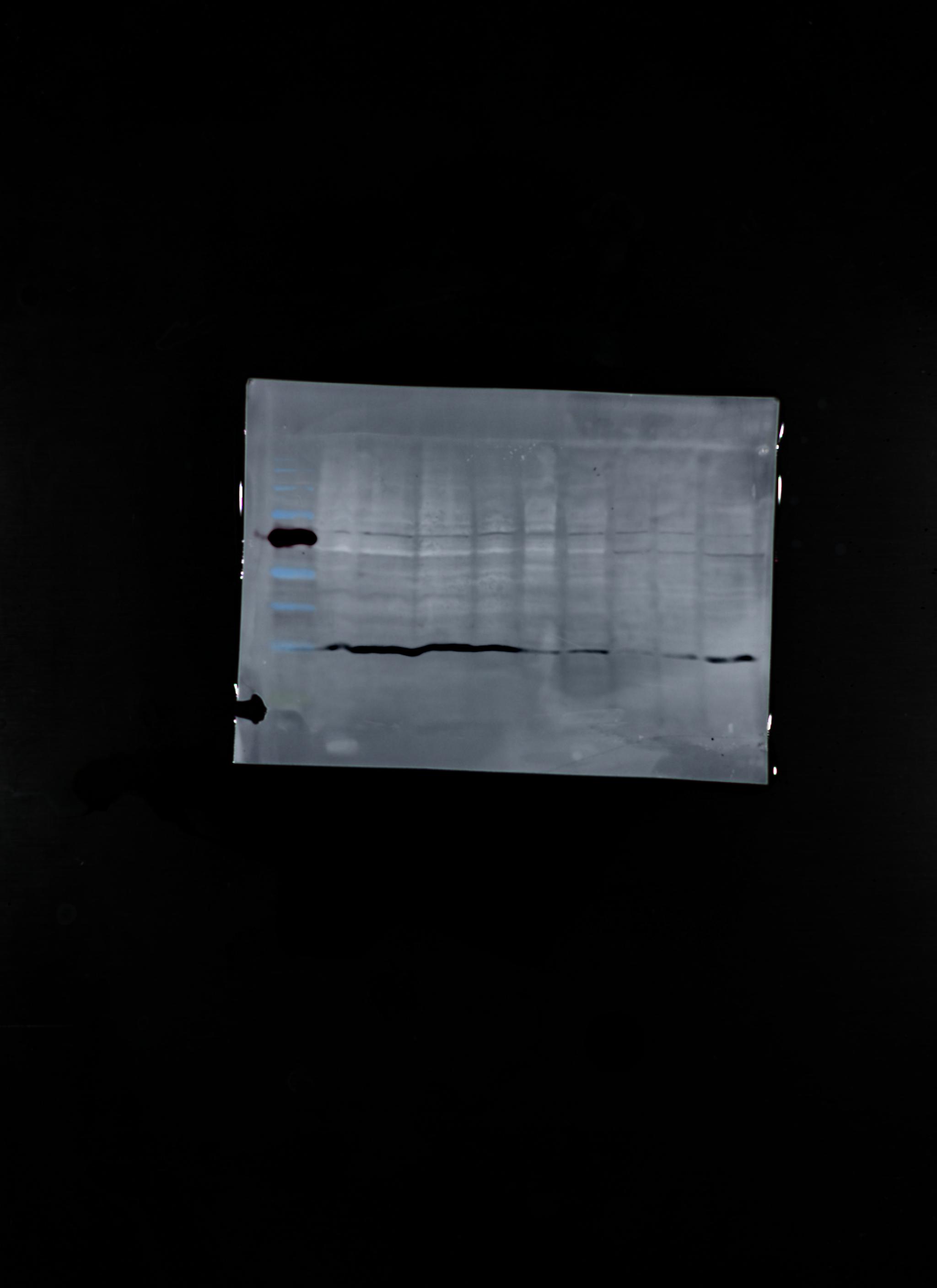

Supplement: Figure 5—source data 7. [file elife-87572-fig5-data7.zip › RPA34/Rep1/E2-rpa34-10% 2023.01.18_16.01.47_Fl-Green/E2-rpa34-10% 2023.01.18_16.01.47_Fl-Green+Marker.jpg]

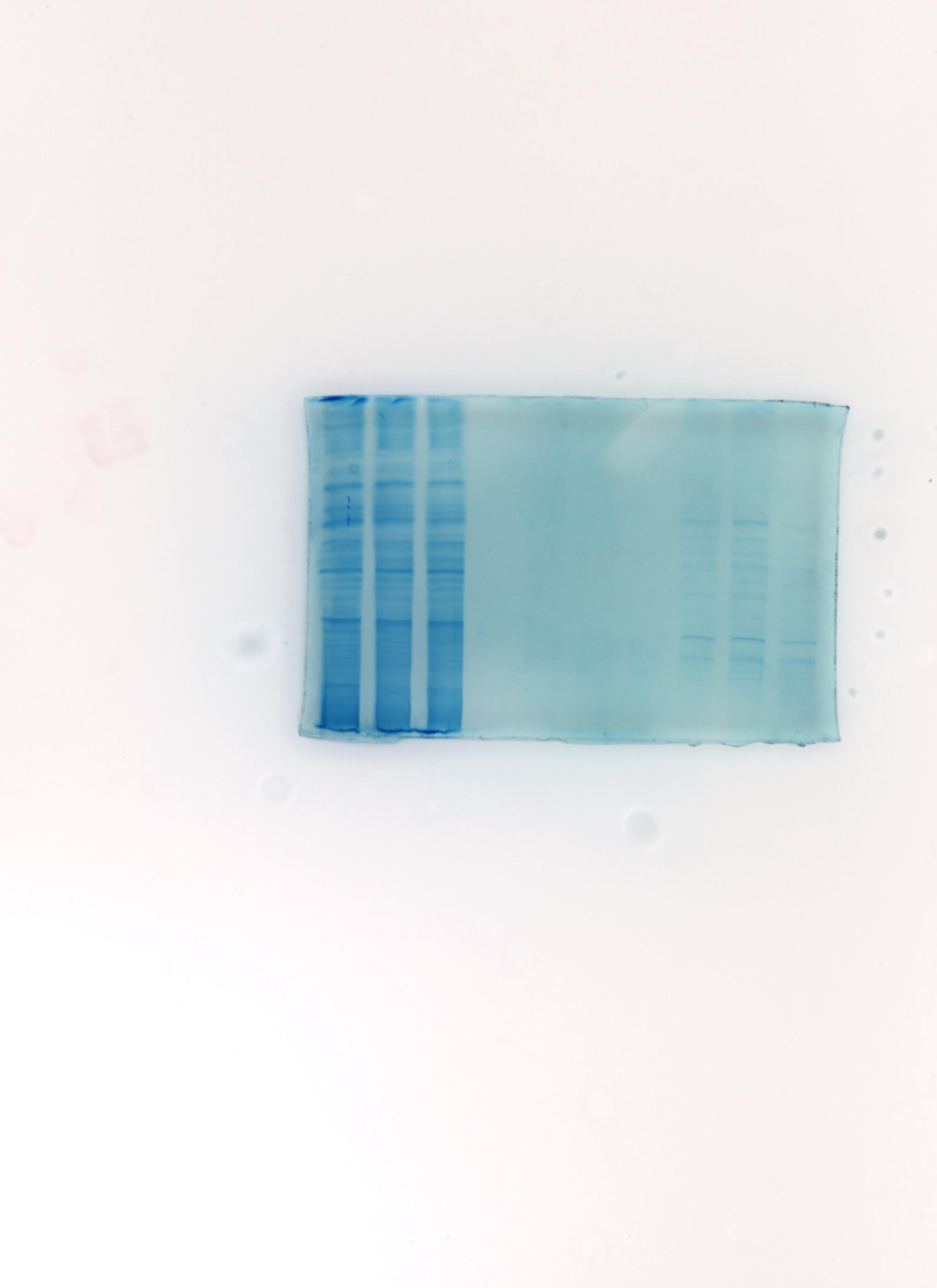

Supplement: Figure 5—source data 8. [file elife-87572-fig5-data8.zip › Tubulin/Rep2/AK E1 E2 E3 Ctr HU Aph 6% 2022.07.28_11.50.34_Co/AK E1 E2 E3 Ctr HU Aph 6% 2022.07.28_11.50.34_Co.jpg]

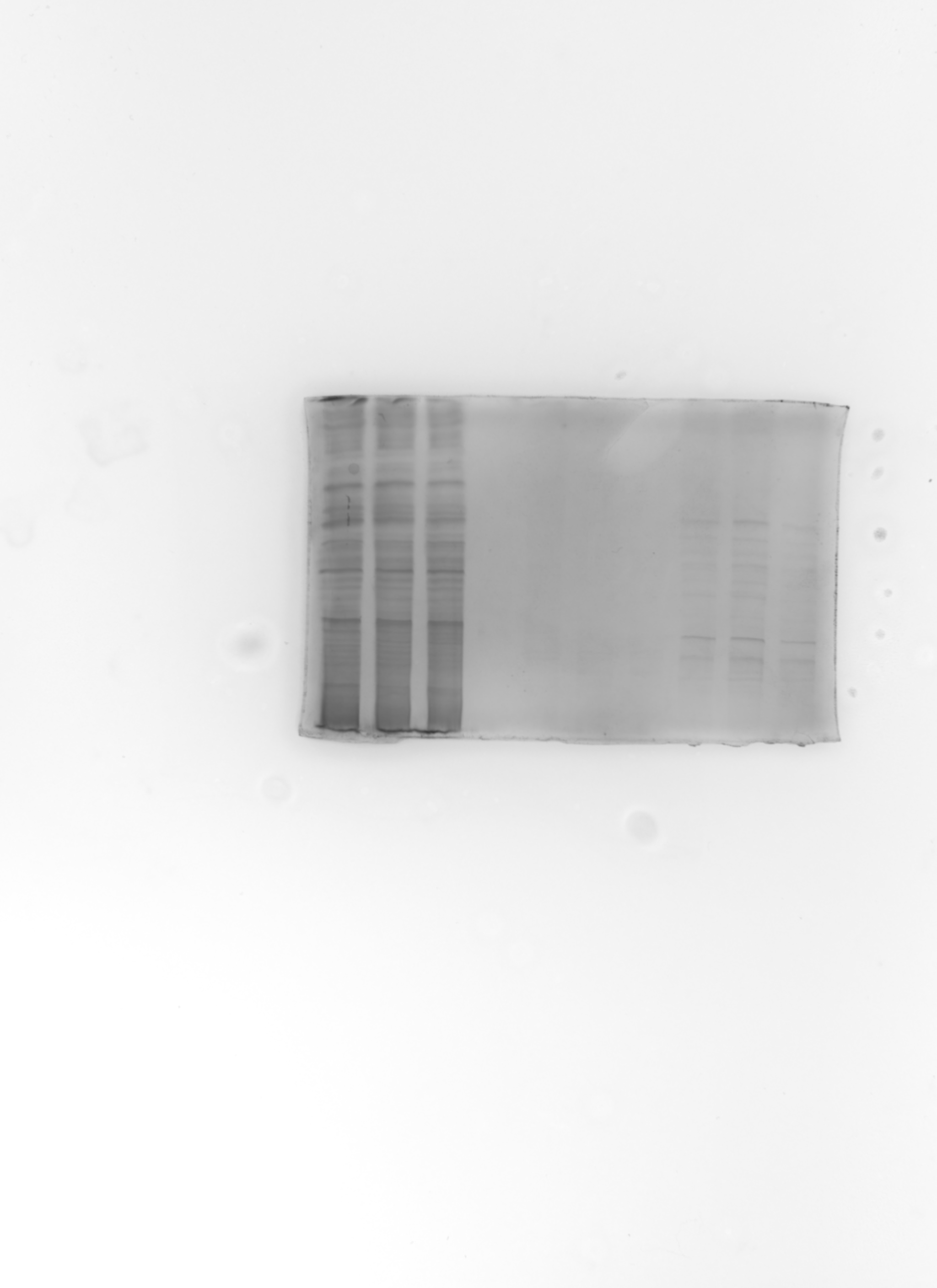

Supplement: Figure 5—source data 8. [file elife-87572-fig5-data8.zip › Tubulin/Rep2/AK E1 E2 E3 Ctr HU Aph 6% 2022.07.28_11.50.34_Co/AK E1 E2 E3 Ctr HU Aph 6% 2022.07.28_11.50.34_Co.tif]

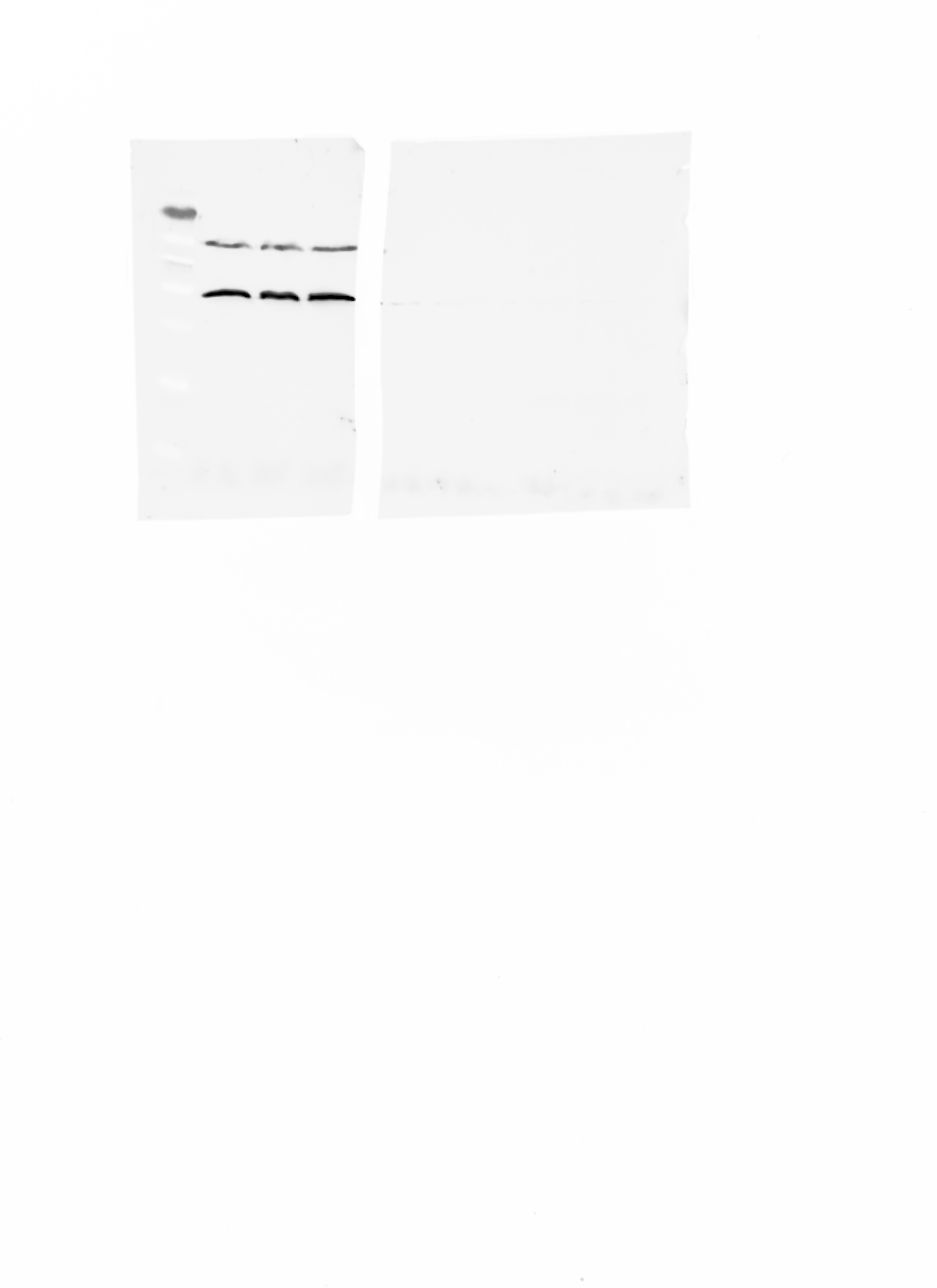

Supplement: Figure 5—source data 8. [file elife-87572-fig5-data8.zip › Tubulin/Rep1/wb pcna tub h2ax 2022.07.20_11.24.26_Fl/wb pcna tub h2ax 2022.07.20_11.24.26_Fl-Green.tif]

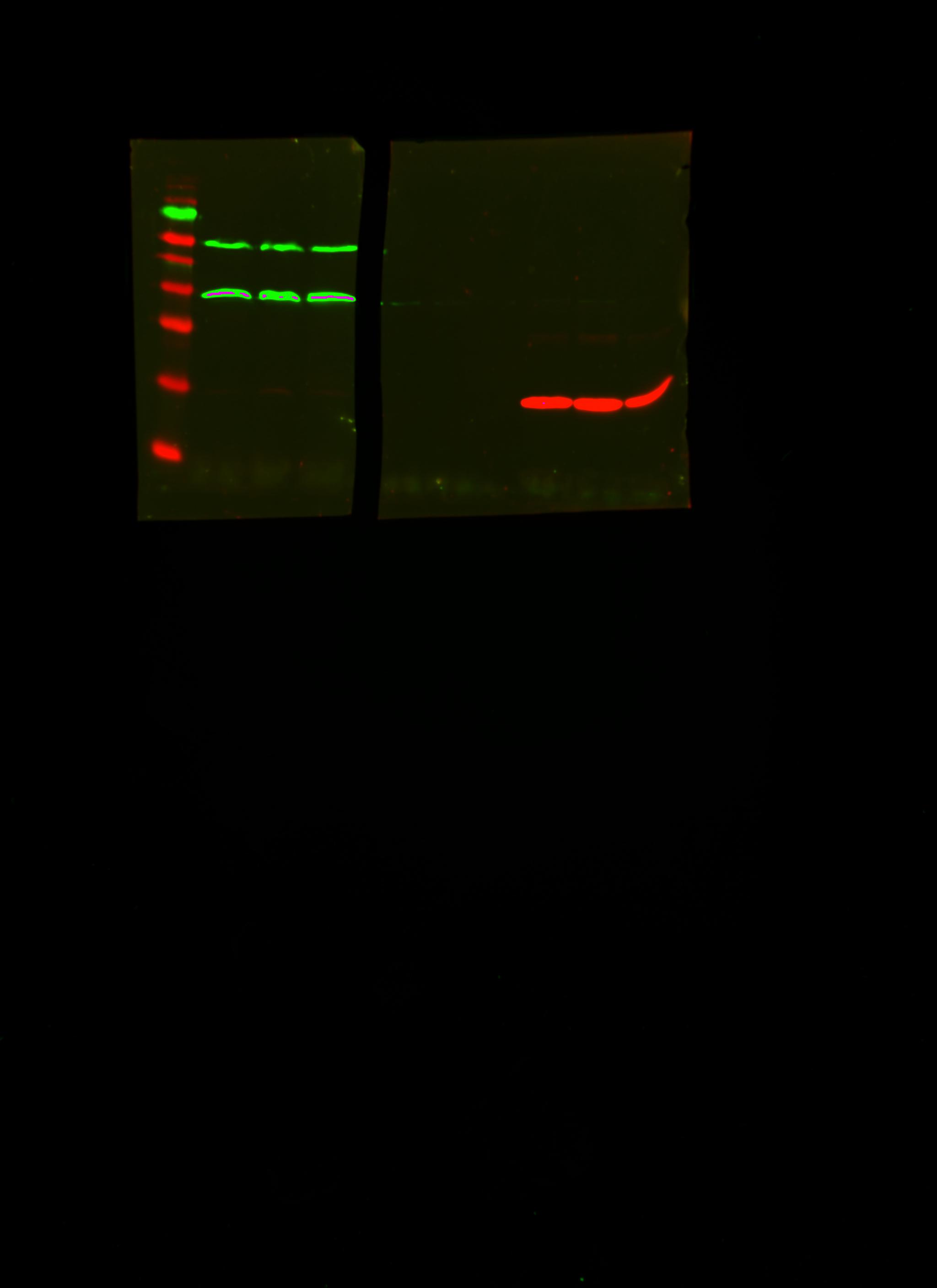

Supplement: Figure 5—source data 8. [file elife-87572-fig5-data8.zip › Tubulin/Rep1/wb pcna tub h2ax 2022.07.20_11.24.26_Fl/wb pcna tub h2ax 2022.07.20_11.24.26_Fl.jpg]

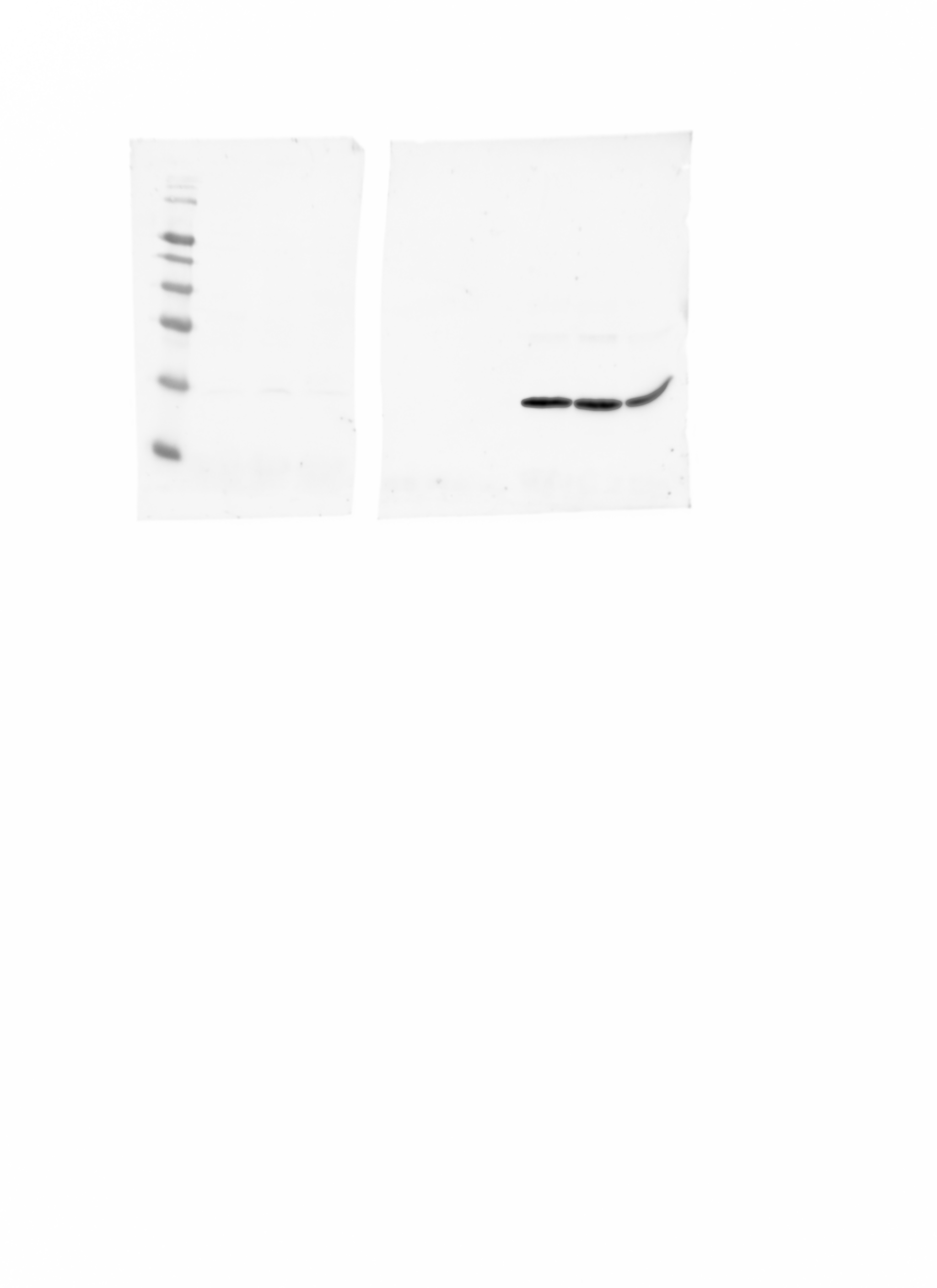

Supplement: Figure 5—source data 8. [file elife-87572-fig5-data8.zip › Tubulin/Rep1/wb pcna tub h2ax 2022.07.20_11.24.26_Fl/wb pcna tub h2ax 2022.07.20_11.24.26_Fl-Red.tif]
