# Supplementary material for: Replisome loading reduces chromatin motion independent of DNA synthesis
Source: eLife. 2023 Oct 31;12:RP87572. doi: 10.7554/eLife.87572 (PMC10617993; doi:10.7554/eLife.87572)
Supplement: Supplementary file 1. [file elife-87572-supp1.docx]

**SUPPLEMENTARY TABLES AND FIGURES**

**Supplementary file 1a: Cell line characteristics**

| **Name** | **Species** | **Type** | **Genome size ( Gbp)** | **Reference** |
| --- | --- | --- | --- | --- |
| HeLa K | Homo sapiens | Cervical adenocarcinoma | 9.682±0.002 | [(Erfle et al., 2007)](https://sciwheel.com/work/citation?ids=1001802&pre=&suf=&sa=0&dbf=0) |
| HeLa K GFP-PCNA | Homo sapiens | Cervical adenocarcinoma | 9.682±0.002 | [(Chagin et al., 2016)](https://sciwheel.com/work/citation?ids=2069726&pre=&suf=&sa=0&dbf=0) |
| HeLa K GFP-RPA34 | Homo sapiens | Cervical adenocarcinoma | 9.682±0.002 | This study |
| HeLa K FRTLacZ | Homo sapiens | Cervical adenocarcinoma | 9.682±0.002 | [(Chagin et al., 2016)](https://sciwheel.com/work/citation?ids=2069726&pre=&suf=&sa=0&dbf=0) |
| IMR90 | Homo sapiens | Fibroblasts from lung tissue | 6.37 | [(Nichols et al., 1977)](https://sciwheel.com/work/citation?ids=8508791&pre=&suf=&sa=0&dbf=0) |

**Supplementary file 1b: Plasmid characteristics**

| **Name** | **pc number** | **Fluorophore** | **Protein of Interest** | **Promoter** | **References** |
| --- | --- | --- | --- | --- | --- |
| pmiRFP670-PCNA | 3385 | miRFP670 | Human PCNA | CMV | [(Rausch et al., 2021)](https://sciwheel.com/work/citation?ids=11830976&pre=&suf=&sa=0&dbf=0) |
| pFRT-B-GRPA | 1232 | GFP | Human RPA34 | EF1⍺ | This study |
| pFRT-B-GPCNA | 1274 | GFP | Human PCNA | EF1⍺ | [(Chagin et al., 2016)](https://sciwheel.com/work/citation?ids=2069726&pre=&suf=&sa=0&dbf=0) |

* pc: plasmid collection.

**Supplementary file 1c: Nucleotide and chemical characteristics**

| **Name** | **Application** | **Detection** | **Cat #** | **Company** |
| --- | --- | --- | --- | --- |
| Cy3-dUTP | Replication labeling  (Labeling of nascent DNA) | - | ENZ-42501 | Enzo life sciences,  Farmingdale, NY, USA |
| Aphidicolin | Replisome disruption by polymerase inhibition | - | A0781-1MG | Sigma-Aldrich, St  Louis, MO, USA |
| 5-ethynyl-2’-  deoxyuridine (EdU) | Labeling of nascent DNA  in pulse (chase) experiments | ClickIT chemistry | E10415 | Thermo Fisher  Scientific, Waltham,  MA, USA |

**Supplementary file 1d: Primary and secondary antibody characteristics**

| **Reactivity** | **Host** | **Clonality** | **Dilution** | **Application** | **Cat / Clone^#^** | **Company / References** |
| --- | --- | --- | --- | --- | --- | --- |
| anti RPA34 | Mouse | Monoclonal | 1:2 | IF, WB | 9H8H4**^#^** | Gift from Mark  Kenny/J. Hurwitz  [(Kenny et al., 1990)](https://sciwheel.com/work/citation?ids=1287800&pre=&suf=&sa=0) |
| anti RPA70A | Mouse | Monoclonal | 1:2 | IF, WB | 7G9E3**^#^** | Gift from Mark  Kenny/J. Hurwitz  [(Kenny et al., 1990)](https://sciwheel.com/work/citation?ids=1287800&pre=&suf=&sa=0) |
| Anti MCM2 | Rabbit | Monoclonal | 1:5000 IF, 1:10000 WB | IF, WB | ab 108935/  EPR4120 | Abcam,  Cambridge,  United Kingdom |
| anti MCM2pS108 | Rabbit | Monoclonal | 1:1000 | IF,WB | 3267-1 | Epitomics,  Burlingame​, CA, United States |
| anti pol Alpha | Mouse | Monoclonal | Undiluted | IF,WB | SJK-287-38**^#^** | ATCC  [(Tanaka et al., 1982)](https://sciwheel.com/work/citation?ids=2084999&pre=&suf=&sa=0) |
| anti pol Delta | Mouse | Monoclonal | 1:500 | IF,WB | 610972 | BD biosciences,  New Jersey, USA  [(Li et al., 2016)](https://sciwheel.com/work/citation?ids=3106104&pre=&suf=&sa=0) |
| anti pol Epsilon | Rabbit | Polyclonal | 1:500 | IF, WB | GTX132100 | GeneTex, Irvine, California, United States |
| anti PCNA | Mouse | Monoclonal | 1:100 | IF*,WB | M0879 / PC10**^#^** | Dako,  Hamburg,  Germany  [(Waseem and Lane, 1990)](https://sciwheel.com/work/citation?ids=2085678&pre=&suf=&sa=0) |
| anti Histone H3 | Rat | Monoclonal | 1:250 | WB | 61647/  1C8B2^#^ | Active Motif,  California, USA |
| anti MacroH2A1 | Rabbit | Polyclonal | 1:1000 | WB | 07-219 | Active Motif,  California, USA |
| anti GFP | Rat | Monoclonal | 1:1000 | WB | 3H9**^#^** | Chromotek 3H9,  Planegg-Martinsried, Germany |
| Anti tubulin alpha | Mouse | Monoclonal | 1:5000 | WB | clone DM1A^#^/ T9026 | Sigma,  Missouri,  United States |
| anti-mouse  IgG Cy3 | Donkey | Polyclonal | 1:800 | IF (fluorescent  secondary) | 715-165-151 | The Jackson  Laboratory, Bar  Harbor, ME, USA |
| anti-rabbit  IgG Cy3 | Donkey | Polyclonal | 1:800 | IF (fluorescent  secondary) | 711-165-152 | The Jackson  Laboratory, Bar  Harbor, ME, USA |
| anti-mouse  IgG Cy5 | Donkey | Polyclonal | 1:800 | IF (fluorescent  secondary) | 715-175-150 | The Jackson  Laboratory, Bar  Harbor, ME, USA |
| anti-rabbit  IgG Cy5 | Donkey | Polyclonal | 1:800 | IF (fluorescent  secondary) | 711-175-152 | The Jackson  Laboratory, Bar  Harbor, ME, USA |
| anti-mouse  IgG AF488 | Goat | Polyclonal | 1:800 | IF (fluorescent  secondary) | 2120125 | Invitrogen,  Waltham, Massachusetts, USA |
| anti-rabbit  IgG AF488 | Donkey | Polyclonal | 1:800 | IF (fluorescent  secondary) | A11034 | Invitrogen,  Waltham, Massachusetts, USA |
| anti rat IgG HRP | Goat | Polyclonal | 1:5000 | WB  (HRP secondary) | 112-035-068 | The Jackson  Laboratory, Bar  Harbor, ME, USA |
| anti mouse IgG HRP | Sheep | Polyclonal | 1:5000 | WB  (HRP secondary) | NA931 | Amersham pharmacia, Amersham, United Kingdom |

*****Methanol treatment required #Clone number

**Supplementary file 1e: Imaging systems characteristics**

| **Microscope/**  **Company** | **Lasers/lamps** | **Filters (ex. &**  **em. [nm])*** | **Objectives/**  **lenses** | **Detection**  **system** | **Incubation**  **system** | **Application** |
| --- | --- | --- | --- | --- | --- | --- |
| Ultra-View  VoX  spinning disk  microscope/  PerkinElmer  Life  Sciences,  UK | solid state  diode lasers  (405 nm,  488 nm,  561 nm,  640 nm) | 405/488/56  8/640**  405: 415–475  488: 505–549  561: 580–650  640: 664–754 | oil immersion  60x Plan-  Apochromat  (NA 1.45) | cooled 14-bit  Hamamatsu®  C9100-50  EMCCD | closed live-cell  microscopy chamber  (ACU control,  Olympus) for  time-lapse  microscopy | time-lapse  microscopy  & confocal z-  stack imaging |
| Widefield microscope  Axiovert  200 /Zeiss,  Germany | HBO100  mercury  lamp | 488: 473-491 &  506-534  561: 550-580 &  590-650  640: 590-650  & 663-738 | oil immersion  63x Plan-  Apochromat  (NA 1.4) | 12-bit  AxioCam  mRM | - | Multi channel wide-field imaging |
| Leica SP5 II confocal microscope /Wetzlar, Germany | 405 nm diode 488 nm Argon, 561 nm DPSS, 633 nm HeNe | AOBS beam splitter | HCX PL APO 63x / 1.4-0.6 oil lambda blue & HCX PL APO 100x (NA 1.44) oil Corr CS | 2 HyD Hybrid Detectors | - | confocal z- stack imaging |
| Amersham AI600 imager | Chemiluminescence, UV transillumination | - | large aperture FUJINON^TM^ f/0.85 43 mm | 16-bit Peltier cooled Fujifilm Super CCD | - | Western blots and DNA agarose gels |
| Operetta high throughput imaging/ PerkinElmer Life Sciences, UK | Xenon fiber-optic light source, 300 W, 360 – 640 nm continuous spectrum LED light source for transmission mode | ex:360/400, 460/490, 560/580  em: 410/480, 500/550, 560/630 | 20x or 40x air (0.45 NA and 0.95 NA) long WD*** | 14 bit Jenoptik firecamj203 Sony Chip ICX285 cooled 20°C below environment | - | high throughput, high content imaging and image analysis |
| Nikon TiE2 inverted with crest spinning disk unit/ Nikon, Japan | SPECTRA X light engine  395/25 nm with 295 mW  440/20 nm with 256 mW  470/24 nm with 196 mW  510/25 nm with 62 mW  540/30 nm with 231 mW  550/15 nm with 260 mW  575/25 nm with 310 mW | LED-DA/FI/TR/Cy5-4X-B  Quadbandpassex:390/18,  475/35,  535/50  em:460/60,  530/43,  580LP | 40x air (0.95 NA) & 250 µm WD*** | Cooled Nikon Qi2 camera and 16.25 megapixel sCMOS sensor. readout noise is: 2.2. electron | - | high throughput, high content imaging and image analysis |

* ex.: excitation & em.: emission, ** dichroic specification, *** WD: working distance.

**Supplementary file 1f: Data description for DNA quantification**

| **Name** | **Cell stage** | **DAPI SUM** | **Correction factor (C)** | **Corrected genome size (GSxC) Gbp** |
| --- | --- | --- | --- | --- |
| Cell1_crop_DAPI_Cy3dUTP_HeLa | SE | 6.55E+08 | 1.05 | 10.185 |
| Cell2_crop_DAPI_Cy3dUTP_HeLa | SE | 8.49E+08 | 1.05 | 10.185 |
| Cell3_crop_DAPI_Cy3dUTP_HeLa | SE | 9.68E+08 | 1.05 | 10.185 |
| Cell4_crop_DAPI_Cy3dUTP_HeLa | SM | 1.14E+09 | 1.25 | 12.125 |
| Cell5_crop_DAPI_Cy3dUTP_HeLa | SL | 1.15E+09 | 1.77 | 17.169 |
| Cell6_crop_DAPI_Cy3dUTP_HeLa | SE | 8.29E+08 | 1.05 | 10.185 |
| Cell7_crop_DAPI_Cy3dUTP_HeLa | SE | 9.49E+08 | 1.05 | 10.185 |
| Cell8_crop_DAPI_Cy3dUTP_HeLa | SM | 1.01E+09 | 1.25 | 12.125 |
| Cell9_crop_DAPI_Cy3dUTP_HeLa | SM | 1.17E+09 | 1.25 | 12.125 |
| Cell10_crop_DAPI_Cy3dUTP_HeLa | SM | 1.00E+09 | 1.25 | 12.125 |
| Cell11_crop_DAPI_Cy3dUTP_HeLa | SL | 1.34E+09 | 1.77 | 17.169 |
| Cell12_crop_DAPI_Cy3dUTP_HeLa | SM | 1.06E+09 | 1.25 | 12.125 |
| Cell13_crop_DAPI_Cy3dUTP_HeLa | SL | 1.49E+09 | 1.77 | 17.169 |
| Cell14_crop_DAPI_Cy3dUTP_HeLa | SE | 8.71E+08 | 1.05 | 10.185 |
| Cell15_crop_DAPI_Cy3dUTP_HeLa | SL | 1.18E+09 | 1.77 | 17.169 |
| Cell16_crop_DAPI_Cy3dUTP_HeLa | SL | 1.48E+09 | 1.77 | 17.169 |
| Cell17_crop_DAPI_Cy3dUTP_HeLa | SL | 1.09E+09 | 1.77 | 17.169 |
| Cell18_crop_DAPI_Cy3dUTP_HeLa | SL | 1.07E+09 | 1.77 | 17.169 |
| Cell19_crop_DAPI_Cy3dUTP_HeLa | SL | 1.17E+09 | 1.77 | 17.169 |
| Cell20_crop_DAPI_Cy3dUTP_HeLa | SM | 1.09E+09 | 1.25 | 12.125 |
| Cell21_crop_DAPI_Cy3dUTP_HeLa | SL | 1.02E+09 | 1.77 | 17.169 |
| Cell22_crop_DAPI_Cy3dUTP_HeLa | SL | 1.02E+09 | 1.77 | 17.169 |
| Cell23_crop_DAPI_Cy3dUTP_HeLa | SE | 9.33E+08 | 1.05 | 10.185 |
| Cell24_crop_DAPI_Cy3dUTP_HeLa | SE | 8.90E+08 | 1.05 | 10.185 |
| Cell25_crop_DAPI_Cy3dUTP_HeLa | SE | 7.67E+08 | 1.05 | 10.185 |
| Cell26_crop_DAPI_Cy3dUTP_HeLa | SE | 8.39E+08 | 1.05 | 10.185 |
| Cell27_crop_DAPI_Cy3dUTP_HeLa | SM | 1.04E+09 | 1.25 | 12.125 |
| Cell28_crop_DAPI_Cy3dUTP_HeLa | SM | 1.01E+09 | 1.25 | 12.125 |
| Cell29_crop_DAPI_Cy3dUTP_HeLa | SM | 1.05E+09 | 1.25 | 12.125 |
| Cell30_crop_DAPI_Cy3dUTP_HeLa | SM | 1.02E+09 | 1.25 | 12.125 |
| Cell1_DAPI_Cy3dUTP_IMR90 | - | 1.08E+09 | - | 6.37 |
| Cell2_DAPI_Cy3dUTP_IMR90 | - | 5.04E+08 | - | 6.37 |
| Cell3_DAPI_Cy3dUTP_IMR90 | - | 1.09E+09 | - | 6.37 |
| Cell4_DAPI_Cy3dUTP_IMR90 | - | 8.72E+08 | - | 6.37 |
| Cell5_DAPI_Cy3dUTP_IMR90 | - | 1.08E+09 | - | 6.37 |
| Cell6_DAPI_Cy3dUTP_IMR90 | - | 5.39E+08 | - | 6.37 |
| Cell7_DAPI_Cy3dUTP_IMR90 | - | 6.33E+08 | - | 6.37 |
| Cell8_DAPI_Cy3dUTP_IMR90 | - | 5.52E+08 | - | 6.37 |
| Cell9_DAPI_Cy3dUTP_IMR90 | - | 6.20E+08 | - | 6.37 |
| Cell10_DAPI_Cy3dUTP_IMR90 | - | 6.68E+08 | - | 6.37 |
| Cell13_DAPI_Cy3dUTP_IMR90 | - | 7.43E+08 | - | 6.37 |
| Cell14_DAPI_Cy3dUTP_IMR90 | - | 6.53E+08 | - | 6.37 |
| Cell15_DAPI_Cy3dUTP_IMR90 | - | 7.25E+08 | - | 6.37 |
| Cell16_DAPI_Cy3dUTP_IMR90 | - | 4.41E+08 | - | 6.37 |
| Cell17_DAPI_Cy3dUTP_IMR90 | - | 1.29E+09 | - | 6.37 |
| Cell18_DAPI_Cy3dUTP_IMR90 | - | 7.51E+08 | - | 6.37 |

**Supplementary file 1g: Data description for 2D confocal fixed images**

| **Name** | **Cell stage** | **Time (s)** | **Channels*** | **Pixel size (nm)** | **frame rate (ms)** |
| --- | --- | --- | --- | --- | --- |
| Cell1_HeLa_fixedcells.tif | SE | 40 | 3 | 120 | 500 |
| Cell2_HeLa_fixedcells.tif | SE | 40 | 3 | 120 | 500 |
| Cell3_HeLa_fixedcells.tif | SE | 40 | 3 | 120 | 500 |
| Cell4_HeLa_fixedcells.tif | SM | 40 | 3 | 120 | 500 |
| Cell5_HeLa_fixedcells.tif | SM | 40 | 3 | 120 | 500 |
| Cell6_HeLa_fixedcells.tif | SM | 40 | 3 | 120 | 500 |
| Cell7_HeLa_fixedcells.tif | SM | 40 | 3 | 120 | 500 |
| Cell8_HeLa_fixedcells.tif | SL | 40 | 3 | 120 | 500 |
| Cell9_HeLa_fixedcells.tif | SL | 40 | 3 | 120 | 500 |
| Cell10_HeLa_fixedcells.tif | SL | 40 | 3 | 120 | 500 |
| Cell11_HeLa_fixedcells.tif | SE | 40 | 3 | 120 | 500 |
| Cell12_HeLa_fixedcells.tif | SE | 40 | 3 | 120 | 500 |
| Cell13_HeLa_fixedcells.tif | SE | 40 | 3 | 120 | 500 |
| Cell14_HeLa_fixedcells.tif | SL | 40 | 3 | 120 | 500 |
| Cell15_HeLa_fixedcells.tif | SE | 40 | 3 | 120 | 500 |
| Cell16_HeLa_fixedcells.tif | SE | 40 | 3 | 120 | 500 |
| Cell17_HeLa_fixedcells.tif | SL | 40 | 3 | 120 | 500 |
| Cell18_HeLa_fixedcells.tif | G2 | 40 | 3 | 120 | 500 |
| Cell19_HeLa_fixedcells.tif | G1 | 40 | 3 | 120 | 500 |
| Cell20_HeLa_fixedcells.tif | SM | 40 | 3 | 120 | 500 |
| Cell21_HeLa_fixedcells.tif | SE | 40 | 3 | 120 | 500 |
| Cell22_HeLa_fixedcells.tif | SE | 40 | 3 | 120 | 500 |
| Cell23_HeLa_fixedcells.tif | SL | 40 | 3 | 120 | 500 |
| Cell24_HeLa_fixedcells.tif | SM | 40 | 3 | 120 | 500 |
| Cell25_HeLa_fixedcells.tif | SM | 40 | 3 | 120 | 500 |
| Cell26_HeLa_fixedcells.tif | SE | 40 | 3 | 120 | 500 |
| Cell27_HeLa_fixedcells.tif | SE | 40 | 3 | 120 | 500 |
| Cell28_HeLa_fixedcells.tif | SE | 40 | 3 | 120 | 500 |
| Cell29_HeLa_fixedcells.tif | SM | 40 | 3 | 120 | 500 |
| Cell30_HeLa_fixedcells.tif | SE | 40 | 3 | 120 | 500 |
| Cell31_HeLa_fixedcells.tif | SL | 40 | 3 | 120 | 500 |
| Cell32_HeLa_fixedcells.tif | SE | 40 | 3 | 120 | 500 |
| Cell33_HeLa_fixedcells.tif | SE | 40 | 3 | 120 | 500 |
| Cell34_HeLa_fixedcells.tif | SE | 40 | 3 | 120 | 500 |
| Cell35_HeLa_fixedcells.tif | SE | 40 | 3 | 120 | 500 |
| Cell1_IMR90_fixed.tif | - | 40 | 3 | 120 | 500 |
| Cell2_IMR90_fixed.tif | - | 40 | 3 | 120 | 500 |
| Cell3_IMR90_fixed.tif | - | 40 | 3 | 120 | 500 |
| Cell4_IMR90_fixed.tif | - | 40 | 3 | 120 | 500 |
| Cell5_IMR90_fixed.tif | - | 40 | 3 | 120 | 500 |
| Cell6_IMR90_fixed.tif | - | 40 | 3 | 120 | 500 |
| Cell7_IMR90_fixed.tif | - | 40 | 3 | 120 | 500 |
| Cell8_IMR90_fixed.tif | - | 40 | 3 | 120 | 500 |
| Cell9_IMR90_fixed.tif | - | 40 | 3 | 120 | 500 |
| Cell10_IMR90_fixed.tif | - | 40 | 3 | 120 | 500 |
| Cell11_IMR90_fixed.tif | - | 40 | 3 | 120 | 500 |
| Cell12_IMR90_fixed.tif | - | 40 | 3 | 120 | 500 |
| Cell13_IMR90_fixed.tif | - | 40 | 3 | 120 | 500 |
| Cell14_IMR90_fixed.tif | - | 40 | 3 | 120 | 500 |
| Cell15_IMR90_fixed.tif | - | 40 | 3 | 120 | 500 |
| Cell16_IMR90_fixed.tif | - | 40 | 3 | 120 | 500 |
| Cell17_IMR90_fixed.tif | - | 40 | 3 | 120 | 500 |
| Cell18_IMR90_fixed.tif | - | 40 | 3 | 120 | 500 |

**Supplementary file 1h: Data description for 2D confocal live images**

| **Name** | **Cell stage** | **Time**  **(s)** | **Exposure time**  **(ms)** | **Channels*** | **Frame rate**  **(ms)** |
| --- | --- | --- | --- | --- | --- |
| n001_G_Aph_4_AT_c01.tif | G1 | 40 | 300 | 1 | 500 |
| n001_G_Aph_4_AT_c02.tif | G1 | - | 500 | 3 | - |
| n001_G_Aph_4_BT_c01.tif | G1 | 40 | 300 | 1 | 500 |
| n001_G_Aph_4_BT_c02.tif | G1 | - | 500 | 3 | - |
| n002_G_Aph_4_AT_c01.tif | G2 | 40 | 300 | 1 | 500 |
| n002_G_Aph_4_AT_c02.tif | G2 | - | 500 | 3 | - |
| n002_G_Aph_4_BT_c01.tif | G2 | 40 | 300 | 1 | 500 |
| n002_G_Aph_4_BT_c02.tif | G2 | - | 500 | 3 | - |
| n003_G_Aph_5_AT_c01.tif | G2 | 40 | 300 | 1 | 500 |
| n003_G_Aph_5_AT_c02.tif | G2 | - | 500 | 3 | - |
| n003_G_Aph_5_BT_c01.tif | G2 | 40 | 300 | 1 | 500 |
| n003_G_Aph_5_BT_c02.tif | G2 | - | 500 | 3 | - |
| n004_G_Aph_5_AT_c01.tif | G2 | 40 | 300 | 1 | 500 |
| n004_G_Aph_5_AT_c02.tif | G2 | - | 500 | 3 | - |
| n004_G_Aph_5_BT_c01.tif | G2 | 40 | 300 | 1 | 500 |
| n004_G_Aph_5_BT_c02.tif | G2 | - | 500 | 3 | - |
| n005_G_Aph_5_AT_c01.tif | G1 | 40 | 300 | 1 | 500 |
| n005_G_Aph_5_AT_c02.tif | G1 | - | 500 | 3 | - |
| n005_G_Aph_5_BT_c01.tif | G1 | 40 | 300 | 1 | 500 |
| n005_G_Aph_5_BT_c02.tif | G1 | - | 500 | 3 | - |
| n006_G_Aph_6_AT_c01.tif | G2 | 40 | 300 | 1 | 500 |
| n006_G_Aph_6_AT_c02.tif | G2 | - | 500 | 3 | - |
| n006_G_Aph_6_BT_c01.tif | G2 | 40 | 300 | 1 | 500 |
| n006_G_Aph_6_BT_c02.tif | G2 | - | 500 | 3 | - |
| n007_G_Aph_10_AT_c01.tif | G1 | 40 | 300 | 1 | 500 |
| n007_G_Aph_10_AT_c02.tif | G1 | - | 500 | 3 | - |
| n007_G_Aph_10_BT_c01.tif | G1 | 40 | 300 | 1 | 500 |
| n007_G_Aph_10_BT_c02.tif | G1 | - | 500 | 3 | - |
| n008_G_Aph_10_AT_c01.tif | G1 | 40 | 300 | 1 | 500 |
| n008_G_Aph_10_AT_c02.tif | G1 | - | 500 | 3 | - |
| n008_G_Aph_10_BT_c01.tif | G1 | 40 | 300 | 1 | 500 |
| n008_G_Aph_10_BT_c02.tif | G1 | - | 500 | 3 | - |
| n009_G_Aph_10_AT_c01.tif | G1 | 40 | 300 | 1 | 500 |
| n009_G_Aph_10_AT_c02.tif | G1 | - | 500 | 3 | - |
| n009_G_Aph_10_BT_c01.tif | G1 | 40 | 300 | 1 | 500 |
| n009_G_Aph_10_BT_c02.tif | G1 | - | 500 | 3 | - |
| n0012_G_Aph_11_AT_c01.tif | G2 | 40 | 300 | 1 | 500 |
| n0012_G_Aph_11_AT_c02.tif | G2 | - | 500 | 3 | - |
| n0012_G_Aph_11_BT_c01.tif | G2 | 40 | 300 | 1 | 500 |
| n0012_G_Aph_11_BT_c02.tif | G2 | - | 500 | 3 | - |
| n0013_G_Aph_11_AT_c01.tif | G2 | 40 | 300 | 1 | 500 |
| n0013_G_Aph_11_AT_c02.tif | G2 | - | 500 | 3 | - |
| n0013_G_Aph_11_BT_c01.tif | G2 | 40 | 300 | 1 | 500 |
| n0013_G_Aph_11_BT_c02.tif | G2 | - | 500 | 3 | - |
| n0015_G_Aph_15_AT_c01.tif | G1 | 40 | 300 | 1 | 500 |
| n0015_G_Aph_15_AT_c02.tif | G1 | - | 500 | 3 | - |
| n0015_G_Aph_15_BT_c01.tif | G1 | 40 | 300 | 1 | 500 |
| n0015_G_Aph_15_BT_c02.tif | G1 | - | 500 | 3 | - |
| n0017_G_Aph_16_AT_c01.tif | G1 | 40 | 300 | 1 | 500 |
| n0017_G_Aph_16_AT_c02.tif | G1 | - | 500 | 3 | - |
| n0017_G_Aph_16_BT_c01.tif | G1 | 40 | 300 | 1 | 500 |
| n0017_G_Aph_16_BT_c02.tif | G1 | - | 500 | 3 | - |
| n0018_G_Aph_18_AT_c01.tif | G1 | 40 | 300 | 1 | 500 |
| n0018_G_Aph_18_AT_c02.tif | G1 | - | 500 | 3 | - |
| n0018_G_Aph_18_BT_c01.tif | G1 | 40 | 300 | 1 | 500 |
| n0018_G_Aph_18_BT_c02.tif | G1 | - | 500 | 3 | - |
| n0020_G_Aph_21_AT_c01.tif | G2 | 40 | 300 | 1 | 500 |
| n0020_G_Aph_21_AT_c02.tif | G2 | - | 500 | 3 | - |
| n0020_G_Aph_21_BT_c01.tif | G2 | 40 | 300 | 1 | 500 |
| n0020_G_Aph_21_BT_c02.tif | G2 | - | 500 | 3 | - |
| n001_S_Aph_1_AT_c01.tif | S | 40 | 300 | 1 | 500 |
| n001_S_Aph_1_AT_c02.tif | S | - | 500 | 3 | - |
| n001_S_Aph_1_BT_c01.tif | S | 40 | 300 | 1 | 500 |
| n001_S_Aph_1_BT_c02.tif | S | - | 500 | 3 | - |
| n002_S_Aph_1_AT_c01.tif | S | 40 | 300 | 1 | 500 |
| n002_S_Aph_1_AT_c02.tif | S | - | 500 | 3 | - |
| n002_S_Aph_1_BT_c01.tif | S | 40 | 300 | 1 | 500 |
| n002_S_Aph_1_BT_c02.tif | S | - | 500 | 3 | - |
| n004_S_Aph_1_AT_c01.tif | S | 40 | 300 | 1 | 500 |
| n004_S_Aph_1_AT_c02.tif | S | - | 500 | 3 | - |
| n004_S_Aph_1_BT_c01.tif | S | 40 | 300 | 1 | 500 |
| n004_S_Aph_1_BT_c02.tif | S | - | 500 | 3 | - |
| n006_S_Aph_2_AT_c01.tif | S | 40 | 300 | 1 | 500 |
| n006_S_Aph_2_AT_c02.tif | S | - | 500 | 3 | - |
| n006_S_Aph_2_BT_c01.tif | S | 40 | 300 | 1 | 500 |
| n006_S_Aph_2_BT_c02.tif | S | - | 500 | 3 | - |
| n008_S_Aph_4_AT_c01.tif | S | 40 | 300 | 1 | 500 |
| n008_S_Aph_4_AT_c02.tif | S | - | 500 | 3 | - |
| n008_S_Aph_4_BT_c01.tif | S | 40 | 300 | 1 | 500 |
| n008_S_Aph_4_BT_c02.tif | S | - | 500 | 3 | - |
| n009_S_Aph_5_AT_c01.tif | S | 40 | 300 | 1 | 500 |
| n009_S_Aph_5_AT_c02.tif | S | - | 500 | 3 | - |
| n009_S_Aph_5_BT_c01.tif | S | 40 | 300 | 1 | 500 |
| n009_S_Aph_5_BT_c02.tif | S | - | 500 | 3 | - |
| n0012_S_Aph_6_AT_c01.tif | S | 40 | 300 | 1 | 500 |
| n0012_S_Aph_6_AT_c02.tif | S | - | 500 | 3 | - |
| n0012_S_Aph_6_BT_c01.tif | S | 40 | 300 | 1 | 500 |
| n0012_S_Aph_6_BT_c02.tif | S | - | 500 | 3 | - |
| n0013_S_Aph_7_AT_c01.tif | S | 40 | 300 | 1 | 500 |
| n0013_S_Aph_7_AT_c02.tif | S | - | 500 | 3 | - |
| n0013_S_Aph_7_BT_c01.tif | S | 40 | 300 | 1 | 500 |
| n0013_S_Aph_7_BT_c02.tif | S | - | 500 | 3 | - |
| n0014_S_Aph_7_AT_c01.tif | S | 40 | 300 | 1 | 500 |
| n0014_S_Aph_7_AT_c02.tif | S | - | 500 | 3 | - |
| n0014_S_Aph_7_BT_c01.tif | S | 40 | 300 | 1 | 500 |
| n0014_S_Aph_7_BT_c02.tif | S | - | 500 | 3 | - |
| n0015_S_Aph_8_AT_c01.tif | S | 40 | 300 | 1 | 500 |
| n0015_S_Aph_8_AT_c02.tif | S | - | 500 | 3 | - |
| n0015_S_Aph_8_BT_c01.tif | S | 40 | 300 | 1 | 500 |
| n0015_S_Aph_8_BT_c02.tif | S | - | 500 | 3 | - |
| n0016_S_Aph_8_AT_c01.tif | S | 40 | 300 | 1 | 500 |
| n0016_S_Aph_8_AT_c02.tif | S | - | 500 | 3 | - |
| n0016_S_Aph_8_BT_c01.tif | S | - | 500 | 3 | 500 |
| n0016_S_Aph_8_BT_c02.tif | S | 40 | 300 | 1 | - |
| n0017_S_Aph_8_AT_c01.tif | S | - | 500 | 3 | 500 |
| n0017_S_Aph_8_AT_c02.tif | S | 40 | 300 | 1 | - |
| n0017_S_Aph_8_BT_c01.tif | S | - | 500 | 3 | 500 |
| n0017_S_Aph_8_BT_c02.tif | S | 40 | 300 | 1 | - |
| n0018_S_Aph_8_AT_c01.tif | S | - | 500 | 3 | 500 |
| n0018_S_Aph_8_AT_c02.tif | S | 40 | 300 | 1 | - |
| n0018_S_Aph_8_BT_c01.tif | S | - | 500 | 3 | 500 |
| n0018_S_Aph_8_BT_c02.tif | S | 40 | 300 | 1 | - |
| n0019_S_Aph_9_AT_c01.tif | S | - | 500 | 3 | 500 |
| n0019_S_Aph_9_AT_c02.tif | S | 40 | 300 | 1 | - |
| n0019_S_Aph_9_BT_c01.tif | S | - | 500 | 3 | 500 |
| n0019_S_Aph_9_BT_c02.tif | S | - | 500 | 3 | - |
| n0020_S_Aph_9_AT_c01.tif | S | 40 | 300 | 1 | 500 |
| n0020_S_Aph_9_AT_c02.tif | S | - | 500 | 3 | - |
| n0020_S_Aph_9_BT_c01.tif | S | 40 | 300 | 1 | 500 |
| n0020_S_Aph_9_BT_c02.tif | S | - | 500 | 3 | - |
| n0021_S_Aph_1_0_AT_c01.tif | S | 40 | 300 | 1 | 500 |
| n0021_S_Aph_1_0_AT_c02.tif | S | - | 500 | 3 | - |
| n0021_S_Aph_1_0_BT_c01.tif | S | 40 | 300 | 1 | 500 |
| n0021_S_Aph_1_0_BT_c02.tif | S | - | 500 | 3 | - |
| n0022_S_Aph_1_2_AT_c01.tif | S | 40 | 300 | 1 | 500 |
| n0022_S_Aph_1_2_AT_c02.tif | S | - | 500 | 3 | - |
| n0022_S_Aph_1_2_BT_c01.tif | S | 40 | 300 | 1 | 500 |
| n0022_S_Aph_1_2_BT_c02.tif | S | - | 500 | 3 | - |
| n0023_S_Aph_1_3_AT_c01.tif | S | 40 | 300 | 1 | 500 |
| n0023_S_Aph_1_3_AT_c02.tif | S | - | 500 | 3 | - |
| n0023_S_Aph_1_3_BT_c01.tif | S | 40 | 300 | 1 | 500 |
| n0023_S_Aph_1_3_BT_c02.tif | S | - | 500 | 3 | - |
| n0024_S_Aph_1_4_AT_c01.tif | S | 40 | 300 | 1 | 500 |
| n0024_S_Aph_1_4_AT_c02.tif | S | - | 500 | 3 | - |
| n0024_S_Aph_1_4_BT_c01.tif | S | 40 | 300 | 1 | 500 |
| n0024_S_Aph_1_4_BT_c02.tif | S | - | 500 | 3 | - |
| n0025_S_Aph_1_7_AT_c01.tif | S | 40 | 300 | 1 | 500 |
| n0025_S_Aph_1_7_AT_c02.tif | S | - | 500 | 3 | - |
| n0025_S_Aph_1_7_BT_c01.tif | S | 40 | 300 | 1 | 500 |
| n0025_S_Aph_1_7_BT_c02.tif | S | - | 500 | 3 | - |
| n0026_S_Aph_1_7_AT_c01.tif | S | 40 | 300 | 1 | 500 |
| n0026_S_Aph_1_7_AT_c02.tif | S | - | 500 | 3 | - |
| n0026_S_Aph_1_7_BT_c01.tif | S | 40 | 300 | 1 | 500 |
| n0026_S_Aph_1_7_BT_c02.tif | S | - | 500 | 3 | - |
| n0027_S_Aph_21_AT_c01.tif | S | 40 | 300 | 1 | 500 |
| n0027_S_Aph_21_AT_c02.tif | S | - | 500 | 3 | - |
| n0027_S_Aph_21_BT_c01.tif | S | 40 | 300 | 1 | 500 |
| n0027_S_Aph_21_BT_c02.tif | S | - | 500 | 3 | - |
| n0028_S_Aph_21_AT_c01.tif | S | 40 | 300 | 1 | 500 |
| n0028_S_Aph_21_AT_c02.tif | S | - | 500 | 3 | - |
| n0028_S_Aph_21_BT_c01.tif | S | 40 | 300 | 1 | 500 |
| n0028_S_Aph_21_BT_c02.tif | S | - | 500 | 3 | - |
| n0001_coloc_c02.tif | S | 40 | 300 | 2 | 500 |
| n0002_coloc_c02.tif | S | 40 | 300 | 2 | 500 |
| n0003_coloc_c02.tif | S | 40 | 300 | 2 | 500 |
| n0004_coloc_c02.tif | S | 40 | 300 | 2 | 500 |
| n0005_coloc_c02.tif | S | 40 | 300 | 2 | 500 |
| n0006_coloc_c02.tif | S | 40 | 300 | 2 | 500 |
| n0007_coloc_c02.tif | S | 40 | 300 | 2 | 500 |
| n0008_coloc_c02.tif | S | 40 | 300 | 2 | 500 |
| n0009_coloc_c02.tif | S | 40 | 300 | 2 | 500 |
| n0010_coloc_c02.tif | S | 40 | 300 | 2 | 500 |
| n0011_coloc_c02.tif | S | 40 | 300 | 2 | 500 |
| n0012_coloc_c02.tif | S | 40 | 300 | 2 | 500 |
| n0013_coloc_c02.tif | S | 40 | 300 | 2 | 500 |
| n0014_coloc_c02.tif | S | 40 | 300 | 2 | 500 |
| n0015_coloc_c02.tif | S | 40 | 300 | 2 | 500 |
| n0016_coloc_c02.tif | S | 40 | 300 | 2 | 500 |
| n0017_coloc_c02.tif | S | 40 | 300 | 2 | 500 |
| n0018_coloc_c02.tif | S | 40 | 300 | 2 | 500 |
| n0019_coloc_c02.tif | S | 40 | 300 | 2 | 500 |
| n0020_coloc_c02.tif | S | 40 | 300 | 2 | 500 |
| n0021_coloc_c02.tif | S | 40 | 300 | 2 | 500 |
| n0022_coloc_c02.tif | S | 40 | 300 | 2 | 500 |
| n0023_coloc_c02.tif | S | 40 | 300 | 2 | 500 |
| n0024_coloc_c02.tif | S | 40 | 300 | 2 | 500 |
| n0025_coloc_c02.tif | S | 40 | 300 | 2 | 500 |
| n0026_coloc_c02.tif | S | 40 | 300 | 2 | 500 |
| n0027_coloc_c02.tif | S | 40 | 300 | 2 | 500 |
| n0028_coloc_c02.tif | S | 40 | 300 | 2 | 500 |
| n0029_coloc_c02.tif | S | 40 | 300 | 2 | 500 |
| n0030_coloc_c02.tif | S | 40 | 300 | 2 | 500 |
| n0031_coloc_c02.tif | S | 40 | 300 | 2 | 500 |
| n0032_coloc_c02.tif | S | 40 | 300 | 2 | 500 |
| n0033_coloc_c02.tif | S | 40 | 300 | 2 | 500 |
| n001_G1_IMR90_PCNA_Cy3dUTP_60x.tif | G1 | 40 | 300 | 2 | 500 |
| n002_G1_IMR90_PCNA_Cy3dUTP_60x.tif | G1 | 40 | 300 | 2 | 500 |
| n003_G1_IMR90_PCNA_Cy3dUTP_60x.tif | G1 | 40 | 300 | 2 | 500 |
| n004_G1_IMR90_PCNA_Cy3dUTP_60x.tif | G1 | 40 | 300 | 2 | 500 |
| n005_G1_IMR90_PCNA_Cy3dUTP_60x.tif | G1 | 40 | 300 | 2 | 500 |
| n006_G1_IMR90_PCNA_Cy3dUTP_60x.tif | G1 | 40 | 300 | 2 | 500 |
| n007_G1_IMR90_PCNA_Cy3dUTP_60x.tif | G1 | 40 | 300 | 2 | 500 |
| n008_G1_IMR90_PCNA_Cy3dUTP_60x.tif | G1 | 40 | 300 | 2 | 500 |
| n009_G1_IMR90_PCNA_Cy3dUTP_60x.tif | G1 | 40 | 300 | 2 | 500 |
| n010_G1_IMR90_PCNA_Cy3dUTP_60x.tif | G1 | 40 | 300 | 2 | 500 |
| n011_G1_IMR90_PCNA_Cy3dUTP_60x.tif | G1 | 40 | 300 | 2 | 500 |
| n012_G1_IMR90_PCNA_Cy3dUTP_60x.tif | G1 | 40 | 300 | 2 | 500 |
| n013_G1_IMR90_PCNA_Cy3dUTP_60x.tif | G1 | 40 | 300 | 2 | 500 |
| n014_G1_IMR90_PCNA_Cy3dUTP_60x.tif | G1 | 40 | 300 | 2 | 500 |
| n001_G2_IMR90_PCNA_Cy3dUTP_60x.tif | G2 | 40 | 300 | 2 | 500 |
| n002_G2_IMR90_PCNA_Cy3dUTP_60x.tif | G2 | 40 | 300 | 2 | 500 |
| n003_G2_IMR90_PCNA_Cy3dUTP_60x.tif | G2 | 40 | 300 | 2 | 500 |
| n004_G2_IMR90_PCNA_Cy3dUTP_60x.tif | G2 | 40 | 300 | 2 | 500 |
| n005_G2_IMR90_PCNA_Cy3dUTP_60x.tif | G2 | 40 | 300 | 2 | 500 |
| n006_G2_IMR90_PCNA_Cy3dUTP_60x.tif | G2 | 40 | 300 | 2 | 500 |
| n007_G2_IMR90_PCNA_Cy3dUTP_60x.tif | G2 | 40 | 300 | 2 | 500 |
| n008_G2_IMR90_PCNA_Cy3dUTP_60x.tif | G2 | 40 | 300 | 2 | 500 |
| n009_G2_IMR90_PCNA_Cy3dUTP_60x.tif | G2 | 40 | 300 | 2 | 500 |
| n010_G2_IMR90_PCNA_Cy3dUTP_60x.tif | G2 | 40 | 300 | 2 | 500 |
| n011_G2_IMR90_PCNA_Cy3dUTP_60x.tif | G2 | 40 | 300 | 2 | 500 |
| n012_G2_IMR90_PCNA_Cy3dUTP_60x.tif | G2 | 40 | 300 | 2 | 500 |
| n013_G2_IMR90_PCNA_Cy3dUTP_60x.tif | G2 | 40 | 300 | 2 | 500 |
| n014_G2_IMR90_PCNA_Cy3dUTP_60x.tif | G2 | 40 | 300 | 2 | 500 |
| n015_G2_IMR90_PCNA_Cy3dUTP_60x.tif | G2 | 40 | 300 | 2 | 500 |
| n001_S_IMR90_PCNA_Cy3dUTP_60x.tif | S | 40 | 300 | 2 | 500 |
| n002_S_IMR90_PCNA_Cy3dUTP_60x.tif | S | 40 | 300 | 2 | 500 |
| n003_S_IMR90_PCNA_Cy3dUTP_60x.tif | S | 40 | 300 | 2 | 500 |
| n004_S_IMR90_PCNA_Cy3dUTP_60x.tif | S | 40 | 300 | 2 | 500 |
| n005_S_IMR90_PCNA_Cy3dUTP_60x.tif | S | 40 | 300 | 2 | 500 |
| n006_S_IMR90_PCNA_Cy3dUTP_60x.tif | S | 40 | 300 | 2 | 500 |
| n007_S_IMR90_PCNA_Cy3dUTP_60x.tif | S | 40 | 300 | 2 | 500 |
| n008_S_IMR90_PCNA_Cy3dUTP_60x.tif | S | 40 | 300 | 2 | 500 |
| n009_S_IMR90_PCNA_Cy3dUTP_60x.tif | S | 40 | 300 | 2 | 500 |
| n010_S_IMR90_PCNA_Cy3dUTP_60x.tif | S | 40 | 300 | 2 | 500 |
| n011_S_IMR90_PCNA_Cy3dUTP_60x.tif | S | 40 | 300 | 2 | 500 |
| n012_S_IMR90_PCNA_Cy3dUTP_60x.tif | S | 40 | 300 | 2 | 500 |
| n0013_S_IMR90_PCNA_Cy3dUTP_60x.tif | S | 40 | 300 | 2 | 500 |
| n0014_S_IMR90_PCNA_Cy3dUTP_60x.tif | S | 40 | 300 | 2 | 500 |

**Supplementary file 1i: Software and macros**

| **Name** | **Version** | **Website** | **Company/University** | **Application** |
| --- | --- | --- | --- | --- |
| Volocity | 6.3 | - | PerkinElmer, USA | Acquiring live cell time lapses |
| ImageJ | 1.53c | <https://imagej.nih.gov/ij/> | Wayne Rasband, National Institutes of Health, USA | Image processing and image analysis |
| RStudio | 1.1.447-1.2.5033 | <https://rstudio.com/> | RStudio | Statistical analysis and plotting |
| Harmony | 3.5.1 | <https://www.perkinelmer.com/product/harmony-4-8-office-hh17000001> | PerkinElmer, USA | High content microscopy imaging and analysis |
| KNIME Analytics Platform | 3.5.2 | https://www.knime.com/knime-analytics-platform | KNIME AG, Switzerland | High content microscopy image processing and analysis |
| Adobe Illustrator CS6 | 16 | <https://www.adobe.com/> | Adobe, USA | Graphical sketch and figures arrangement |

**Supplementary references:**

[Chagin VO, Casas-Delucchi CS, Reinhart M, Schermelleh L, Markaki Y, Maiser A, Bolius JJ, Bensimon A, Fillies M, Domaing P, Rozanov YM, Leonhardt H, Cardoso MC. 2016. 4D Visualization of replication foci in mammalian cells corresponding to individual replicons. *Nat Commun* **7**:11231. doi:10.1038/ncomms11231](https://sciwheel.com/work/bibliography/2069726)

[Erfle H, Neumann B, Liebel U, Rogers P, Held M, Walter T, Ellenberg J, Pepperkok R. 2007. Reverse transfection on cell arrays for high content screening microscopy. *Nat Protoc* **2**:392–399. doi:10.1038/nprot.2006.483](https://sciwheel.com/work/bibliography/1001802)

[Kenny MK, Schlegel U, Furneaux H, Hurwitz J. 1990. The role of human single-stranded DNA binding protein and its individual subunits in simian virus 40 DNA replication. *J Biol Chem* **265**:7693–7700. doi:10.1016/S0021-9258(19)39170-7](https://sciwheel.com/work/bibliography/1287800)

[Li CM, Miao Y, Lingeman RG, Hickey RJ, Malkas LH. 2016. Partial purification of a megadalton DNA replication complex by free flow electrophoresis. *PLoS ONE* **11**:e0169259. doi:10.1371/journal.pone.0169259](https://sciwheel.com/work/bibliography/3106104)

[Nichols WW, Murphy DG, Cristofalo VJ, Toji LH, Greene AE, Dwight SA. 1977. Characterization of a new human diploid cell strain, IMR-90. *Science* **196**:60–63. doi:10.1126/science.841339](https://sciwheel.com/work/bibliography/8508791)

[Rausch C, Zhang P, Casas-Delucchi CS, Daiß JL, Engel C, Coster G, Hastert FD, Weber P, Cardoso MC. 2021. Cytosine base modifications regulate DNA duplex stability and metabolism. *Nucleic Acids Res* **49**:12870–12894. doi:10.1093/nar/gkab509](https://sciwheel.com/work/bibliography/11830976)

[Tanaka S, Hu SZ, Wang TS, Korn D. 1982. Preparation and preliminary characterization of monoclonal antibodies against human DNA polymerase alpha. *J Biol Chem* **257**:8386–8390. doi:10.1016/S0021-9258(18)34343-6](https://sciwheel.com/work/bibliography/2084999)

[Waseem NH, Lane DP. 1990. Monoclonal antibody analysis of the proliferating cell nuclear antigen (PCNA). Structural conservation and the detection of a nucleolar form. *J Cell Sci* **96 ( Pt 1)**:121–129. doi:10.1242/jcs.96.1.121](https://sciwheel.com/work/bibliography/2085678)
